# Chemoenzymatic synthesis of daptomycin analogs active against daptomycin-resistant strains

**DOI:** 10.1007/s00253-020-10790-x

**Published:** 2020-07-28

**Authors:** Erin M. Scull, Chandrasekhar Bandari, Bryce P. Johnson, Eric D. Gardner, Marco Tonelli, Jianlan You, Robert H. Cichewicz, Shanteri Singh

**Affiliations:** 1 Department of Chemistry and Biochemistry, Stephenson Life Sciences Research Center, University of Oklahoma, 101 Stephenson Parkway, Norman, OK 73019 USA; 2 National Magnetic Resonance Facility at Madison, University of Wisconsin-Madison, 411 Babcock Drive, Madison, WI 45005 USA

**Keywords:** Biocatalysis, Enzyme promiscuity, Tryptophan, Indole, Daptomycin

## Abstract

**Abstract:**

Daptomycin is a last resort antibiotic for the treatment of infections caused by many Gram-positive bacterial strains, including vancomycin-resistant *Enterococcus* (VRE) and methicillin- and vancomycin-resistant *Staphylococcus aureus* (MRSA and VRSA). However, the emergence of daptomycin-resistant strains of *S. aureus* and *Enterococcus* in recent years has renewed interest in synthesizing daptomycin analogs to overcome resistance mechanisms. Within this context, three aromatic prenyltransferases have been shown to accept daptomycin as a substrate, and the resulting prenylated analog was shown to be more potent against Gram-positive strains than the parent compound. Consequently, utilizing prenyltransferases to derivatize daptomycin offered an attractive alternative to traditional synthetic approaches, especially given the molecule’s structural complexity. Herein, we report exploiting the ability of prenyltransferase CdpNPT to synthesize alkyl-diversified daptomycin analogs in combination with a library of synthetic non-native alkyl-pyrophosphates. The results revealed that CdpNPT can transfer a variety of alkyl groups onto daptomycin’s tryptophan residue using the corresponding alkyl-pyrophosphates, while subsequent scaled-up reactions suggested that the enzyme can alkylate the N1, C2, C5, and C6 positions of the indole ring. In vitro antibacterial activity assays using 16 daptomycin analogs revealed that some of the analogs displayed 2–80-fold improvements in potency against MRSA, VRE, and daptomycin-resistant strains of *S. aureus* and *Enterococcus faecalis*. Thus, along with the new potent analogs, these findings have established that the regio-chemistry of alkyl substitution on the tryptophan residue can modulate daptomycin’s potency. With additional protein engineering to improve the regio-selectivity, the described method has the potential to become a powerful tool for diversifying complex indole-containing molecules.

**Key points:**

*• CdpNPT displays impressive donor promiscuity with daptomycin as the acceptor.*

*• CdpNPT catalyzes N1-, C2-, C5-, and C6-alkylation on daptomycin’s tryptophan residue.*

*• Differential alkylation of daptomycin’s tryptophan residue modulates its activity.*

**Electronic supplementary material:**

The online version of this article (10.1007/s00253-020-10790-x) contains supplementary material, which is available to authorized users.

## Introduction

**Fig. 1:**
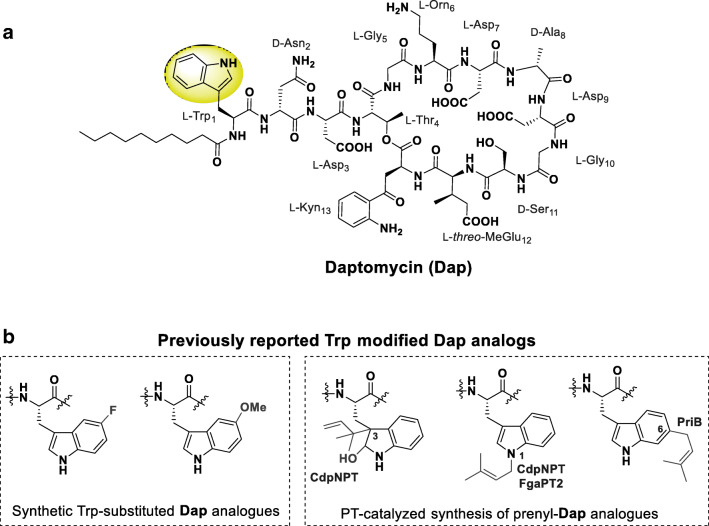
**a** Structure of Dap highlighting Trp residue of interest to the current study in yellow. **b** Structures of previously reported Trp-modified analogs of Dap (Elshahawi et al. 2017; He et al. 2012)

Daptomycin (**Dap**, Fig. 1a) belongs to a family of calcium-dependent lipodepsipeptide antibiotics produced by *Streptomyces roseosporus* (Debono et al. 1987; Miao et al. 2005). It is active against several resistant strains of Gram-positive bacteria, including methicillin-resistant *Staphylococcus aureus* (MRSA), vancomycin-intermediate *Staphylococcus aureus* (VISA), vancomycin-resistant *Staphylococcus aureus* (VRSA), and vancomycin-resistant *Enterococci* (VRE) (Steenbergen et al. 2005; Tally and Debruin 2000). As such, **Dap** (Cubicin®) was approved in 2003 for the treatment of complicated skin and soft tissue infections, and subsequently in 2006 for *S. aureus* bacteremia and right-sided infective endocarditis (Arbeit et al. 2004; Chan Tompkins and Harnicar 2008; Eisenstein et al. 2010; Fowler et al. 2006; Sakoulas 2009). **Dap** is also one of the most promising drugs currently under evaluation for the treatment of pneumococcal meningitis (Cottagnoud et al. 2004; Muri et al. 2018), and while it is active against *Streptococcus pneumoniae*, it does not meet non-inferiority criteria for the treatment of community-acquired pneumonia due to inhibition by lung surfactants (Silverman et al. 2005). These potent antibacterial activities have been traced back to **Dap**’s complex structure (Fig. 1a) leading to its unique mechanism of action (MOA), which has recently been shown to involve binding to regions of increased fluidity (RIFs) within the Gram-positive cell membrane (Müller et al. 2016). **Dap** was believed to rigidify the RIFs immediately upon binding and thereby interrupt protein-lipid interactions that rely on the regions’ increased fluidity (Gray and Wenzel 2020; Müller et al. 2016; Pogliano et al. 2012). De-anchoring of the lipid II synthase MurG and the phospholipid synthase PlsX was proposed to hinder synthesis of the cell wall and outer membrane, respectively, resulting in **Dap**’s bactericidal effect (Gray and Wenzel 2020; Müller et al. 2016).

However, several key steps in this process remain unresolved, including the involvement of oligomers in membrane permeabilization (Taylor et al. 2017; Zhang et al. 2014). Furthermore, the emergence of **Dap**-resistant (**Dap**R) *S. aureus* and *Enterococcus* (Bayer et al. 2013; Boudjemaa et al. 2018; Goméz Casanova et al. 2017; Pader et al. 2016; Vilhena and Bettencourt 2012) has provided further impetus for developing new derivatives (Barnawi et al. 2018; Doekel et al. 2008; Grünewald et al. 2004; He et al. 2012; Hill et al. 2003; Lam et al. 2013; Lin et al. 2016; Lohani et al. 2015a; Lohani et al. 2015b; Miao et al. 2006; Robbel and Marahiel 2010; Siedlecki et al. 2003) to pursue more extensive structure-activity relationship (SAR) studies and circumvent resistance mechanisms. Several biosynthetic (Doekel et al. 2008; Miao et al. 2006; Robbel and Marahiel 2010), chemoenzymatic (Grünewald et al. 2004), and solid- and solution-phase strategies (Barnawi et al. 2018; He et al. 2012; Hill et al. 2003; Lam et al. 2013; Lin et al. 2016; Lohani et al. 2015a; Lohani et al. 2015b; Siedlecki et al. 2003) have indeed been developed for **Dap** synthesis and optimization. Despite this progress, only a limited variety of analogs have been prepared, and most of them encompass specific amino acid mutations and modifications of the lipid chain or the δ-amino group of ornithine (Barnawi et al. 2018; Doekel et al. 2008; Grünewald et al. 2004; Hill et al. 2003; Lam et al. 2013; Lin et al. 2016; Lohani et al. 2015a; Lohani et al. 2015b; Miao et al. 2006; Robbel and Marahiel 2010; Siedlecki et al. 2003; Yin et al. 2015). While some acyl-modified analogs of **Dap** exhibited improved in vitro activity against **Dap**R strains (Yin et al. 2015), a new strategy with the ability to provide additional structural diversity will enable further optimization of the **Dap** scaffold and the generation of even more analogs potent against **Dap**R bacteria.

In moving toward such strategies, recent studies have demonstrated replacement of Trp1 on **Dap** (Fig. 1b) with certain unnatural aromatic amino acids, and/or substitution on the indole ring of Trp1, resulted in analogs with activity against *S. aureus* (Elshahawi et al. 2017; He et al. 2012). These studies thus establish Trp1 as an important target within **Dap** for analog synthesis. However, a systematic study of Trp1-based modifications has remained elusive due to the lack of efficient synthetic strategies for such complex scaffolds. Therefore, a new method is needed in general to introduce additional diversity on **Dap** and enable systematic SAR studies**,** while one capable of selectively and efficiently modifying the Trp on **Dap** is perhaps even more urgently needed.

Within this context, a class of natural product enzymes known as aromatic prenyltransferases (PTs) demonstrates broad substrate specificities and is known to modify diverse positions of the indole moiety within complex natural products (Elshahawi et al. 2017; Tanner 2015; Tello et al. 2008). Furthermore, three indole PTs (FgaPT2, CdpNPT, and PriB) have been shown to specifically prenylate **Dap** using dimethylallyl pyrophosphate as the prenyl donor (Elshahawi et al. 2017). Among the three, CdpNPT demonstrated the highest yield of total product synthesis, and the reaction’s major product (*N1*-Trp-prenyl-**Dap**) demonstrated higher potency against *S. aureus* compared with the parent compound (Elshahawi et al. 2017). Because PTs are also known to display donor substrate flexibilities (Bandari et al. 2019; Bandari et al. 2017; Johnson et al. 2020; Liebhold and Li 2013; Liebhold et al. 2012; Liebhold et al. 2013; Winkelblech et al. 2015; Yu et al. 2015), we hypothesized that **Dap** diversification could be achieved if CdpNPT utilized a library of non-native alkyl-pyrophosphates (alkyl-PPs) with **Dap** as an acceptor. If so, the system would provide facile access to a previously unexplored chemical space for **Dap**, as well as an opportunity to explore the SAR of Trp1-modified **Dap** analogs.

**Fig. 2:**
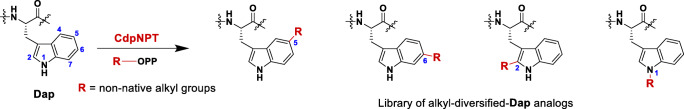
This study involving utilization of CdpNPT in the chemoenzymatic generation of Dap analogs with non-native alkyl-PPs (R-OPPs)

Therefore, we report herein the expansion of the alkyl-donor substrate scope of the indole PT CdpNPT from *Aspergillus fumigatus* (Schuller et al. 2012) using 38 non-native alkyl-donors and **Dap** as an acceptor. We demonstrate CdpNPT to have exceptional flexibility toward alkyl groups and thus present the first reported example of PT-catalyzed alkyl-diversification of **Dap** using unnatural donors. Furthermore, 16 alkyl-diversified-**Dap** analogs were scaled-up, purified, and characterized on a semi-preparative scale, confirming alkyl substitution at the N1, C2, C5, or C6 positions of Trp (Fig. 2). Subsequent in vitro antibacterial assays of the newly generated **Dap** analogs revealed some displayed potency against **Dap**R strains of *S. aureus* and *Enterococcus faecalis*. Significant increases in potency against **Dap** susceptible (**Dap**S) strains were also observed for several analogs, showing the regiochemistry of alkyl substitution on the Trp residue can modulate **Dap**’s potency. Thus, the study highlighted the plasticity and broad potential of CdpNPT for alkyl-diversification of natural products (NPs) using non-native substrate analogs. Furthermore, the development of novel **Dap** analogs has legitimized PT-based diversification of complex NP scaffolds as a viable method of analog synthesis, and the SAR discussed herein could potentially guide the development of optimized antibacterial leads.

## Materials and methods

### General materials

**Dap** was purchased from TCI (Portland, OR, USA), while all other reagents were purchased from Sigma-Aldrich (St. Louis, MO, USA), Acros (New Jersey, USA), Alfa-Aesar (Ward Hill, MA, USA), or TCI (Portland, OR, USA) and were of reagent grade or better. PD-10 and Ni-NTA superflow columns were purchased from GE Healthcare (Piscataway, NJ). All alkyl-PPs were synthesized as previously reported (Bandari et al. 2019; Bandari et al. 2017; Johnson et al. 2020). The CdpNPT plasmid was generously provided by Prof. Jon S. Thorson (UK, Lexington, KY). All bacterial strains were obtained from the American Type Culture Collection (ATCC) or the Biodefense and Emerging Infections Research Resources Repository (BEI Resources).

### General HPLC and MS methods

Analytical-scale reactions were analyzed, and **Dap** analogs were purified, on an Agilent 1220 HPLC system equipped with a DAD. *Method A*: The CdpNPT-catalyzed reactions were monitored on an analytical reverse-phase (RP) HPLC using a Gemini-NX, C-18 (5 μm, 4.6 mm × 250 mm) column (Phenomenex, Torrance, California, USA) **[**gradient of 10% to 45% B for 10 min, 45% to 100% B for 12 min, 100% to 10% B for 1 min, 10% B for 7 min (A = ddH_2_O with 0.1% TFA; B = acetonitrile); flow rate = 1 mL min^−1^; A_230/265_]. The reactions were monitored by the retention time difference between the starting material and the product. *Method B*: Semi-preparative RP HPLC was conducted on a Gemini-NX, C-18 (5 μm, 10 mm × 250 mm) column (Phenomenex, Torrance, California, USA) to purify the **Dap** analogs [gradient of 10% to 45% B for 5 min, 45% to 65% B for 20 min, 65% to 100% B for 5 min, 100% B for 2 min, 100% to 10% B for 1 min, 10% B for 7 min (A = ddH_2_O with 0.05% formic acid; B = acetonitrile); flow rate = 2 mL min^−1^; A_230/265_]. HRMS and liquid chromatography mass spectrometric (LCMS) data were obtained on an Agilent 6545-QTOF W/1290 HPLC mass spectrometer at the University of Oklahoma Department of Chemistry and Biochemistry.

### NMR spectroscopy

NMR spectra (300–350 μL final volume) were collected at 25 °C in 5 mm Shigemi tubes in 99.9% DMSO-d_6_ with 0.05% v/v TMS (Cambridge Isotope Laboratories, Tewksbury, MA, USA) on a 600 or 800 MHz Varian (Palo Alto, CA) VNMRS spectrometer equipped with a z-axis gradient 5 mm HCN cold probe at the National Magnetic Resonance Facility at Madison (NMRFAM). For each compound, a set of 1D-proton, ^1^H-^1^H-2D-COSY, ^1^H-^1^H-2D-TOCSY (mixing time = 80 ms), sensitivity-enhanced ^1^H-^13^C-2D-HSQC (2048 × 256 complex data points), ^1^H-^13^C-2D-HMBC (1024 × 256 complex data points), and/or HSQC-TOCSY (1024 × 256 complex data points; with a spin lock field of 7.5 kHz and 24 ms) were recorded for resonance assignments. Processing of the spectra was accomplished using the NMRPipe software package (Delaglio et al. 1995). Analysis of NMR data was performed using the NMRFAM-SPARKY software package (Lee et al. 2015).

### CdpNPT assays

CdpNPT was over-expressed and purified as described previously using Ni-NTA chromatography (Elshahawi et al. 2017). Analytical-scale, in vitro CdpNPT reactions were conducted in a volume of 20 μL with 1.2 mM alkyl-PP, 1 mM **Dap**, and 5 μM purified CdpNPT in a reaction buffer (25 mM Tris pH 8.0, 5 mM CaCl_2_, 50 mM KCl). Reactions were incubated at 35 °C for 16 h and were quenched by adding an equal volume of methanol. Quenching was followed by centrifugation (10,000 ×*g* for 15 min) to remove the precipitated protein, and product formation for each reaction was subsequently analyzed by RP-HPLC using method A. For each reaction, percent yield was based upon the integration of species peaks (at 230 nm or 265 nm) and calculated by dividing the integrated area of individual products by the total integrated area of products and remaining substrate. All putative products were subsequently confirmed by HRMS using positive (+) and/or negative (−) modes (see Supporting Information Tables S1 and S2). The **Dap** reactions with > 30% product yield were scaled up using standard conditions (7.5 mM alkyl-PP analog, 5 mM **Dap**, 5 μM CdpNPT, 5 mM CaCl_2_, 50 mM KCl, 25 mM Tris-HCl buffer pH 7.5, total volume 5.0 mL, incubated 16 h at 35 °C) and purified by semi-preparative HPLC using method B. The putative products were confirmed by HRMS (Supporting Information Table S2) and NMR (Supporting Information Table S3).

### Antibacterial activity assays

All **Dap** analog stocks were calibrated at absorbance (*Ɛ*_366 nm_ = 4000 L mol^−1^ cm^−1^) (Debono et al. 1988), and all bioactivity assays were conducted in triplicate. All bacterial strains were obtained from the American Type Culture Collection (ATCC) or the Biodefense and Emerging Infections Research Resources Repository (BEI Resources). The strains for which minimum inhibitory concentrations (MICs) were determined included twelve **Dap**S and three **Dap**R strains. The **Dap**S strains were *S. aureus* (ATCC 25923); *S. aureus*, MRSA (ATCC 700787); *Staphylococcus epidermidis* (ATCC 12228); *Enterococcus faecalis*, VRE (ATCC 700802); *Bacillus subtilis* (ATCC 6051); *S. aureus*, VISA, strain SR220 (BEI NR-50512); *S. aureus*, strain SR1129 (BEI NR-50506); *S. aureus*, strain SR2609 (BEI NR-50507); *S. aureus*, strain SR2852 (BEI NR-50508); *S. aureus*, strain SR3777 (BEI NR-50509); *S. aureus*, strain SR4035 (BEI NR-50510); and *Enterococcus faecalis*, strain S613 (BEI HM-334). The **Dap**R strains were *S. aureus*, strain JE2, transposon mutant NE573 (BEI NR-47116); *S. aureus*, strain JE2, transposon mutant NE1656 (BEI NR-48198); and *Enterococcus faecalis*, strain R712 (BEI HM-335). MIC testing against all strains was performed in Mueller-Hinton Broth (MHB) medium supplemented with 50 mg L^−1^ of calcium (MHBc) using NCCLS guidelines for broth microdilution methods and inoculum of 1 × 10^5^–5 × 10^5^ CFU/mL (CLSI: *Performance Standards for Antimicrobial Susceptibility Testing*. 29th ed.). The serial two-fold dilutions of compounds ranged from 5 to 0.039 μM and 1.6 to 0.0125 μM. Briefly, the overnight cultures were grown at 35 °C in MHBc, while **Dap** and **Dap** analogs were serially diluted in MHBc. The serially diluted media was then inoculated with 10,000 counts of bacterial cells from an overnight culture, after which the culture plates were incubated with shaking at 35 °C for 20–24 h. Growth was evaluated using the absorbance at 600 nm. The lowest concentration causing 90% inhibition microbial growth was defined as the MIC, and the concentration units were converted to μg mL^−1^ in Table 1 for consistency with literature values. Growth and sterility controls were included in each experiment, and two representative Gram-negative bacteria, *Escherichia coli* (ATCC 11775) and *Pseudomonas aeruginosa* (ATCC 10145), were included as additional negative controls. The cancer cell-line cytotoxicity assays were used as a measure of general eukaryotic cell toxicity and employed the human hepatocyte carcinoma cell line HepG2 (ATCC HB-8065). The cytotoxicity of **Dap** and its analogs were tested against the HepG2 cell line at 20 μM by using the MTT assay (You et al. 2010).

**Table 1:**
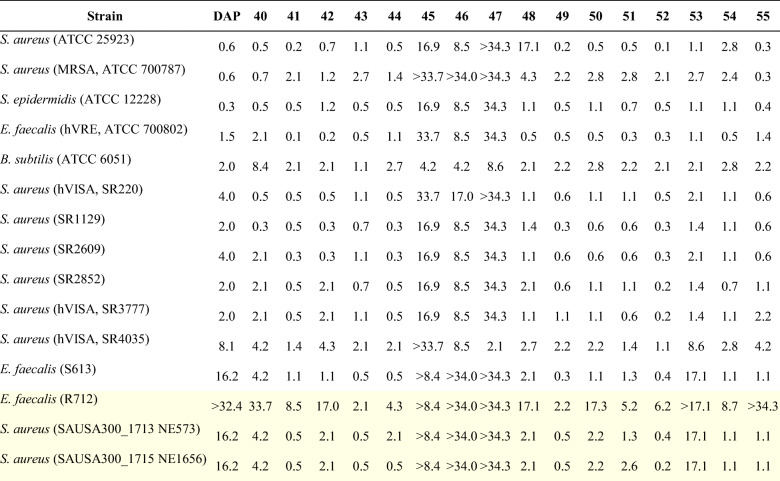
MIC (in μg mL^−1^) of alkyl-Dap analogs against a panel of twelve DapS and three DapR strains (highlighted with yellow background) obtained using a standard microdilution assay as MIC_90_ after incubation at 35 °C for 22 h

## Results

### Library of non-native alkyl-PPs

**Fig. 3:**
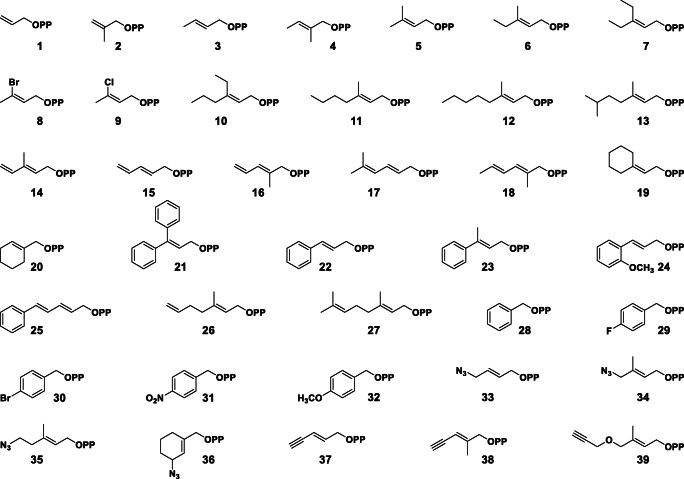
The alkyl-PP library utilized in this study

The assessment of CdpNPT-catalyzed alkylation of **Dap** was carried out using a library of 38 non-native alkyl-PPs synthesized in-house as described previously (**1**–**4**, **6**–**39**, Fig. 3) (Bandari et al. 2019; Bandari et al. 2017; Johnson et al. 2020). The assay also included the previously reported natural analog **5** for comparison purposes (Elshahawi et al. 2017). Each alkyl-PP contained a double bond at the β-position (Liebhold and Li 2013; Liebhold et al. 2012), but beyond this commonality, the synthetic donors represented a wide chemical space. Most of the resulting library consisted of allylic moieties (**1**–**27**, **33**–**39**) containing variations in alkyl-chain length, electronic character, and the presence or positioning of branching methyl groups. A small number of analogs were benzylic in nature (**28**–**32**) and featured differences in the strength of electron-donating and electron-withdrawing substituents. Additionally, a subset of alkyl-PPs contained moieties amenable to downstream chemoselective reactions (terminal alkenes: **1**, **2**, **14**–**16**, **26**; azides: **33**–**36**; alkynes: **37**–**39**). Thus, the overall library covers a vast chemical space of alkyl-donors not previously explored for CdpNPT.

### Substrate scope of CdpNPT-catalyzed reactions with **Dap**

**Fig. 4:**
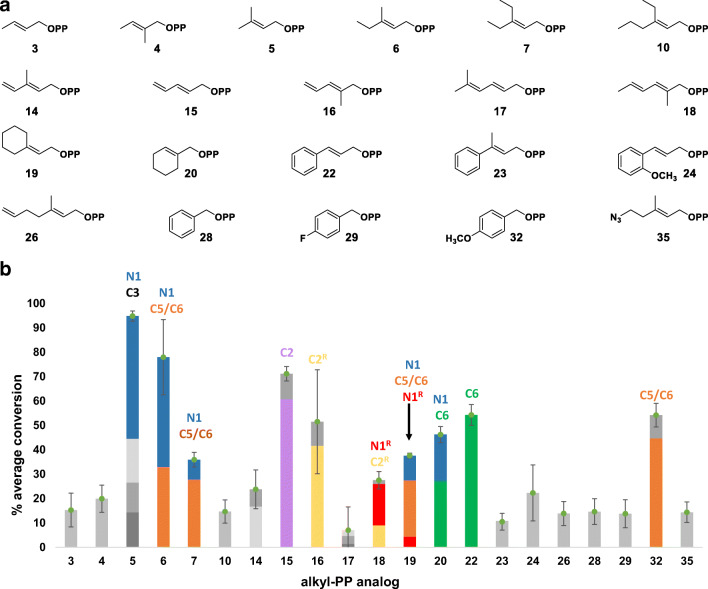
Donor profile of analytical-scale CdpNPT-catalyzed reactions with Dap. **a** Structures of analogs whose corresponding reactions gave turnover. **b** Average percent conversion of all positive reactions with associated standard deviation (*n* = 3) as determined by RP-HPLC (see Supporting Information). Production of individual peaks within each reaction is shown using different colored sections of the full bar. NMR-confirmed structures were given specific colors: blue, N1; red, N1^R^ (reverse-alkylated); purple, C2; yellow, C2^R^ (reverse-alkylated); orange, C5/C6 mix; green, C6. Each reaction was carried out in a 20-μL volume and contained 1.2 mM alkyl-PP, 1 mM Dap, and 5 μM purified CdpNPT in a reaction buffer (25 mM Tris, pH 8.0, 5 mM CaCl_2_, 50 mM KCl) incubated at 35 °C for 16 h. No product formation was observed in the absence of CdpNPT or alkyl-PP

Initial assessment of the CdpNPT-catalyzed alkylation of **Dap** was illustrated in Fig. 4. Products were detected for 21 of the 39 alkyl-PP analogs (**3**–**7**, **10**, **14**–**20**, **22**–**24**, **26**, **28**, **29**, **32**, **35**) and were subsequently confirmed by HRMS analysis (see Supporting Information Table S1). Most of these **Dap** analogs were mono-alkylated, but in two instances involving **5** and **15**, a small amount of di-alkylated **Dap** was also detected and confirmed by HRMS (Supporting Information Table S1). Of the 21 positive reactions, six (**5**, **6**, **15**, **16**, **22**, and **32**) led to appreciable (> 50%) production of alkyl-**Dap** derivatives, six (**7**, **14**, **18**–**20**, **24**) resulted in moderate (20–50%) conversion, and the remaining nine (**3**, **4**, **10**, **17**, **23**, **26**, **28**, **29**, **35**) offered detectable product formation (< 20%) under the standard conditions (Fig. 4). Furthermore, the CdpNPT-catalyzed reactions showed a preference for allylic alkyl-PPs, as 17 (**3**–**7**, **10**, **14**–**20**, **22**–**24**, **26**) of the 21 positive reactions fit this description. The remaining four hits included three benzylic groups (**28**, **29**, **32**) and a single reactive handle (the azide **35**). Additionally, 10 of the positive allylic reactions (**5**–**7**, **14**–**20**) and the *p*-methoxybenzyl analog (**32**) resulted in multiple alkylated **Dap** products, which were indicated by the presence of multiple peaks in the HPLC chromatograms (see Supporting Information Fig. S2). Thus, as with other PTs, CdpNPT shows impressive promiscuity toward non-native donors even in the presence of an unnatural acceptor (Bandari et al. 2019; Bandari et al. 2017; Johnson et al. 2020; Liebhold and Li 2013; Liebhold et al. 2012; Liebhold et al. 2013; Winkelblech et al. 2015; Yu et al. 2015).

### Structural characterization of selected alkyl-**Dap** analogs

**Fig. 5:**
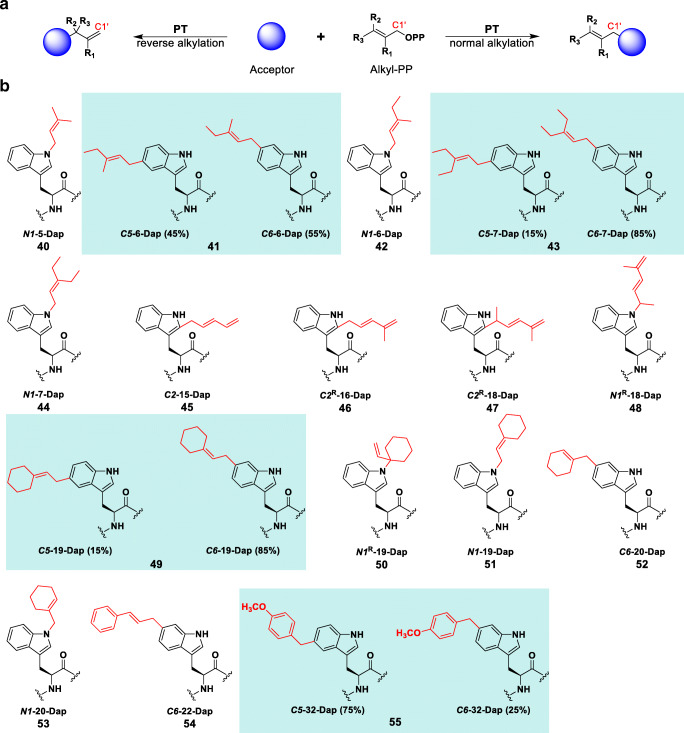
**a** Generalized scheme showing PT-catalyzed normal and reverse alkylations of an alkyl-acceptor. **b** NMR-characterized structures of alkyl-Dap analogs. Blue-boxed structures represent inseparable mixtures of C5-/C6-regio-isomers (see Supporting Information). Small Rs indicate reverse-alkylated products

The analytical scale reactions affording ≥ 30% product yield (Fig. 4, alkyl-PPs **5**–**7**, **15**, **16**, **18**–**20**, **22**, and **32**) were scaled up and purified for subsequent characterization using HRMS and NMR spectroscopy. The reaction of **5** produced *N1*- and *C3*-regioisomers of **Dap** in the previous study, with the *N1*-prenylated major product (**40**) exhibiting increased antibacterial activity compared with **Dap** (Elshahawi et al. 2017). Thus, we isolated **40** in the current study and included it as a positive control for comparison purposes. As for the non-native alkyl-PPs, NMR analyses of the alkylated **Dap** products revealed that CdpNPT catalyzes *N1*-, *C2*-, *C5*- or *C6*-alkylation of **Dap** using these donors (Fig. 5). Specifically, *N1*-alkylation was observed with alkyl-PPs **6**, **7**, and **18**–**20**; *C2*-alkylation with **15**, **16**, and **18**; *C5*-alkylation with **6**, **7**, **19**, and **32**; and *C6*-alkylation with **6**, **7**, **19**, **20**, **22**, and **32**. Thus, *C*2-regioisomers were only produced as a major product with diene-containing alkyl-PPs. Furthermore, four of the alkyl-**Dap** analogs (**46**–**48**, **50**) exhibited “reverse” alkylation, a phenomenon observed in the natural CdpNPT reaction in which the new C-C or C-N bond forms using a carbon atom of the alkyl-PP other than C1′ (Fig. 5a) (Bandari et al. 2019; Schuller et al. 2012). The majority of these analogs (**46**–**48**) arose from diene-containing alkyl-PPs, though **50** involved a cyclic derivative of **5** (**19**). Considered together, these two patterns seem to indicate the existence of active-site interactions prevalent to the conjugated, non-aromatic carbocations of the diene-containing alkyl-PPs. Additionally, while NMR confirmed the purity of most analogs to be > 90% single regio-isomers, four of the sixteen alkyl-**Dap** analogs (**41**, **43**, **49**, and **55**) were found to contain inseparable mixtures of *C5*-/*C6*-regioisomers (blue background in Fig. 5b, Supporting Information Table S3). After several attempts to separate these combinations of regioisomers failed, it was decided that any further evaluation of their structures or antibacterial activities would be assessed as mixtures. Notable among these mixtures was the generally lower concentration of *C5*-regioisomers compared with their *C6*-counterparts, except in the case of **55**. Coupled with the lack of free *C5*-regioisomers, this pattern could indicate C5 as a less accessible position on the Trp moiety than either C6 or N1 and more easily reached by benzylic analogs, such as **32**. Thus, while limited by the characterization of only a handful of products, the trends observed here offer initial considerations for future engineering work in pursuit of regio-specifically modified alkyl-**Dap** analogs. Additionally, the compounds isolated here provide a novel example of a chemoenzymatic scheme capable of alkylating the relatively inert benzene carbons of the indole moiety on a complex NP.

### Antibacterial activity of purified alkyl-**Dap** analogs

Once fully characterized, the entire set of alkyl-**Dap** analogs was tested for antibacterial activity against a panel of fifteen Gram-positive bacteria, including both **Dap**S and **Dap**R strains, and two Gram-negative controls (Table 1, Supporting Information Fig. S1). The assay included the previously reported analog **40** for comparison purposes (Elshahawi et al. 2017) and the parent **Dap** as a third control. Additionally, the analogs were tested for general toxicity against eukaryotic cells, which remained largely unchanged compared with **Dap**, using human hepatocyte carcinoma cell line HepG2. As expected, none of the compounds exhibited antimicrobial activity against Gram-negative *P. aeruginosa* and *E. coli*. However, several alkyl-**Dap** analogs possessed significant activity against most of the Gram-positive strains (Table 1, Supporting Information Fig. S1), most notably the *C6*-, *C5*-, and *N1*-substituted analogs. In general, these regioisomers displayed improved activity against **Dap**S *S. aureus* compared with the parent compound, with **52** showing the greatest improvement for most strains. In terms of the other **Dap**S strains, significant changes in MIC were not observed for either *S. epidermidis* or *B. subtilis*, while analogs **41**, **42**, **51**, and **52** displayed a 5–15-fold enhanced activity toward vancomycin-resistant *Enterococcus* (Table 1, Fig. S1).

Even more interesting, however, were the activities of the *C6*-, *C5*-, and *N1*-substituted analogs with **Dap**R strains. The analogs **41**, **43**, **49**, and **52** showed a MIC range of 0.2–0.5 μg mL^−1^ (Table 1) and a 30–80-fold improvement in antibacterial activity over **Dap** with **Dap**R *S. aureus* (Supporting Information Fig. S1). Meanwhile, all except **53** and the *C2*-alkyl substituted analogs (**45**–**47**) had MIC ≤ 4 μg mL^−1^ with *Enterococcus faecalis* strain S613 (**Dap** MIC of ~16 μg mL^−1^), with the four best analogs (**43**, **44**, **49**, and **52**) possessing a MIC range of 0.3–0.6 μg mL^−1^. Analogs **43** and **49** even displayed MICs of ~ 2 μg mL^−1^ against the highly **Dap**R *Enterococcus faecalis* strain R712 (Table 1). Thus, in general, the most potent alkyl-**Dap** analogs against **Dap**S and **Dap**R strains were either a single *C6*-alkyl-substituted **Dap** (**52**) or a mixture of *C5*- and *C6*- regioisomers (**41**, **43**, and **49**). Such large increases in activity among the analogs, especially considering the similar toxicity profile to the parent compound, point to the potential of the alkyl-**Dap** compounds developed here to become new drug leads. In contrast to these potent **Dap** analogs, a sharp decrease in overall activity was observed for *C2*-substituted-Trp analogs (**45**–**47**) when compared with **Dap**. A single MIC decrease was observed for one of these analogs among the **Dap**S strains (**47** with *S. aureus* SR4035, Table 1), with all other MICs being 2–100-fold higher than the parent **Dap**. Experiments with the **Dap**R strains fared little better, as most of the MIC values for the *C2*-regioisomers were comparable to or higher than the values for **Dap**. Taken together, these trends build on previous work (Elshahawi et al. 2017; He et al. 2012) to more firmly establish the effect of Trp-modification on the activity of **Dap**. Furthermore, several alkyl-**Dap** analogs produced here have the potential to transition to “drug lead” status based on their potency against both **Dap**S and **Dap**R bacterial strains.

## Discussion

A deeper analysis of the turnover ratios observed in the analytical scale reactions revealed interesting patterns to consider in future studies of CdpNPT. DMAPP (**5**) provided the highest overall turnover (94.8 ± 2.1%) with **Dap**, which is unsurprising considering it is the natural donor for CdpNPT. However, the presence of four peaks in the chromatogram implied that the induced fit of donor and acceptor in the active site is not optimal, resulting in low regiospecificity. The structure of CdpNPT has been solved previously (Schuller et al. 2012), so additional crystallographic studies with **Dap** and **5** seem like a plausible next step in the pursuit of optimized enzymes. As for the other analogs, the most successful allylic reactions included analogs very similar to **5** in the length of their alkyl-chain (4–6 unbranched carbons: **6**, **7**, **14**–**16**, **18–20**), while several analogs with shorter (< 4 carbons: **1** and **2**) or longer (> 6 carbons: **11**–**13**, **23**–**27**) chains showed 0–20% turnover. The binding pocket of CdpNPT has been optimized by nature to utilize DMAPP with indole moieties, so it is highly probable that donors of similar overall structure would also be transferred with relatively similar efficiency.

Unbranched chain length, however, did not seem to be the only steric factor at play. Notably, the conjugated analog series of **21**–**24** experienced significant losses in activity as larger substituents were added to the core scaffold of **22**. For example, the introduction of an *o*-methoxy group (**24**) substantially lowered the overall turnover. Perhaps even more astonishing was the addition of a methyl group on C3′ of **23**, which lowered the turnover even further despite the analog’s closeness in structure to **5**. The bulky phenyl group attached to C3′ of **21** destroyed activity altogether, further highlighting the impact of steric hindrance in the active site. Additionally, carbocation stabilization likely played a role in substrate acceptance. For example, the removal (**1**, **2**, **3**), substitution (**8**, **9**), or movement (**2**, **4**) of the branching methyl groups from C3′ of **5** may explain the lower turnover of the corresponding analogs, as these modifications would likely cause a loss of hyperconjugation. Removal/migration of this methyl group from C3′ also removed the possibility of forming tertiary carbocations by resonance, further reducing stability. Thus, while chain length seems to be an acceptable general criterion for predicting allylic donor acceptance, more subtle steric and electronic factors play significant roles as well.

Similar patterns were identified within the other classes of donors. For the benzylated analogs, the substrate scope appeared to arise from an intricate balance between electron donation/withdrawal and steric bulk. The core benzylic scaffold (**28**) was taken marginally (14.6 ± 5.3%) and the turnover was unaffected by inductive electron withdrawal (*p*-fluorinated **29**, 13.8 ± 5.7%) except for the much bulkier *p*-bromo analog (**30**). In contrast, the strong electron withdrawing effect of nitro substituents (**31**) destroyed activity completely, while strong electron donation through delocalization (**32**) increased turnover nearly 4-fold. These patterns indicated a strong dependence of the alkylation reaction on carbocation stability in addition to available space in the active site. As for the analogs with chemoselective functionality, there appeared to be a clear distinction between the types of patterns observed for each subclass. For example, the dienes and the terminal alkene were relatively straightforward. Five of the six dienes were utilized to varying extents, with individual turnovers connected to the presence and placement of methyl groups on the core scaffold of **15**. The bulky phenyl group of **25**, however, appeared to exclude this analog from activity altogether. The terminal alkene **26** was utilized with relatively low turnover (13.8 ± 4.9%), most likely due to its carbon chain length as described earlier.

**Fig. 6:**
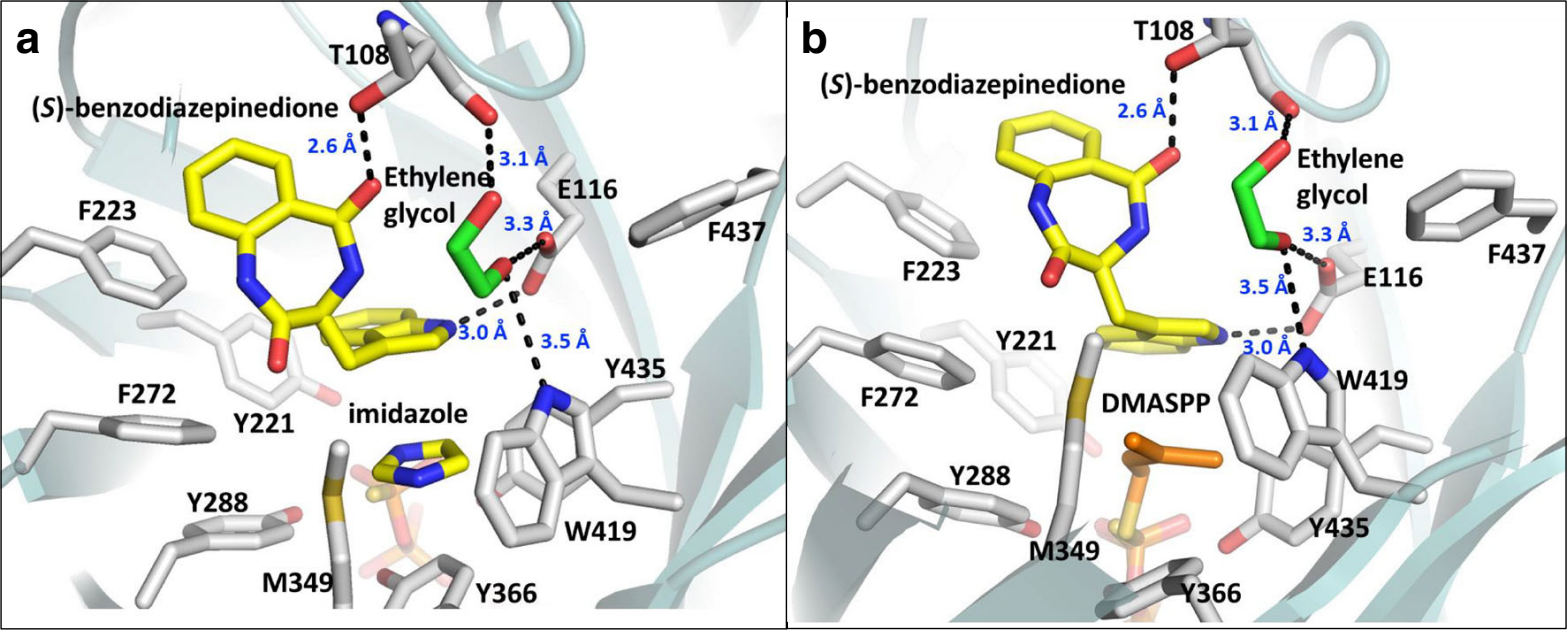
The crystal structure of CdpNPT (PDB ID: 4E0U) containing either the originally bound imidazole molecule (**a**) or a manually docked DMASPP molecule (**b**). Polar interactions are shown as black dashes, and their corresponding lengths (in Å) are given in blue

The azides, however, presented much more interesting patterns. Only one of the four (**35**, the longest chain of unbranched carbons) was utilized to a small extent (14.3 ± 4.3%). Analog **36** was likely hindered by its relatively higher three-dimensionality compared with the other azides, but the activity pattern of **33**–**35** was especially surprising considering the more flexible and hydrophobic **11** and **12** gave no turnover at all. Using the published ternary complex of CdpNPT (PDB ID: 4E0U) (Schuller et al. 2012), we manually docked DMASPP into the active site to understand the potential interactions that could explain this turnover (Fig. 6). As shown in Fig. 6, there are a considerable number of polar contacts observed within the binding site, several of which are derived from the hydroxyl groups of an ethylene glycol (EG) likely obtained from the crystallization mixture. The contacts included side-chain interactions with Glu116 and Trp419, as well as an interaction with the main-chain carboxyl group of Thr108. Based on the presence of these contacts in the ternary complex, we speculate that the turnover observed for azide **35** could be derived from a stabilizing interaction with one or more of the polar groups in place of EG. The need for a polar interaction would explain the inability of **11** and **12** to turn over, as their hydrophobic tails would be excluded from the EG binding site, while the separation of the EG and DMASPP binding sites could explain why the shorter azides (**33**, **34**) gave no turnover. Their alkyl-tails may not have been long enough to reach the EG binding site, resulting in their azide groups being unable to form the needed polar contact for stabilization.

As for the alkynes, none of the three (**37**–**39**) gave detectable turnover, which likely arose from unfavorable steric interactions in the active site. For example, while **37** and **38** were not utilized, their alkene analogs (**15** and **16**, respectively) were accepted relatively well by CdpNPT. The linear alkyne groups of **37** and **38** likely abolished the ability of these analogs to fit easily into the binding site and enter active conformations. The dienes **15** and **16**, while still having few rotatable bonds, are slightly more flexible than their alkyne counterparts, likely allowing them to enter the active site more easily. As for **39**, it most likely suffered from similar inflexibility due to the terminal alkyne, though its inactivity could also be attributed to exclusion from the EG binding site. In terms of chain length, alkyne **39** has a similar number of unbranched atoms to azide **35**, which would imply that it could fit spatially in a similar conformation. However, the presence of the hydrophobic terminal alkyne would disfavor any proximity toward the polar atoms known to interact with EG. Thus, even among analogs with much less variation, activity appeared to stem from the complex interaction of three-dimensional structure and electronic stabilization. Such factors will be essential to consider in future alkyl-diversification efforts with both CdpNPT and the PT class as a whole.

Within this same vein, the alteration of a single **Dap** residue seemed to introduce new structural interactions that significantly affected its activity. The *C6*-, *C5*-, and *N1*-substituted **Dap** analogs produced in the current study exhibited modest to impressive increases in antibacterial activity compared with the parent compound. Meanwhile, the *C2*-substituted analogs displayed significantly worse activity than both the other regioisomers as well as unsubstituted **Dap**. These differences in activity could be due to differences between the structures of **Dap** and its analogs either in complex with Gram-positive membranes or in their unbound form. However, with no three-dimensional structure data for the analogs, only conjecture can be offered as to the cause. Based on the available 3D-NMR data for the parent **Dap** (Ball et al. 2004; Jung et al. 2004), the benzene ring of Trp is thought to stick out away from the peptide core and lie roughly parallel to the main axis of the acyl tail. Assuming this orientation is maintained upon alkylation of C5 or C6, the additional hydrophobicity of the added alkyl group could increase the frequency and strength of **Dap** insertion into the cell membrane, thus increasing the analog’s activity. In the case of *N1*-substitution, the hydrophobic alkyl group may not extend to the same length along the acyl chain as in *C5*-/*C6*-substitution, causing a decrease in activity compared with the *C5*/*C6*-regioisomers. As for the *C2*-substituted analogs, new steric strain introduced at the normally inward-facing position could cause a rotation of the indole ring and subsequent change in peptide fold that interferes with oligomerization. A similar argument could be made for the *C3*-reverse-prenylated **Dap** compound produced previously (Elshahawi et al. 2017), which also suffered from a decrease in activity compared with its *N1*-normal-prenylated regioisomer. Conversely, the diene moieties attached at the C2 position are prone to conjugate addition reactions which would prevent the proper binding conformation or even enable degradation pathways. Therefore, to check their stability, all purified **Dap** analogs were reinjected on RP-HPLC after their structural and activity characterization (see Supporting Information Fig. S3). Notably, the chromatogram of **45** seemed to indicate significant degradation of the final product (see Supporting Information Fig. S3). While future kinetic and engineering studies will likely answer these questions, the SAR identified in this study will inform future **Dap** development efforts using the CdpNPT-based diversification scheme. The *C6*-, *C5*-, and *N1*-substituted **Dap** analogs developed here even have the potential to progress further in the drug discovery pipeline as lead compounds.

In summary, the current study has provided a proof-of-concept example of indole functionalization on complex NPs using a PT. Specifically, the late-stage diversification of **Dap** has been achieved using CdpNPT in conjunction with a library of 39 native and non-native alkyl-PPs, 21 of which resulted in successful production of alkylated product. These analytical-scale reactions outlined the previously unreported donor scope of CdpNPT with **Dap** as the acceptor, which allowed for preliminary analysis of how steric factors and carbocation stability affected activity. Further scale-up experiments produced 16 alkyl-**Dap** analogs for structural elucidation by NMR and eventual antibacterial activity assays. The resulting spectra indicated that CdpNPT was capable of alkylating multiple positions on the Trp moiety of **Dap** (including N1, C2, C5, and C6), and the subsequent antibacterial assays produced the first SAR studies of Trp-alkylation of **Dap**. The results of these experiments revealed the dramatic effect of differential alkylation on an analog’s ability to kill Gram-positive strains. In general, *N1*-, *C5*-, and *C6*-substituted analogs showed higher potency against both **Dap**S and **Dap**R strains compared with the parent molecule, while the *C2*-substituted analogs were much less effective, all possibly due to changes in how the analog interacts with the membrane compared with **Dap**. Thus, the current study has delivered several **Dap** analogs effective against **Dap**R bacteria, further strengthening the utility of CdpNPT as a legitimate drug discovery tool. While a lack of regioselectivity currently limits the approach, the advent of protein engineering is likely to improve this aspect of the described methodology. Finally, once the reaction has been optimized with enzyme variants, more in-depth SAR studies and in vivo infection models become much more accessible for the potential drug leads identified here.

## Electronic supplementary material

ESM 1 (PDF 8680 kb)

## Data Availability

All data are available upon reasonable request.
