# Supplementary material for: Chemoenzymatic synthesis of daptomycin analogs active against daptomycin-resistant strains
Source: Appl Microbiol Biotechnol. 2020 Jul 28;104(18):7853–65. doi: 10.1007/s00253-020-10790-x (PMC7447621; doi:10.1007/s00253-020-10790-x)
Supplement: Supplementary file 1 — (PDF 8680 kb) [file 253_2020_10790_MOESM1_ESM.pdf]

Applied Microbiology and Biotechnology

Supporting Information

Chemoenzymatic Synthesis of Daptomycin Analogs Active Against Daptomycin-Resistant Strains.

Erin M. Scull[‡,†], Chandrasekhar Bandari[‡,†], Bryce P. Johnson[†], Eric D. Gardner[†], Marco Tonelli[§], Jianlan You[†], Robert H. Cichewicz[†], and Shanteri Singh[*,†]

[†]Department of Chemistry and Biochemistry, University of Oklahoma, Stephenson Life Sciences Research Center, 101 Stephenson Parkway, Norman, Oklahoma 73019, United States. [§]University of Wisconsin-Madison, National Magnetic Resonance Facility at Madison, 411 Babcock Drive, Madison, Wisconsin 45005, United States.

[‡]These authors contributed equally.
[*]To whom correspondence should be addressed. *E-mail:* shanteri.singh@ou.edu

Table of Contents

1. **Table S1**: HRMS data of alkyl-**Dap** products of analytical-scale reactions S2
2. **Table S2**: HRMS data of pure alkyl-**Dap** analogs S3
3. **Figure S1:** The relative potency of alkyl-**Dap** analogs S4
4. **Figure S2**: HPLC plots of CdpNPT catalyzed analytical scale reactions S5
5. **Figure S3**: HPLC plots of pure alkyl-**Dap** analogs S6
6. **Table S3**: NMR assignment of alkyl-**Dap** analogs S7-S12
7. HRMS of **Dap** S13
8. HRMS of CdpNPT-catalyzed analytical-scale reactions of alkyl-PPs with **Dap** S14-S41
9. HRMS of purified alkyl-**Dap** analogs (**40-55**) S42-S57
10. NMR spectra of **Dap** S58-S62
11. NMR spectra of purified alkyl-**Dap** analogs (**40-55**) S63-S152

Table S1: Summary of HRMS data for alkylated **Dap** products from CdpNPT-catalyzed analytical-scale reactions of **Dap** with alkyl-PPs.

Enzyme Product*	Chemical Formula $[M+H]^+$/ $[M+2H]^{2+}$	Calculated Mass (m/z)	Observed Mass (m/z)	Retention Time (mins)
**Dap**	$[C_{72}H_{102}N_{17}O_{26}]^+$	1620.7182	1620.7181	4.032
**3-Dap**	$[C_{76}H_{108}N_{17}O_{26}]^+$	1674.7651	1674.7670	4.499
**4-Dap**	$[C_{77}H_{110}N_{17}O_{26}]^+$	1688.7807	1688.7753	4.610
**5-Dap**	$[C_{77}H_{110}N_{17}O_{26}]^+$	1688.7807	1688.7813[a]	4.424
	$[C_{77}H_{110}N_{17}O_{26}]^+$	1688.7807	1688.7824[b]	4.490
	$[C_{82}H_{118}N_{17}O_{26}]^+$	1756.8433	1756.8416[c#]	4.722
**6-Dap**	$[C_{78}H_{112}N_{17}O_{26}]^+$	1702.7964	1702.7978[a]	4.641
	$[C_{78}H_{112}N_{17}O_{26}]^+$	1702.7964	1702.7973[b]	4.790
**7-Dap**	$[C_{79}H_{114}N_{17}O_{26}]^+$	1716.8120	1716.8121	4.852
**10-Dap**	$[C_{80}H_{116}N_{17}O_{26}]^+$	1730.8277	1730.8261	4.780
**14-Dap**	$[C_{78}H_{110}N_{17}O_{26}]^+$	1700.7807	1700.7802	4.508
**15-Dap**	$[C_{77}H_{108}N_{17}O_{26}]^+$	1686.7651	1686.7638[a]	4.564
	$[C_{82}H_{114}N_{17}O_{26}]^+$	1752.8120	1752.8094[b#]	4.713
**16-Dap**	$[C_{78}H_{110}N_{17}O_{26}]^+$	1700.7807	1700.7830[a]	4.431
	$[C_{78}H_{110}N_{17}O_{26}]^+$	1700.7807	1700.7823[b]	4.762
**17-Dap**	$[C_{79}H_{113}N_{17}O_{26}]^{2+}$	857.9021	857.9004	4.688
**18-Dap**	$[C_{79}H_{112}N_{17}O_{26}]^+$	1714.7964	1714.7982[a]	4.578
	$[C_{79}H_{112}N_{17}O_{26}]^+$	1714.7964	1714.7984[b]	4.752
**19-Dap**	$[C_{80}H_{114}N_{17}O_{26}]^+$	1728.8120	1728.8111	4.835
**20-Dap**	$[C_{79}H_{112}N_{17}O_{26}]^+$	1714.7964	1714.7986	4.794
**22-Dap**	$[C_{81}H_{110}N_{17}O_{26}]^+$	1736.7807	1736.7806	4.706
**23-Dap**	$[C_{82}H_{112}N_{17}O_{26}]^+$	1750.7964	1750.7978	4.734
**24-Dap**	$[C_{82}H_{112}N_{17}O_{27}]^+$	1766.7913	1766.7914	4.366
**26-Dap**	$[C_{80}H_{114}N_{17}O_{26}]^+$	1728.8120	1728.8067	4.756
**28-Dap**	$[C_{79}H_{108}N_{17}O_{26}]^+$	1710.7650	1710.7569	4.536
**29-Dap**	$[C_{79}H_{107}FN_{17}O_{26}]^+$	1728.7557	1728.7498	4.517
**32-Dap**	$[C_{80}H_{110}N_{17}O_{27}]^+$	1740.7756	1740.7767[a]	4.410
	$[C_{80}H_{110}N_{17}O_{27}]^+$	1740.7756	1740.7749[b]	4.617
**35-Dap**	$[C_{78}H_{111}N_{20}O_{26}]^+$	1743.7978	1743.7892	4.621

***The number preceding the hyphen denotes the alkyl-PP analog used in the reaction. [#]di-alkylated **Dap**

Table S2: Summary of HRMS data and isolated yields of purified alkyl-**Dap** analogs.

Compound	Chemical Formula $[M+H]^+$/ $[M-H]^-$	Calculated Mass (m/z)	Observed Mass (m/z)	Isolated Yield (%)
**Dap**	$[C_{72}H_{102}N_{17}O_{26}]^+$	1620.7182	1620.7181	
**40**	$[C_{77}H_{108}N_{17}O_{26}]^-$	1686.7651	1686.7659	23%
**41**	$[C_{78}H_{110}N_{17}O_{26}]^-$	1700.7808	1700.7816	22%
**42**	$[C_{78}H_{110}N_{17}O_{26}]^-$	1700.7808	1700.7818	26%
**43**	$[C_{79}H_{112}N_{17}O_{26}]^-$	1714.7964	1714.7979	21%
**44**	$[C_{79}H_{112}N_{17}O_{26}]^-$	1714.7964	1714.7978	9%
**45**	$[C_{77}H_{108}N_{17}O_{26}]^+$	1686.7651	1686.7638	9%
**46**	$[C_{78}H_{108}N_{17}O_{26}]^-$	1698.7651	1698.7605	18%
**47**	$[C_{79}H_{110}N_{17}O_{26}]^-$	1712.7808	1712.7800	5%
**48**	$[C_{79}H_{110}N_{17}O_{26}]^-$	1712.7808	1712.7814	11%
**49**	$[C_{80}H_{114}N_{17}O_{26}]^+$	1728.8121	1728.8111	8%
**50**	$[C_{80}H_{112}N_{17}O_{26}]^-$	1726.7964	1726.7957	3%
**51**	$[C_{80}H_{112}N_{17}O_{26}]^-$	1726.7964	1726.7956	2%
**52**	$[C_{79}H_{112}N_{17}O_{26}]^+$	1714.7964	1714.7995	6%
**53**	$[C_{79}H_{112}N_{17}O_{26}]^+$	1714.7964	1714.7970	21%
**54**	$[C_{81}H_{108}N_{17}O_{26}]^-$	1734.7651	1734.7659	27%
**55**	$[C_{80}H_{108}N_{17}O_{27}]^-$	1738.7601	1738.7578	28%

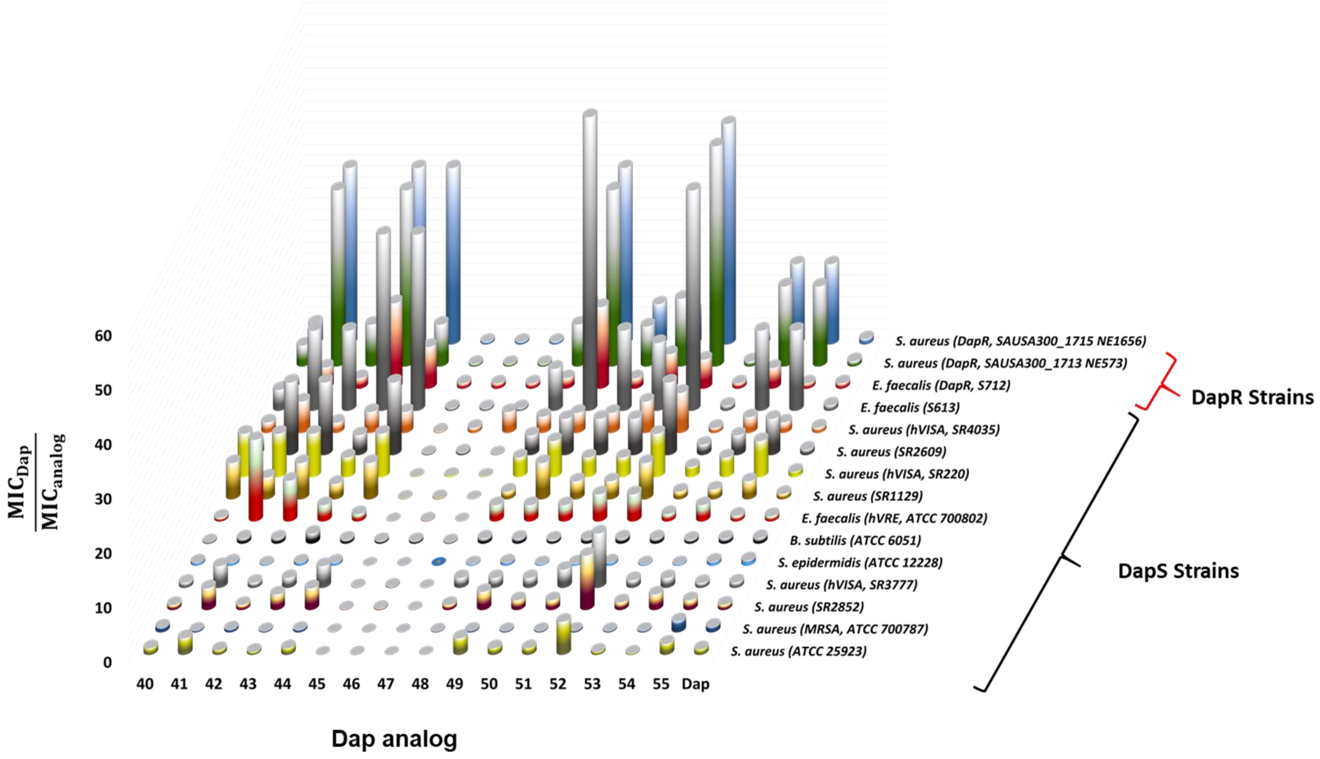

Figure S1. The relative potency of **Dap** analogs against a panel of twelve **Dap**S and three **Dap**R strains (listed in the table below) obtained using a standard microdilution assay as MIC_{90} after incubation at 35 °C for 22 h. None of the **Dap** analogs exhibited antimicrobial activity at 34 µg mL^{-1} against two Gram negative controls, *Escherichia coli* and *Pseudomonas aeruginosa*.

**List of bacterial strains used in this study**			
**Organism**	**Public Repository Strain ID**	**Type Code in Paper**	**Public Source/Repository**
Escherichia coli	11775	Gram negative	ATCC
Pseudomonas aeruginosa	10145	Gram negative	ATCC
Staphylococcus aureus	25923	DapS	ATCC
Staphylococcus aureus	700787	DapS	ATCC
Staphylococcus epidermidis	12228	DapS	ATCC
Enterococcus. faecalis	700802	VRE, DapS	ATCC
Bacillus subtilis	6051	DapS	ATCC
Staphylococcus aureus, Strain SR220	NR-50512	VISA, DapS	BEI Resources
Staphylococcus aureus, Strain SR1129	NR-50506	DapS	BEI Resources
Staphylococcus aureus, Strain SR2609	NR-50507	DapS	BEI Resources
Staphylococcus aureus, Strain SR2852	NR-50508	DapS	BEI Resources
Staphylococcus aureus, Strain SR3777	NR-50509	DapS	BEI Resources
Staphylococcus aureus, Strain SR4035	NR-50510	DapS	BEI Resources
Enterococcus faecalis S613	HM-334	DapS	BEI Resources
Enterococcus faecalis R712	HM-335	DapR	BEI Resources
Staphylococcus aureus, Strain JE2, Transposon Mutant NE573	NR-47116	DapR	BEI Resources
Staphylococcus aureus, Strain JE2, Transposon Mutant NE1656	NR-48198	DapR	BEI Resources

Figure S2: RP-HPLC plots of for alkyl-Dap formation in CdpNPT-catalyzed analytical scale reactions. Chromatograms represent alkyl-**Dap** formation for the corresponding alkyl-PP labelled on the right of each chromatogram. Each reaction was carried out in a 20-μL volume and contained 1.2 mM alkyl-PP analog, 1 mM **Dap**, and 5 μM purified CdpNPT in a reaction buffer (25 mM Tris pH 8.0, 5 mM $CaCl_2$, 50 mM KCl) incubated at 35 °C for 16 h. No product formation was observed in the absence of CdpNPT or alkyl-PP.

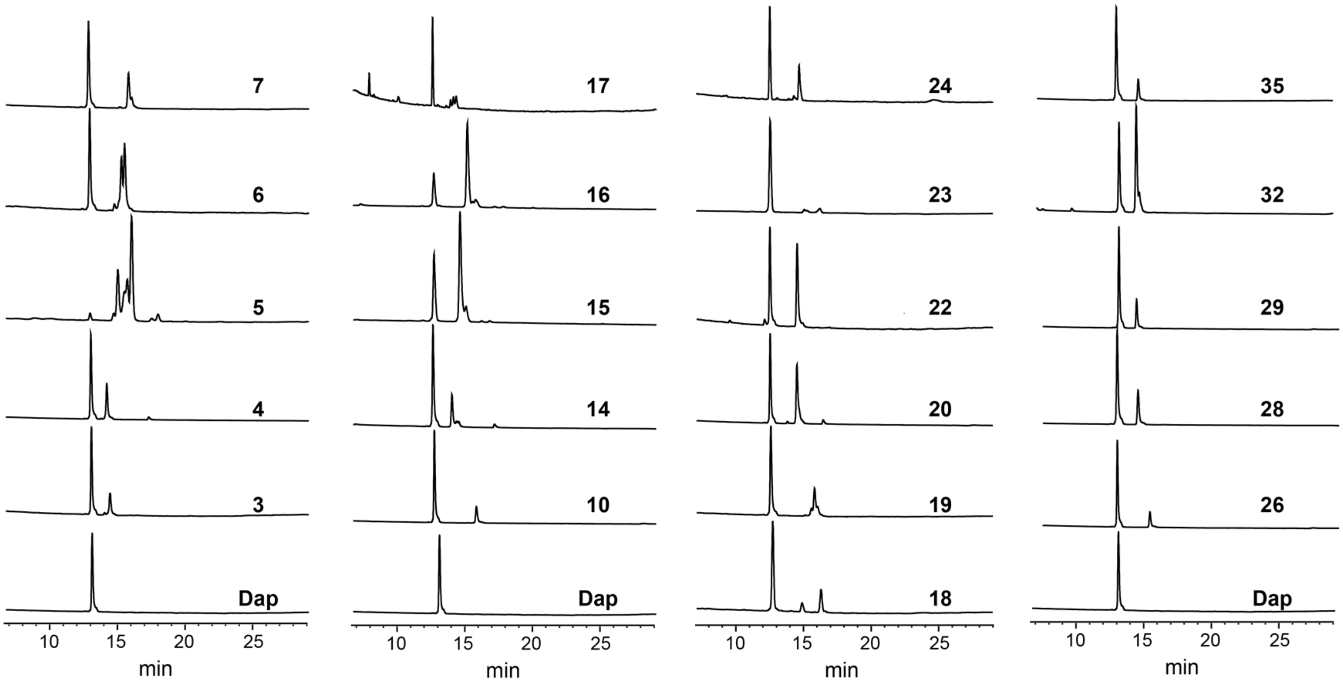

Figure S3: RP-HPLC plots of purified alkyl-**Dap** analogs (the chromatogram of **45** shows decomposition).

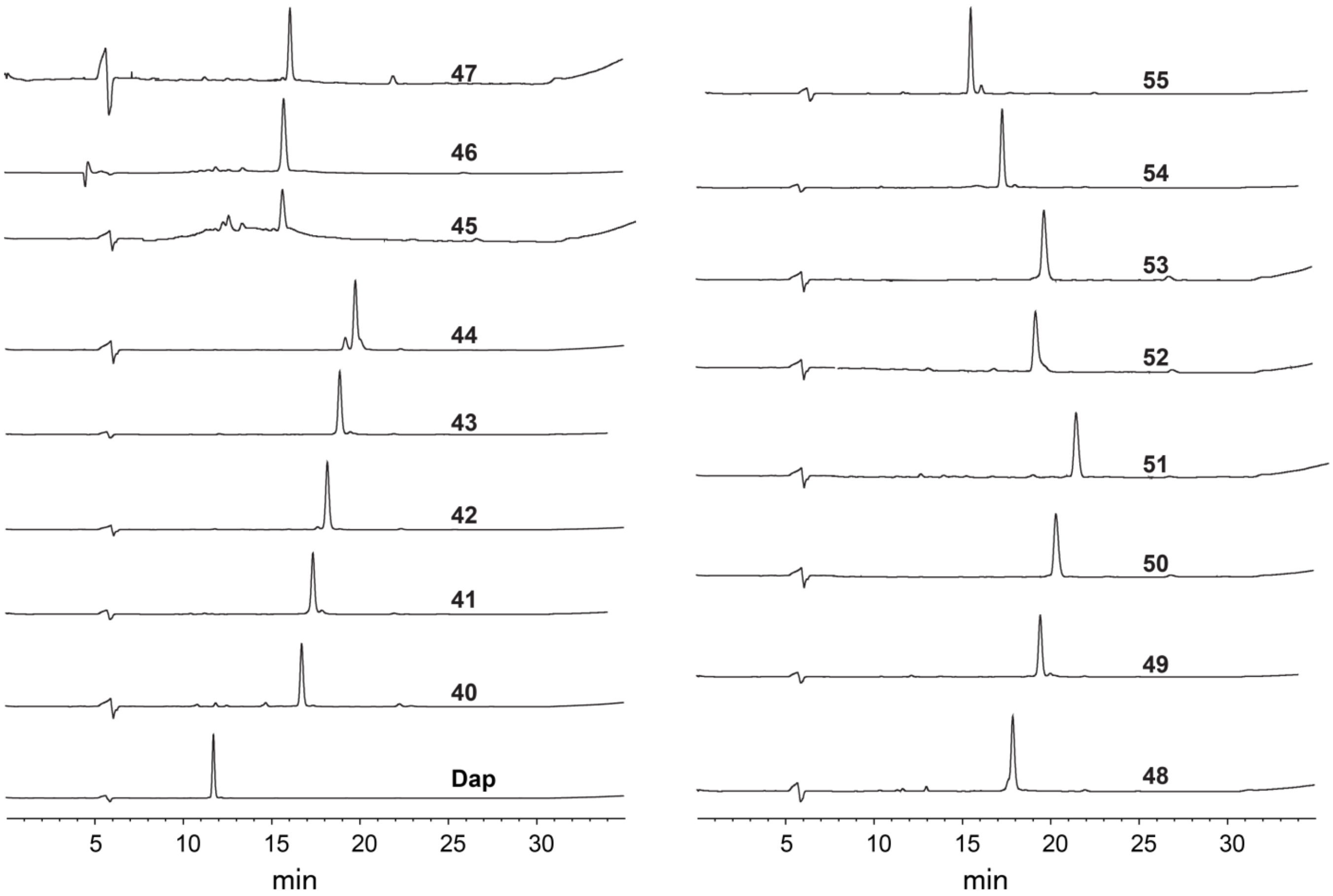

Table S3. Summary of NMR chemical shift assignments of the alkyl-**Dap** analogs in DMSO-d_6 (600 or 800 MHz) where δ_H and δ_C values for only Trp_1 of **Dap** along with the alkyl attachment are given (in ppm).

= COSY and = HMBC correlations.

	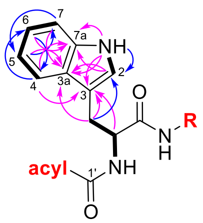		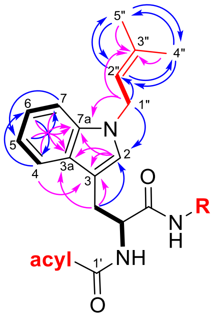		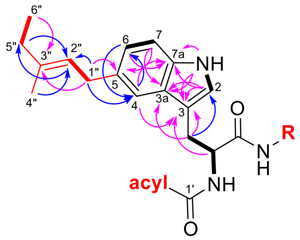		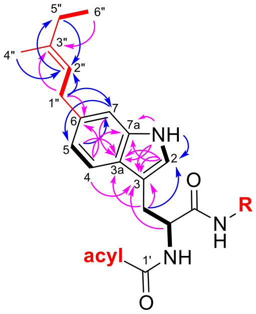	
**Position**	**Dap**		***N1*-5-Dap (40)**		***C5*-6-Dap** (45% in **41**)		***C6*-6-Dap** (55% in **41**)	
	***δ*C , type**	***δ*H**	***δ*C, type**	***δ*H**	***δ*C, type**	***δ*H**	***δ*C, type**	***δ*H**
NH		8.03		8.03		8.03		8.03
α	54.3, CH	4.37	54.2, CH	4.36	54.4, CH	4.34	54.4, CH	4.34
β	27.0, CH2	3.03, 2.91	27.0, CH2	3.00, 2.90	26.9, CH2	3.02, 2.90	26.9, CH2	3.02, 2.90
1		10.77				10.65		10.60
2	123.8, CH	7.15	126.6, CH	7.13	123.7, CH	7.10	123.1, CH	7.07
3	109.8, C		109.5, C		109.4, C		109.7, C	
3a	127.2, C		127.8, C		127.5, C		125.3, C	
4	118.2, CH	7.55	118.7, CH	7.57	117.1, CH	7.31	118.1, CH	7.45
5	118.3, CH	6.96	118.3, CH	7.01	130.9,C		119.2, CH	6.79
6	120.8, CH	7.05	120.9, CH	7.11	121.6, CH	6.85	133.9, C	
7	111.3, CH	7.31	109.6, CH	7.34	111.0, CH	7.21	110.3, CH	7.08
7a	136.1, C		135.7, C		134.4, C		136.4, C	
C=O	172.0, C		172.0, C		171.7, C		171.7, C	
1"			43.4, CH2	4.69	33.9, CH2	3.37	33.6, CH2	3.37
2"			120.6, CH	5.28	123.3, CH	5.32	122.7, CH	5.32
3"			135.1, C		136.2, C		136.2, C	
4"			17.8, CH3	1.80	15.9, CH3	1.72	15.9, CH3	1.71
5"			25.4, CH3	1.70	31.7, CH2	2.01	31.7, CH2	2.01
6"					12.6, CH3	0.97	12.6, CH3	0.97

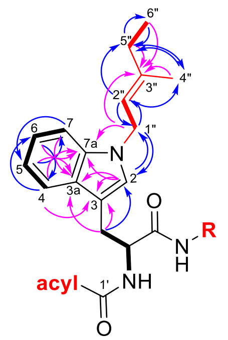

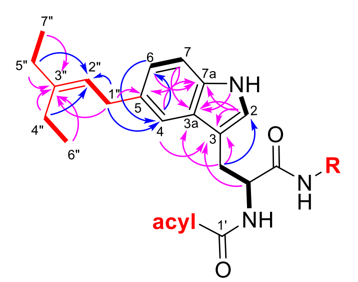

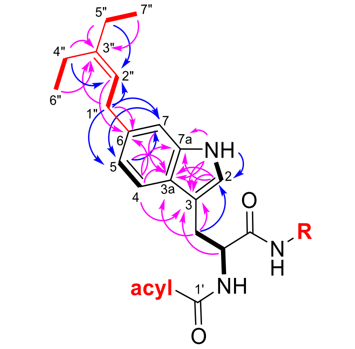

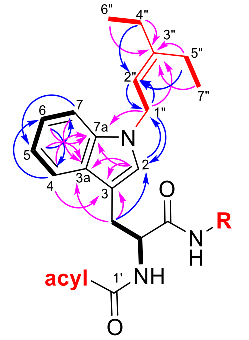

Position	***N1*-6-Dap** (**42**)		***C5*-7-DAP** (15% in **43**)		***C6*-7-Dap** (85% in **43**)		***N1*-7-Dap** (90% in **44**)	
	**δC, type**	**δH**	**δC, type**	**δH**	**δC, type**	**δH**	**δC, type**	**δH**
NH		8.03		7.99		7.99		7.99
α	54.1, CH	4.37	54.2, CH	4.35	54.2, CH	4.35	54.1, CH	4.37
β	26.9, CH2	3.02, 2.90	27.0, CH2	2.98, 2.88	27.0, CH2	2.98, 2.88	27.0, CH2	2.98, 2.88
1				10.65		10.61		
2	126.4, CH	7.13	123.7, CH	7.10	123.1, CH	7.08	126.4, CH	7.13
3	109.6, C		109.4, C		109.7, C		109.5, C	
3a	127.7, C		127.5, C		125.3, C		127.7, C	
4	118.6, CH	7.57	117.1, CH	7.31	118.1, CH	7.45	118.6, CH	7.57
5	118.3, CH	6.99	130.9,C		119.2, CH	6.80	118.3, CH	7.00
6	120.8, CH	7.10	121.6, CH	6.86	134.0, C		120.9, CH	7.11
7	109.7, CH	7.33	111.0, CH	7.22	110.3, CH	7.09	109.5, CH	7.35
7a	135.6, C		134.4, C		136.4, C		135.7, C	
C=O	171.7, C		171.7, C		171.7, C		171.7, C	
1"	43.2, CH2	4.69	33.4, CH2	3.39	33.4, CH2	3.39	42.9, CH2	4.73
2"	118.8, CH	5.28	122.3, CH	5.26	122.3, CH	5.26	118.4, CH	5.21
3"	140.2, C		141.8, C		141.8, C		145.7, C	
4"	16.0, CH3	1.81	22.7, CH2	2.14	22.7, CH2	2.14	23.0, CH2	2.24
5"	31.5, CH2	2.01	28.7, CH2	2.03	28.7, CH2	2.03	28.4, CH2	2.03
6"	12.2, CH3	0.96	13.1, CH3	0.99	13.1, CH3	0.99	13.1, CH3	1.02
7"			12.7, CH3	0.97	12.7, CH3	0.97	12.4, CH3	0.95

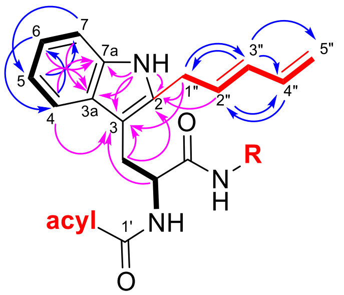

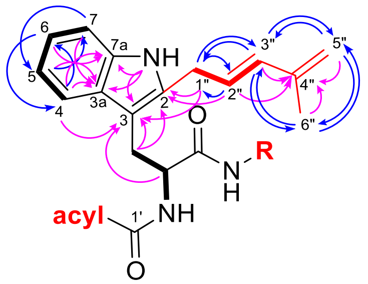

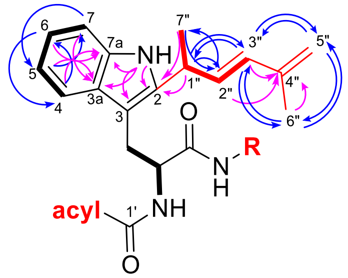

Position	*C2*-15-Dap (45)		*C2*^{R}-16-Dap (46)		*C2*^{R}-18-Dap (47)	
	***δ*C, type**	***δ*H**	***δ*C, type**	***δ*H**	***δ*C, type**	***δ*H**
NH		7.92		7.92		7.92
α	54.6, CH	4.32	54.4, CH	4.32	54.4, CH	4.32
β	26.5, CH2	3.01, 2.89	26.3, CH2	3.01, 2.89	26.3, CH2	3.01, 2.89
1		10.68		10.68		10.69
2	134.7, C		134.7, C		139.4, C	
3	106.1, C		105.8, C		105.1, C	
3a	128.1, C		128.1, C		127.9, C	
4	118.1, CH	7.52	117.9, CH	7.52	118.0, CH	7.50
5	118.1, CH	6.92	118.0, CH	6.92	118.0, CH	6.91
6	120.2, CH	6.98	120.2, CH	6.98	120.2, CH	6.98
7	110.5, CH	7.23	110.5, CH	7.23	110.5, CH	7.23
7a	135.4, C		135.2, C		135.3, C	
C=O	171.7, C		171.7, C		171.7, C	
1"	28.6, CH2	3.52	28.8, CH2	3.57, 3.52	33.0, CH	3.95
2"	132.1, CH	5.87	127.8, CH	5.82	133.7, CH	5.90
3"	131.5, CH	6.12	133.4, CH	6.22	131.1, CH	6.11
4"	136.9, CH	6.32	141.3, C		141.3, C	
5"	116.0, CH2	5.00, 5.15	115.3, CH2	4.91	115.5, CH2	4.90
6"			18.5, CH3	1.79	18.4, CH3	1.77
7"					20.2, CH3	1.41

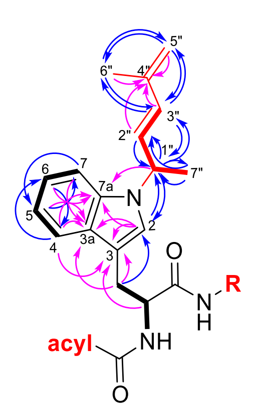

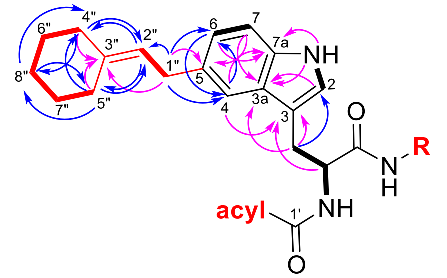

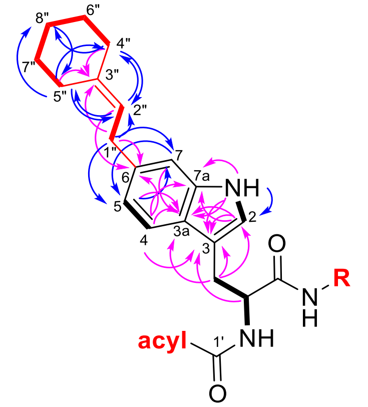

Position	*N1*^{R}-18-Dap (48)		*C5*-19-Dap (15% in 49)		*C6*-19-Dap (85% in 49)	
	*δ*C, type	*δ*H	*δ*C, type	*δ*H	*δ*C, type	*δ*H
NH		8.00		7.99		7.99
α	53.8, CH	4.43	54.2, CH	4.35	54.2, CH	4.35
β	27.1, CH2	3.03, 2.91	26.9, CH2	2.98, 2.88	26.9, CH2	2.98, 2.88
1				10.65		10.61
2	123.5, CH	7.28	123.7, CH	7.10	123.2, CH	7.07
3	109.9, C		109.3, C		109.6, C	
3a	127.6 C		127.4, C		125.3, C	
4	118.6, CH	7.57	117.2, CH	7.31	118.1, CH	7.45
5	118.4, CH	6.99	130.9, C		119.3, CH	6.79
6	120.8, CH	7.09	121.6, CH	6.86	133.9, C	
7	109.8, CH	7.42	111.1, CH	7.22	110.3, CH	7.08
7a	135.4, C		134.4, C		136.3, C	
C=O	171.7, C		171.7, C		171.7, C	
1"	51.8, CH	5.23	33.1, CH2	3.37	33.1, CH2	3.36
2"	131, CH	5.83	121.0 CH	5.26	121.0 CH	5.26
3"	132.4, CH	6.19	138.6, C		138.6, C	
4"	140.7, C		28.2, CH2	2.24	28.2, CH2	2.24
5"	117.1, CH2	4.96	36.6, CH2	2.08	36.6, CH2	2.08
6"	18.3, CH3	1.76	27.3, CH2	1.53	27.3, CH2	1.53
7"	20.2, CH3	1.57	28.2, CH2	1.51	28.2, CH2	1.51
8"			26.3, CH2	1.54	26.3, CH2	1.54

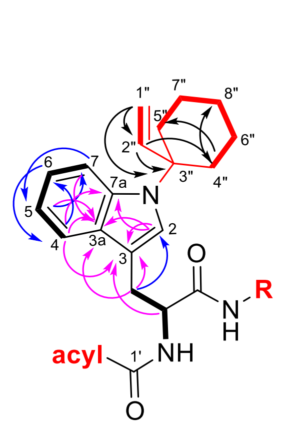

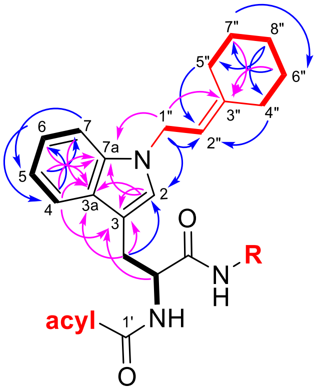

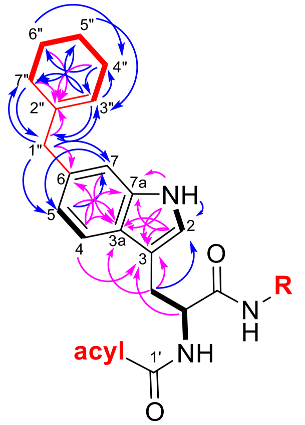

Position	***N1***^{R}**-19-Dap** (95% in **50**)		***N1*-19-Dap (51)**		***C6*-20-Dap** (95% in **52**)	
	***δ*C, type**	***δ*H**	***δ*C, type**	***δ*H**	***δ*C, type**	***δ*H**
NH		8.04		7.99		8.00
α	53.8, CH	4.44	54.0, CH	4.37	54.2, CH	4.35
β	27.0, CH2	3.02, 2.90	26.9, CH2	3.02, 2.91	26.9, CH2	2.98, 2.88
1						10.62
2	125.1, CH	7.44	126.4, CH	7.12	123.1, CH	7.09
3	108.9, C		109.4, C		109.5, C	
3a	129.0, C		127.7, C		125.5, C	
4	118.5, CH	7.57	118.5, CH	7.57	117.8, CH	7.44
5	118.0, CH	6.98	118.2, CH	7.00	119.5, CH	6.79
6	120.2, CH	7.00	120.8, CH	7.10	132.5, C	
7	113.9, CH	7.38	109.5, CH	7.34	110.9, CH	7.08
7a	134.6, C		135.6, C		136.3, C	
C=O	171.7, C		171.7, C		171.7, C	
1"	114.1, CH2	5.09. 4.75	42.4, CH2	4.69	44.3, CH2	3.26
2"	142.1, CH	6.06	117.1, CH	5.22	137.6, C	
3"	61.3, C		142.7, C		121.5, CH	5.47
4"	35.5, CH2	2.27	28.2, CH2	2.34	24.8, CH2	1.98
5"	35.4, CH2	2.09	36.2, CH2	2.06	22.3, CH2	1.51
6"	24.9, CH2	1.49, 1.45	27.1, CH2	1.56	22.1, CH2	1.48
7"	21.8, CH2	1.58, 1.50	27.9, CH2	1.49	27.4, CH2	1.80
8"	28.0, CH2	1.50	26.0, CH2	1.56		

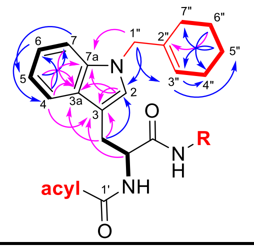

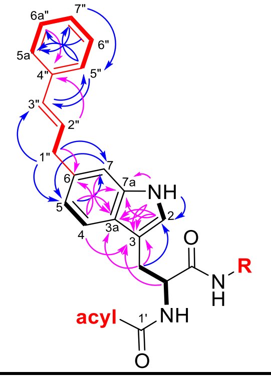

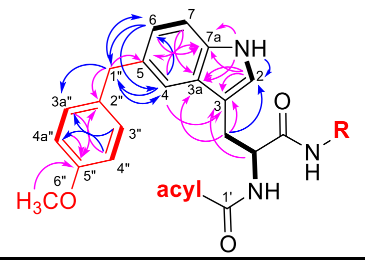

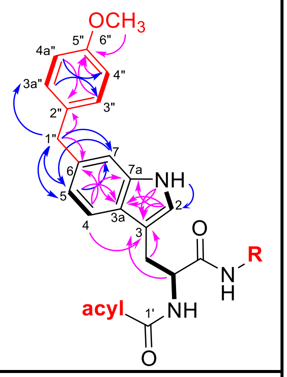

Position	***N1*-20-Dap** (95% in **53**)		***C6*-22-Dap** (85% pure **54**)		***C5*-32-Dap** (75% in **55**)		***C6*-32-Dap** (25% in **55**)	
	***δ*C, type**	***δ*H**	***δ*C, type**	***δ*H**	***δ*C, type**	***δ*H**	***δ*C, type**	***δ*H**
NH		7.99		8.01		8.01		8.01
α	54.1, CH	4.37	54.2, CH	4.35	54.2, CH	4.36	54.2, CH	4.36
β	27.0, CH2	2.98, 2.88	26.9, CH2	2.98, 2.88	26.9, CH2	2.99, 2.89	26.9, CH2	2.99, 2.89
1				10.67		10.68		10.68
2	127.3, CH	7.09	123.3, CH	7.09	123.7, CH	7.10	123.1, CH	7.08
3	109.5, C		109.5, C		109.4, C		109.4, C	
3a	127.6, C		125.6, C		127.4, C		125.5, C	
4	118.5, CH	7.57	118.2, CH	7.49	117.7, CH	7.39	118.1, CH	7.45
5	118.2, CH	6.98	119.3, CH	6.88	131.2, C		119.6, CH	6.84
6	120.9, CH	7.08	132.4, C		122.0, CH	6.86	134.4, C	
7	109.8, CH	7.35	110.7, CH	7.17	111.1, CH	7.20	110.8, CH	7.07
7a	136.1, C		136.4, C		134.6, C		136.4, C	
C=O	171.7, C		171.7, C		171.7, C		171.7, C	
1"	51.6, CH2	4.58	38.9, CH2	3.58	40.6, CH2	3.92	40.6, CH2	3.92
2"	134.6, C		130.3, CH	6.45	134.5, C		134.5, C	
3"	122.9, CH	5.48	129.7, CH	6.48	129.5, CH	7.13, 7.13	129.5, CH	7.13, 7.13
4"	24.3, CH2	1.94	137.1, C		113.5, CH	6.82, 6.82	113.5, CH	6.82, 6.82
5"	21.9, CH2	1.47	125.7, CH	7.39, 7.39	157.2, C		157.2, C	
6"	22.0, CH2	1.48	128.4, CH	7.30, 7.30	54.8, CH3	3.69	54.8, CH3	3.69
7"	25.4, CH2	1.74	126.9, CH	7.20				

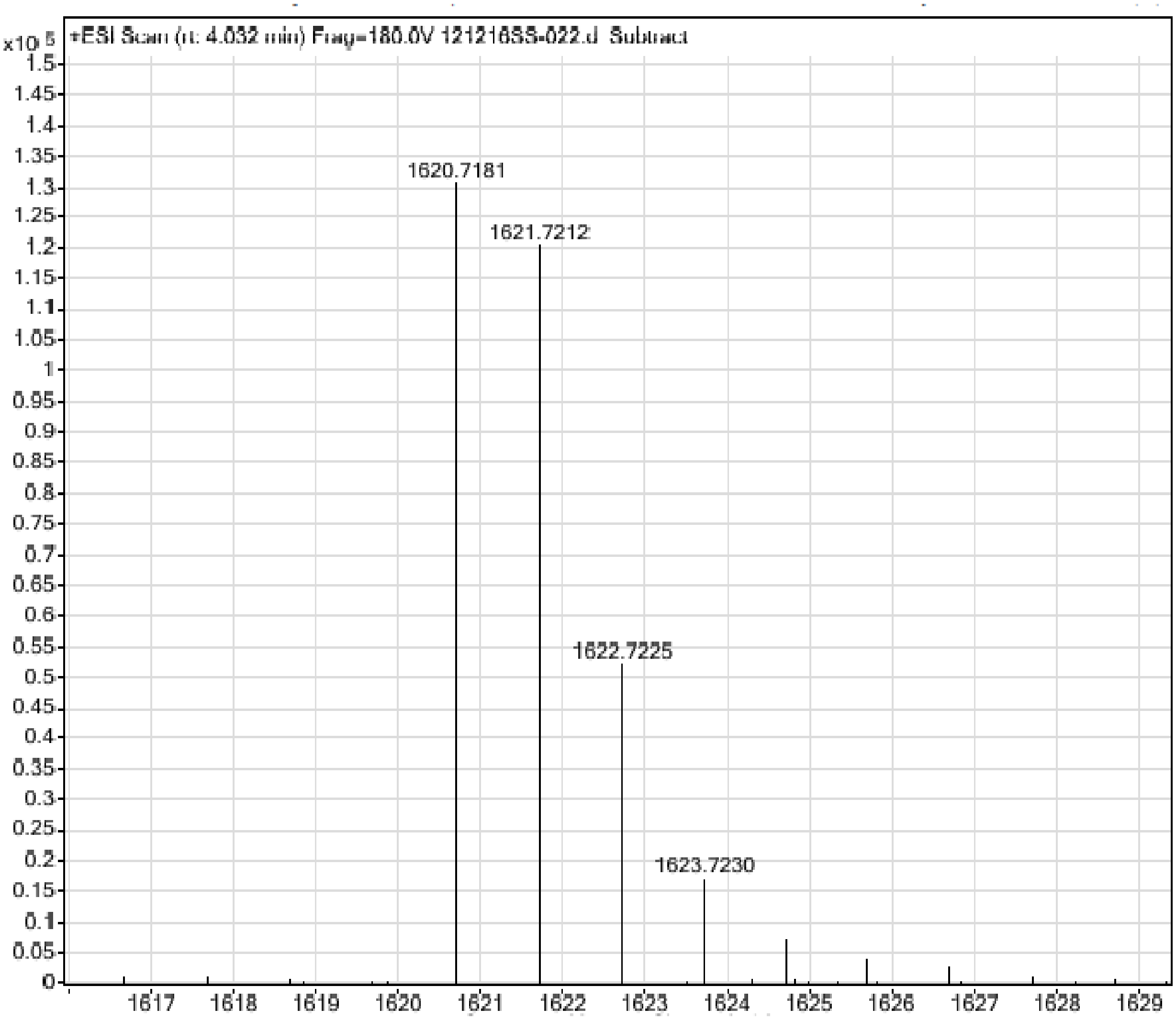

+ESI Scan (rt: 4.032 min) Frag=180.0V 12121633-022.d Subtract
x10 5
1620.7181
1621.7212
1622.7225
1623.7230

HRMS of Dap

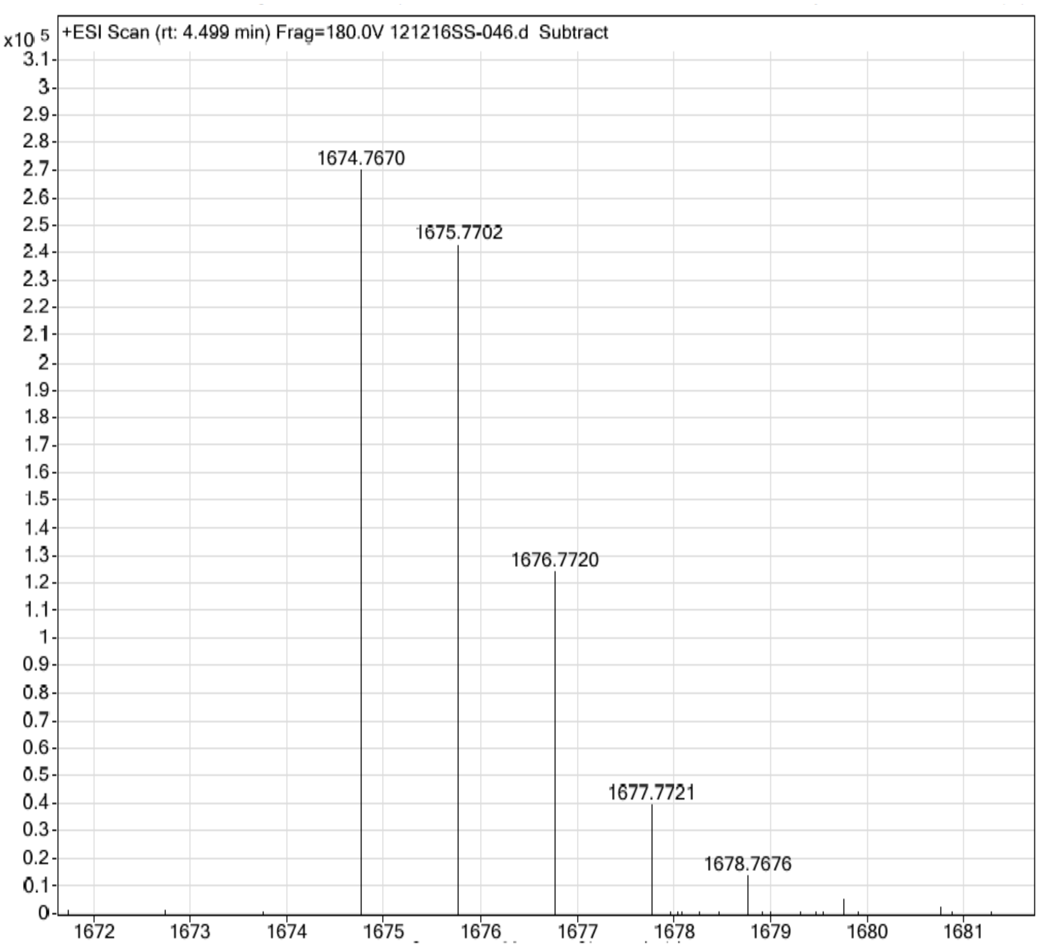

x10 5
+ESI Scan (rt: 4.499 min) Frag=180.0V 121216SS-046.d Subtract
1674.7670
1675.7702
1676.7720
1677.7721
1678.7676
3.1
3
2.9
2.8
2.7
2.6
2.5
2.4
2.3
2.2
2.1
2
1.9
1.8
1.7
1.6
1.5
1.4
1.3
1.2
1.1
1
0.9
0.8
0.7
0.6
0.5
0.4
0.3
0.2
0.1
0
1672
1673
1674
1675
1676
1677
1678
1679
1680
1681

HRMS of 3-Dap

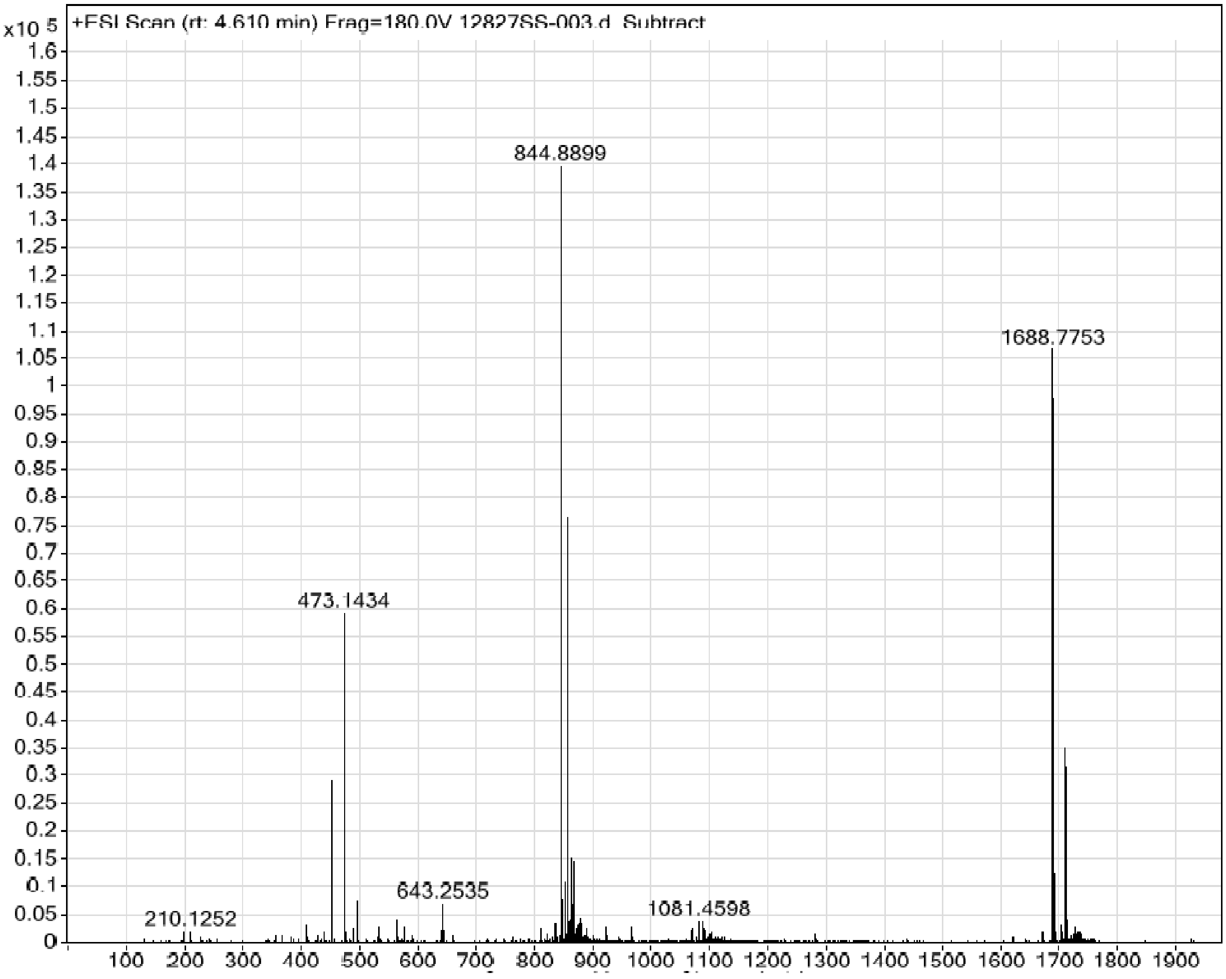
x10 5
+ESI Scan (rt: 4.610 min) Frag=180.0V 12827SS-003.d Subtract
844.8899
1688.7753
473.1434
643.2535
1081.4598
210.1252

HRMS of 4-Dap

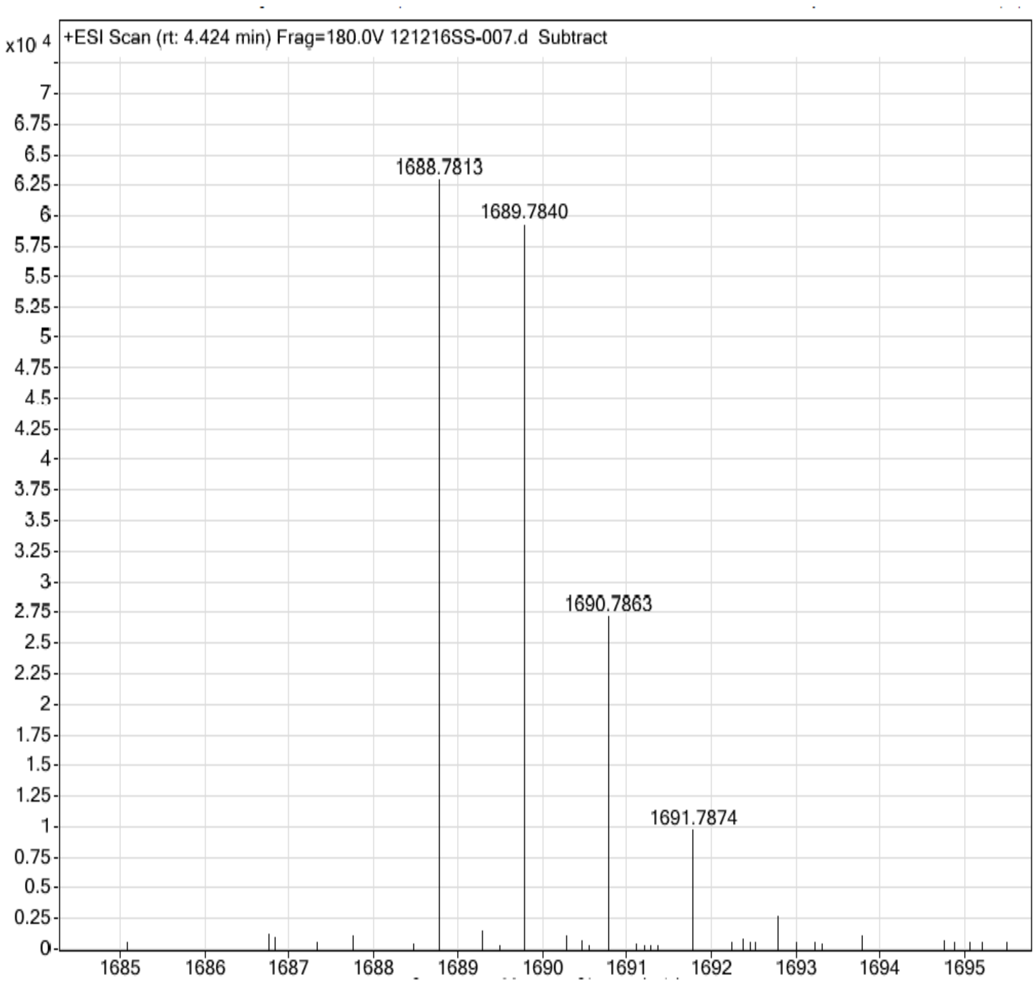

HRMS of 5-Dap (a)

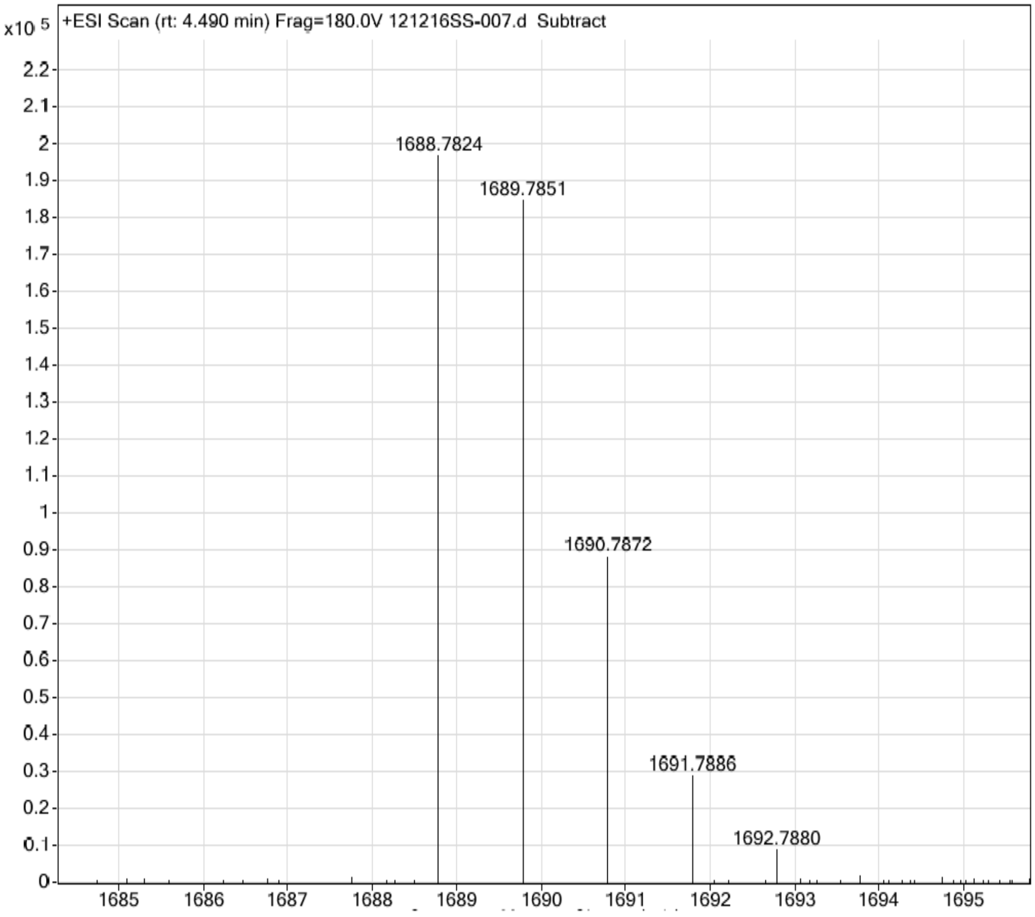

HRMS of 5-Dap (b)

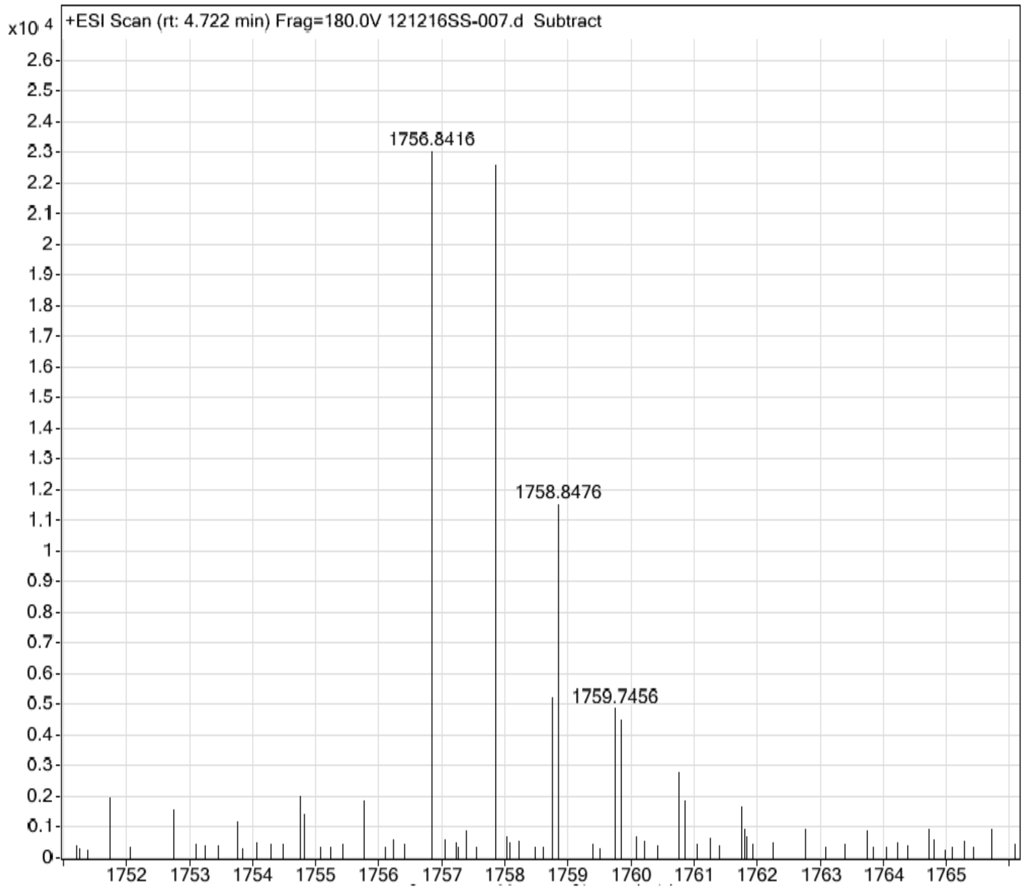

HRMS of 5-Dap (c#)

#di-alkylated Dap

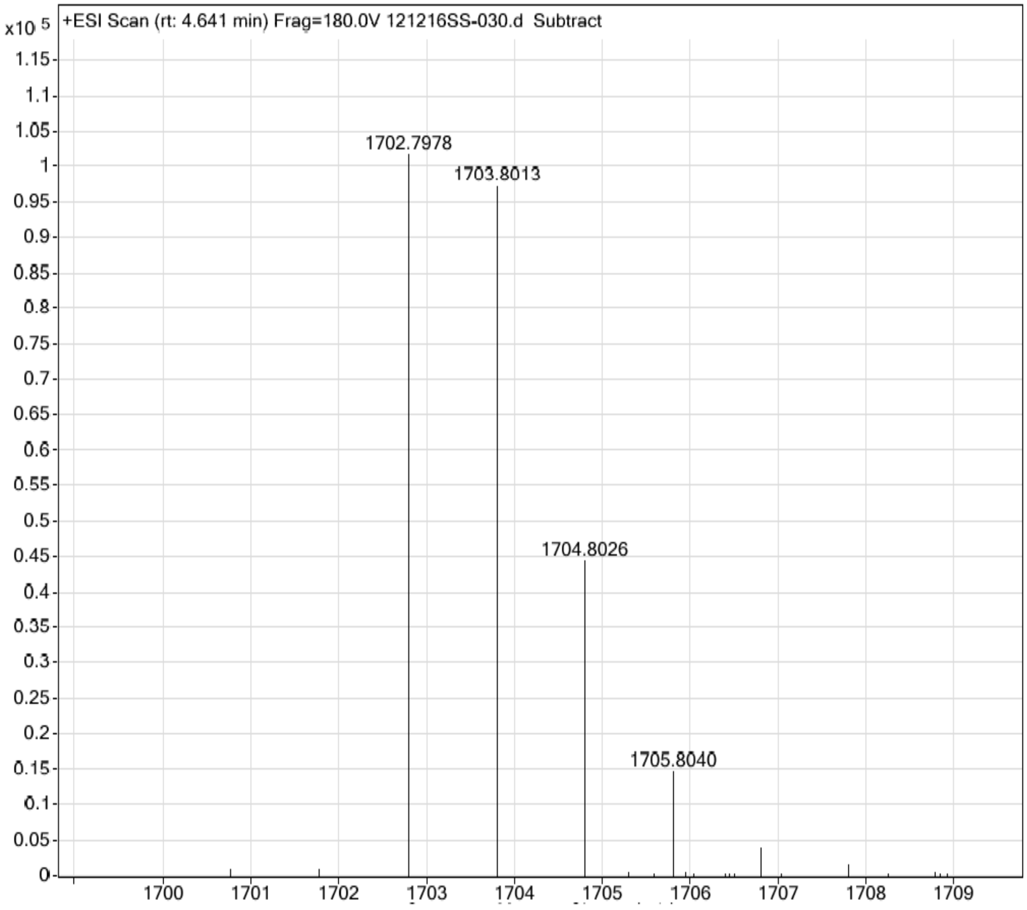

HRMS of 6-Dap (a)

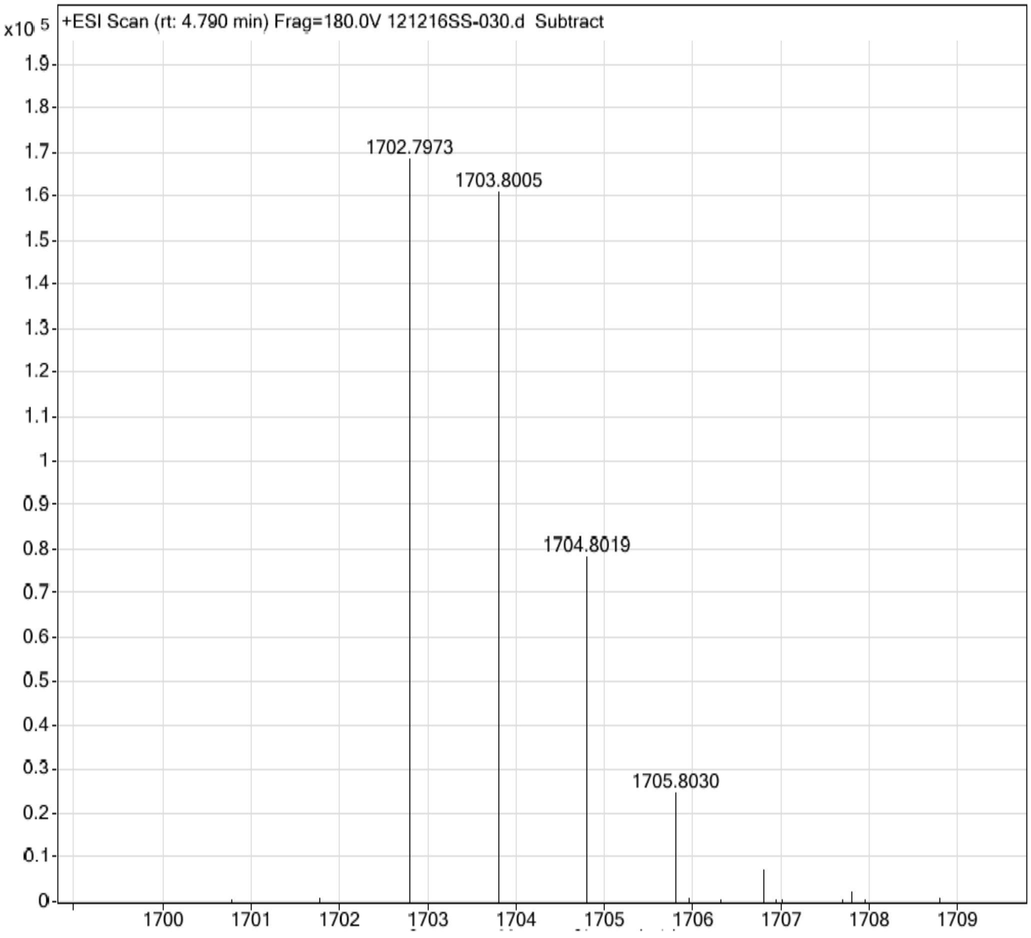

HRMS of 6-Dap (b)

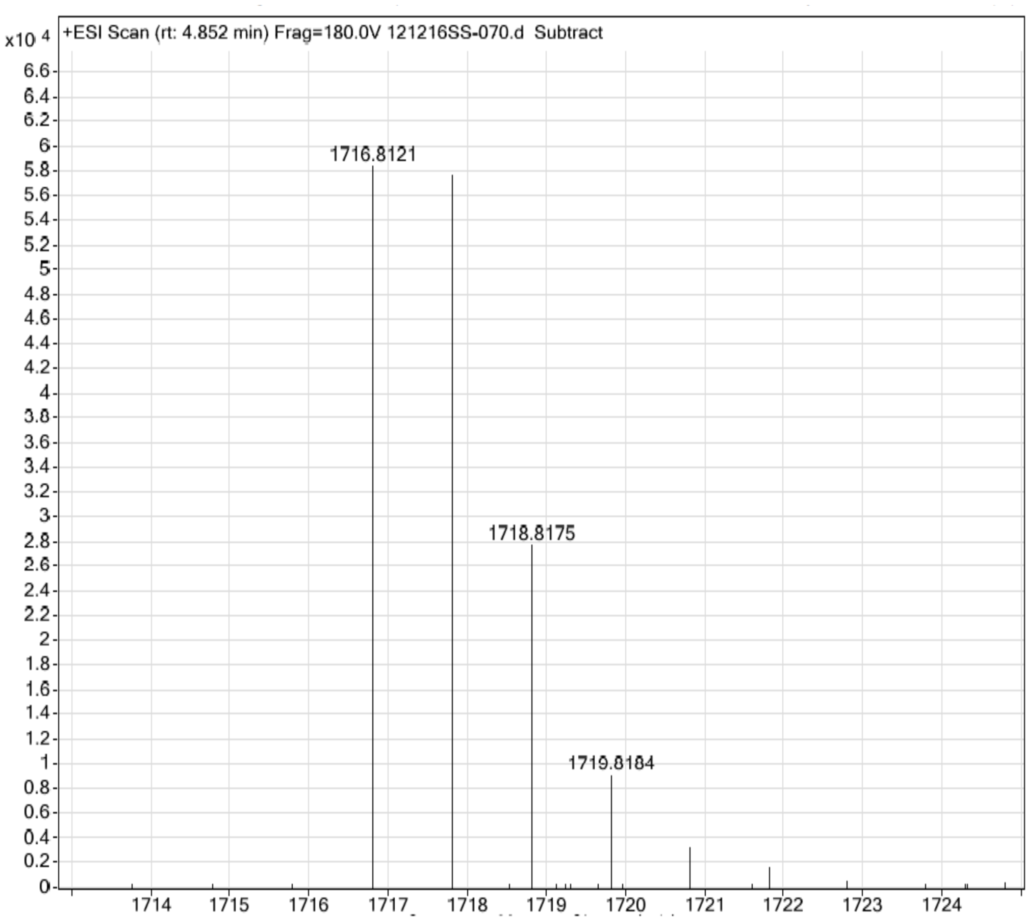
+ESI Scan (rt: 4.852 min) Frag=180.0V 121216SS-070.d Subtract
x10 4
1716.8121
1718.8175
1719.8184
1714
1715
1716
1717
1718
1719
1720
1721
1722
1723
1724

HRMS of 7-Dap

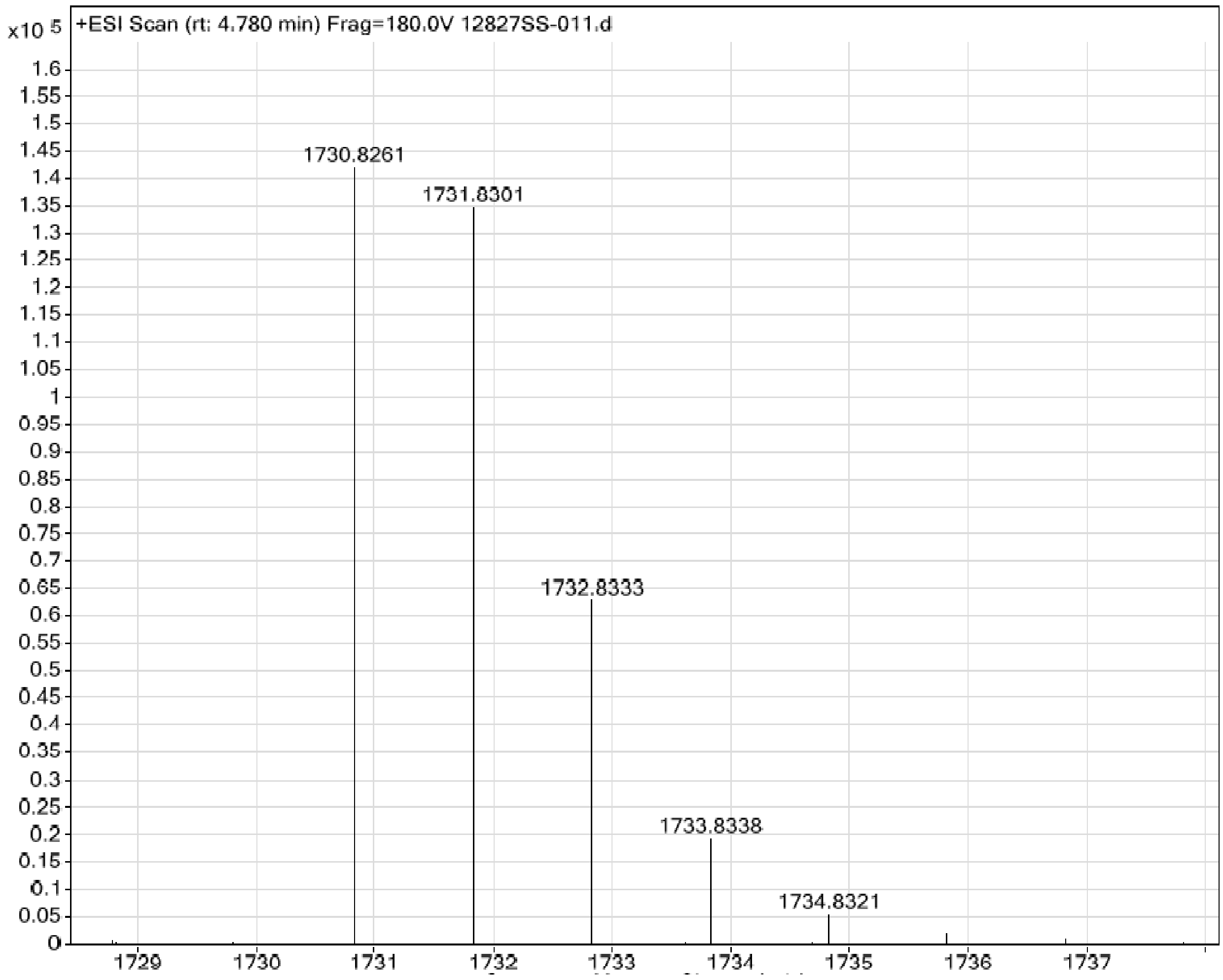

HRMS of 10-Dap

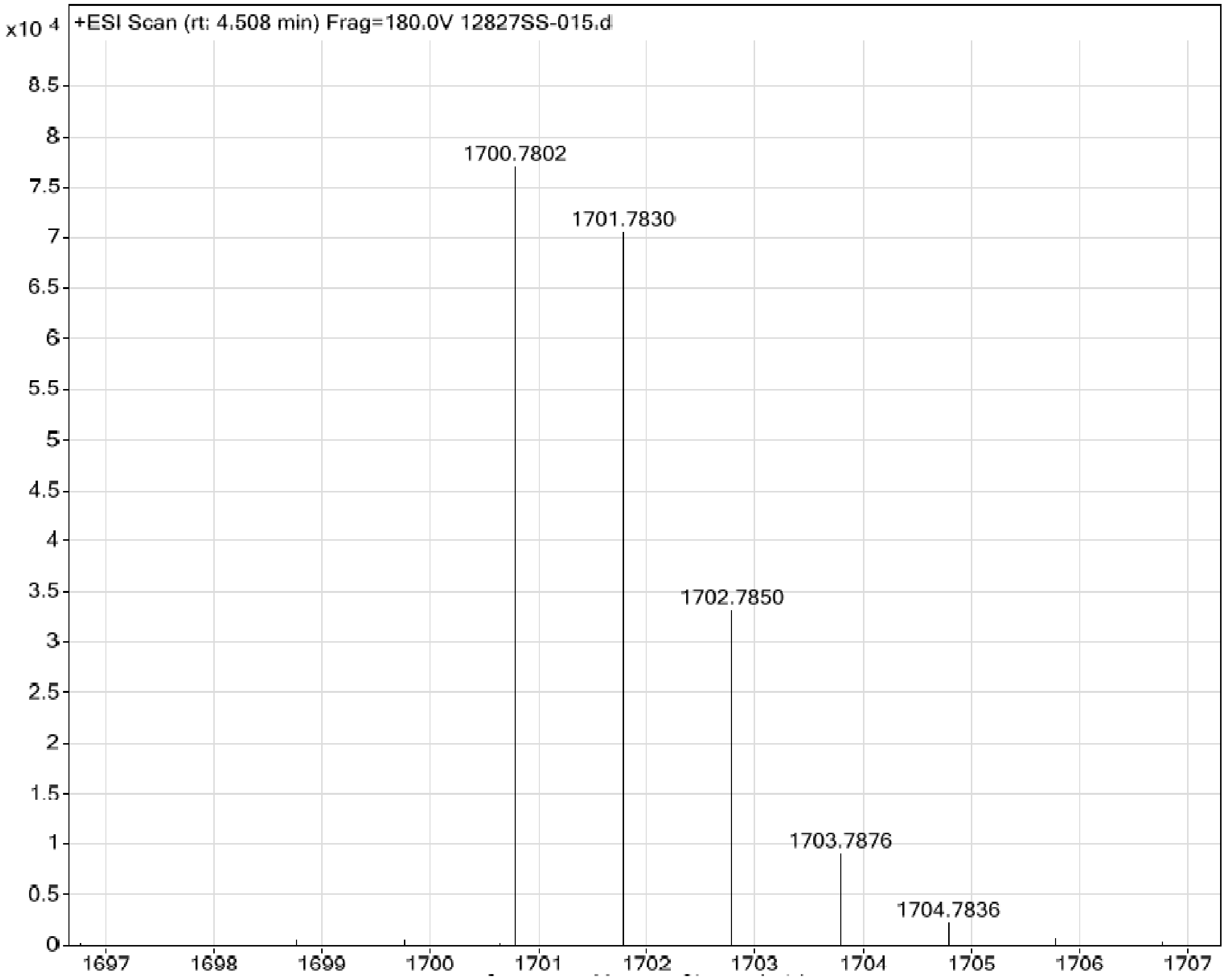

HRMS of 14-Dap

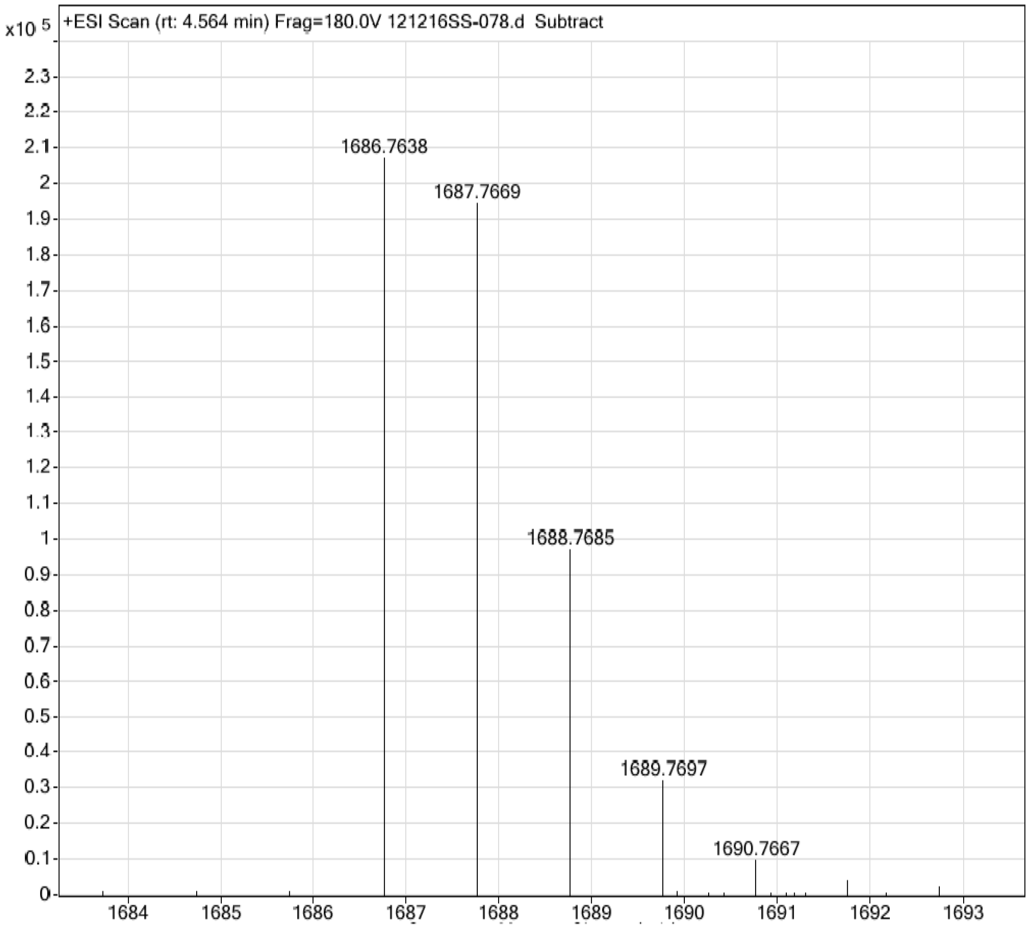

HRMS of 15-Dap (a)

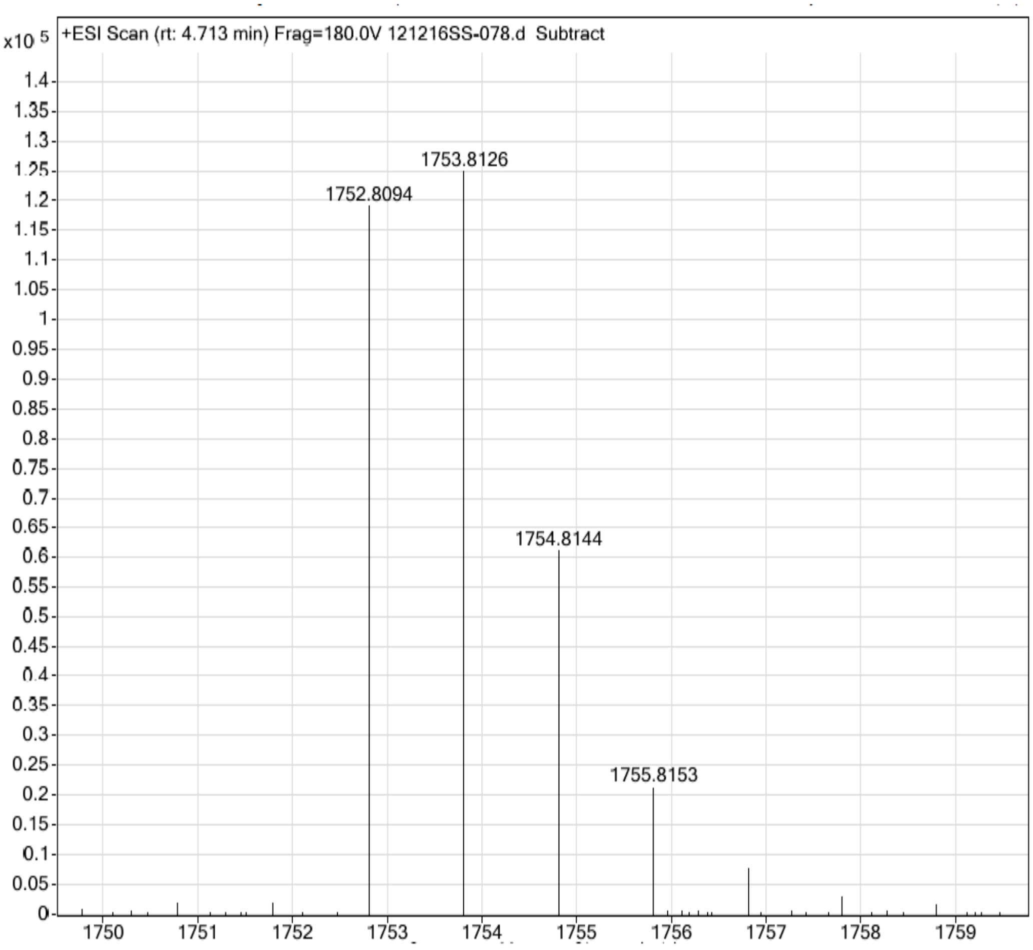

HRMS of 15-Dap (b)

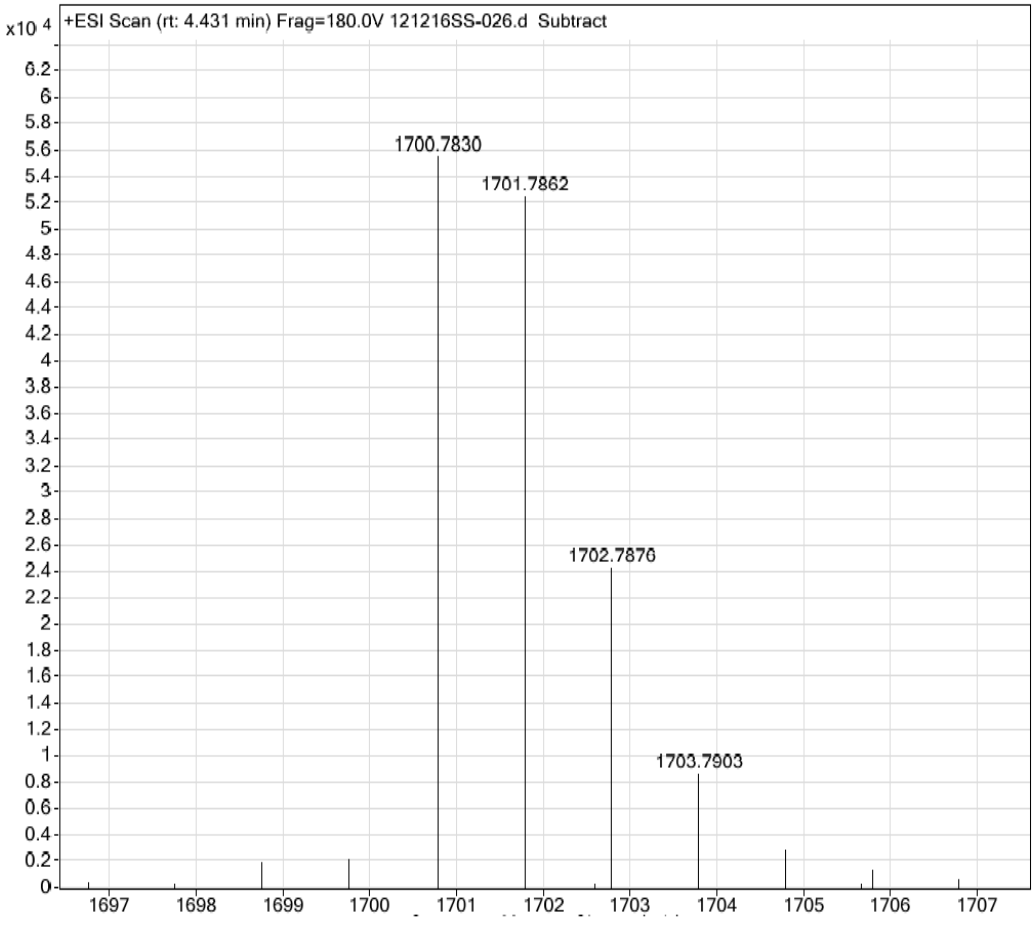

HRMS of 16-Dap (a)

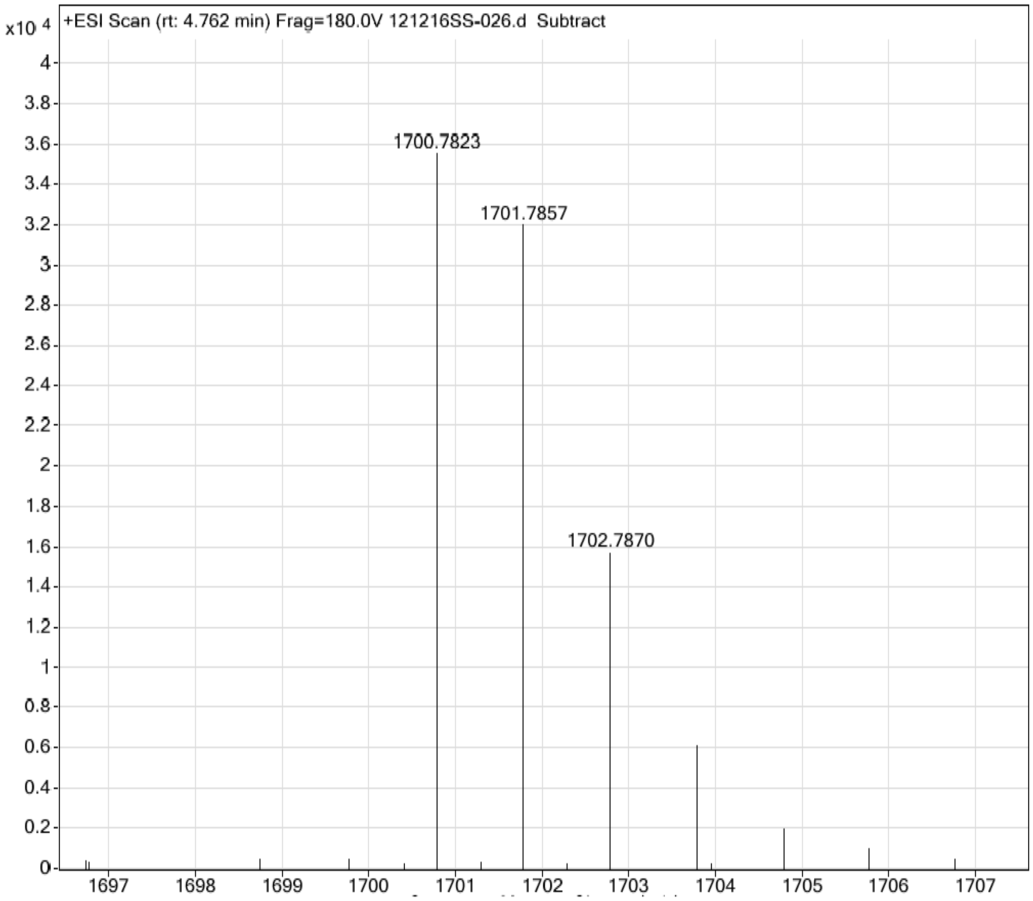

HRMS of 16-Dap (b)

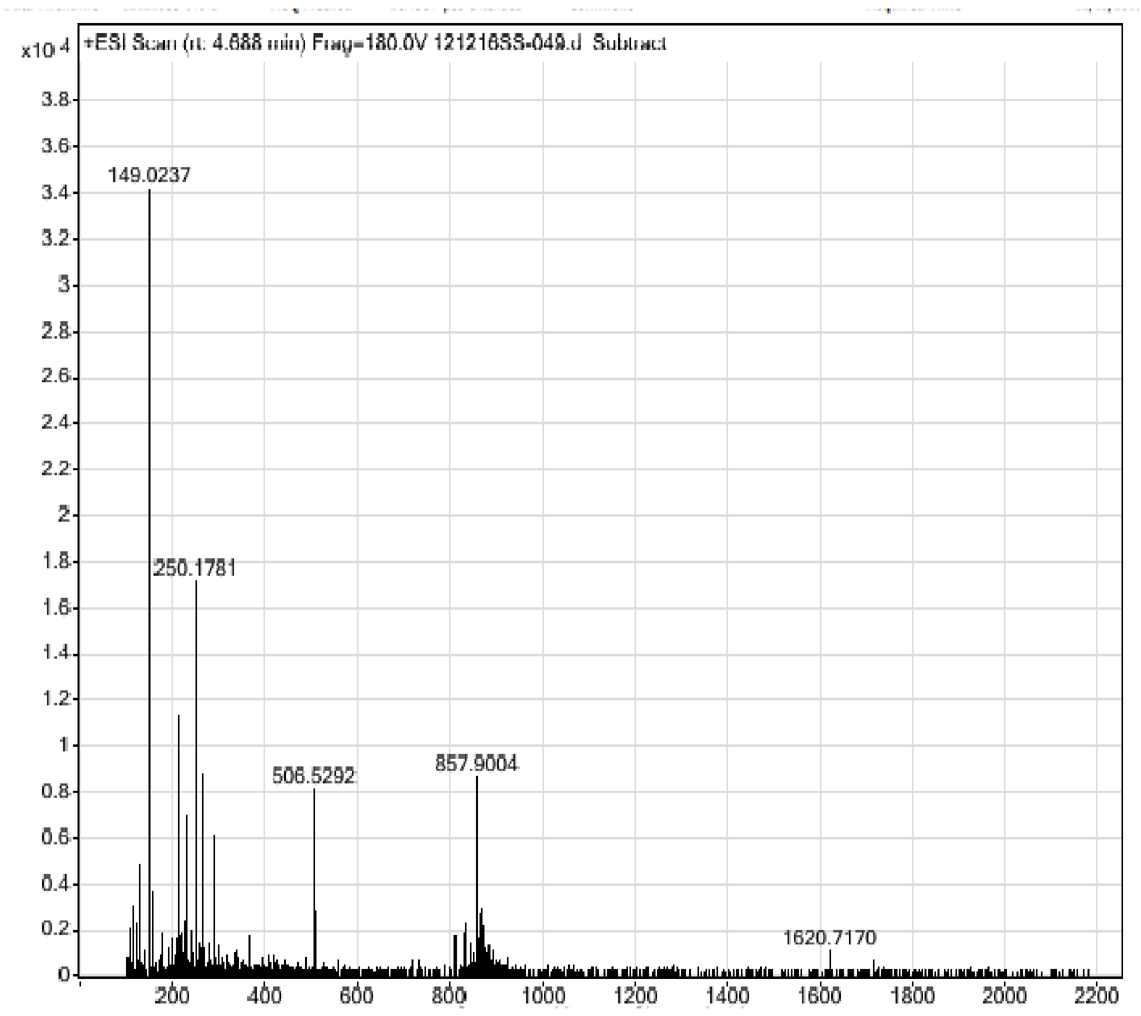

HRMS of 17-Dap*

***[M+2H]⁺**

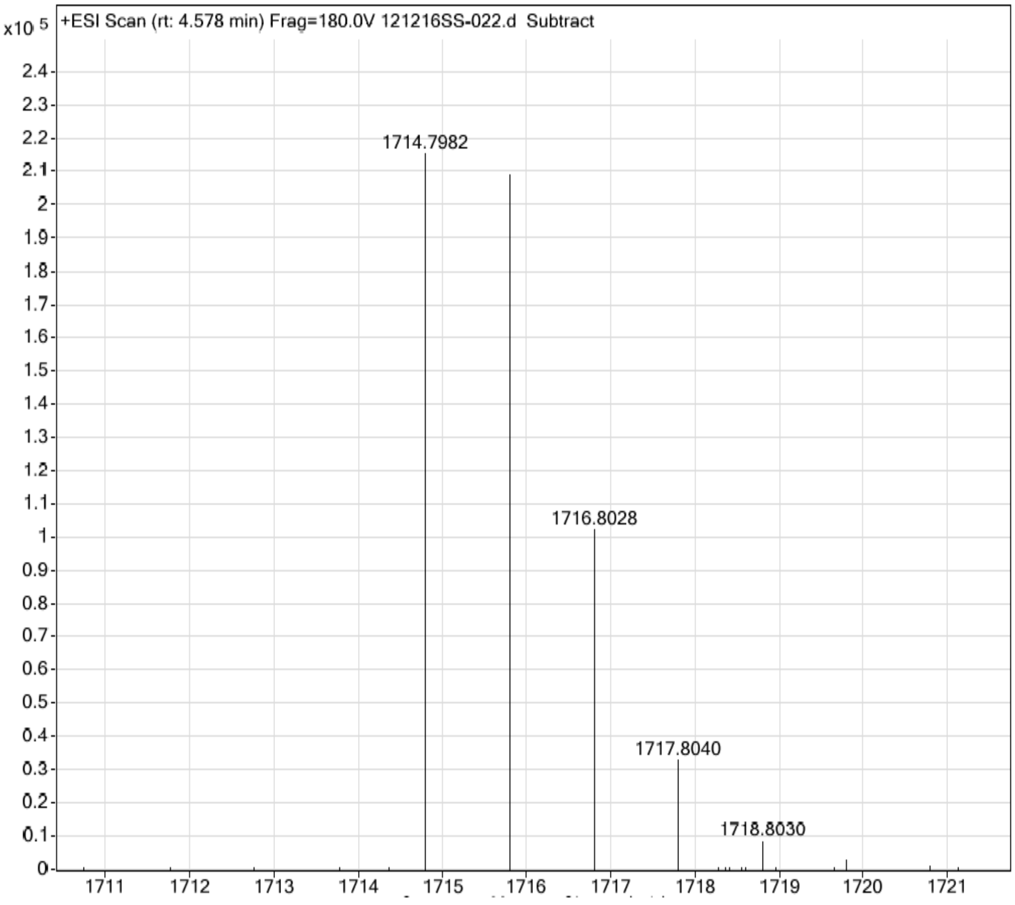

HRMS of 18-Dap (a)

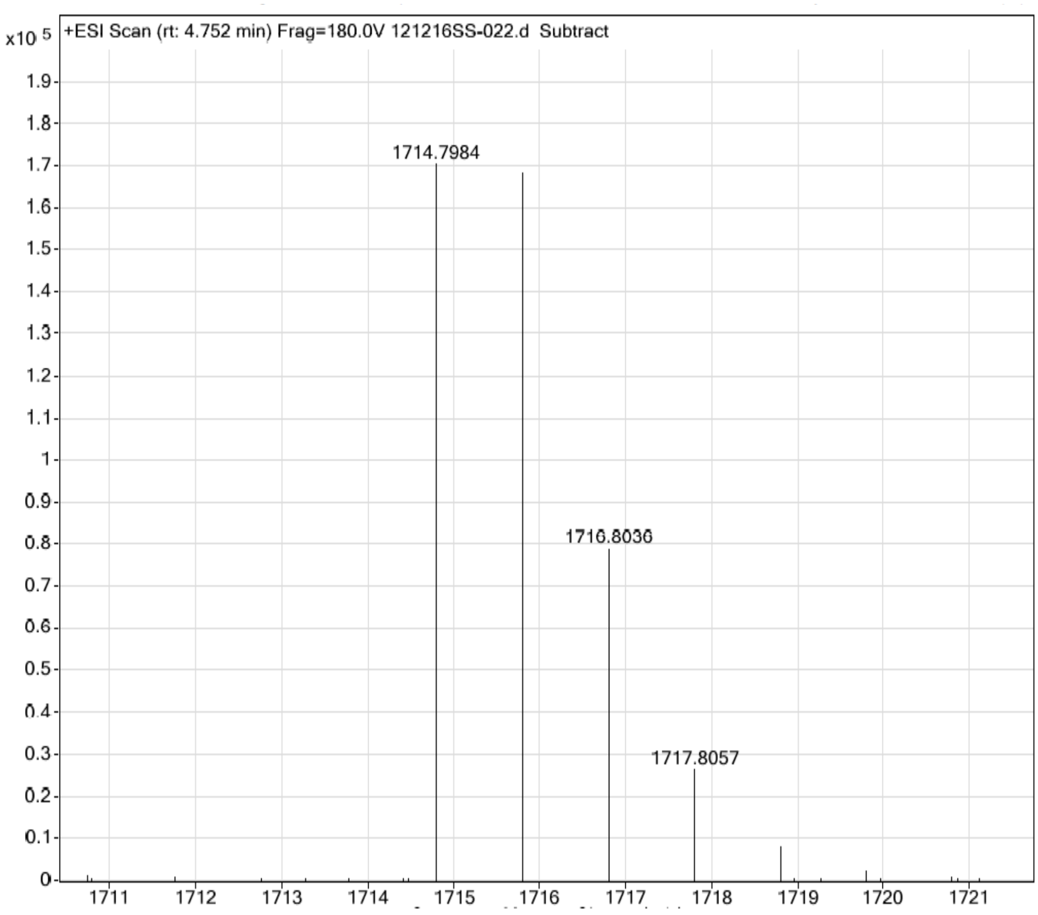

HRMS of 18-Dap (b)

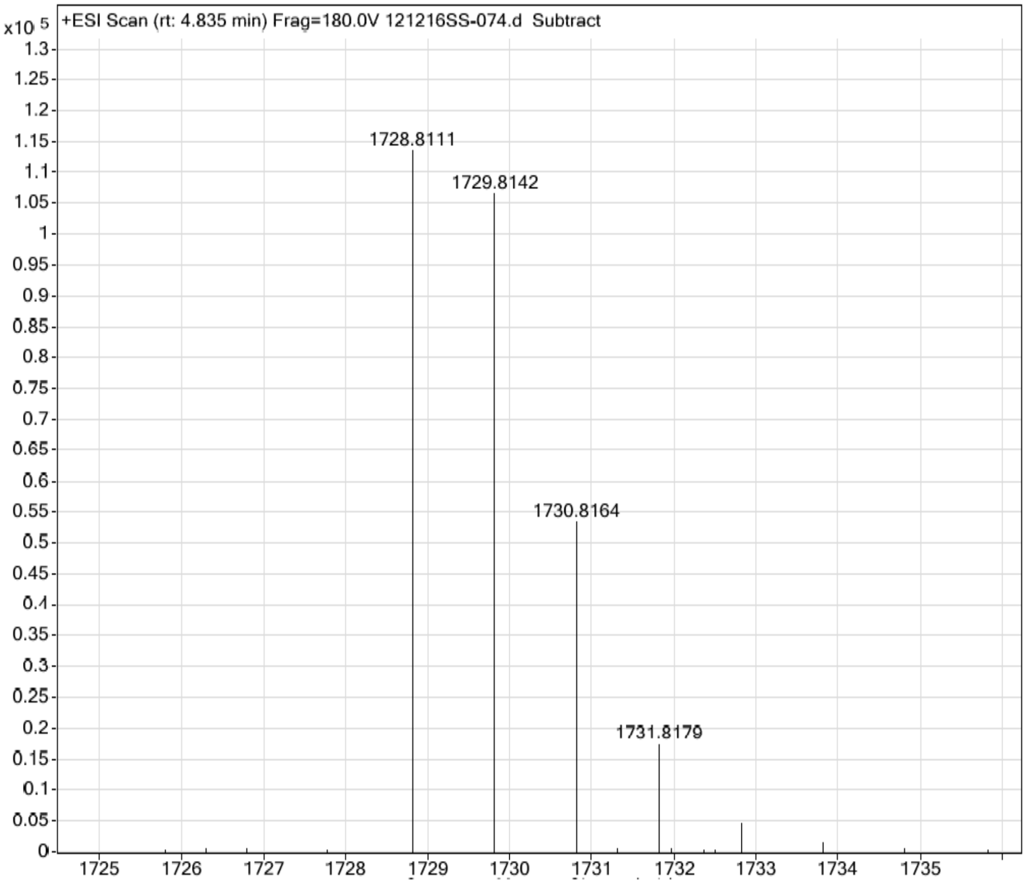

HRMS of 19-Dap

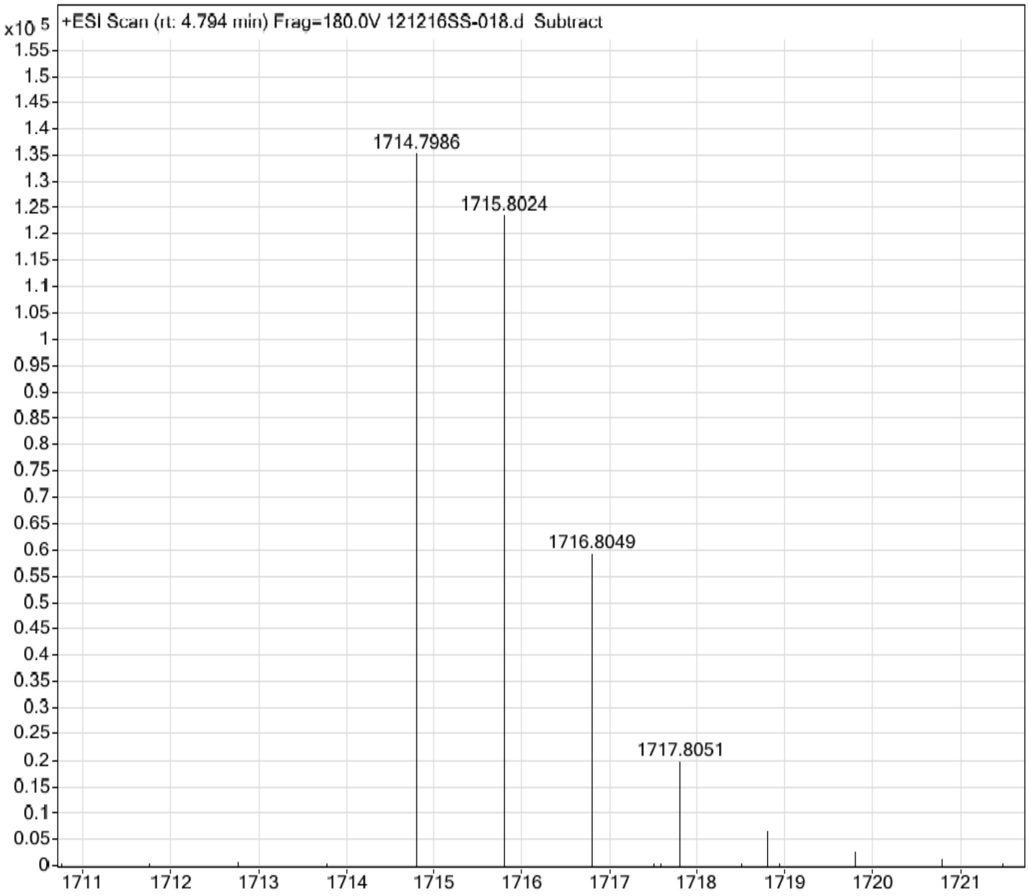

HRMS of 20-Dap

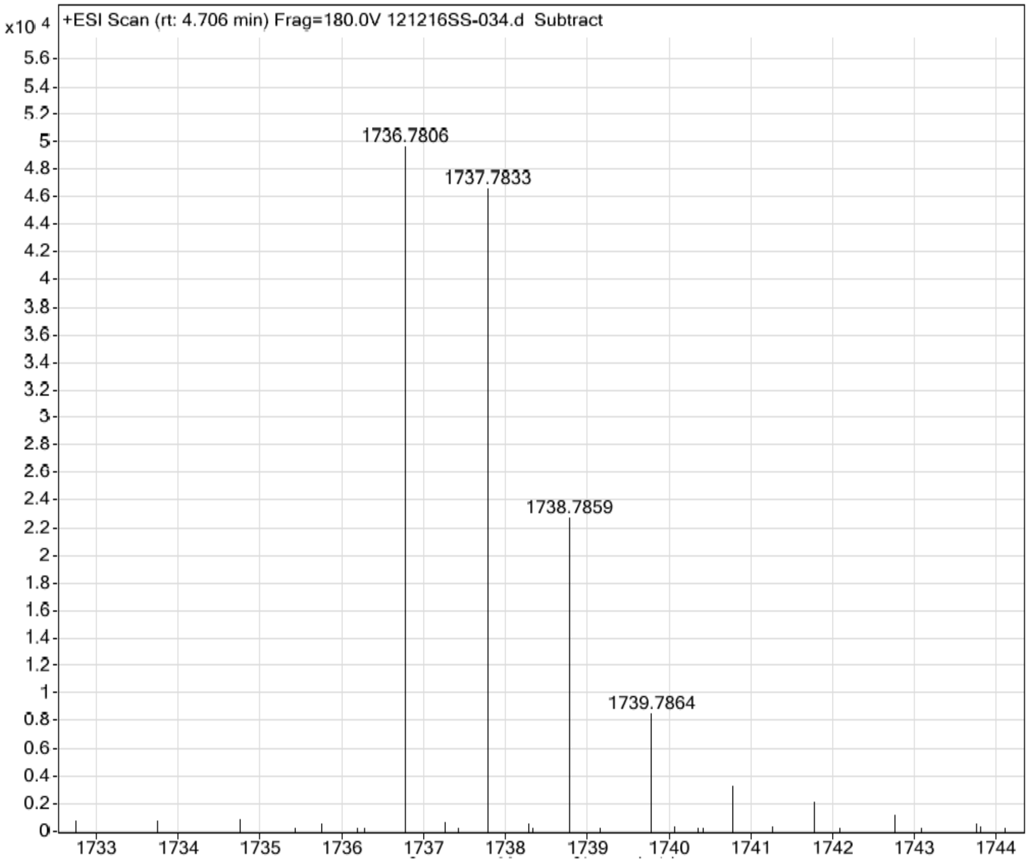

HRMS of 22-Dap

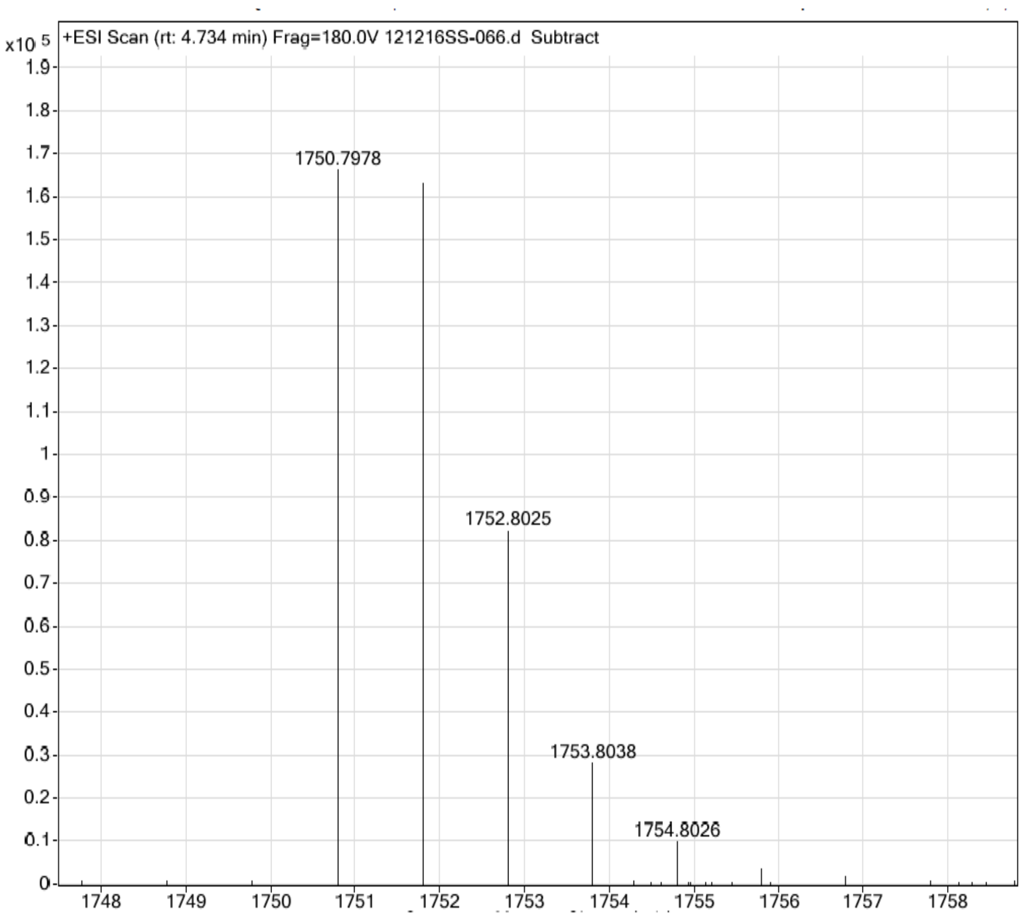

HRMS of 23-Dap

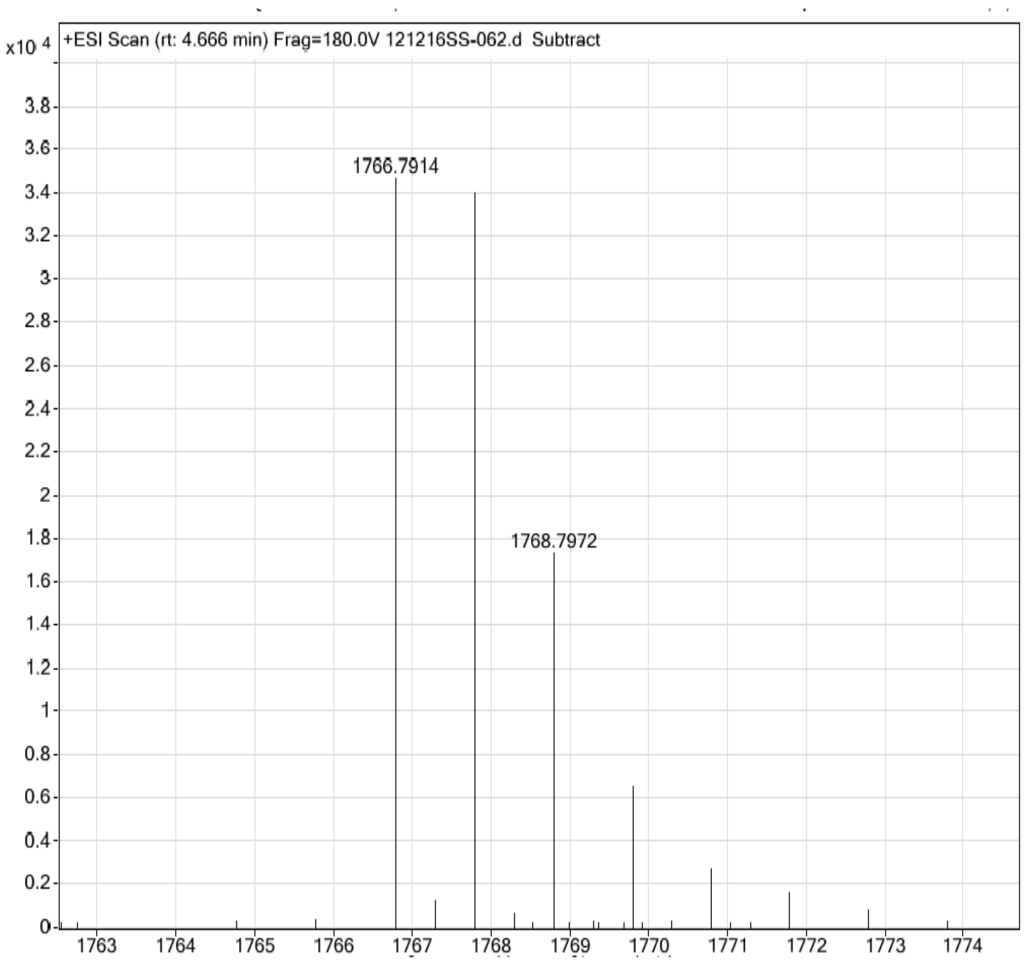

HRMS of 24-Dap

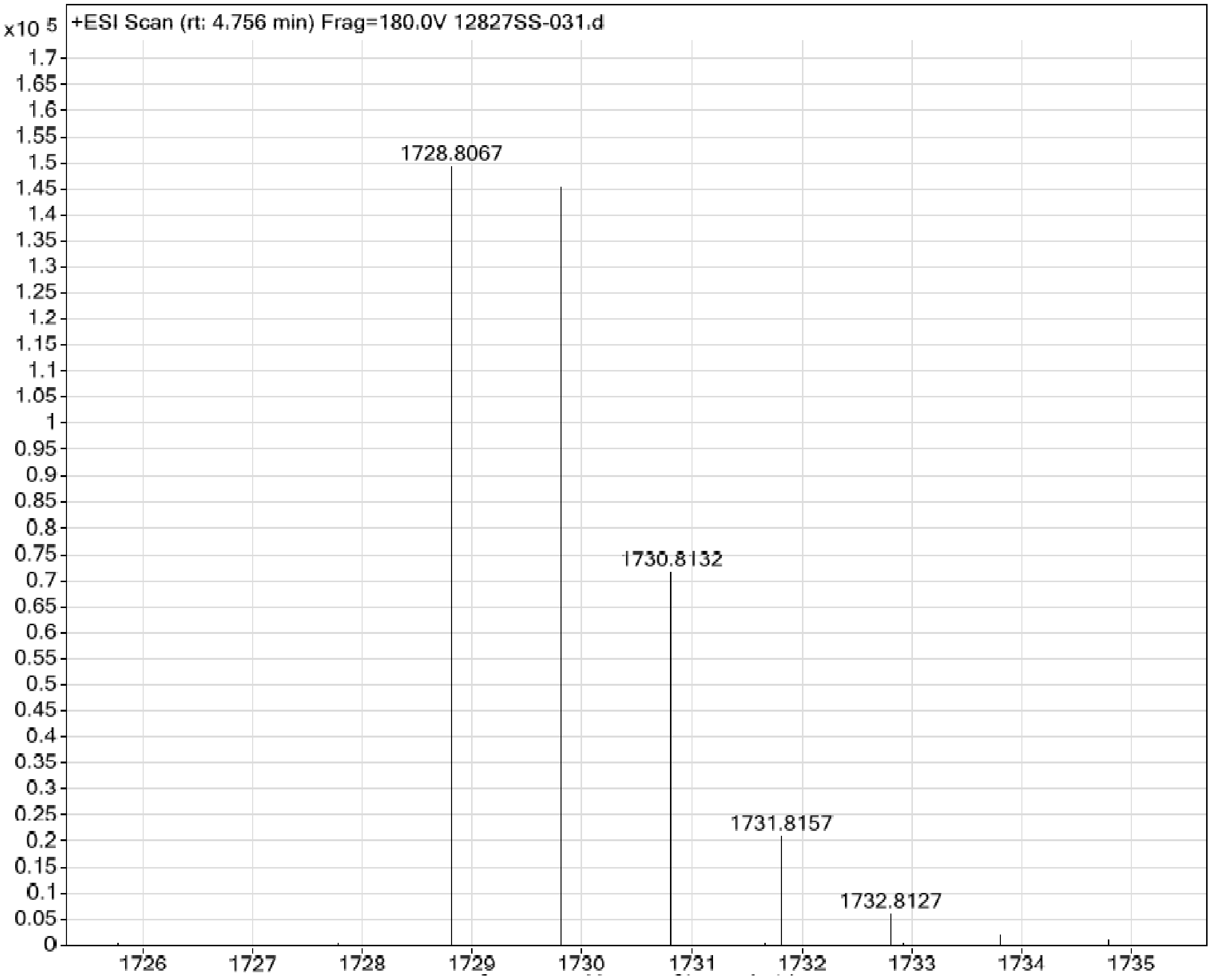

HRMS of 26-Dap

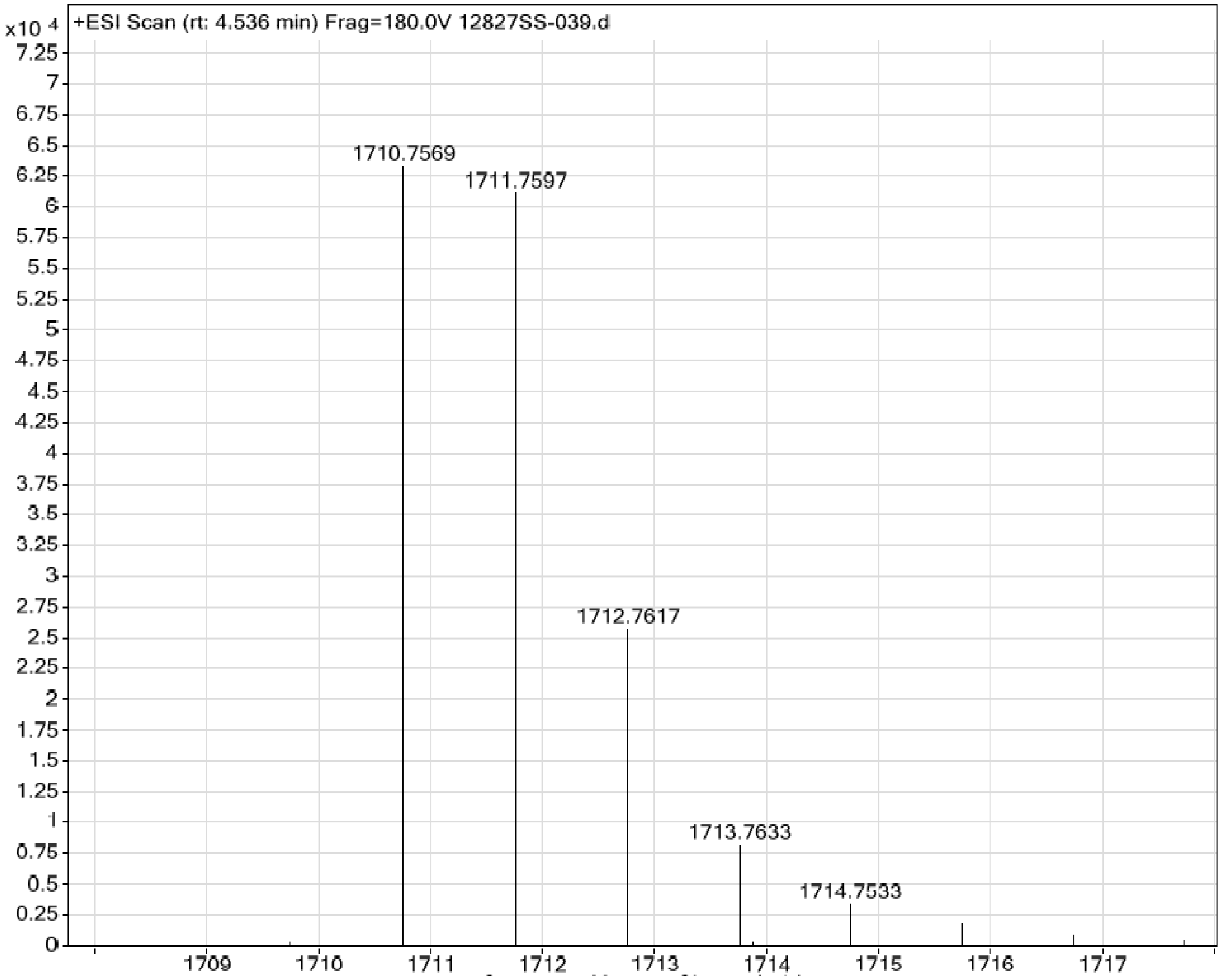

HRMS of 28-Dap

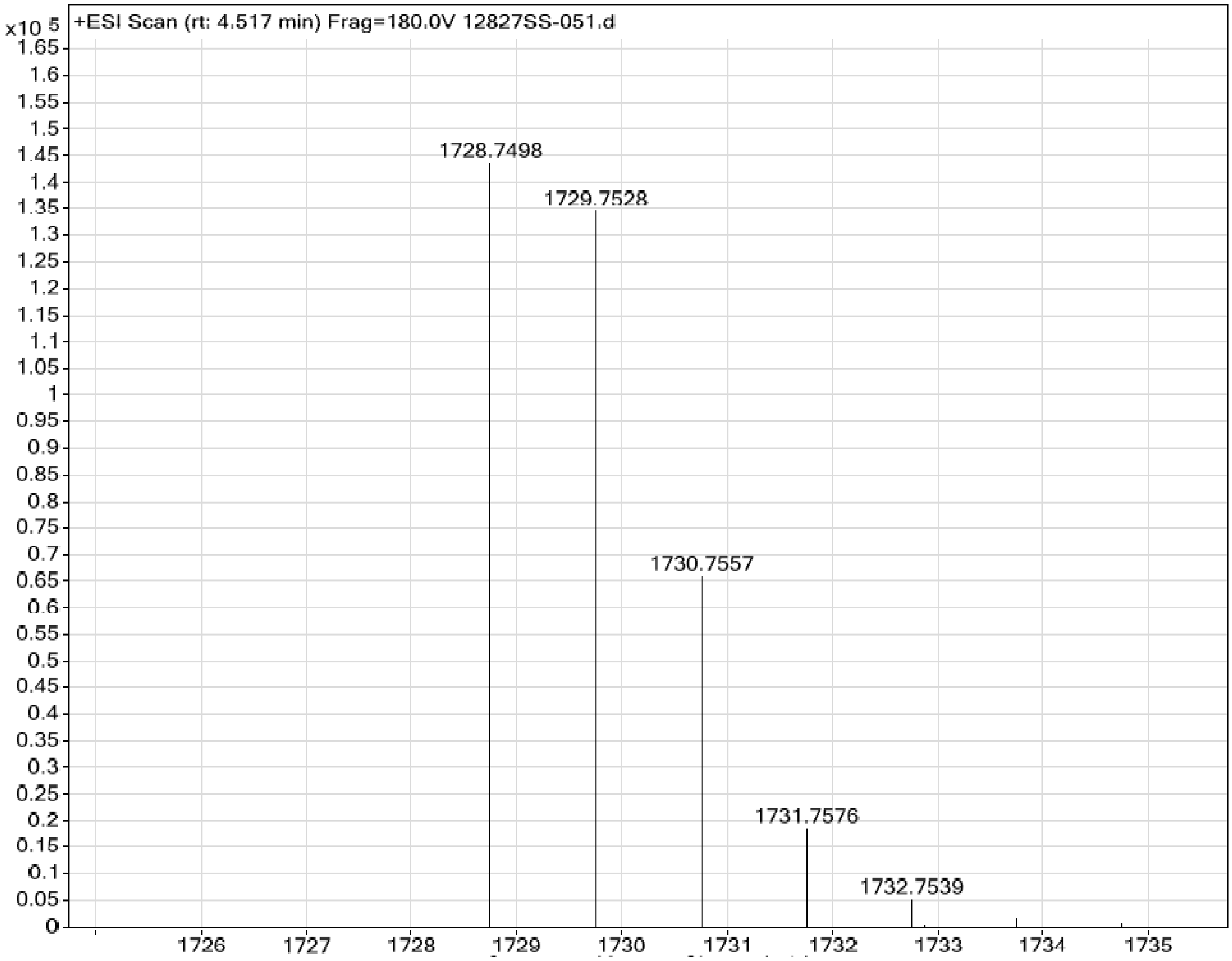

HRMS of 29-Dap

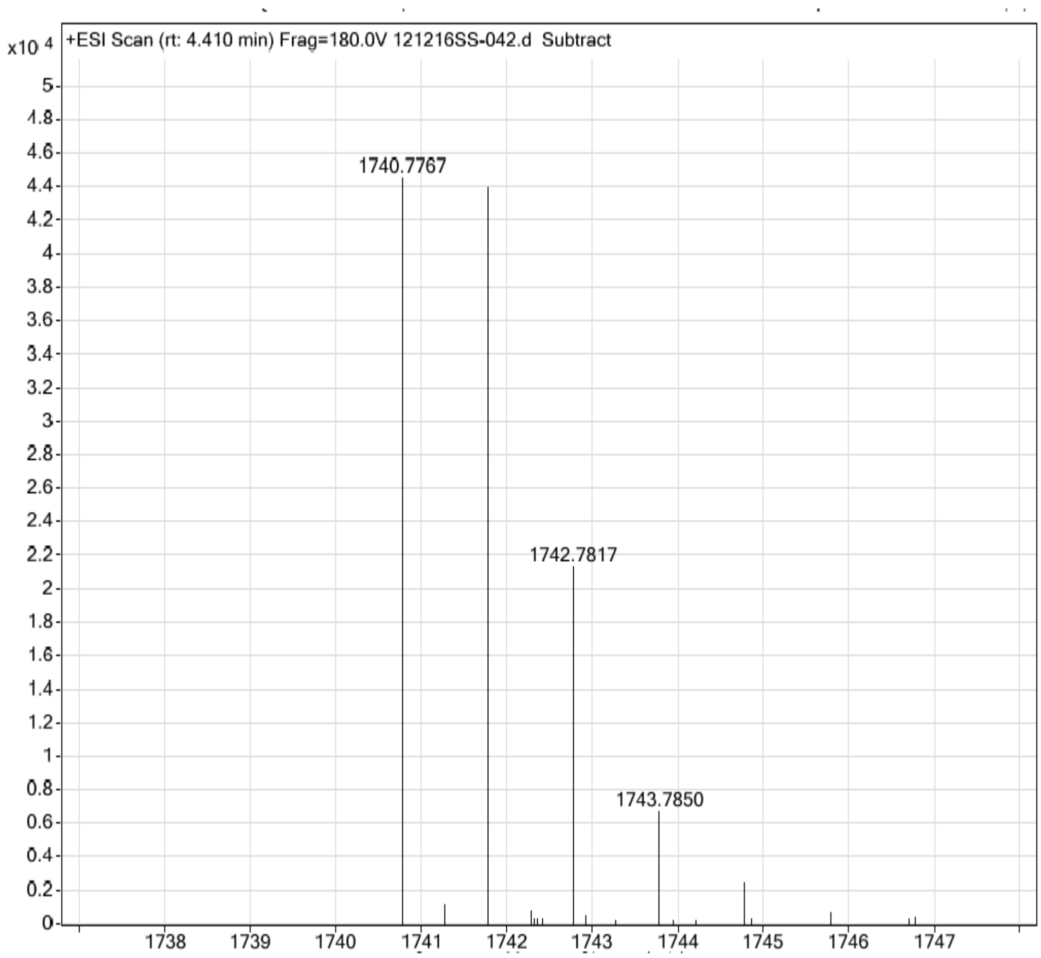

HRMS of 32-Dap (a)

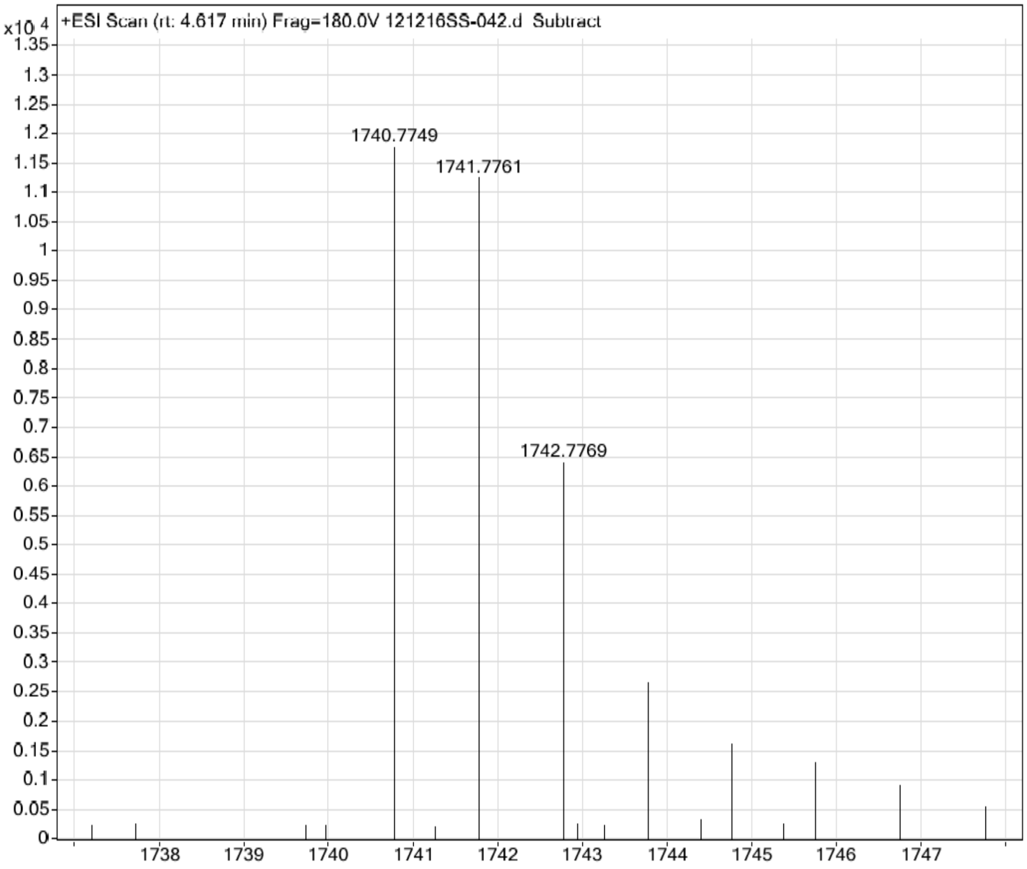

HRMS of 32-Dap (b)

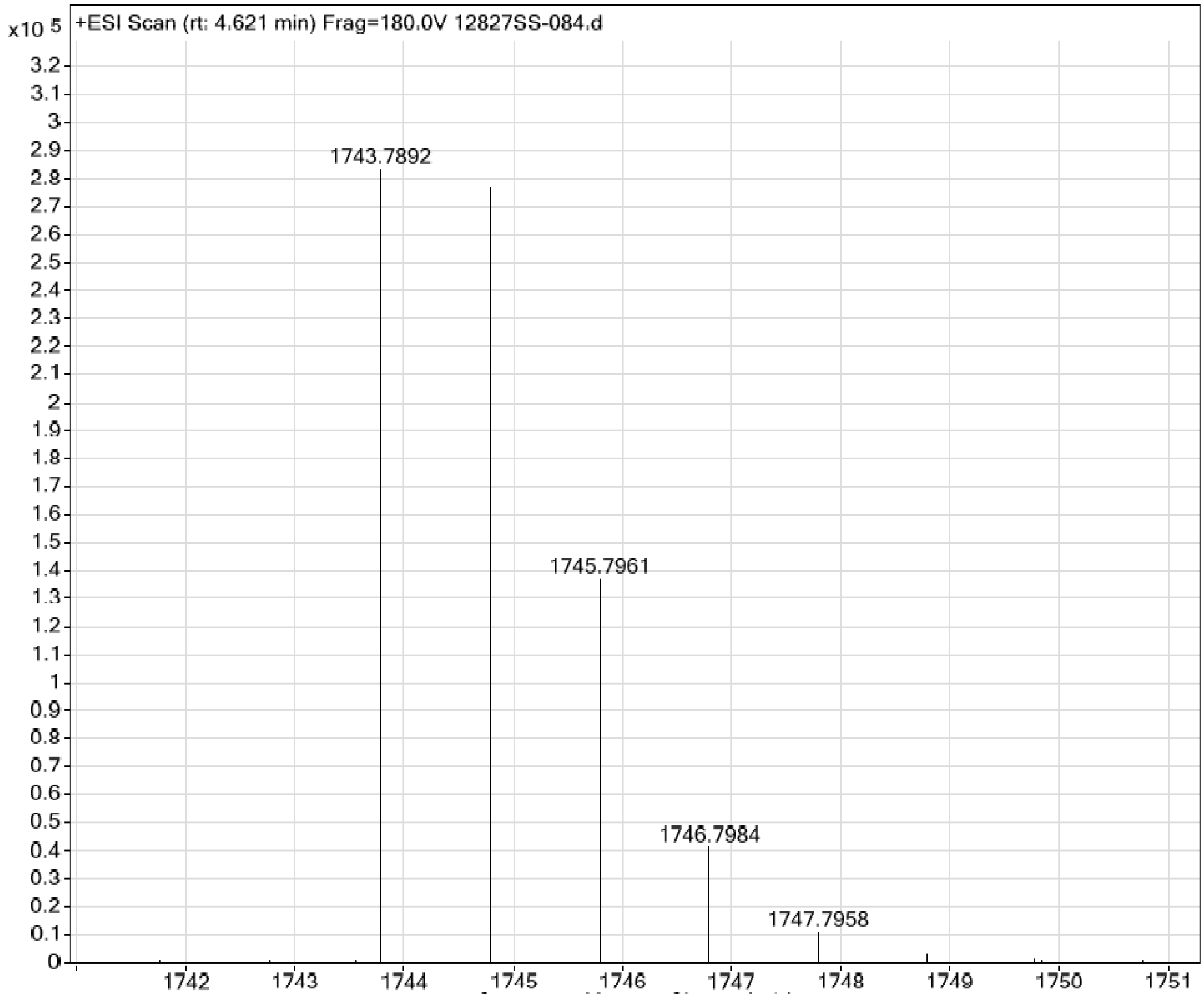

HRMS of 35-Dap

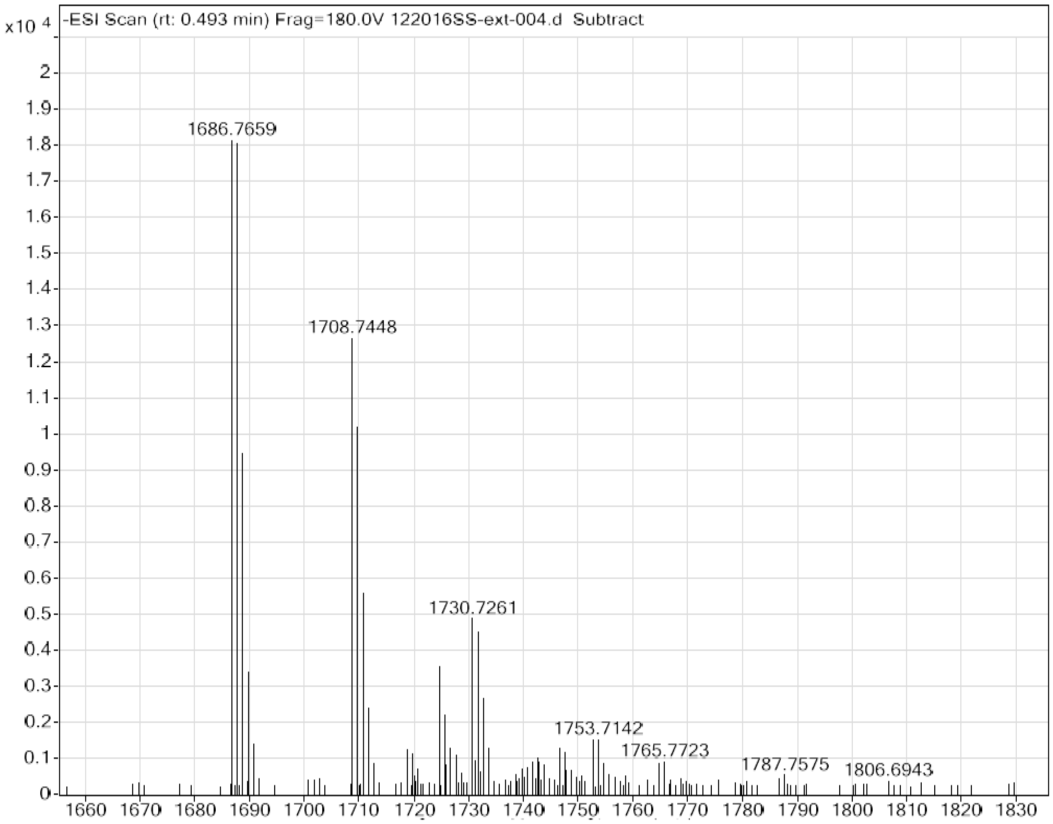
x10 4
-ESI Scan (rt: 0.493 min) Frag=180.0V 122016SS-ext-004.d Subtract
1686.7659
1708.7448
1730.7261
1753.7142
1765.7723
1787.7575
1806.6943
2
1.9
1.8
1.7
1.6
1.5
1.4
1.3
1.2
1.1
1
0.9
0.8
0.7
0.6
0.5
0.4
0.3
0.2
0.1
0
1660
1670
1680
1690
1700
1710
1720
1730
1740
1750
1760
1770
1780
1790
1800
1810
1820
1830

HRMS of 40

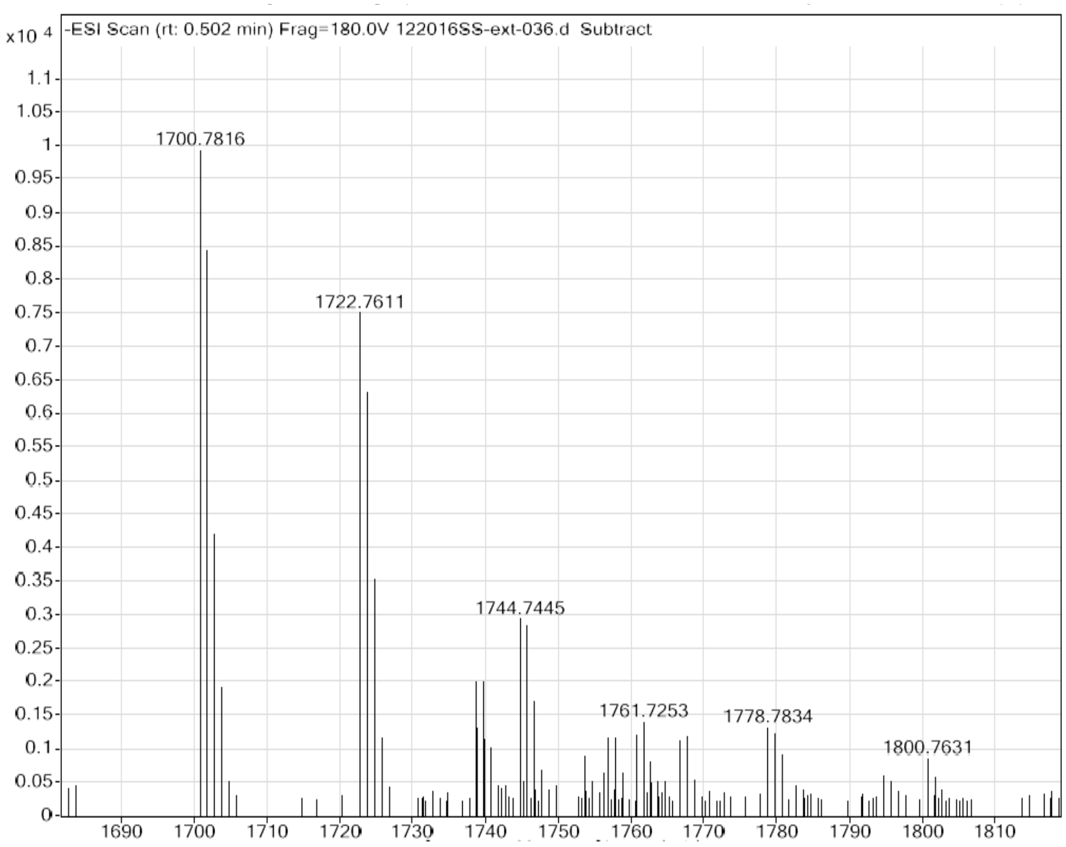
x10 4
-ESI Scan (rt: 0.502 min) Frag=180.0V 122016SS-ext-036.d Subtract
1700.7816
1722.7611
1744.7445
1761.7253
1778.7834
1800.7631

HRMS of 41

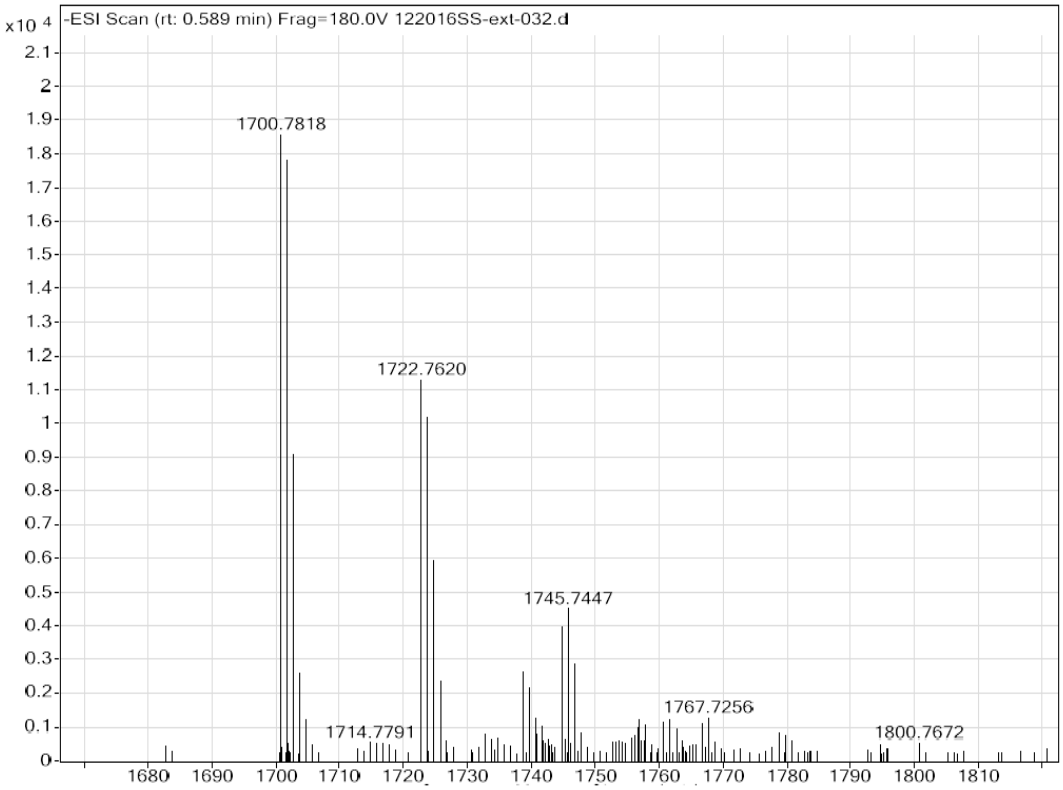
x10 4
-ESI Scan (rt: 0.589 min) Frag=180.0V 122016SS-ext-032.d
2.1
2
1.9
1.8
1.7
1.6
1.5
1.4
1.3
1.2
1.1
1
0.9
0.8
0.7
0.6
0.5
0.4
0.3
0.2
0.1
0
1700.7818
1722.7620
1745.7447
1714.7791
1767.7256
1800.7672
1680
1690
1700
1710
1720
1730
1740
1750
1760
1770
1780
1790
1800
1810

HRMS of 42

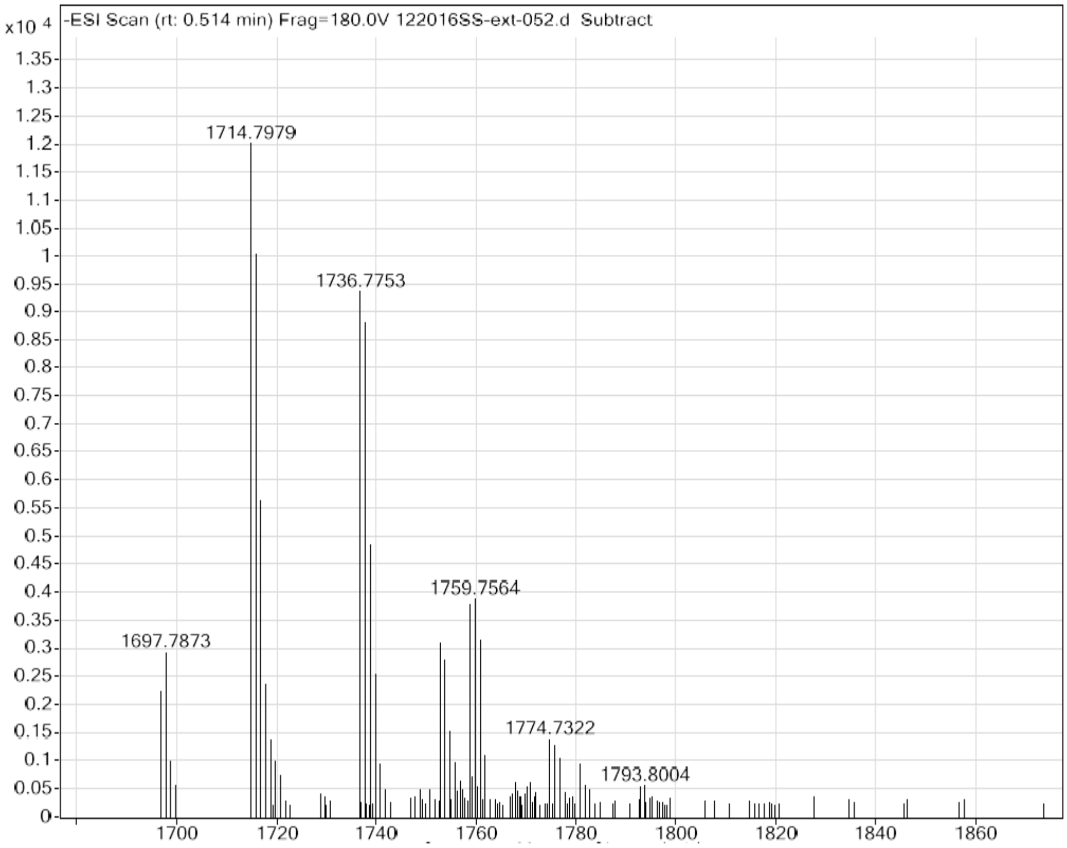
x10 4
-ESI Scan (rt: 0.514 min) Frag=180.0V 122016SS-ext-052.d Subtract
1714.7979
1736.7753
1759.7564
1697.7873
1774.7322
1793.8004
1.35
1.3
1.25
1.2
1.15
1.1
1.05
1
0.95
0.9
0.85
0.8
0.75
0.7
0.65
0.6
0.55
0.5
0.45
0.4
0.35
0.3
0.25
0.2
0.15
0.1
0.05
0
1700
1720
1740
1760
1780
1800
1820
1840
1860

HRMS of 43

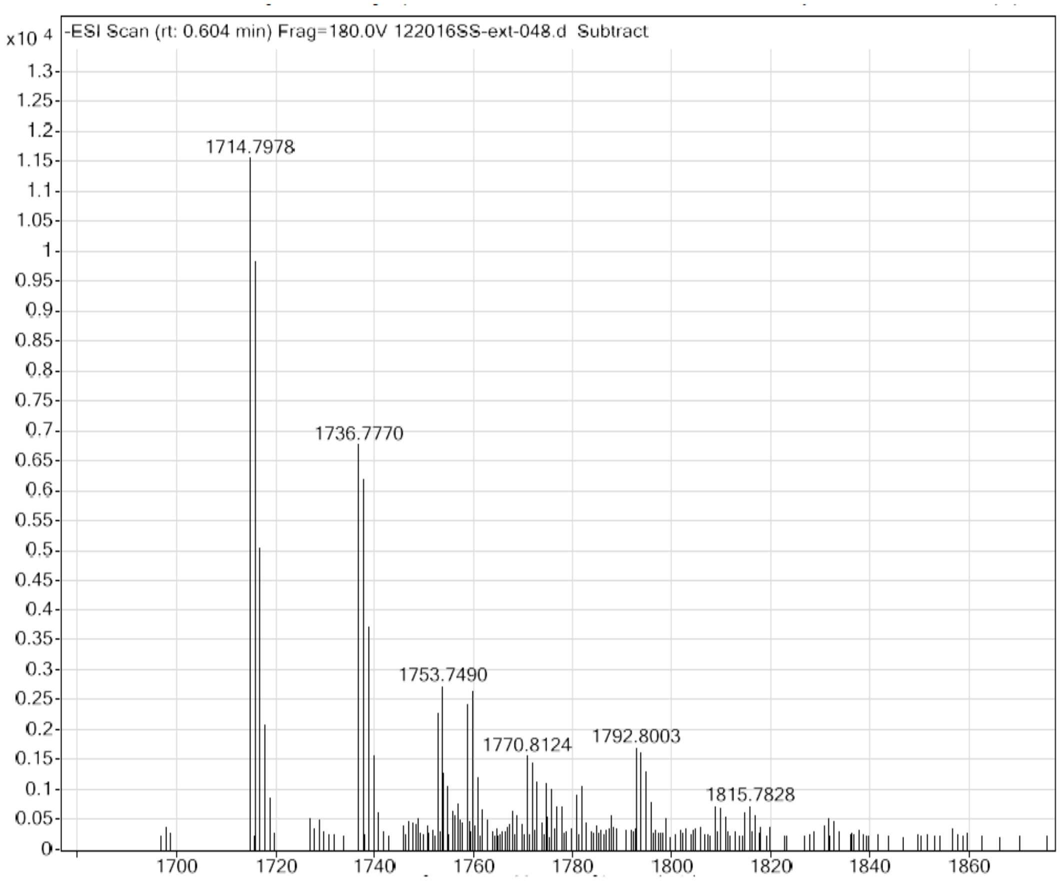

x10 4
-ESI Scan (rt: 0.604 min) Frag=180.0V 122016SS-ext-048.d Subtract
1714.7978
1736.7770
1753.7490
1770.8124
1792.8003
1815.7828

HRMS of 44

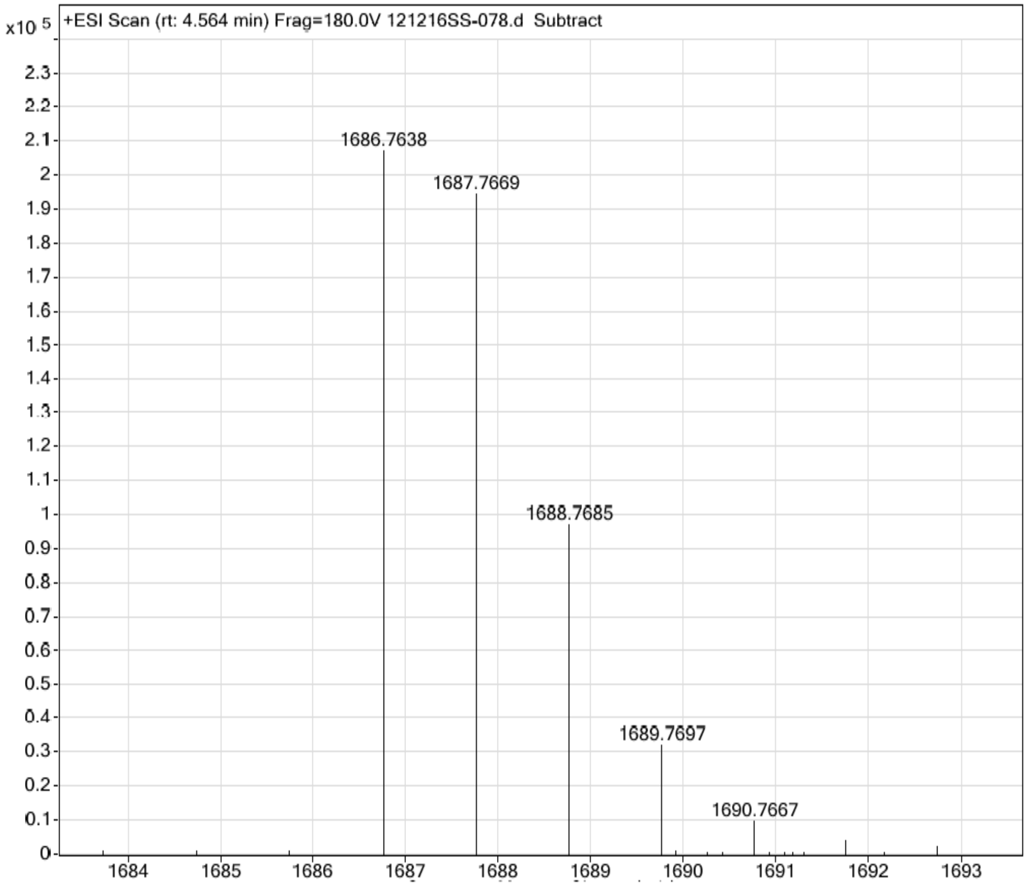
x10 5
+ESI Scan (rt: 4.564 min) Frag=180.0V 121216SS-078.d Subtract
1686.7638
1687.7669
1688.7685
1689.7697
1690.7667
2.3
2.2
2.1
2
1.9
1.8
1.7
1.6
1.5
1.4
1.3
1.2
1.1
1
0.9
0.8
0.7
0.6
0.5
0.4
0.3
0.2
0.1
0
1684
1685
1686
1687
1688
1689
1690
1691
1692
1693

HRMS of 45

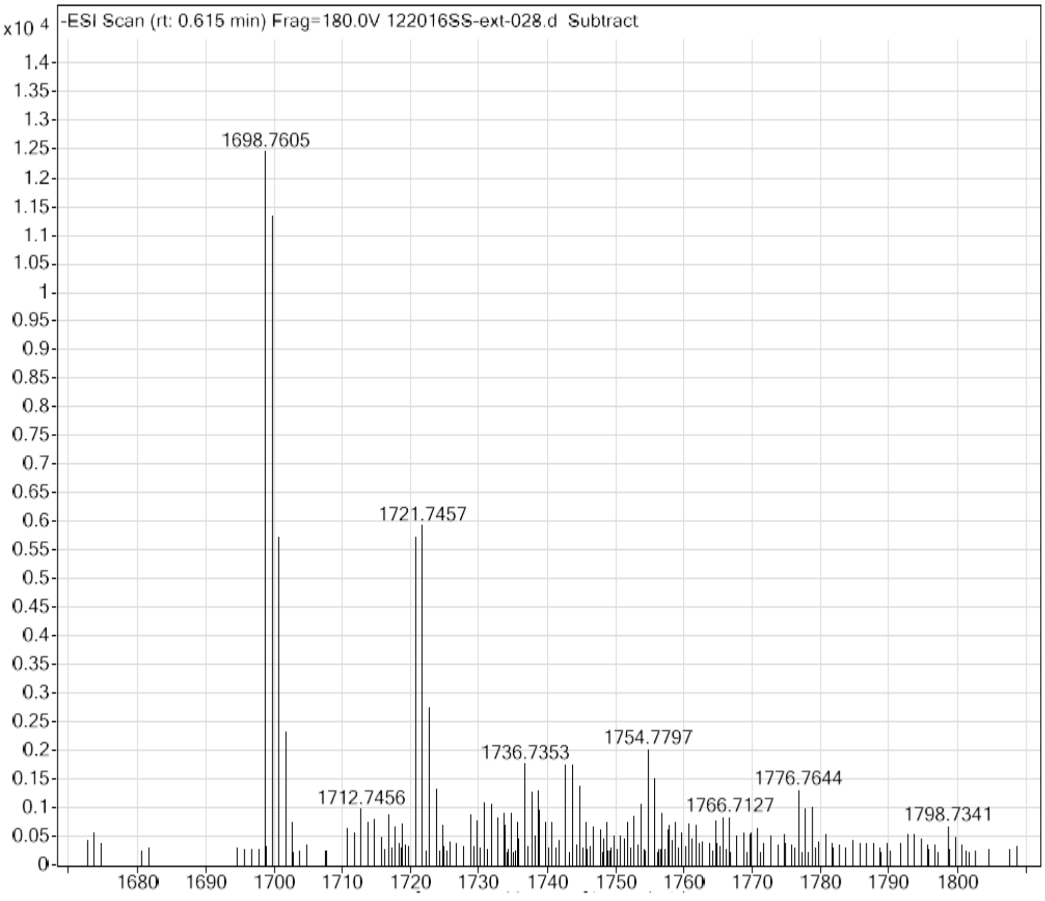

x10 4
-ESI Scan (rt: 0.615 min) Frag=180.0V 122016SS-ext-028.d Subtract
1698.7605
1721.7457
1712.7456
1736.7353
1754.7797
1766.7127
1776.7644
1798.7341

HRMS of 46

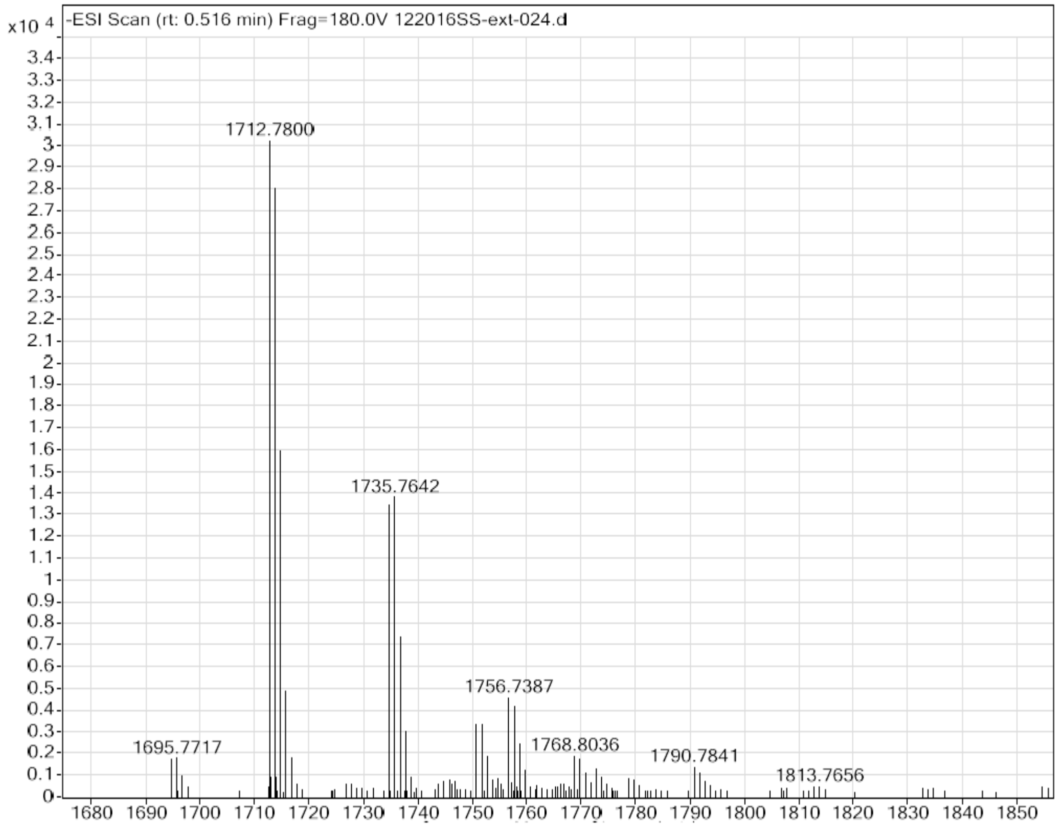
-ESI Scan (rt: 0.516 min) Frag=180.0V 122016SS-ext-024.d
x10 4
1712.7800
1735.7642
1756.7387
1768.8036
1790.7841
1813.7656
1695.7717

HRMS of 47

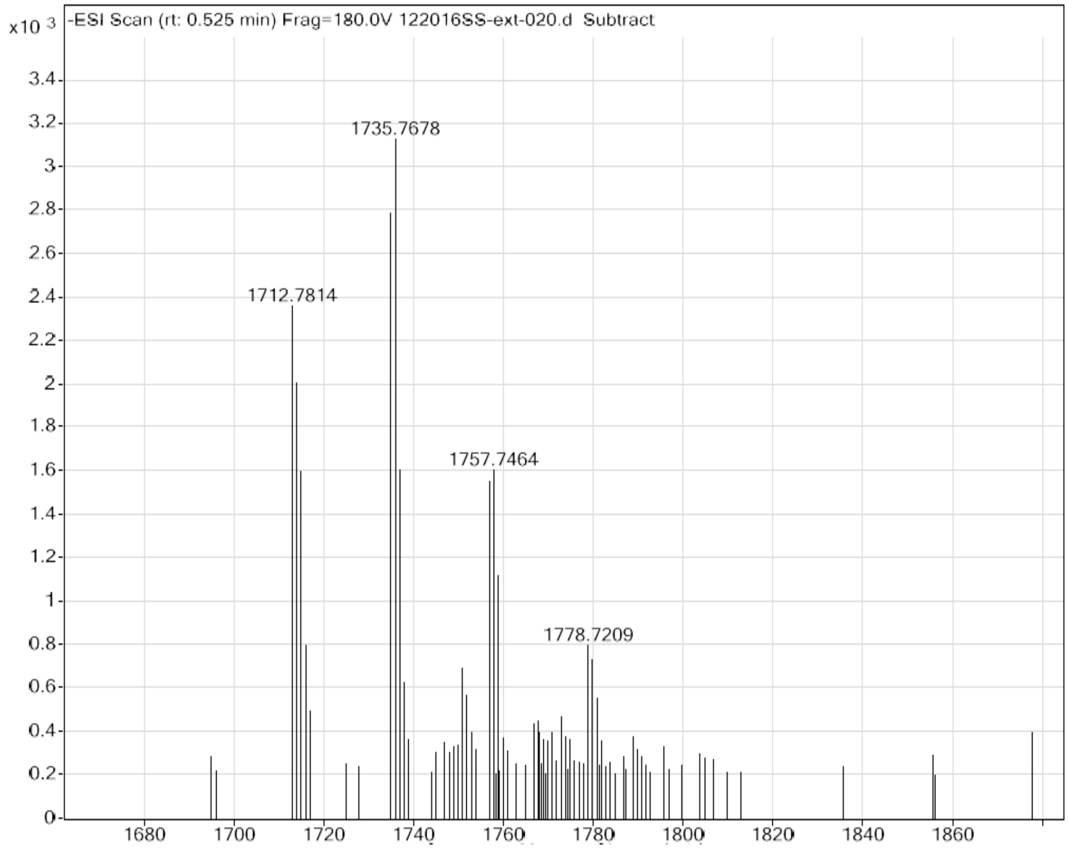
x10 3
-ESI Scan (rt: 0.525 min) Frag=180.0V 122016SS-ext-020.d Subtract
1735.7678
1712.7814
1757.7464
1778.7209
3.4
3.2
3
2.8
2.6
2.4
2.2
2
1.8
1.6
1.4
1.2
1
0.8
0.6
0.4
0.2
0
1680
1700
1720
1740
1760
1780
1800
1820
1840
1860

HRMS of 48

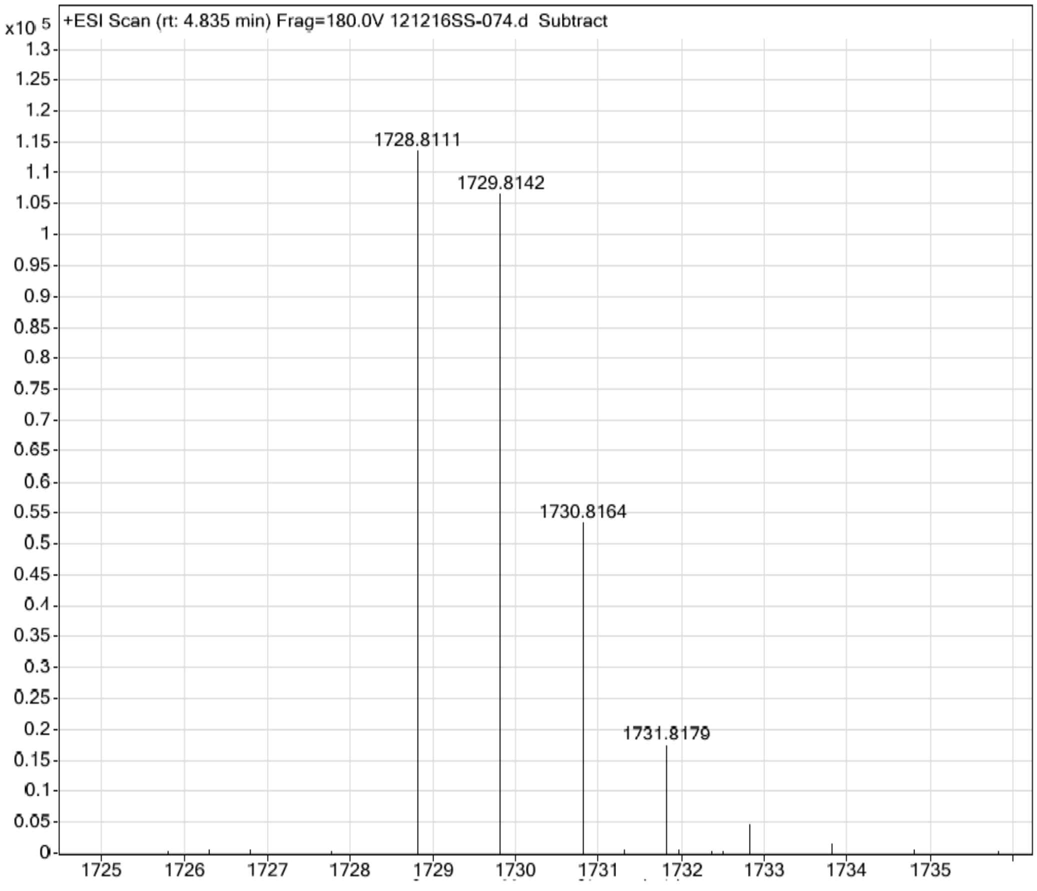

x10 5
+ESI Scan (rt: 4.835 min) Frag=180.0V 121216SS-074.d Subtract
1728.8111
1729.8142
1730.8164
1731.8179
1.3
1.25
1.2
1.15
1.1
1.05
1
0.95
0.9
0.85
0.8
0.75
0.7
0.65
0.6
0.55
0.5
0.45
0.4
0.35
0.3
0.25
0.2
0.15
0.1
0.05
0
1725
1726
1727
1728
1729
1730
1731
1732
1733
1734
1735

HRMS of 49

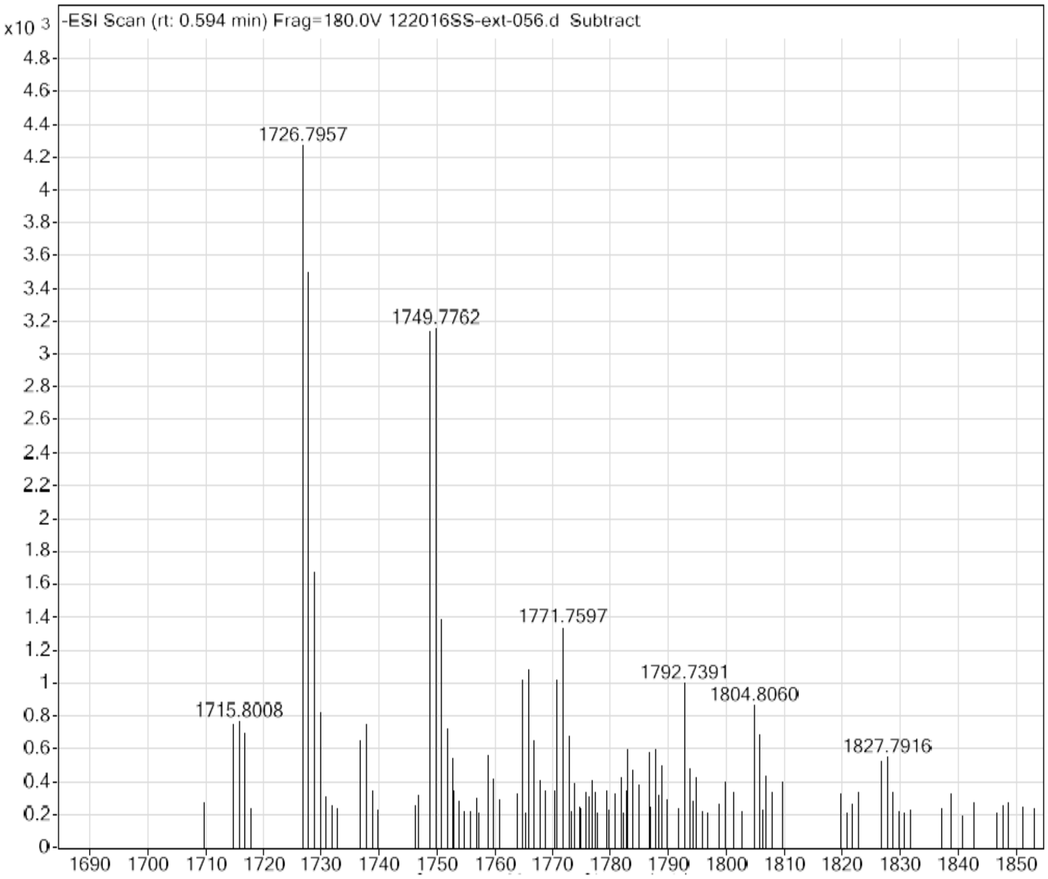
x10 3
-ESI Scan (rt: 0.594 min) Frag=180.0V 122016SS-ext-056.d Subtract
1726.7957
1749.7762
1771.7597
1792.7391
1804.8060
1715.8008
1827.7916

HRMS of 50

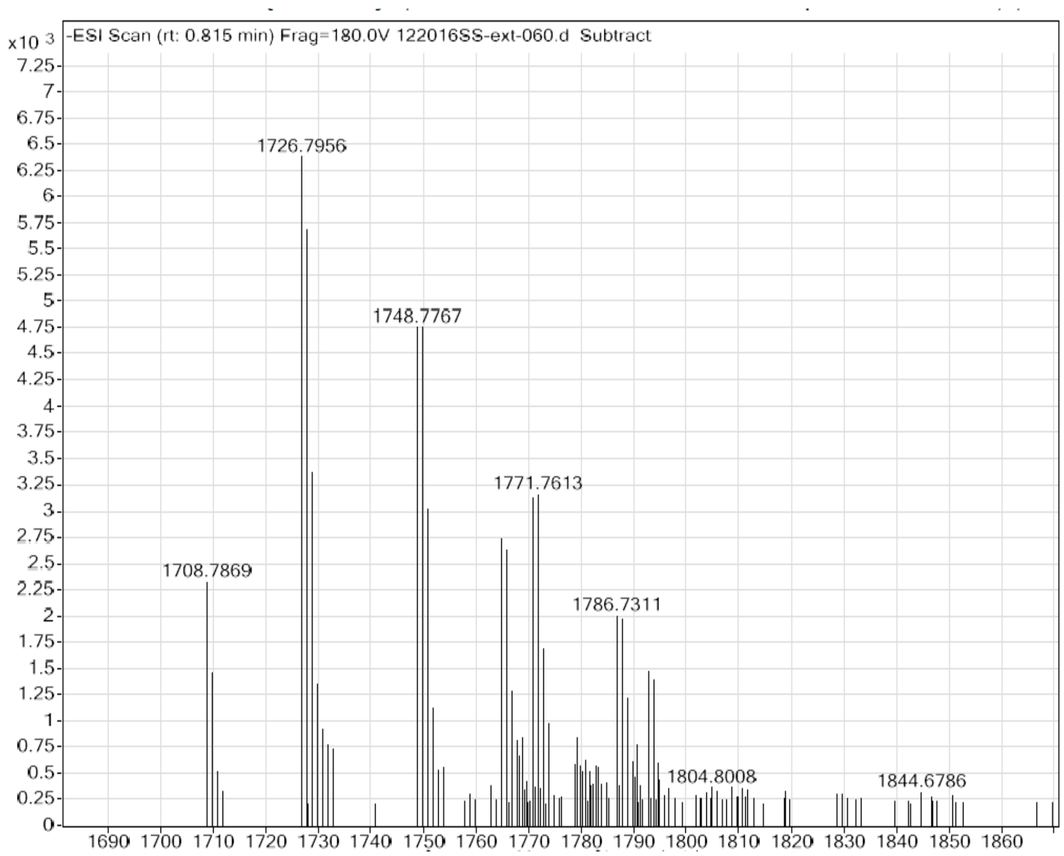
x10 3
-ESI Scan (rt: 0.815 min) Frag=180.0V 122016SS-ext-060.d Subtract
1726.7956
1748.7767
1771.7613
1708.7869
1786.7311
1804.8008
1844.6786
7.25
7
6.75
6.5
6.25
6
5.75
5.5
5.25
5
4.75
4.5
4.25
4
3.75
3.5
3.25
3
2.75
2.5
2.25
2
1.75
1.5
1.25
1
0.75
0.5
0.25
0
1690 1700 1710 1720 1730 1740 1750 1760 1770 1780 1790 1800 1810 1820 1830 1840 1850 1860

HRMS of 51

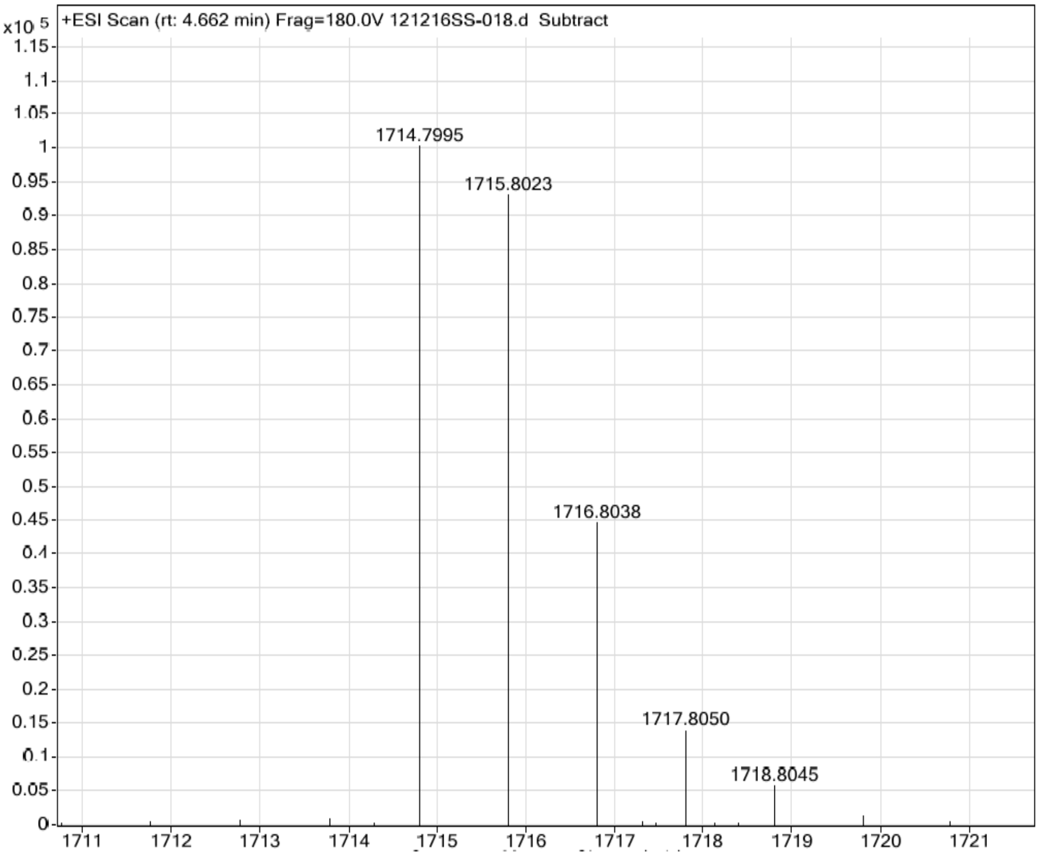

x10 5
+ESI Scan (rt: 4.662 min) Frag=180.0V 121216SS-018.d Subtract
1.15
1.1
1.05
1
0.95
0.9
0.85
0.8
0.75
0.7
0.65
0.6
0.55
0.5
0.45
0.4
0.35
0.3
0.25
0.2
0.15
0.1
0.05
0
1714.7995
1715.8023
1716.8038
1717.8050
1718.8045
1711
1712
1713
1714
1715
1716
1717
1718
1719
1720
1721

HRMS of 52

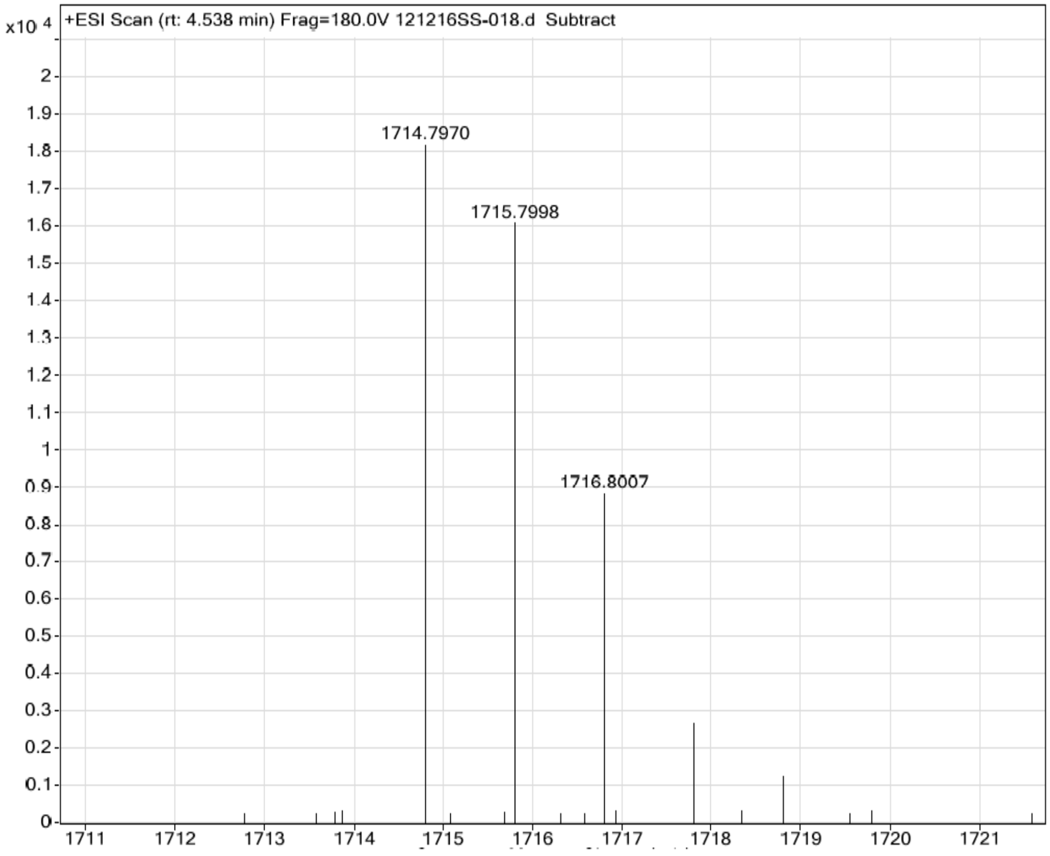
x10 4
+ESI Scan (rt: 4.538 min) Frag=180.0V 121216SS-018.d Subtract
2
1.9
1.8
1.7
1.6
1.5
1.4
1.3
1.2
1.1
1
0.9
0.8
0.7
0.6
0.5
0.4
0.3
0.2
0.1
0
1714.7970
1715.7998
1716.8007
1711
1712
1713
1714
1715
1716
1717
1718
1719
1720
1721

HRMS of 53

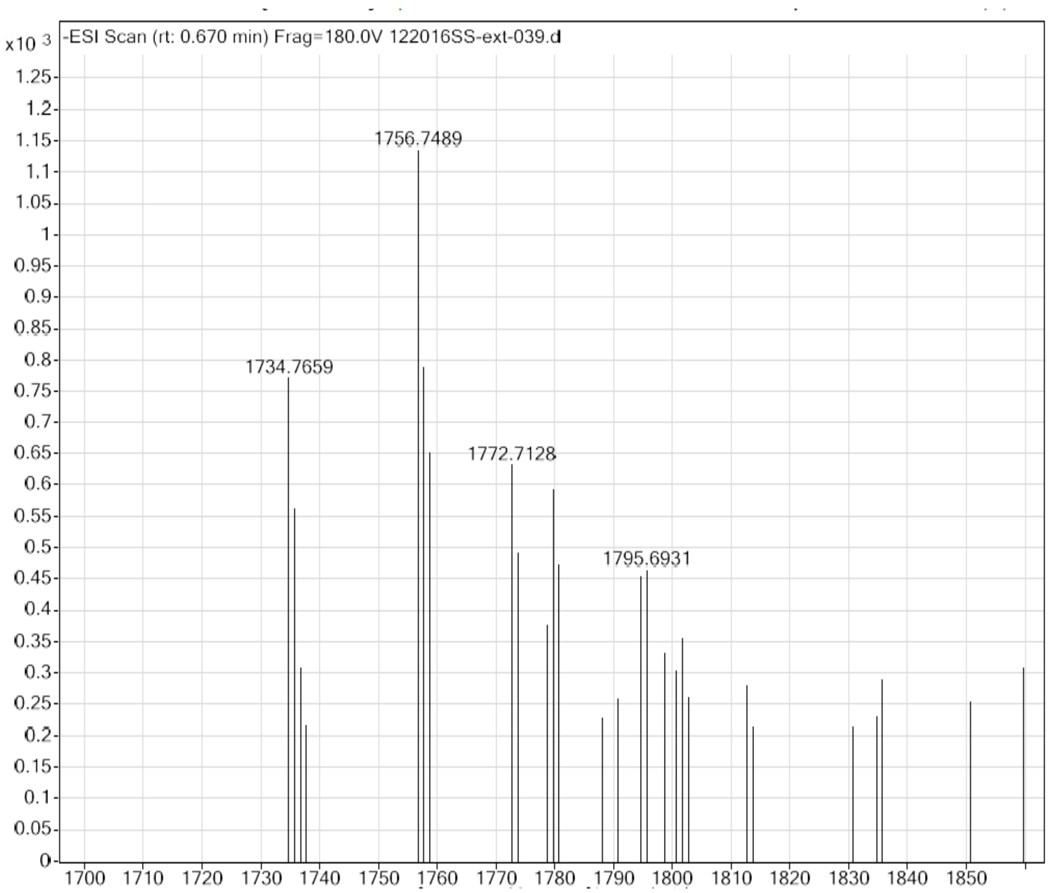
-ESI Scan (rt: 0.670 min) Frag=180.0V 122016SS-ext-039.d
x10 3
1756.7489
1734.7659
1772.7128
1795.6931
1700
1710
1720
1730
1740
1750
1760
1770
1780
1790
1800
1810
1820
1830
1840
1850

HRMS of 54

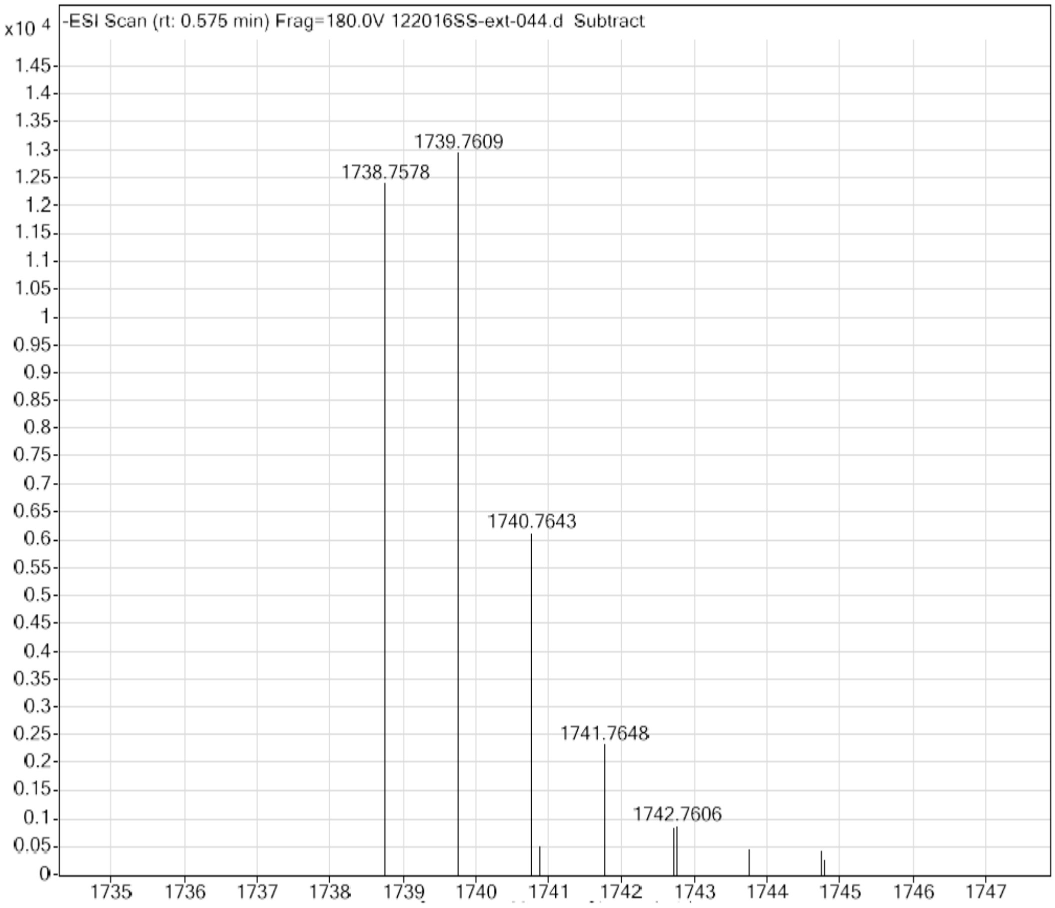
x10 4
-ESI Scan (rt: 0.575 min) Frag=180.0V 122016SS-ext-044.d Subtract
1739.7609
1738.7578
1740.7643
1741.7648
1742.7606
1.45
1.4
1.35
1.3
1.25
1.2
1.15
1.1
1.05
1
0.95
0.9
0.85
0.8
0.75
0.7
0.65
0.6
0.55
0.5
0.45
0.4
0.35
0.3
0.25
0.2
0.15
0.1
0.05
0
1735
1736
1737
1738
1739
1740
1741
1742
1743
1744
1745
1746
1747

HRMS of 55

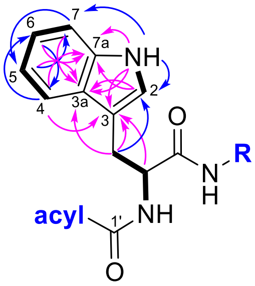

Dap

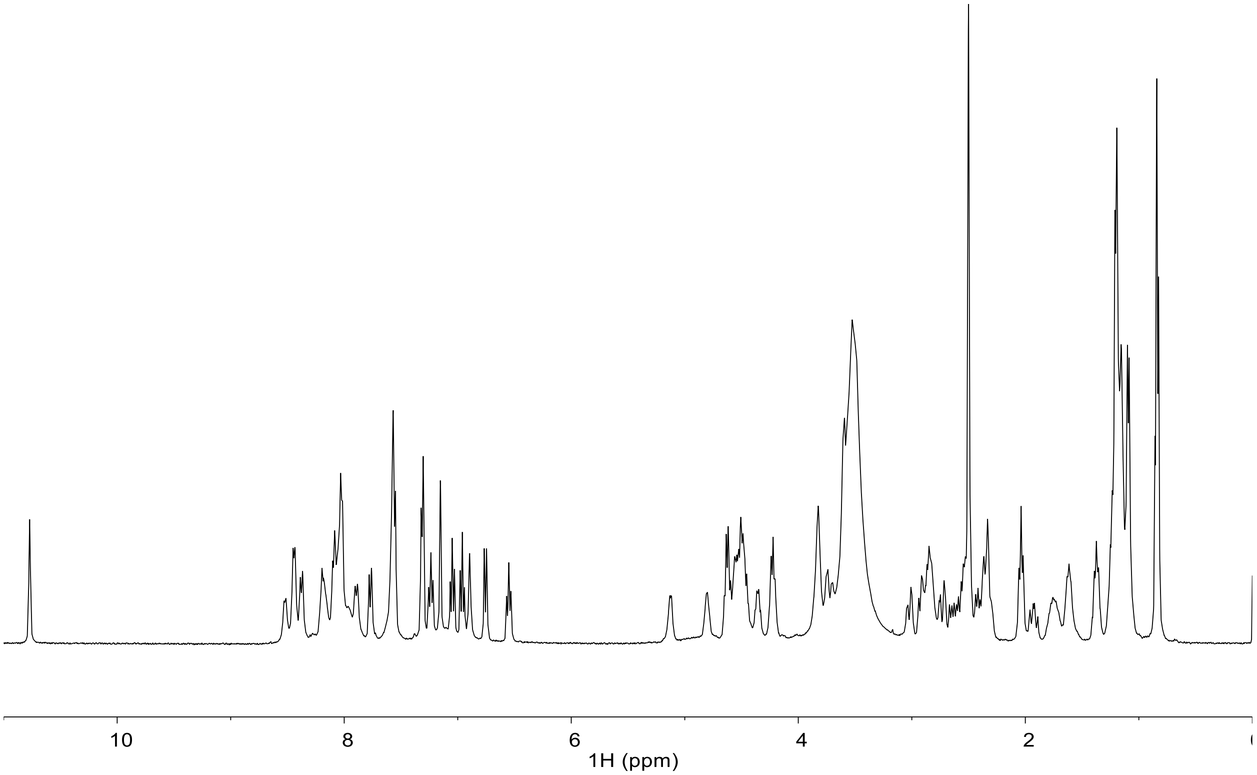

1D ^{1}H NMR spectrum (d_6-DMSO, 600 MHz of ^{1}H) of **Dap**

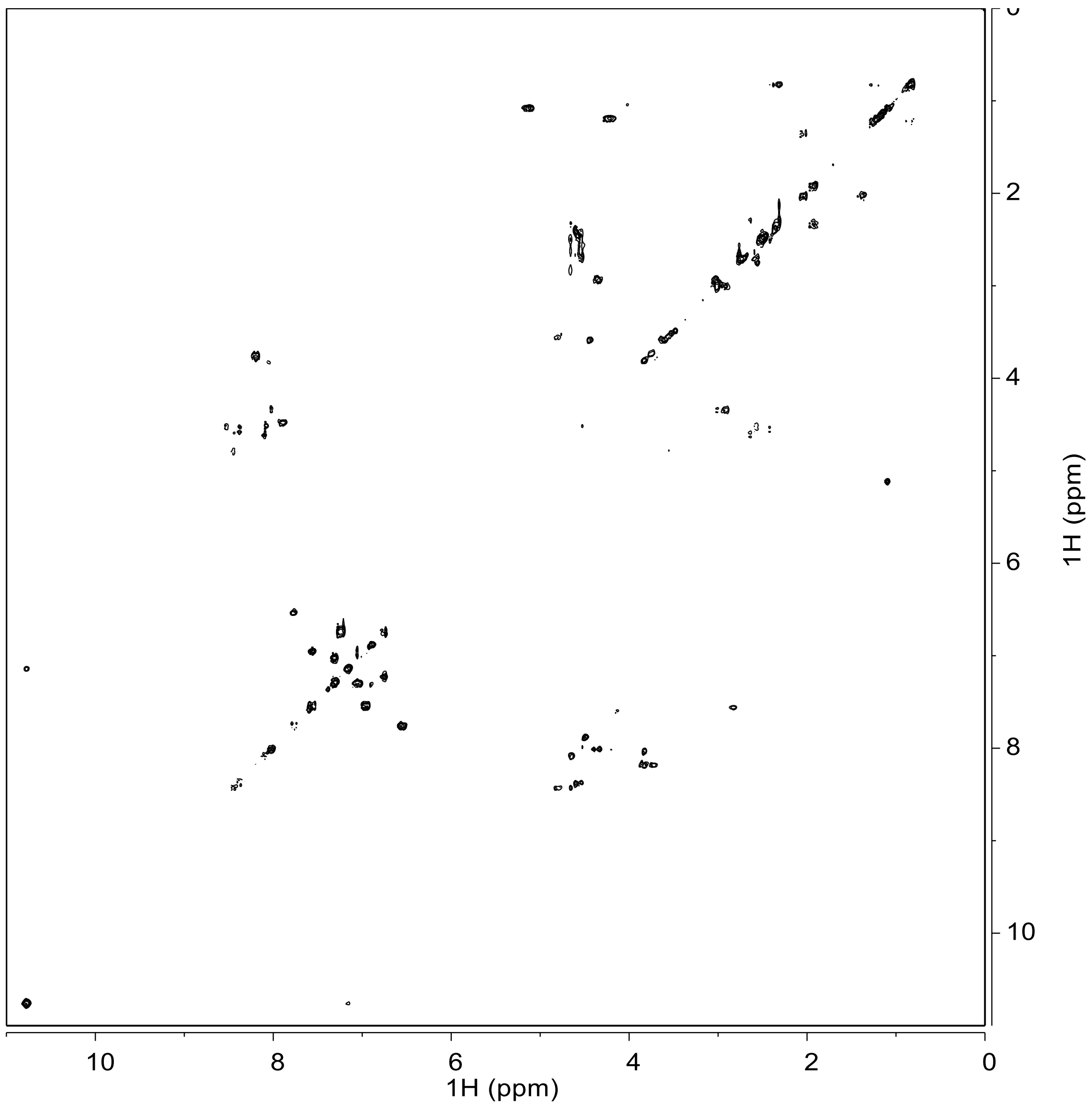

2D ^1H-^1H COSY NMR spectrum (d_6-DMSO, 600 MHz of ^1H) of **Dap**

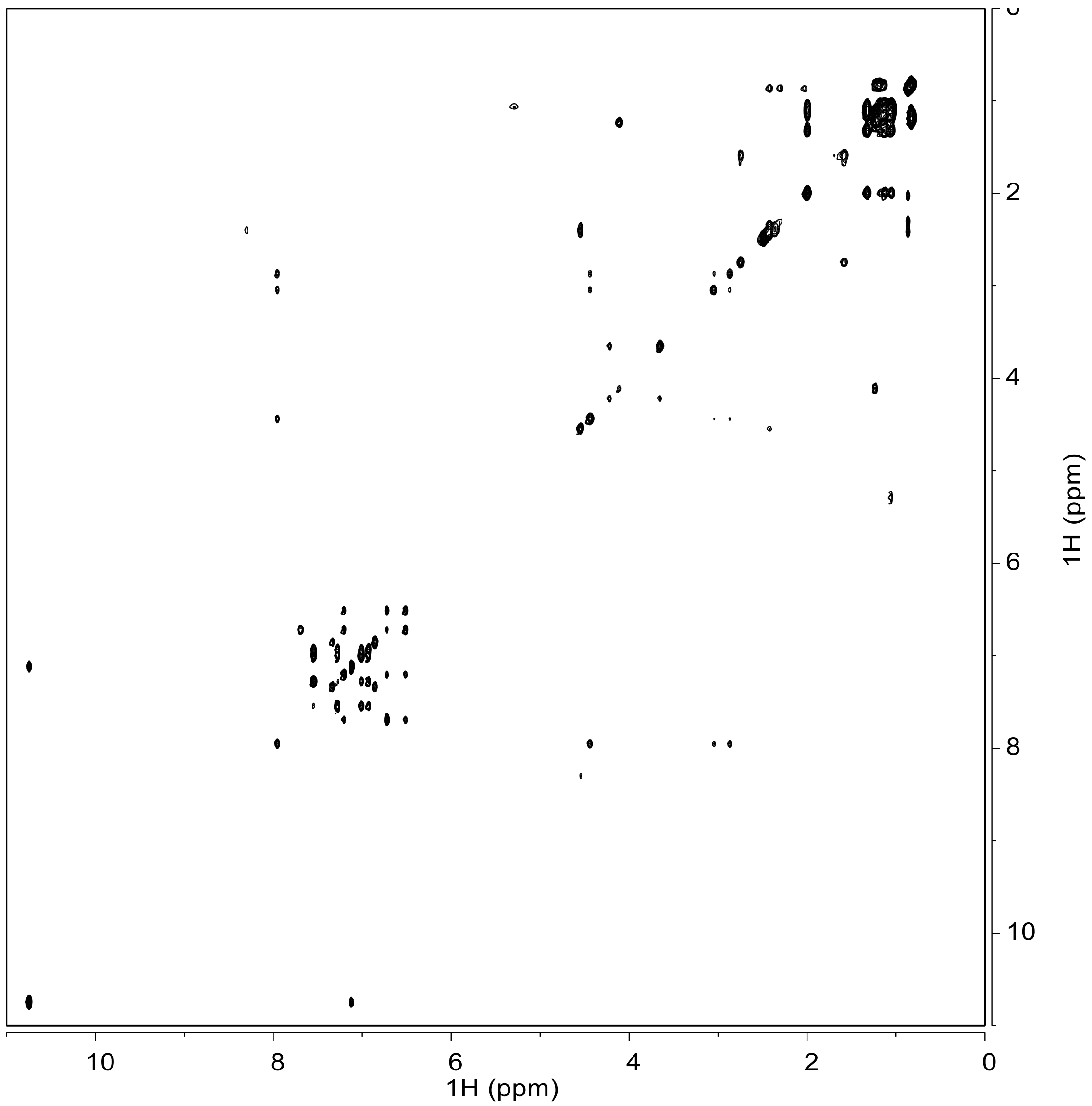

2D ^1H-^1H TOCSY NMR spectrum (d_6-DMSO, 600 MHz of ^1H) of **Dap**

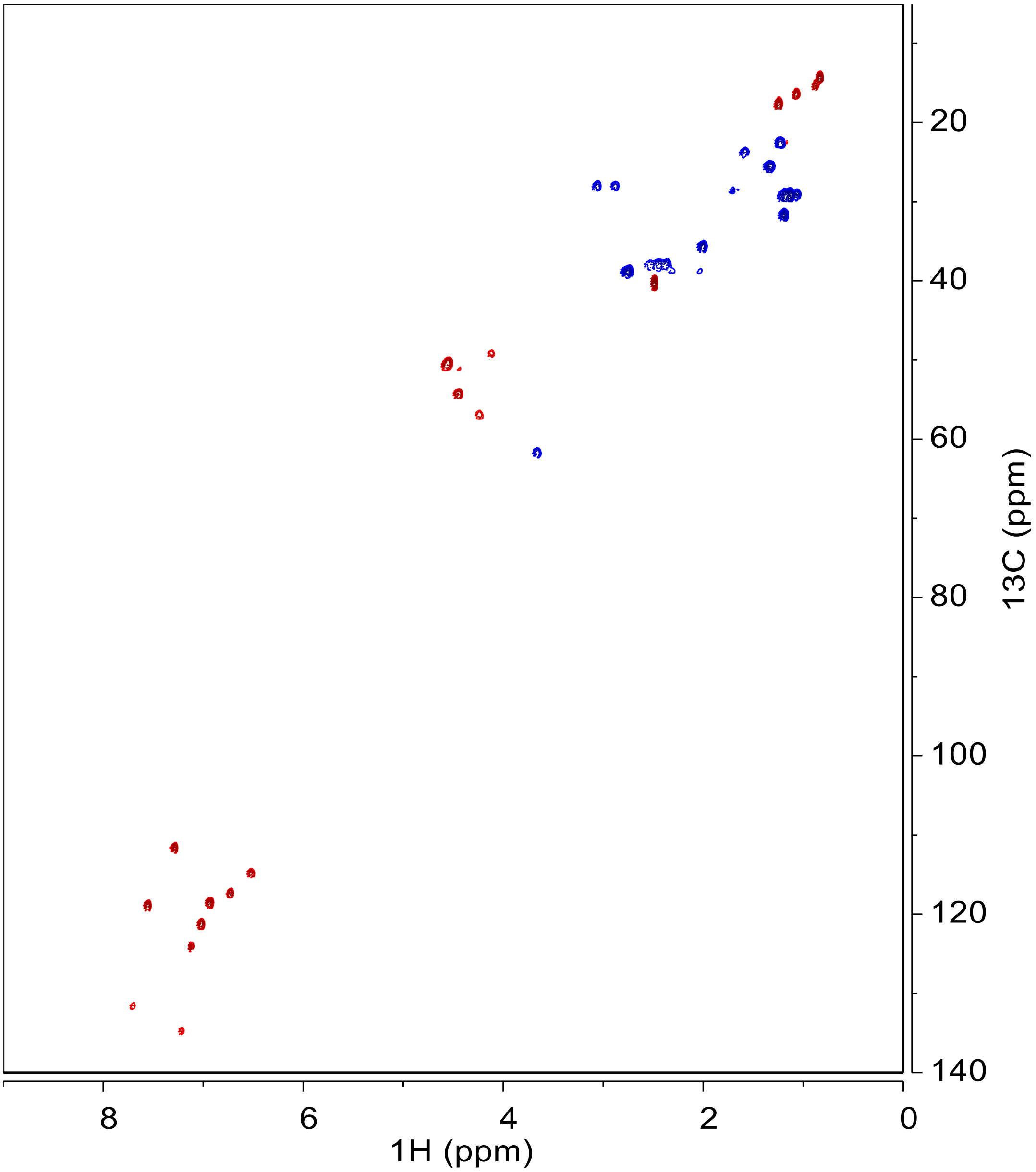

2D ^{1}H-^{13}C HSQC NMR spectrum (d_6-DMSO, 600 MHz of ^{1}H) of **Dap**

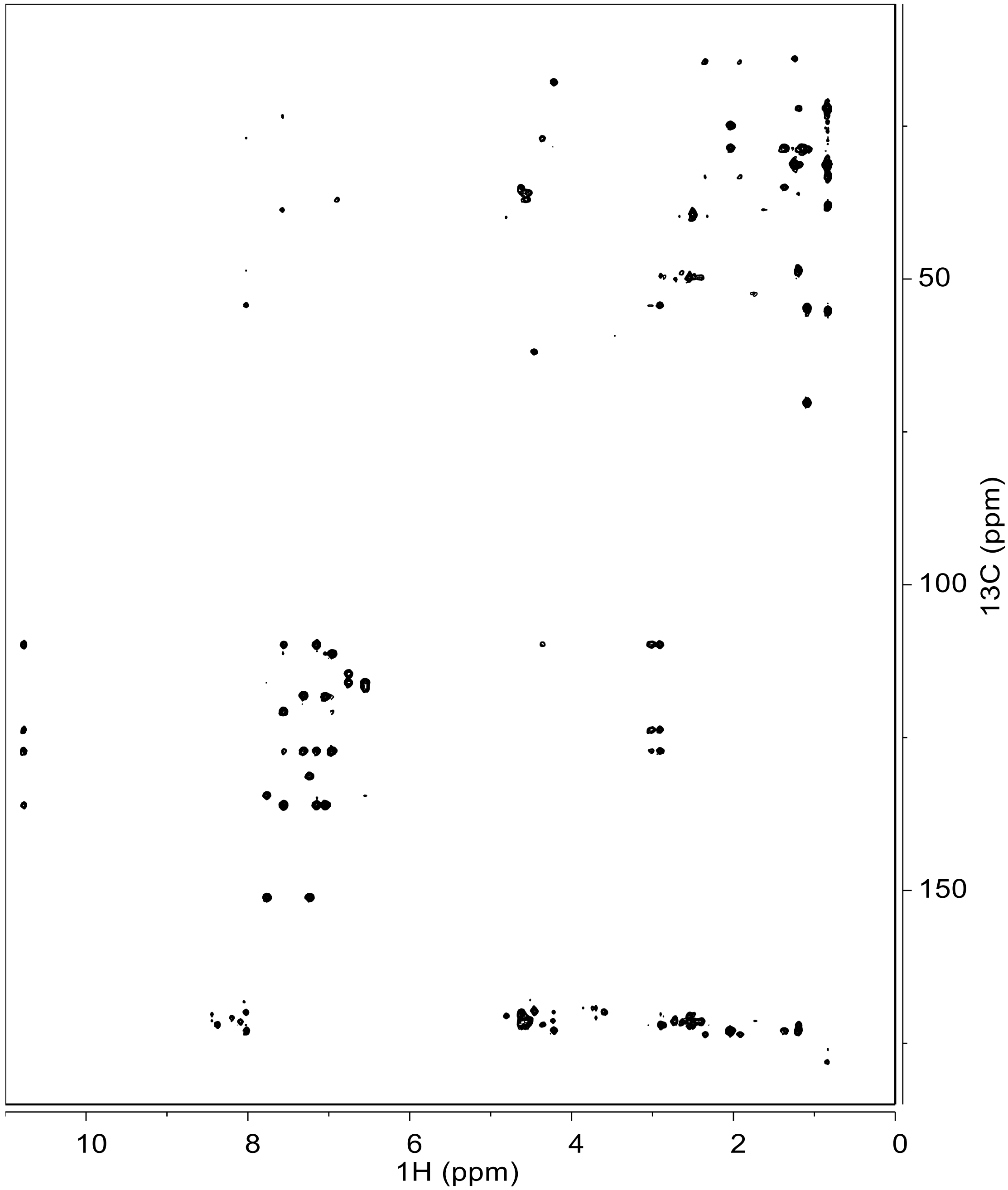

2D ^{1}H-^{13}C HMBC NMR spectrum (d_6-DMSO, 600 MHz of ^{1}H) of **Dap**

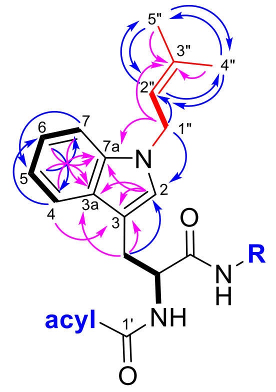

***N1*-5-Dap (40)**

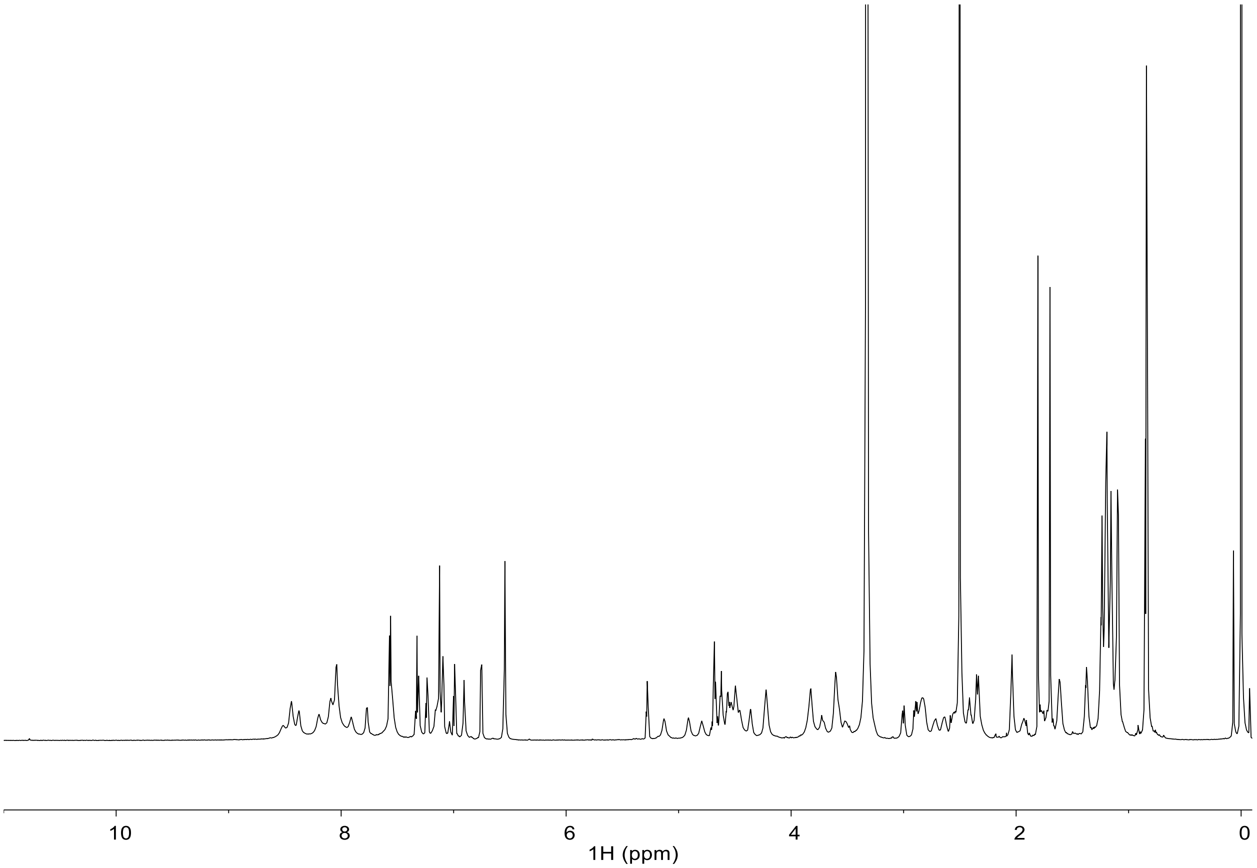

1D ^{1}H NMR spectrum (d_6-DMSO, 600 MHz of ^{1}H) of ***N1*-5-Dap (40)**

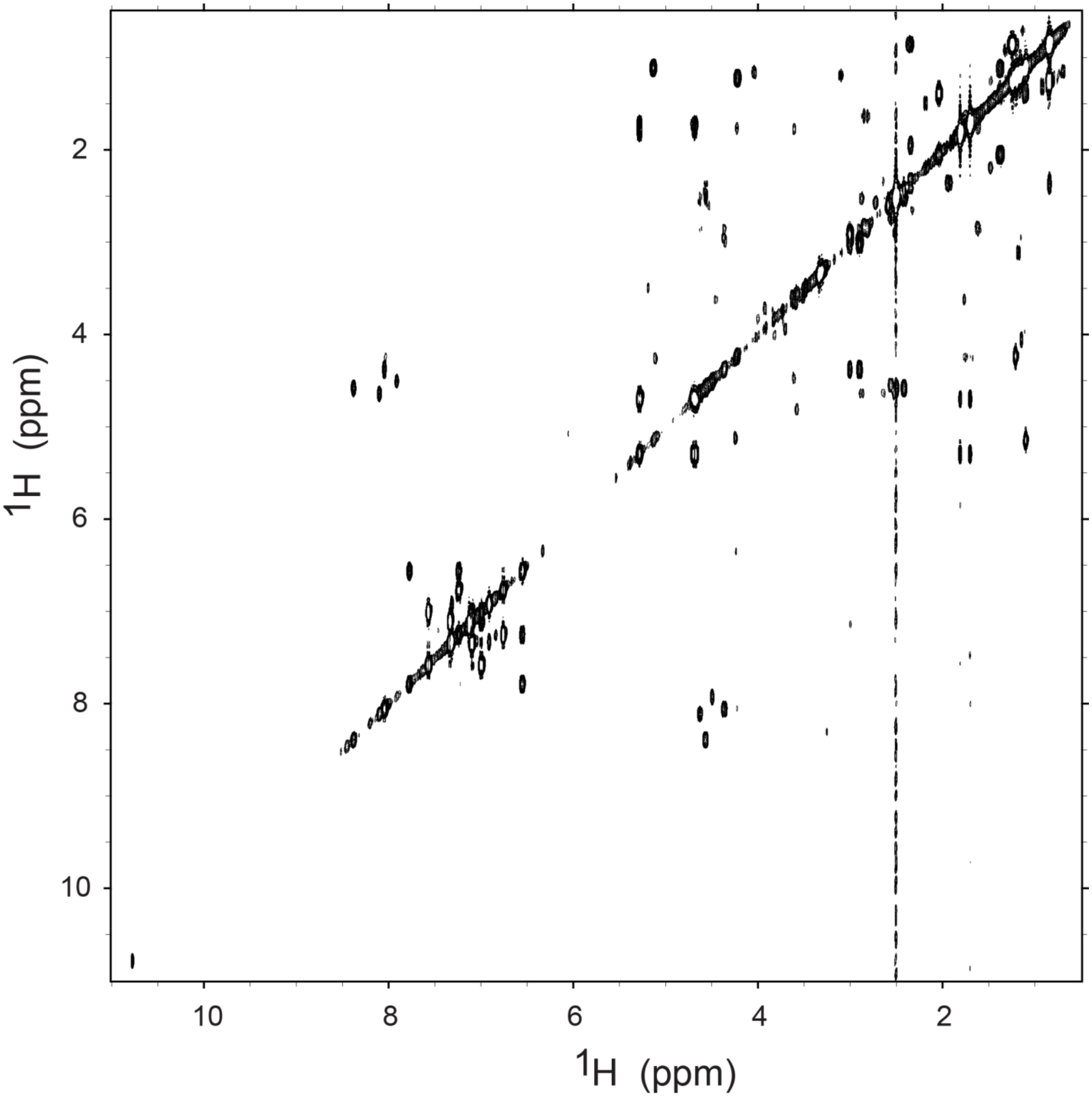

2D ^{1}H-^{1}H COSY NMR spectrum (d_6-DMSO, 600 MHz of ^{1}H) of ***N1*-5-Dap (40)**

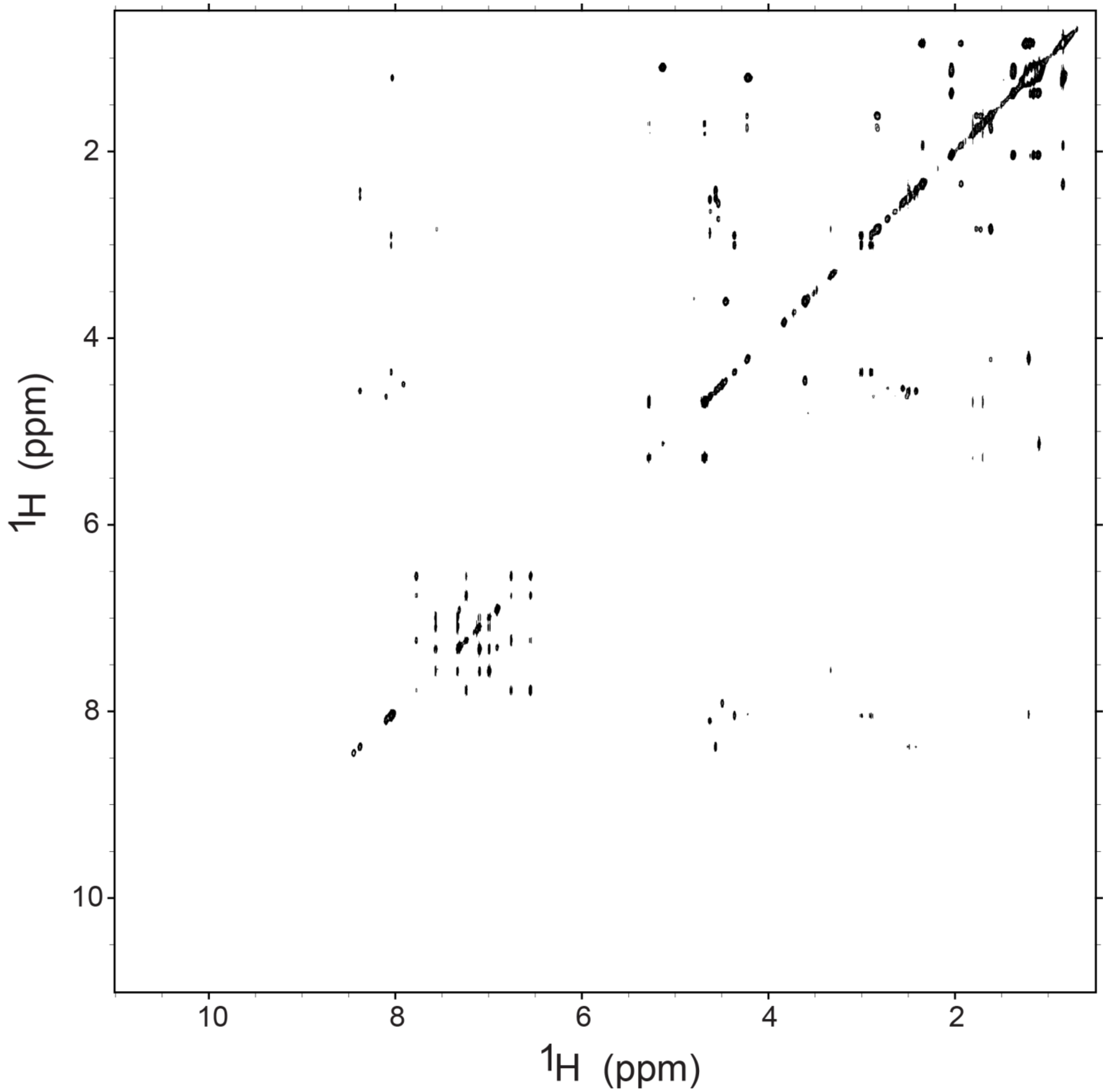

2D ^{1}H-^{1}H TOCSY NMR spectrum (d_6-DMSO, 600 MHz of ^{1}H) of ***N1*-5-Dap** (**40**)

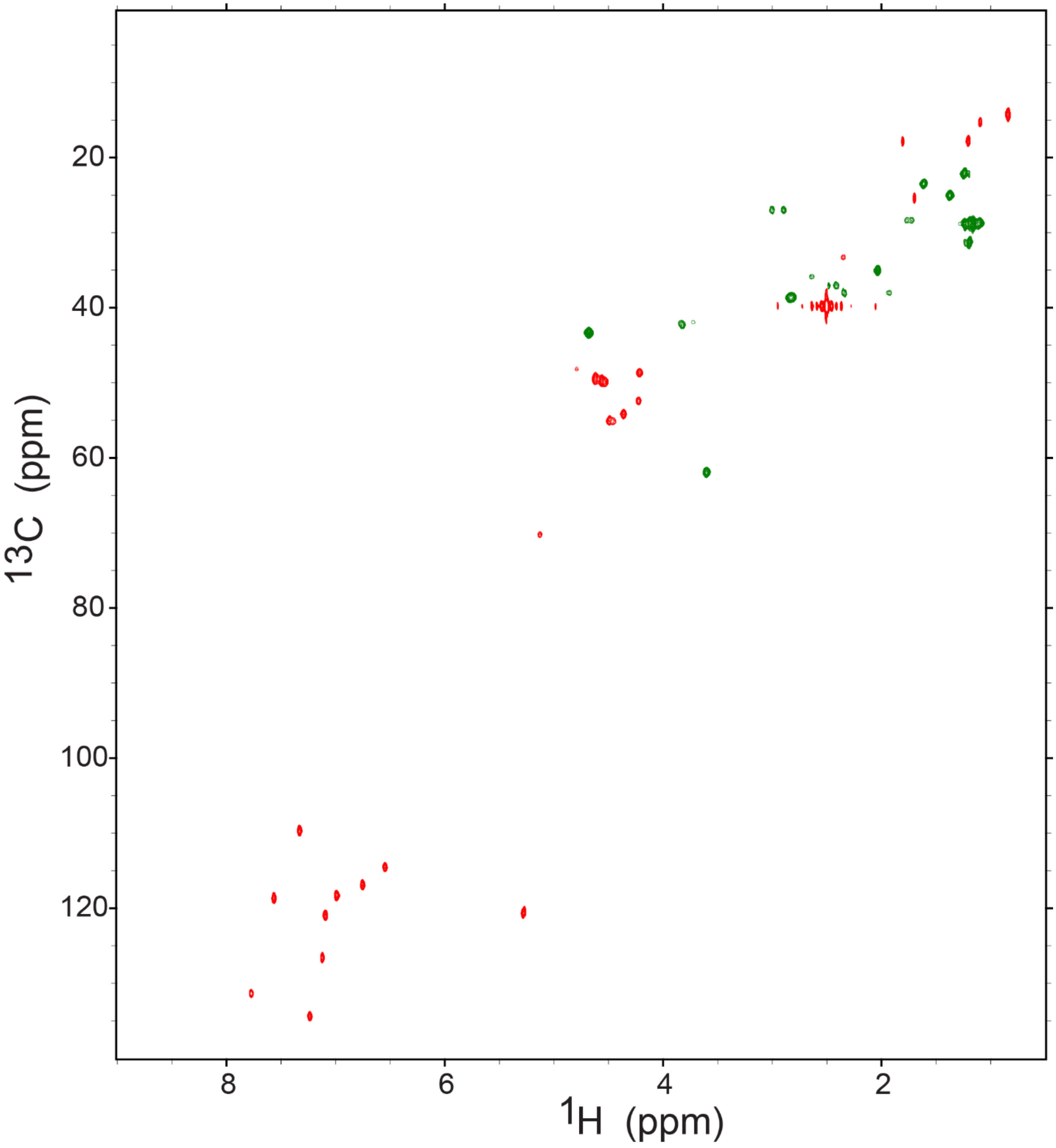

2D ^{1}H-^{13}C HSQC NMR spectrum (d_6-DMSO, 600 MHz of ^{1}H) of ***N1*-5-Dap (40)**

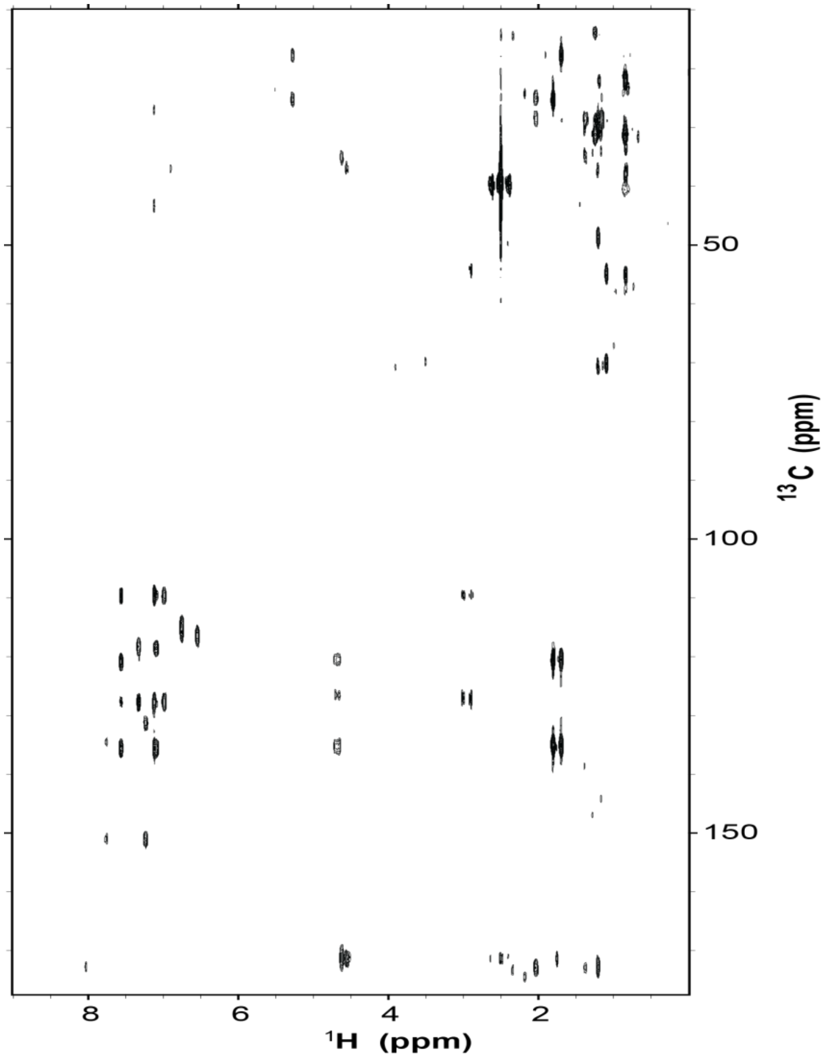

2D ^{1}H-^{13}C HMBC NMR spectrum (d_6-DMSO, 600 MHz of ^{1}H) of ***N1*-5-Dap** (**40)**

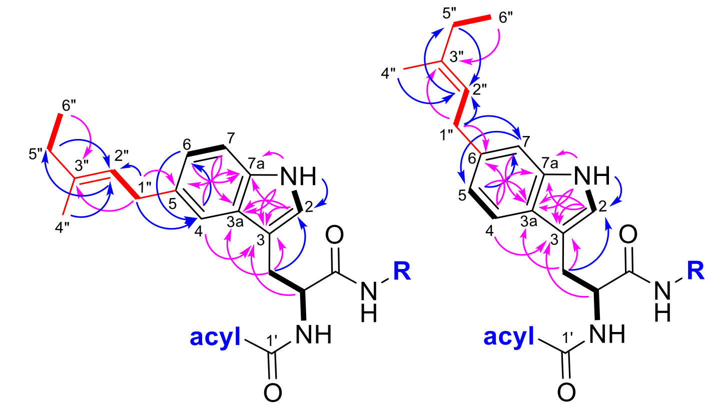

***C5*-6-Dap**

45%

***C6*-6-Dap**

55%

(41)

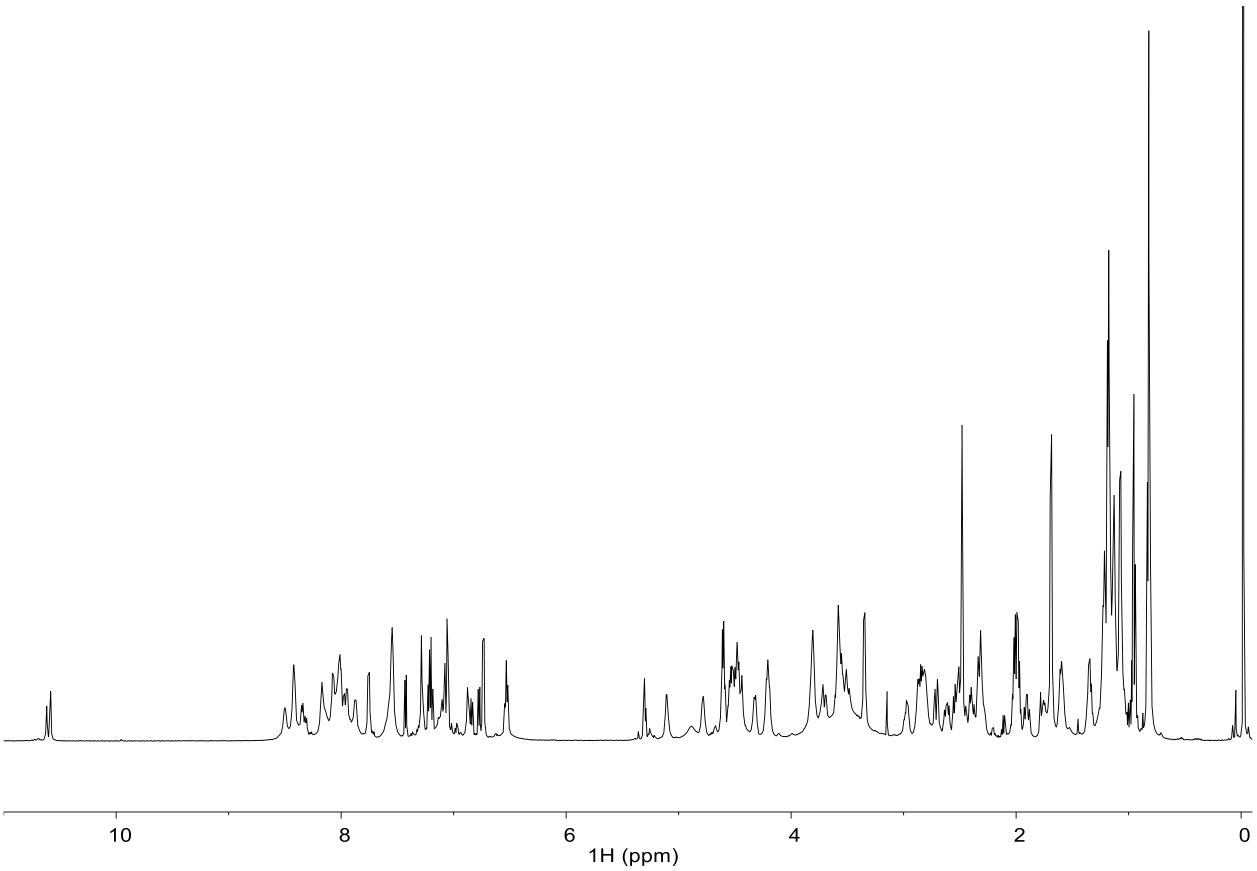

1D ^{1}H NMR spectrum (d_6-DMSO, 600 MHz of ^{1}H) of

0.45: 0.55 mixture of ***C*5-** and ***C*6-6-Dap (41)**

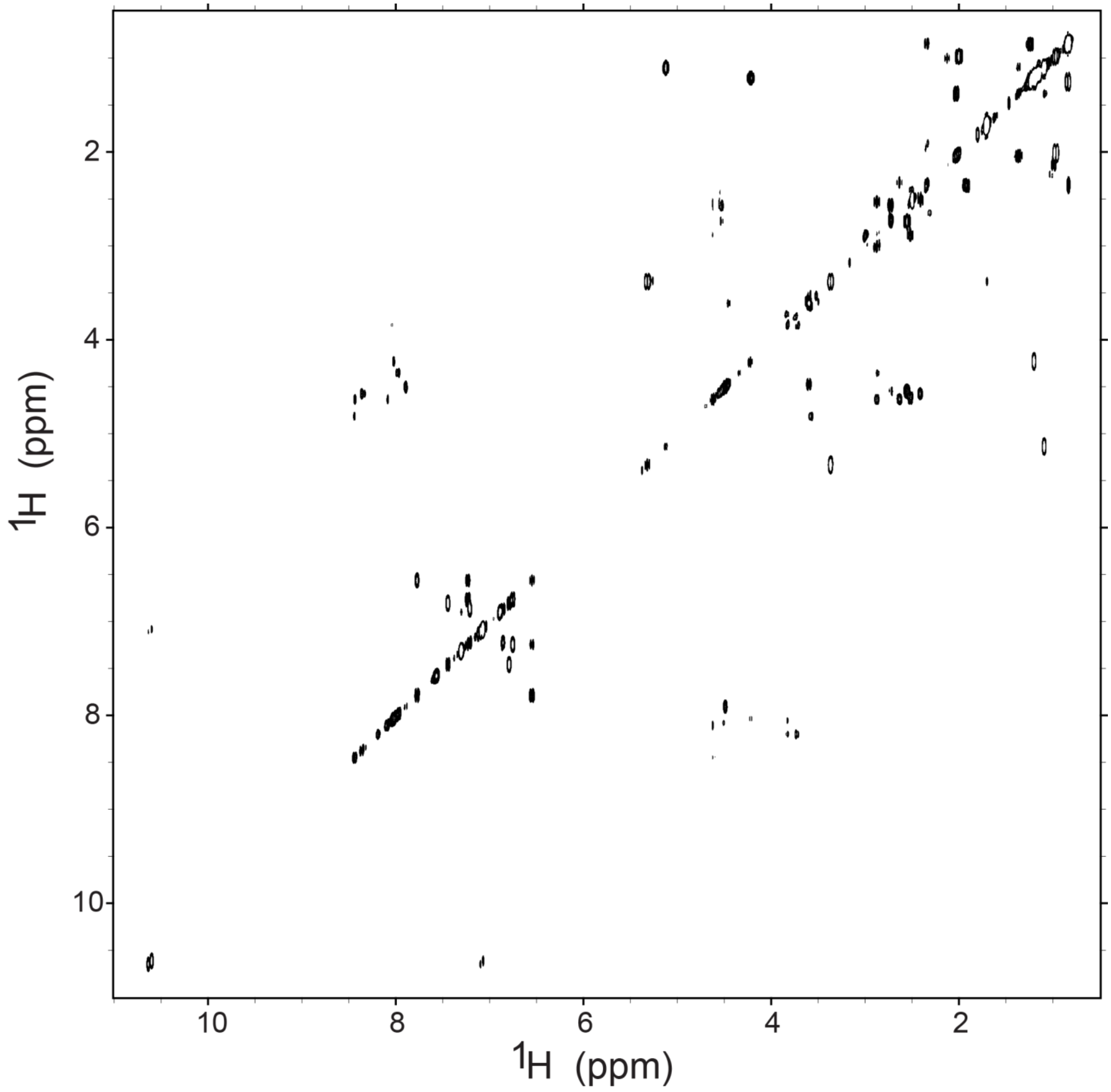

2D ^{1}H-^{1}H COSY NMR spectrum (d_6-DMSO, 600 MHz of ^{1}H) of

0.45: 0.55 mixture of ***C*5-** and ***C*6-6-Dap (41)**

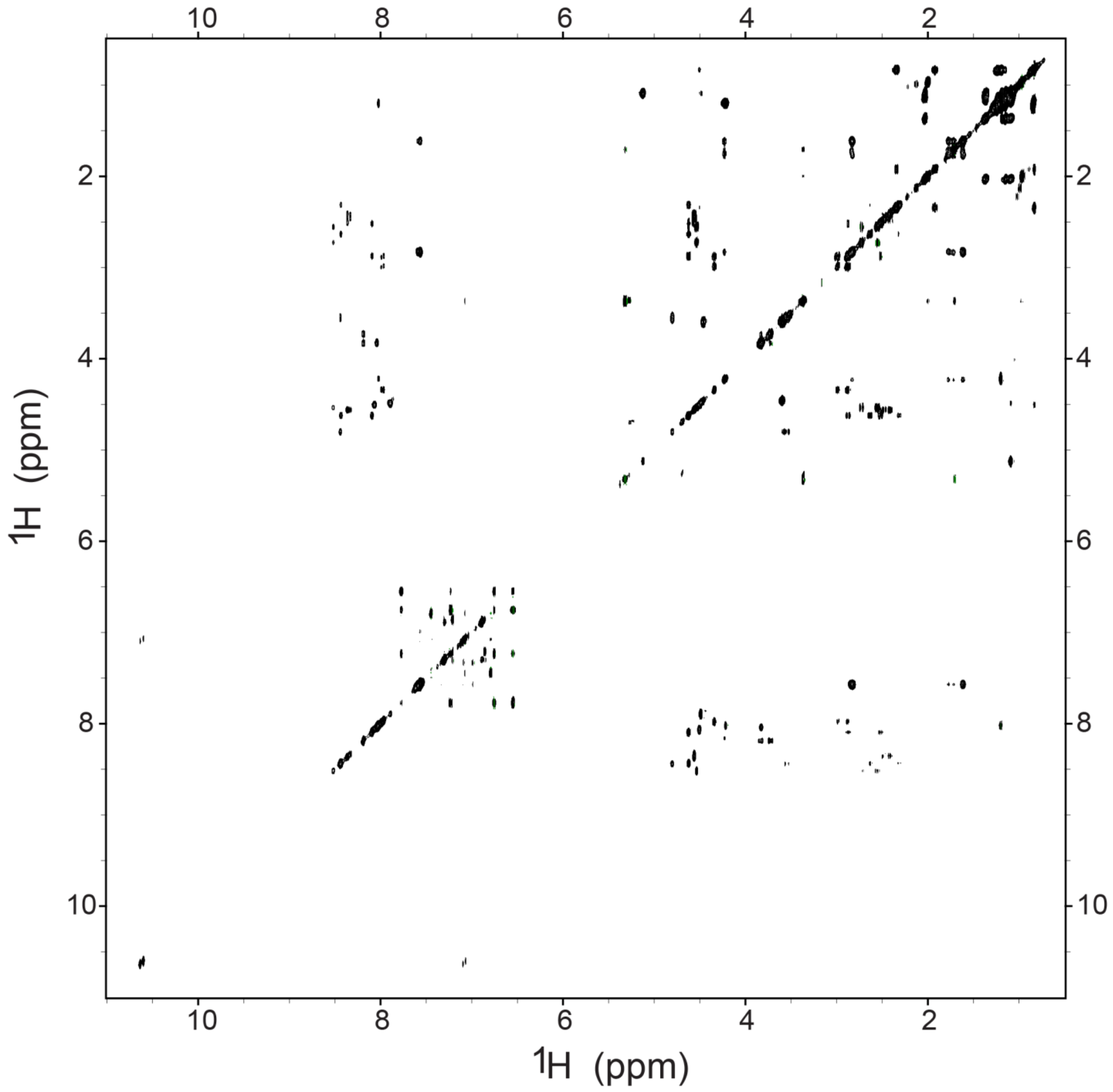

2D ^{1}H-^{1}H TOCSY NMR spectrum (d_6-DMSO, 600 MHz of ^{1}H) of

0.45: 0.55 mixture of ***C*5-** and ***C*6-6-Dap (41)**

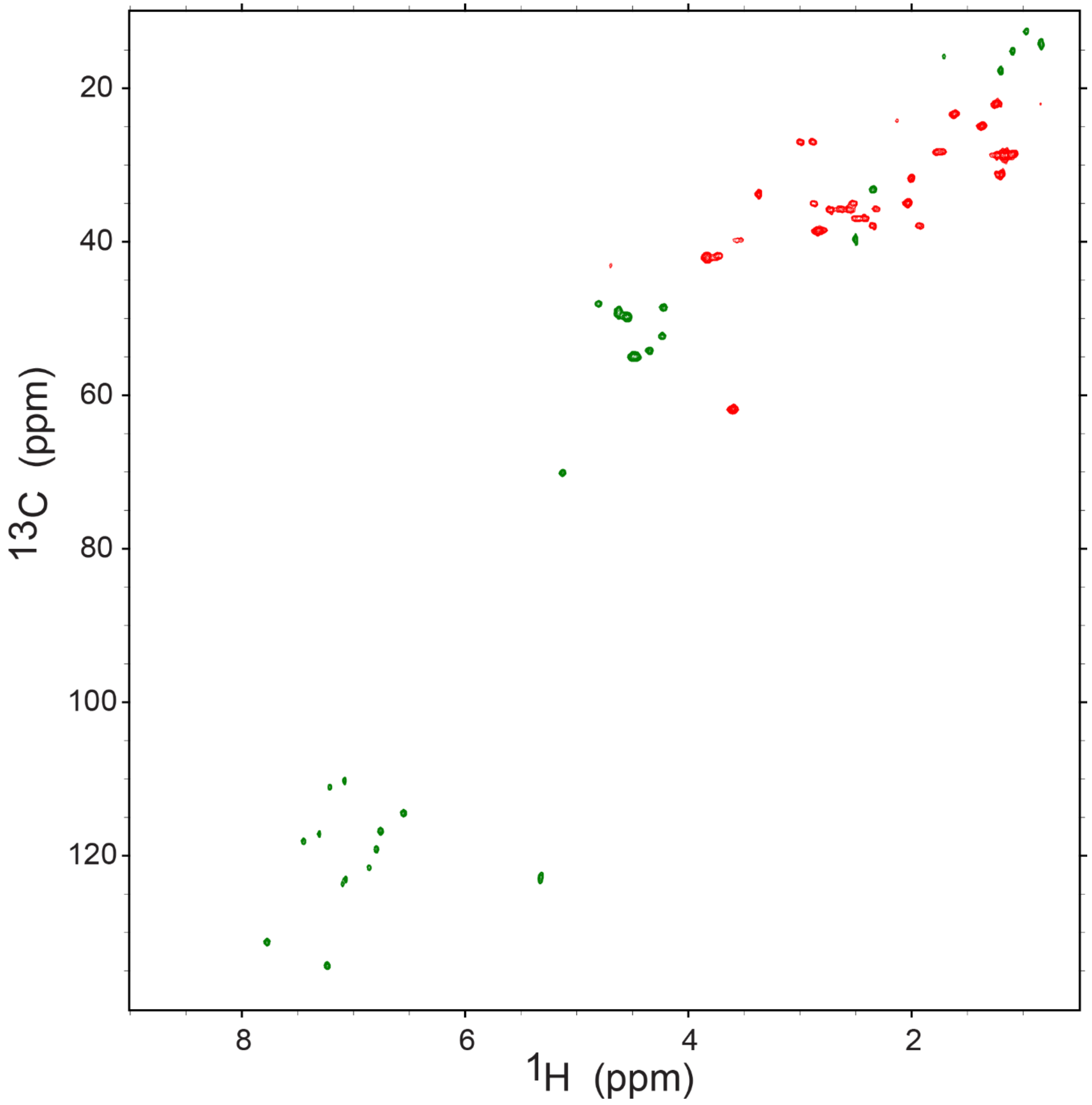

2D ^{1}H-^{13}C HSQC NMR spectrum (d$_6$-DMSO, 600 MHz of ^{1}H) of

0.45: 0.55 mixture of ***C*5-** and ***C*6-6-Dap (41)**

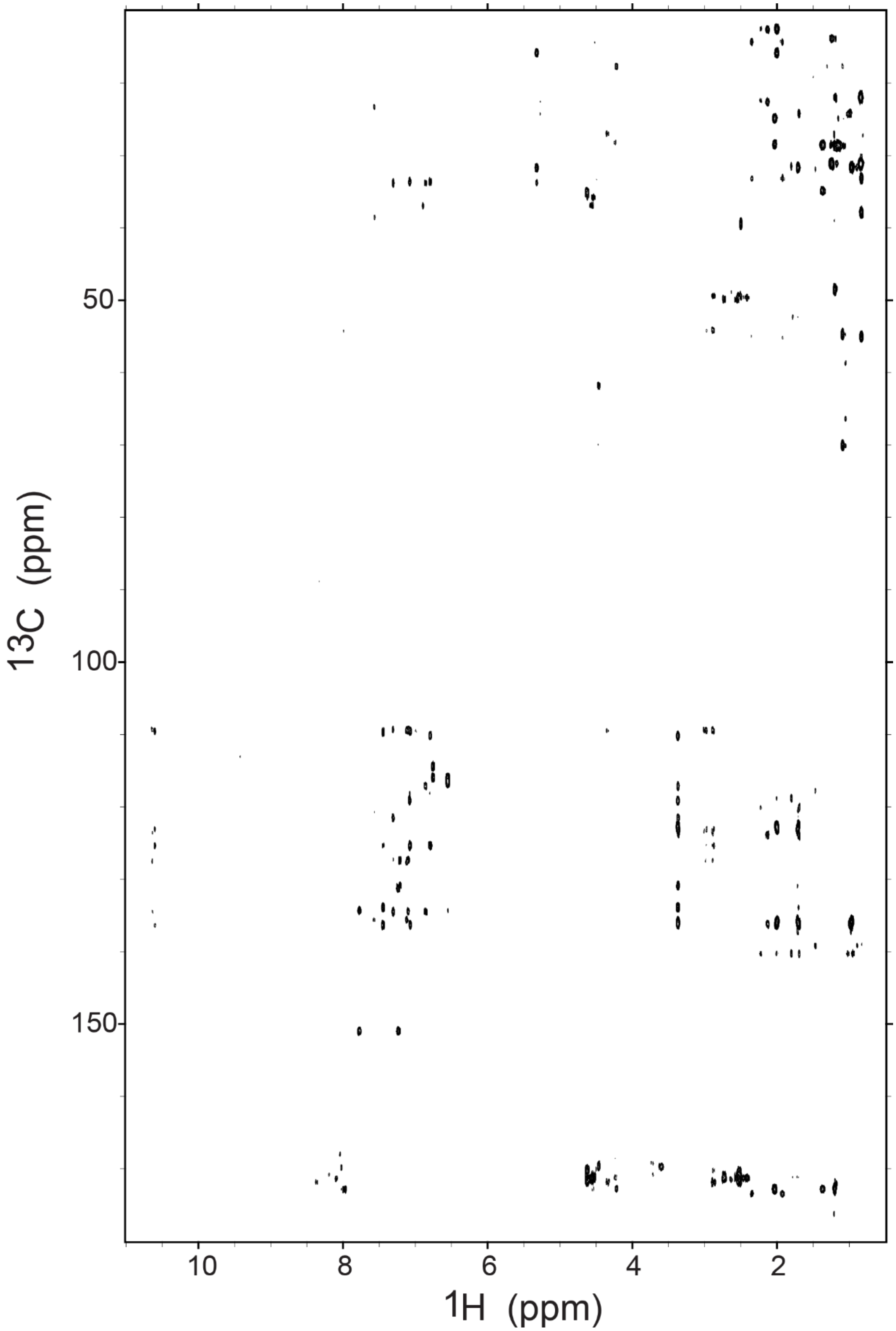

2D ^{1}H-^{13}C HMBC NMR spectrum (d_6-DMSO, 600 MHz of ^{1}H) of

0.45: 0.55 mixture of ***C*5-** and ***C*6-6-Dap (41)**

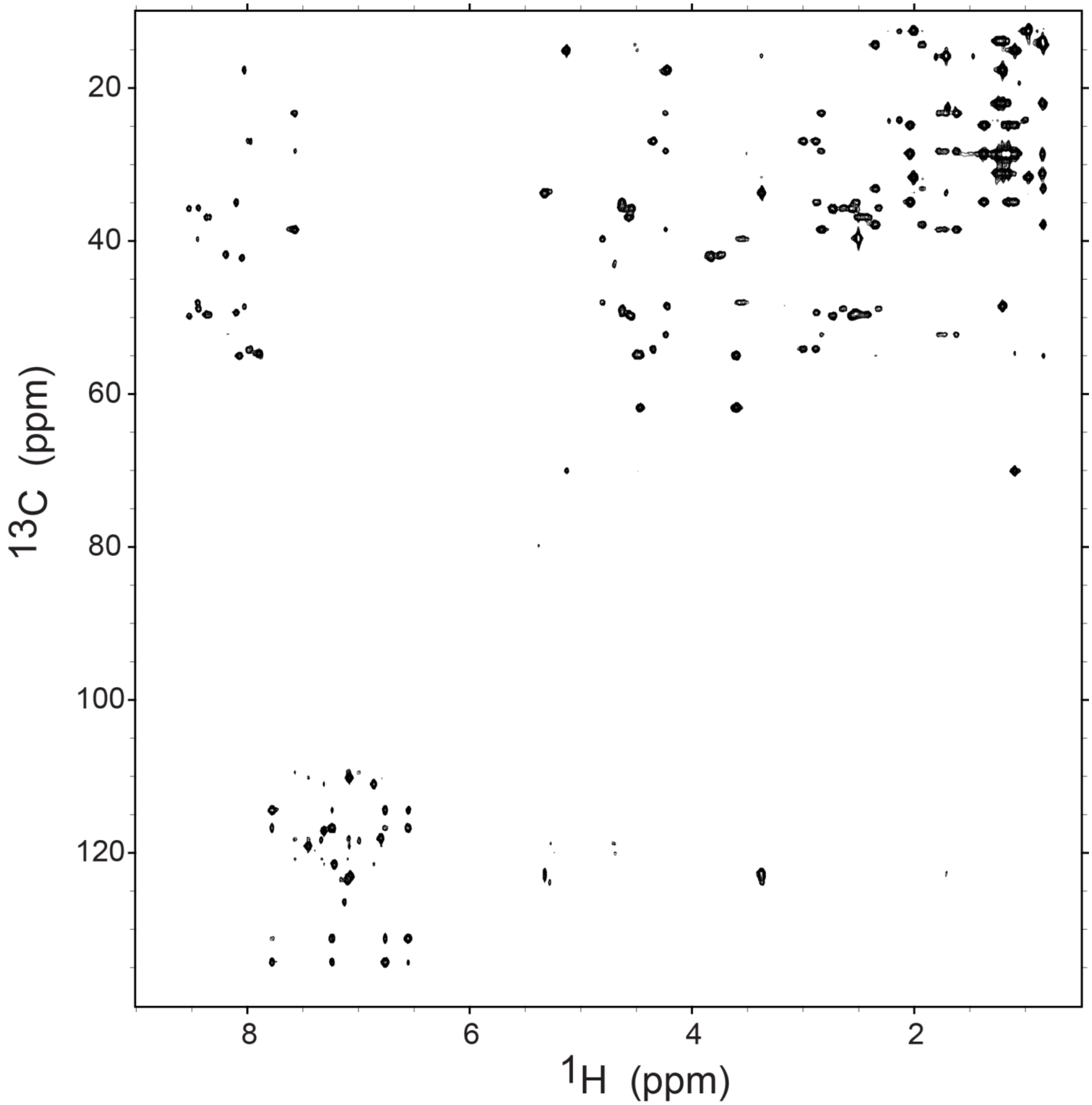

2D ^{1}H-^{13}C HSQC-TOCSY NMR spectrum (d_6-DMSO, 600 MHz of ^{1}H) of 0.45: 0.55 mixture of ***C*5-** and ***C*6-6-Dap (41)**

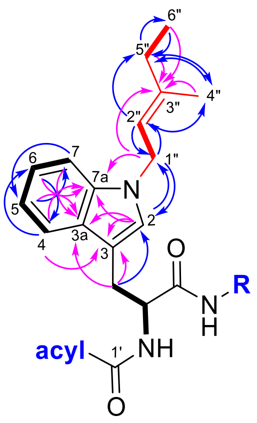

***N1*-6-Dap (42)**

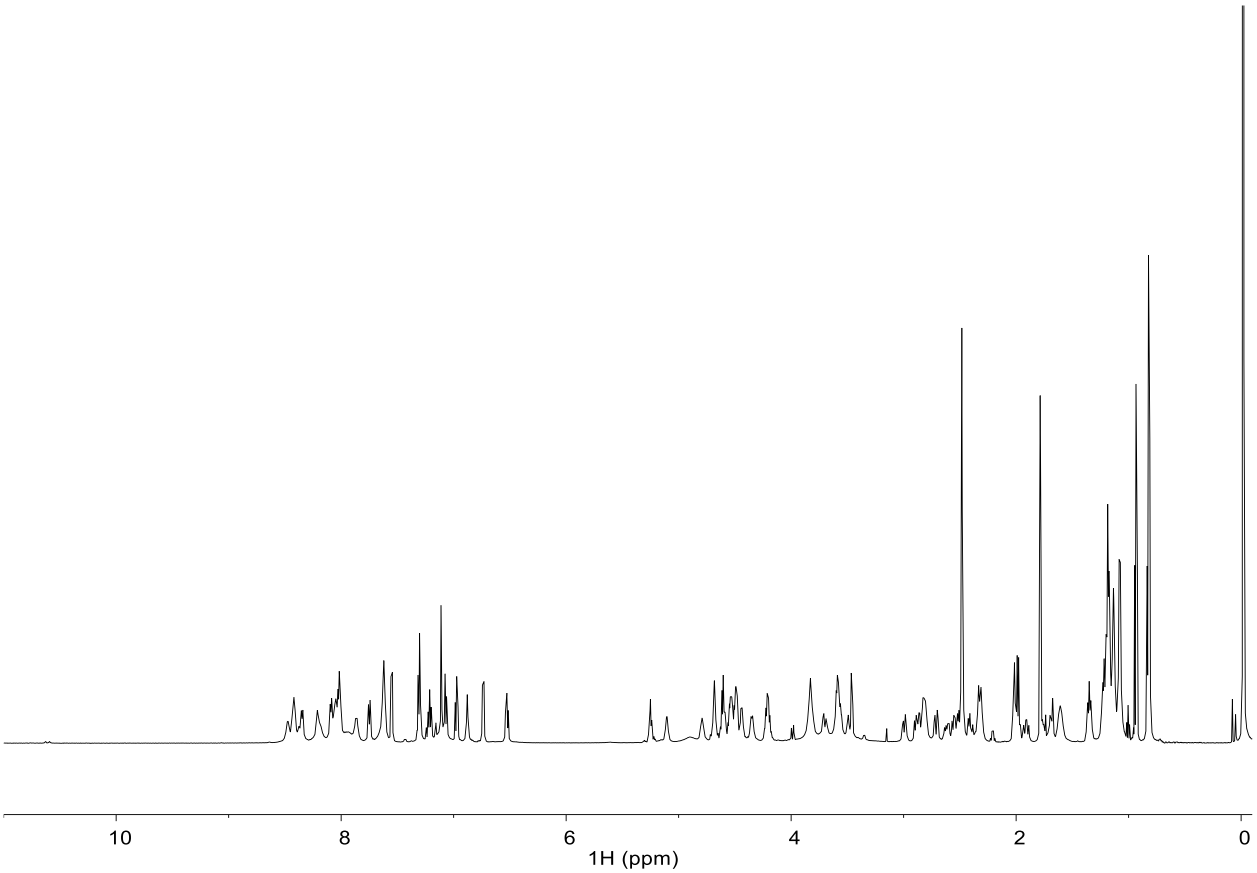

1D ^{1}H NMR spectrum (d_6-DMSO, 600 MHz of ^{1}H) of ***N1*-6-Dap (42)**

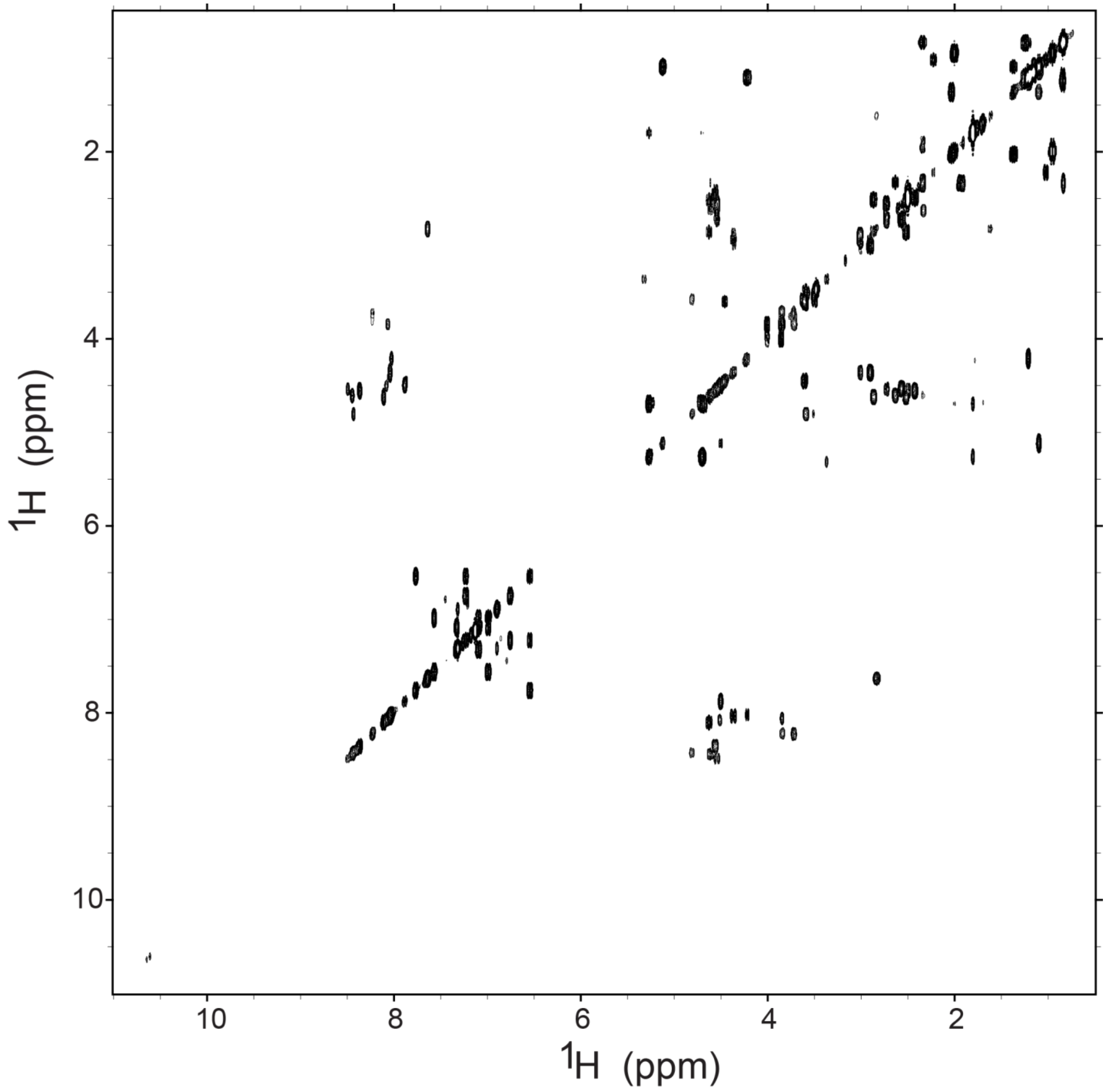

2D ^{1}H-^{1}H COSY NMR spectrum (d_6-DMSO, 600 MHz of ^{1}H) of ***N1*-6-Dap (42)**

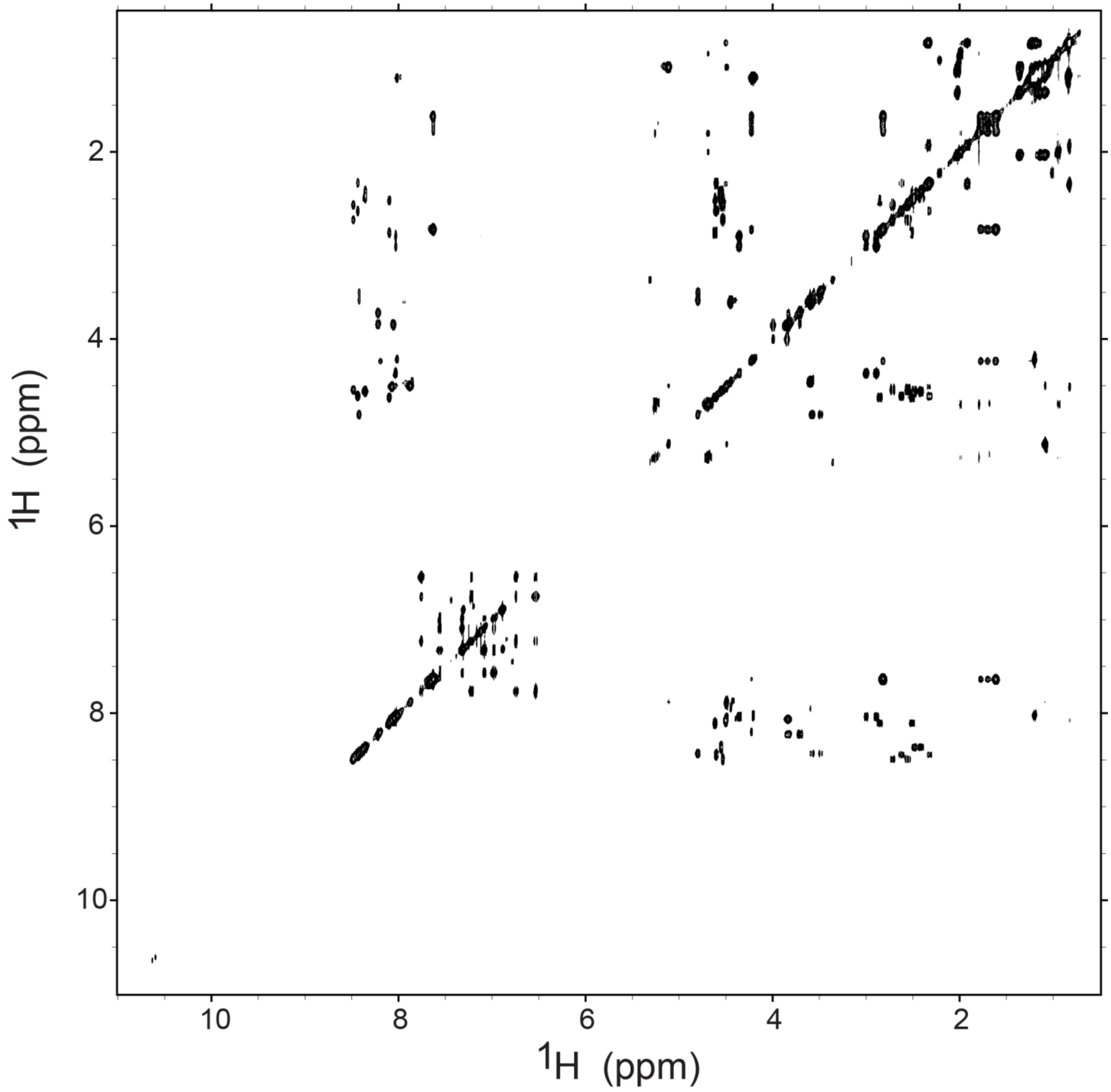

2D ^{1}H-^{1}H TOCSY NMR spectrum (d_6-DMSO, 600 MHz of ^{1}H) of ***N1*-6-Dap (42)**

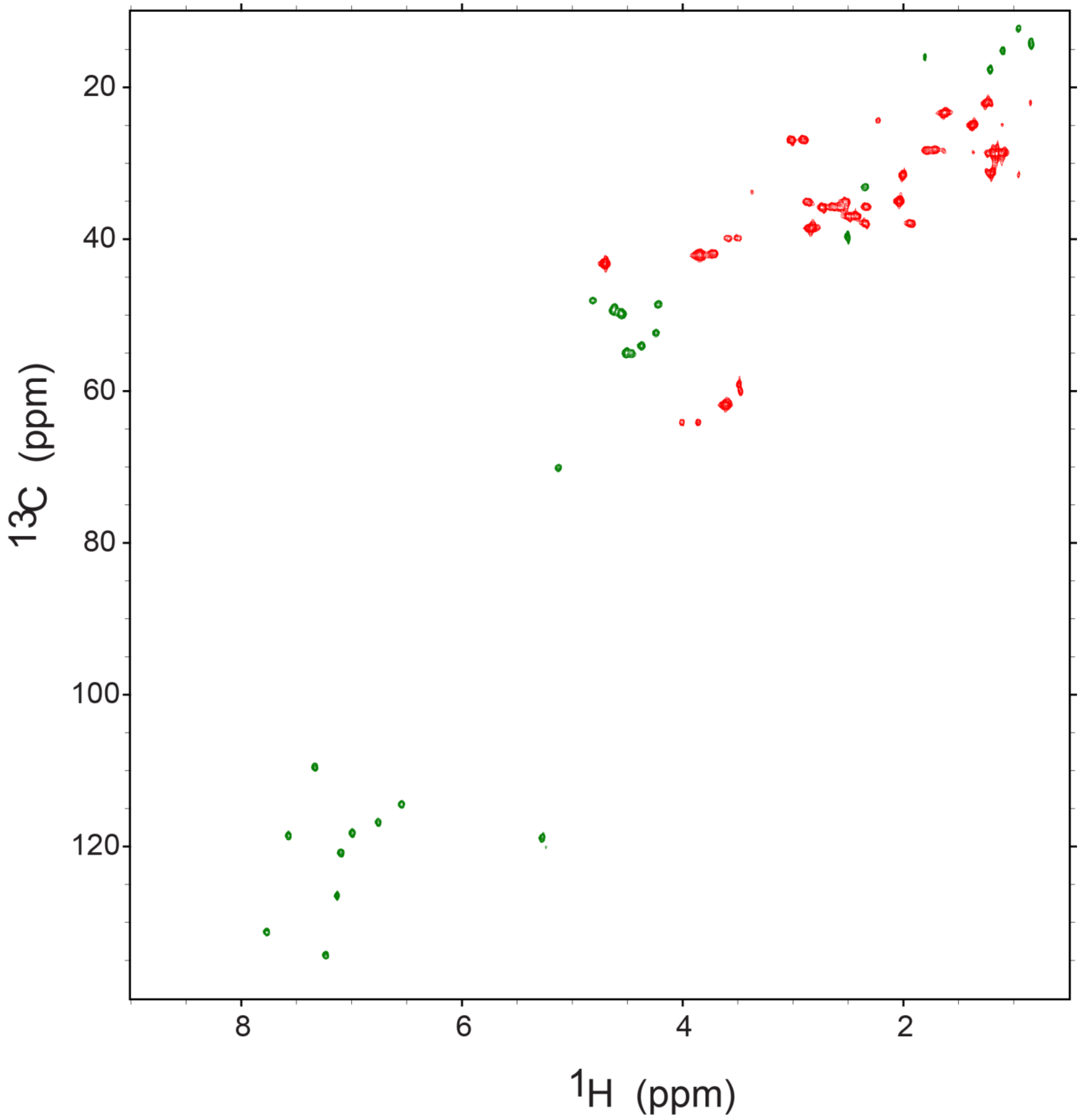

2D ^{1}H-^{13}C HSQC NMR spectrum (d_6-DMSO, 600 MHz of ^{1}H) of ***N1*-6-Dap (42)**

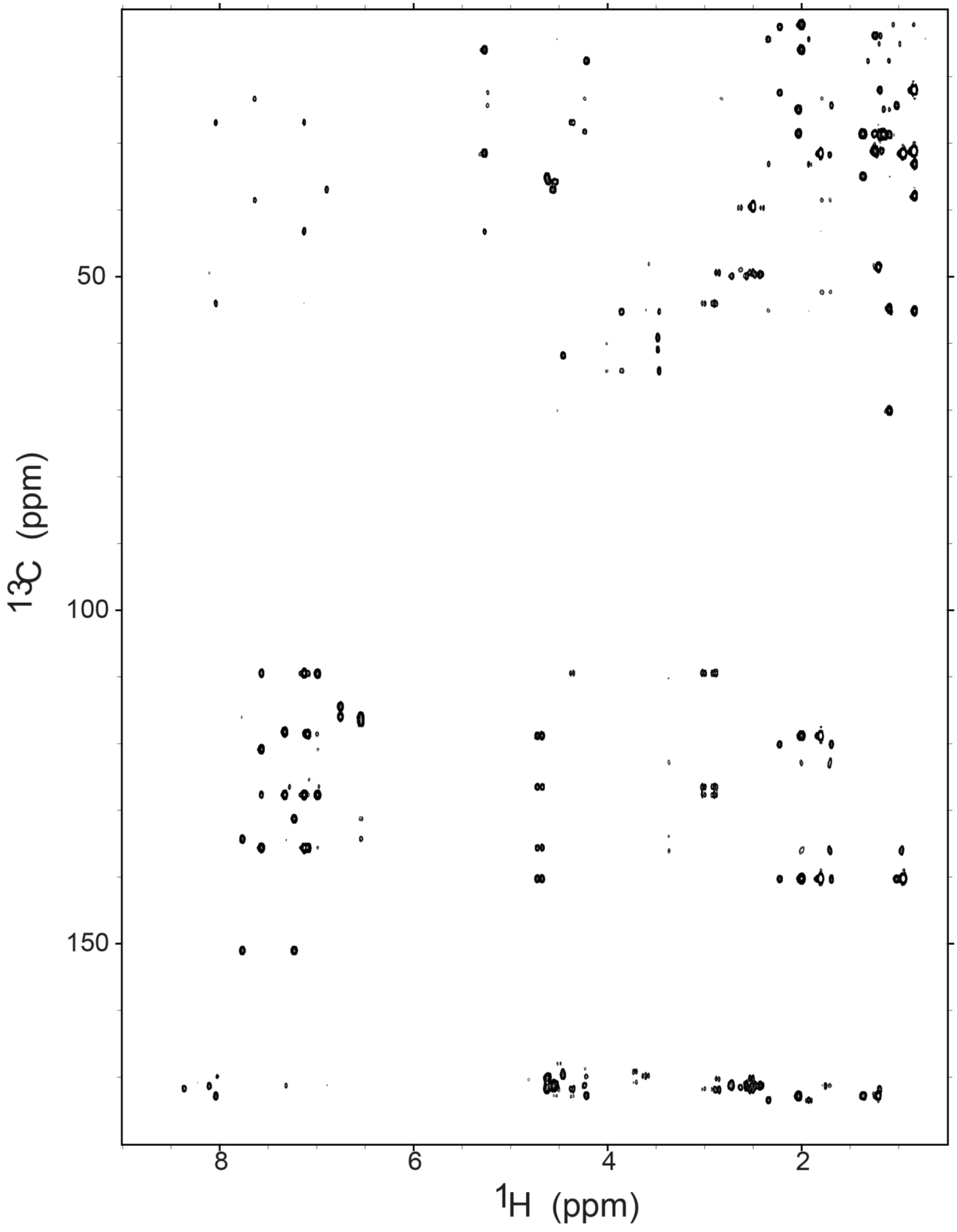

2D ^{1}H-^{13}C HMBC NMR spectrum (d_6-DMSO, 600 MHz of ^{1}H) of ***N1*-6-Dap (42)**

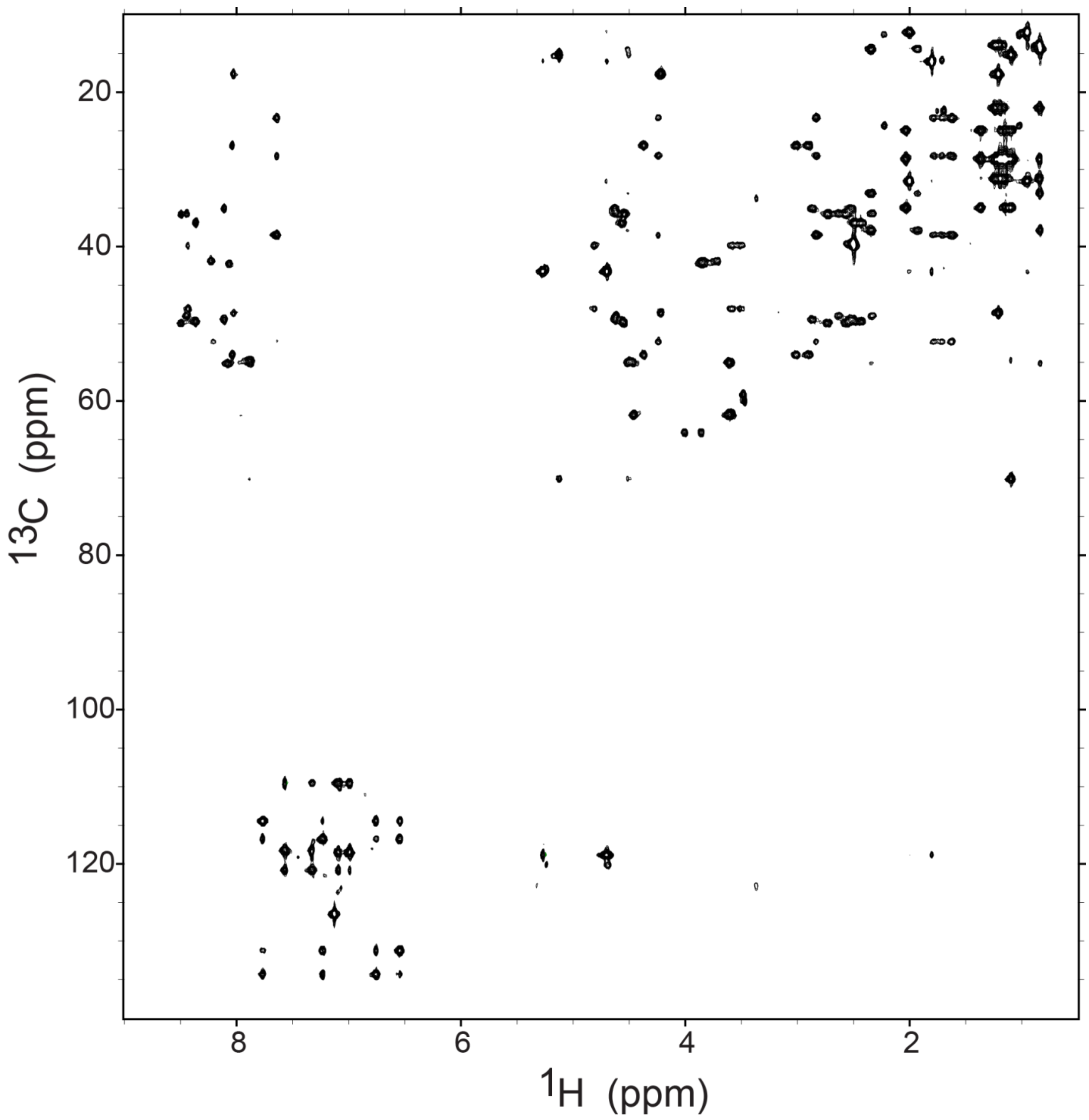

2D ^{1}H-^{13}C HSQC-TOCSY NMR spectrum (d_6-DMSO, 600 MHz of ^{1}H) of ***N1*-6-Dap (42)**

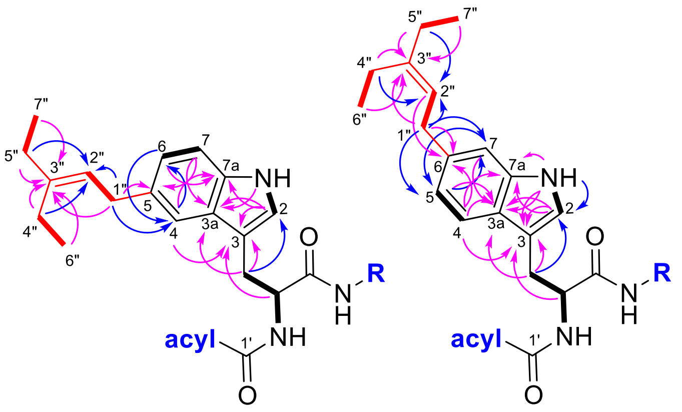

***C5*-7-Dap**
15%

***C6*-7-Dap**
85%

(43)

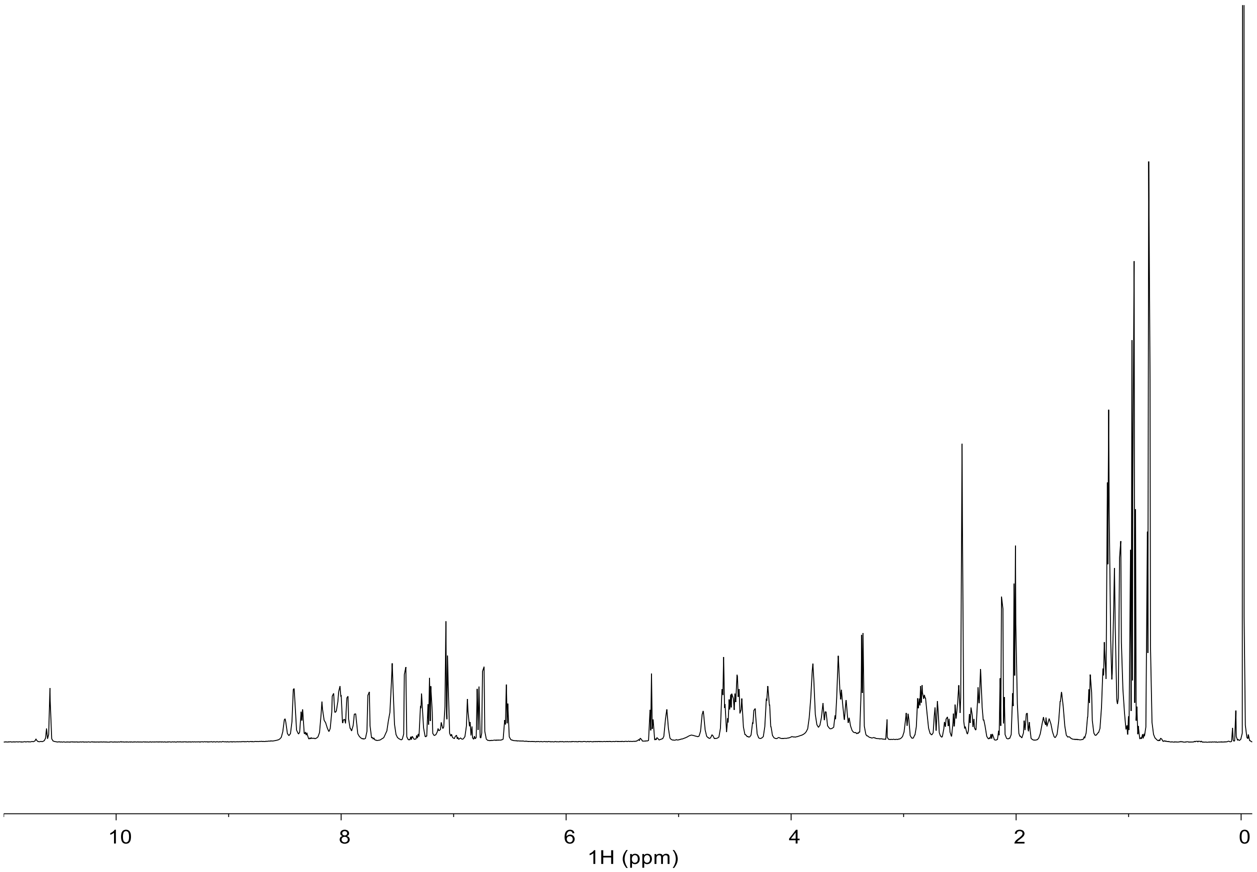

1D ^{1}H NMR spectrum (d$_6$-DMSO, 600 MHz of ^{1}H) of a mixture of 0.15:0.85 mixture of ***C5*-** and ***C6*-7-Dap (43)**

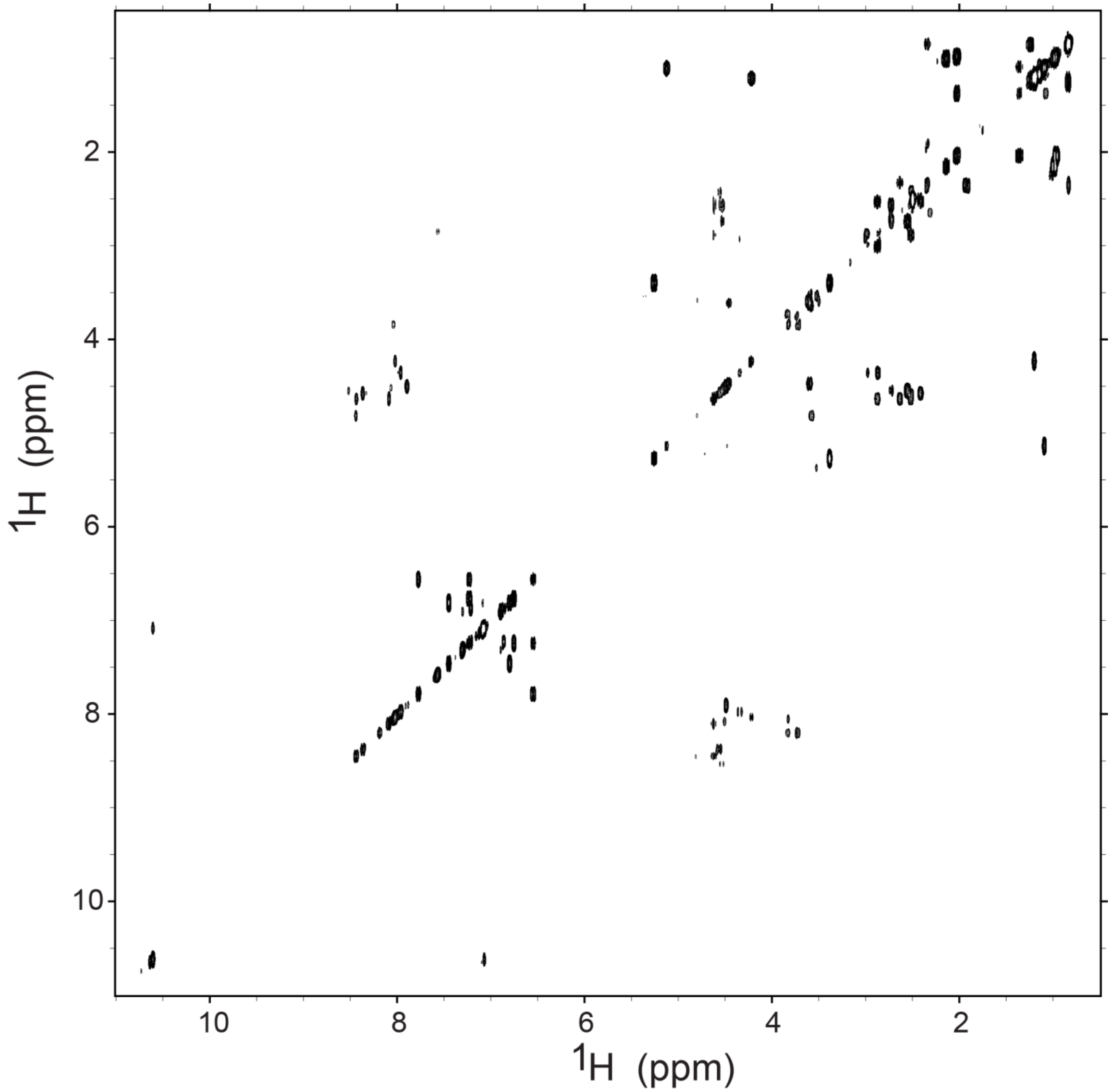

2D ^{1}H-^{1}H COSY NMR spectrum (d_6-DMSO, 600 MHz of ^{1}H) of

0.15:0.85 mixture of ***C5*- and *C6*-7-Dap (43)**

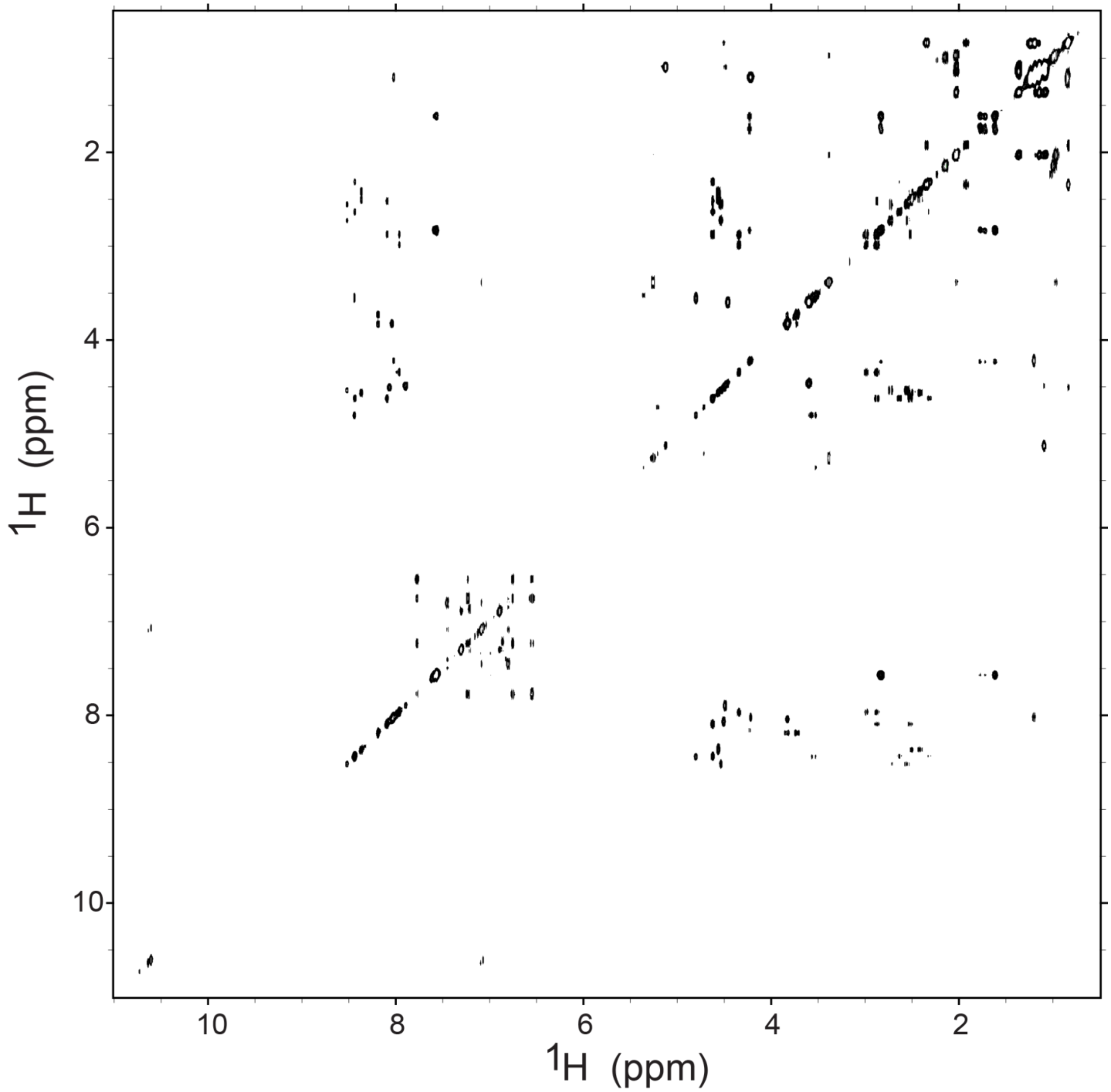

2D ^{1}H-^{1}H TOCSY NMR spectrum (d_6-DMSO, 600 MHz of ^{1}H) of

0.15:0.85 mixture of ***C5*- and *C6*-7-Dap (43)**

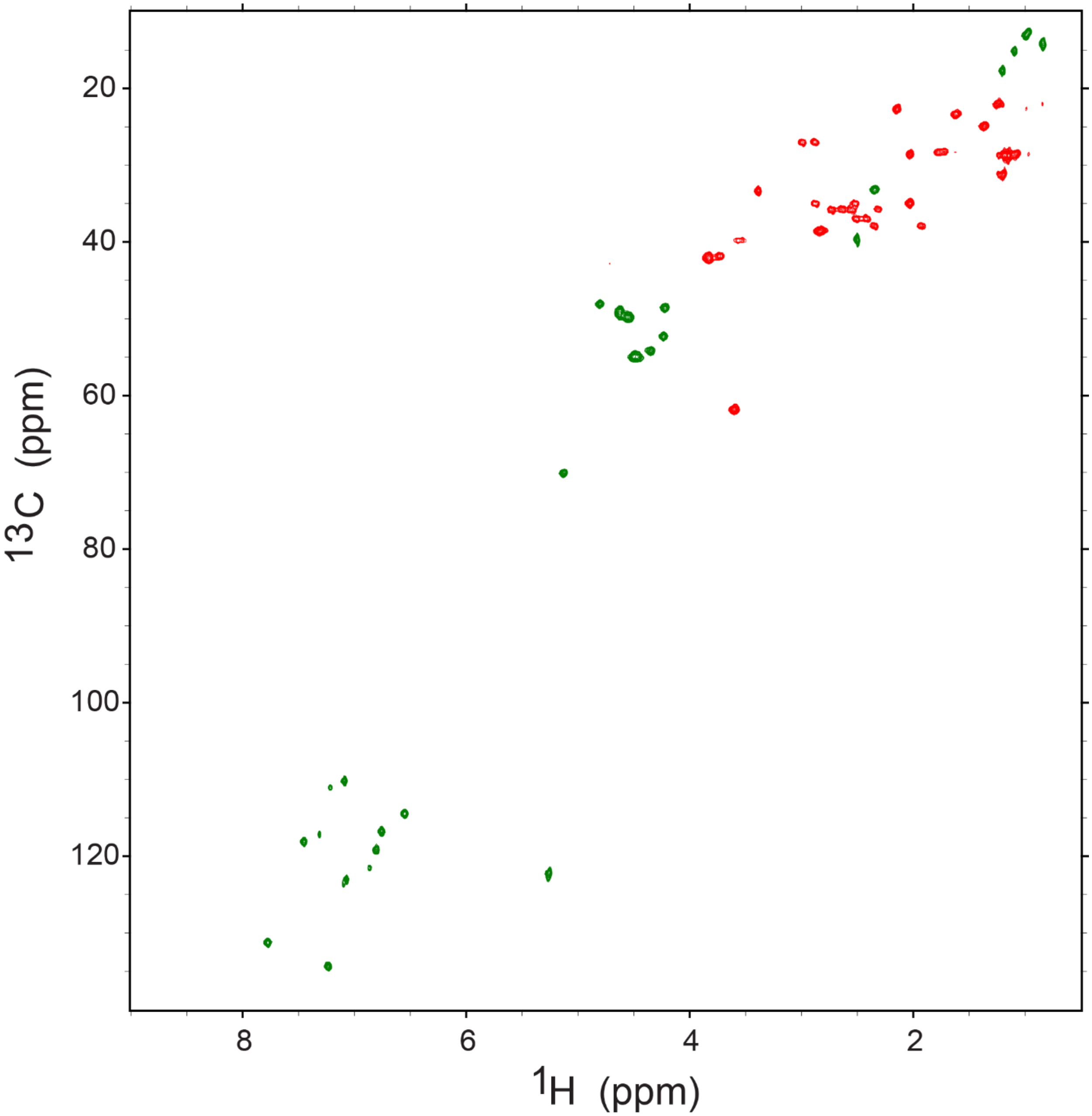

2D ^{1}H-^{13}C HSQC NMR spectrum (d_6-DMSO, 600 MHz of ^{1}H) of

0.15:0.85 mixture of ***C5-*** and ***C6*-7-Dap (43)**

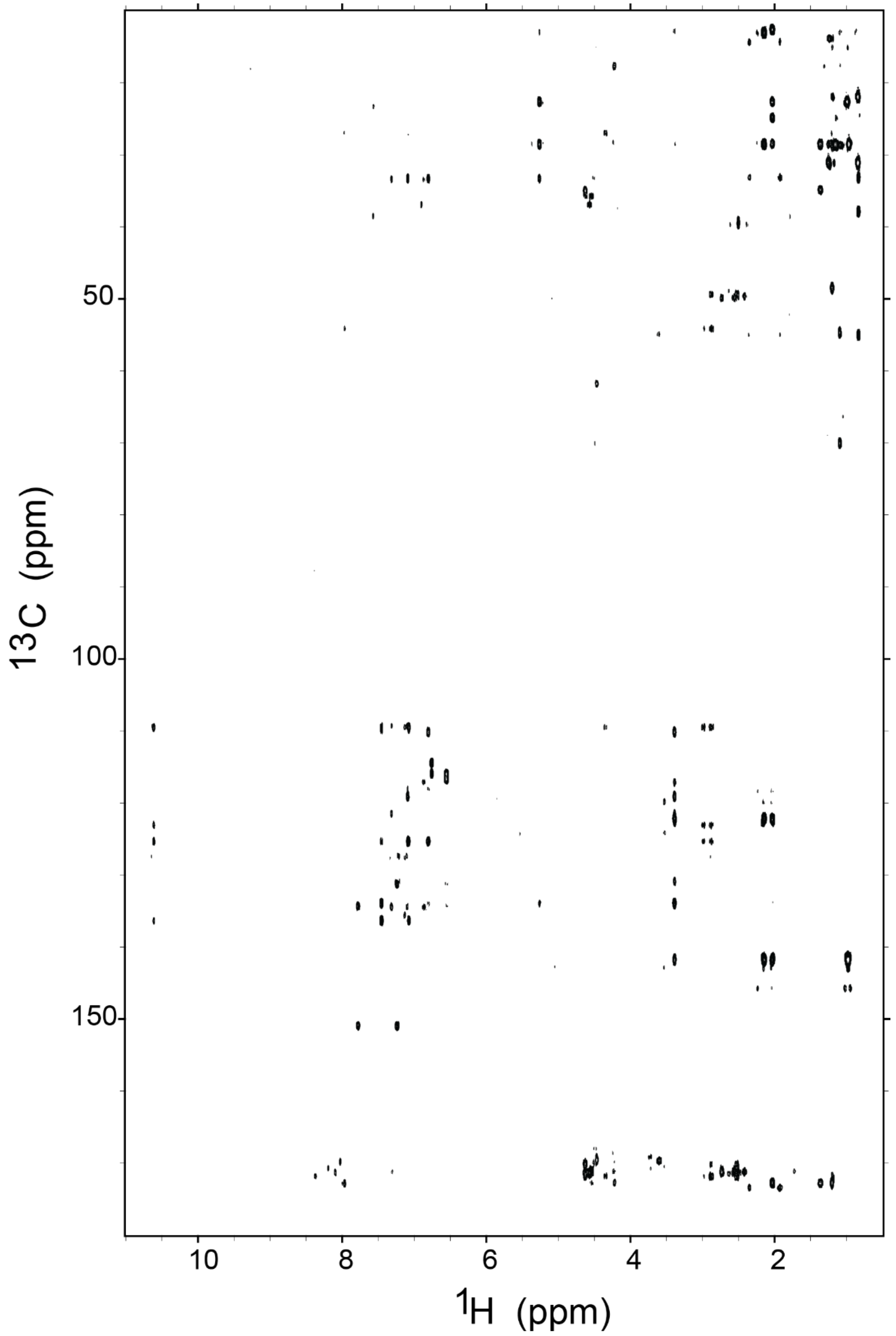

2D ^{1}H-^{13}C HMBC NMR spectrum (d_6-DMSO, 600 MHz of ^{1}H) of 0.15:0.85 mixture of ***C5*-** and ***C6*-7-Dap (43)**

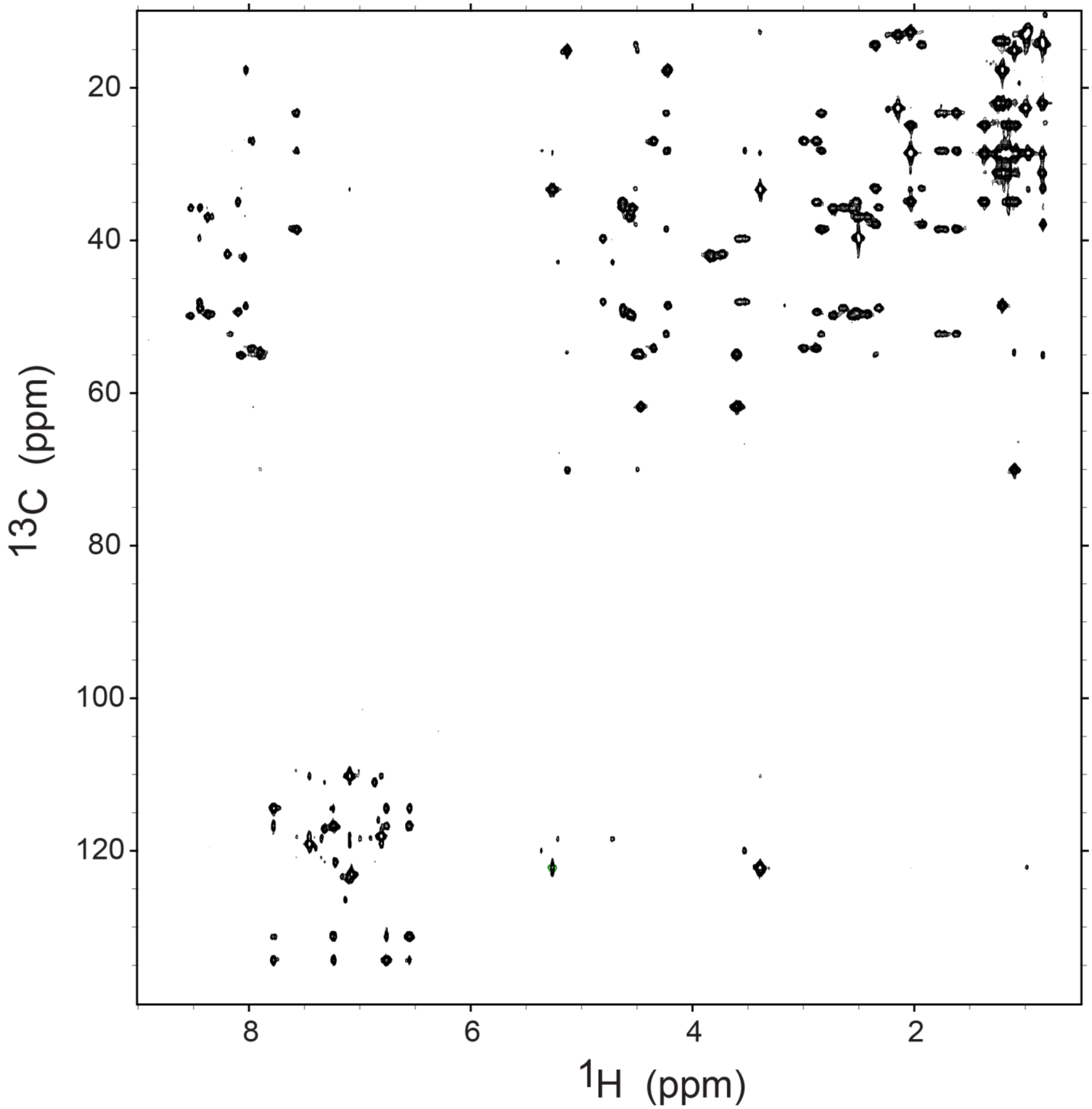

2D ^{1}H-^{13}C HSQC-TOCSY NMR spectrum (d_6-DMSO, 600 MHz of ^{1}H) of 0.15:0.85 mixture of ***C5*-** and ***C6*-7-Dap (43)**

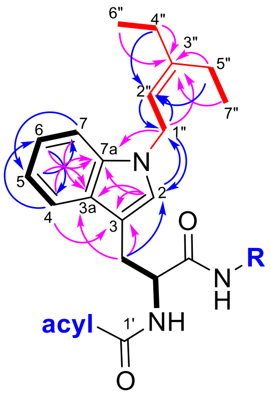

***N1*-7-Dap**
90%
(44)

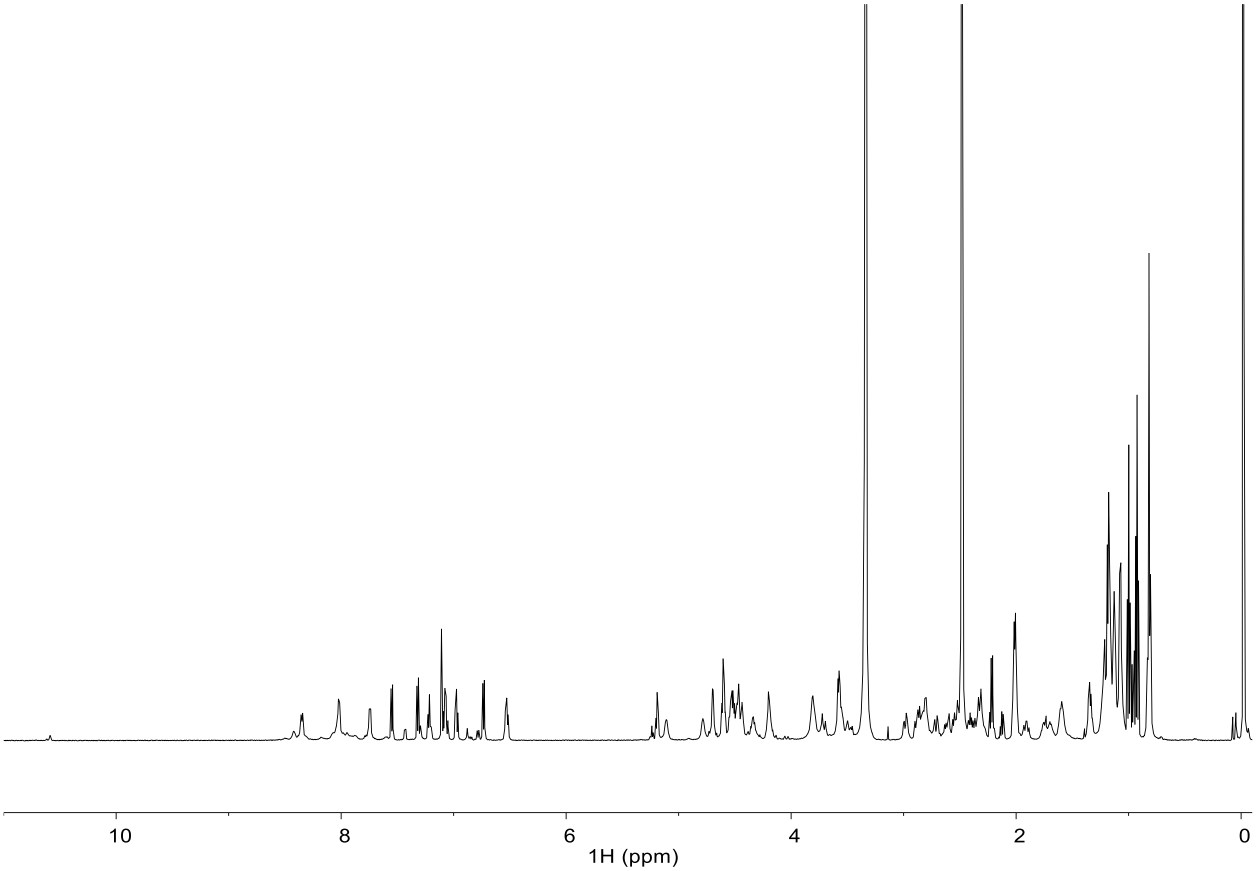

1D ^{1}H NMR spectrum (d_6-DMSO, 600 MHz of ^{1}H) of

90% pure ***N1*-7-Dap (44)**

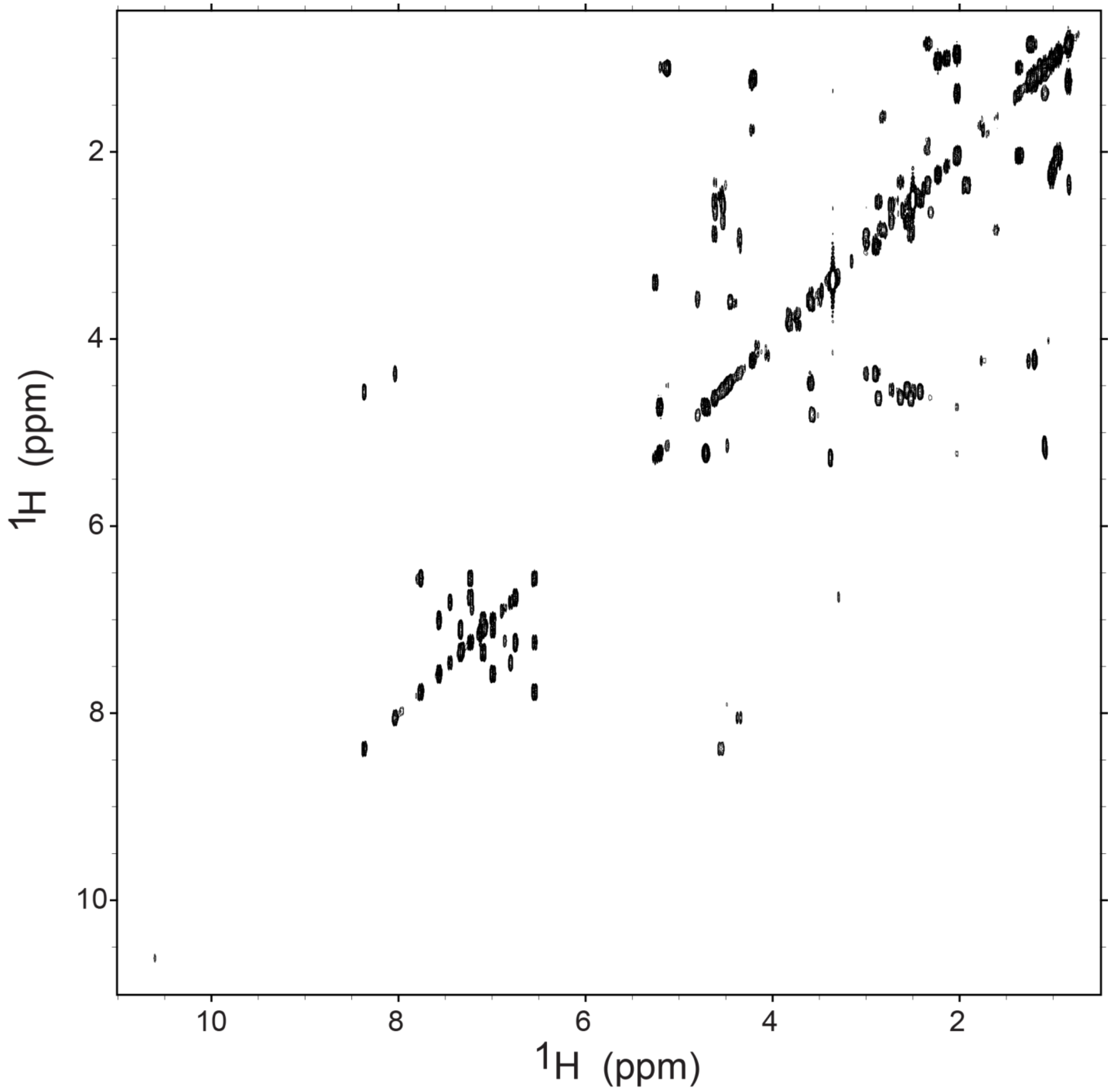

2D ^{1}H-^{1}H COSY NMR spectrum (d_6-DMSO, 600 MHz of ^{1}H) of

90% pure ***N1*-7-Dap (44)**

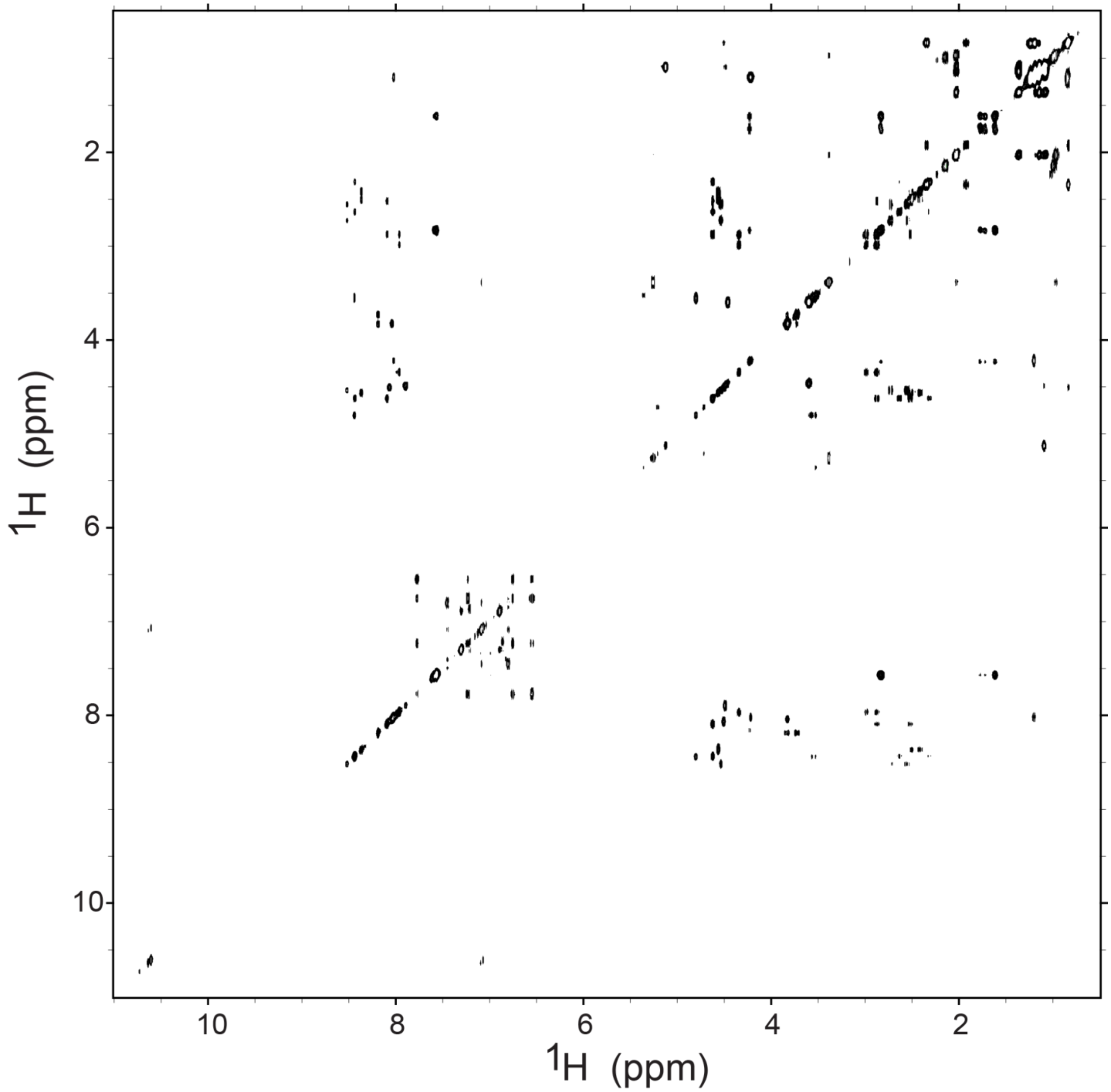

2D ^{1}H-^{1}H TOCSY NMR spectrum (d_6-DMSO, 600 MHz of ^{1}H) of

90% pure ***N1*-7-Dap (44)**

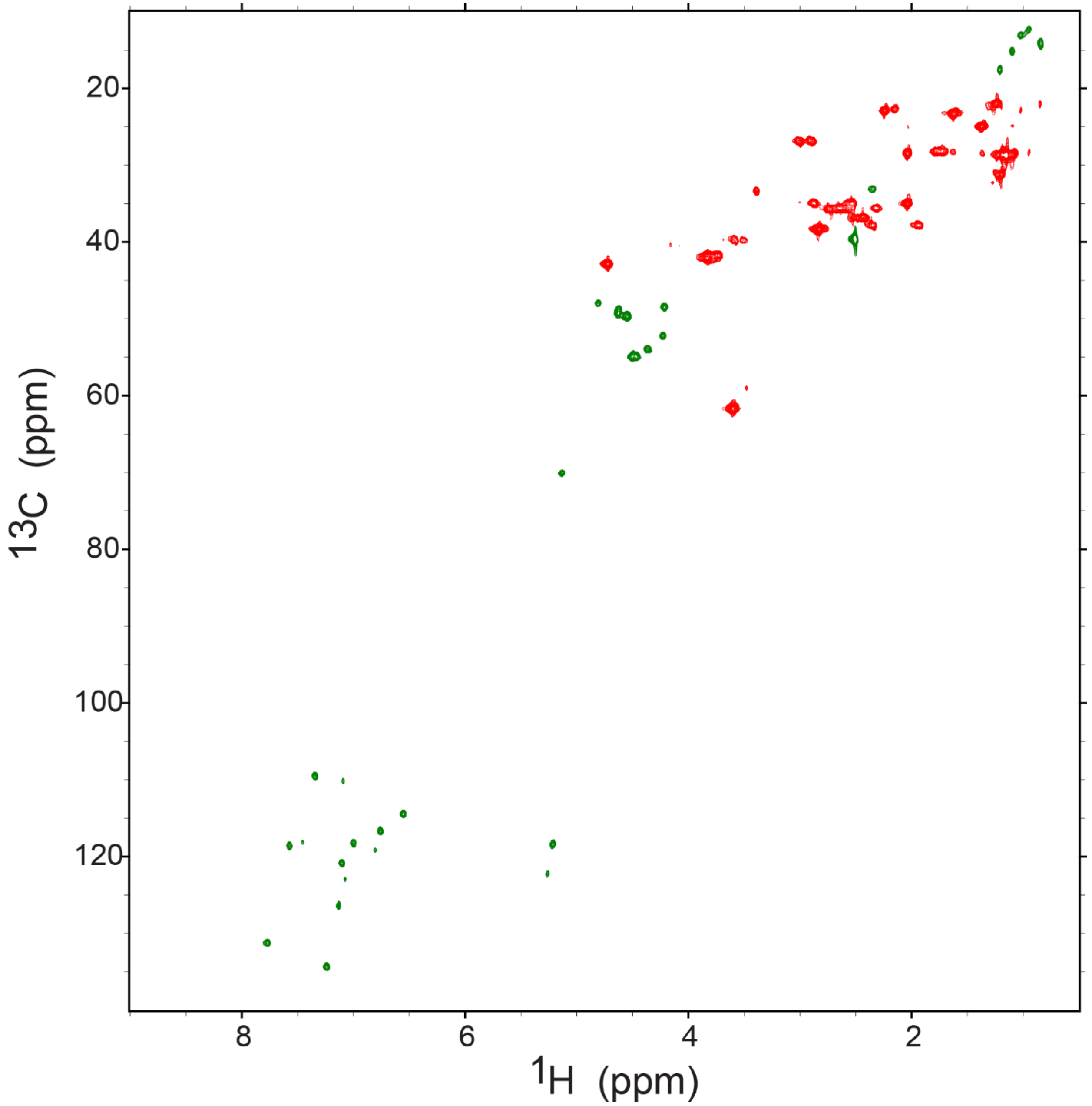

2D ^{1}H-^{13}C HSQC NMR spectrum (d_6-DMSO, 600 MHz of ^{1}H) of

90% pure ***N1*-7-Dap (44)**

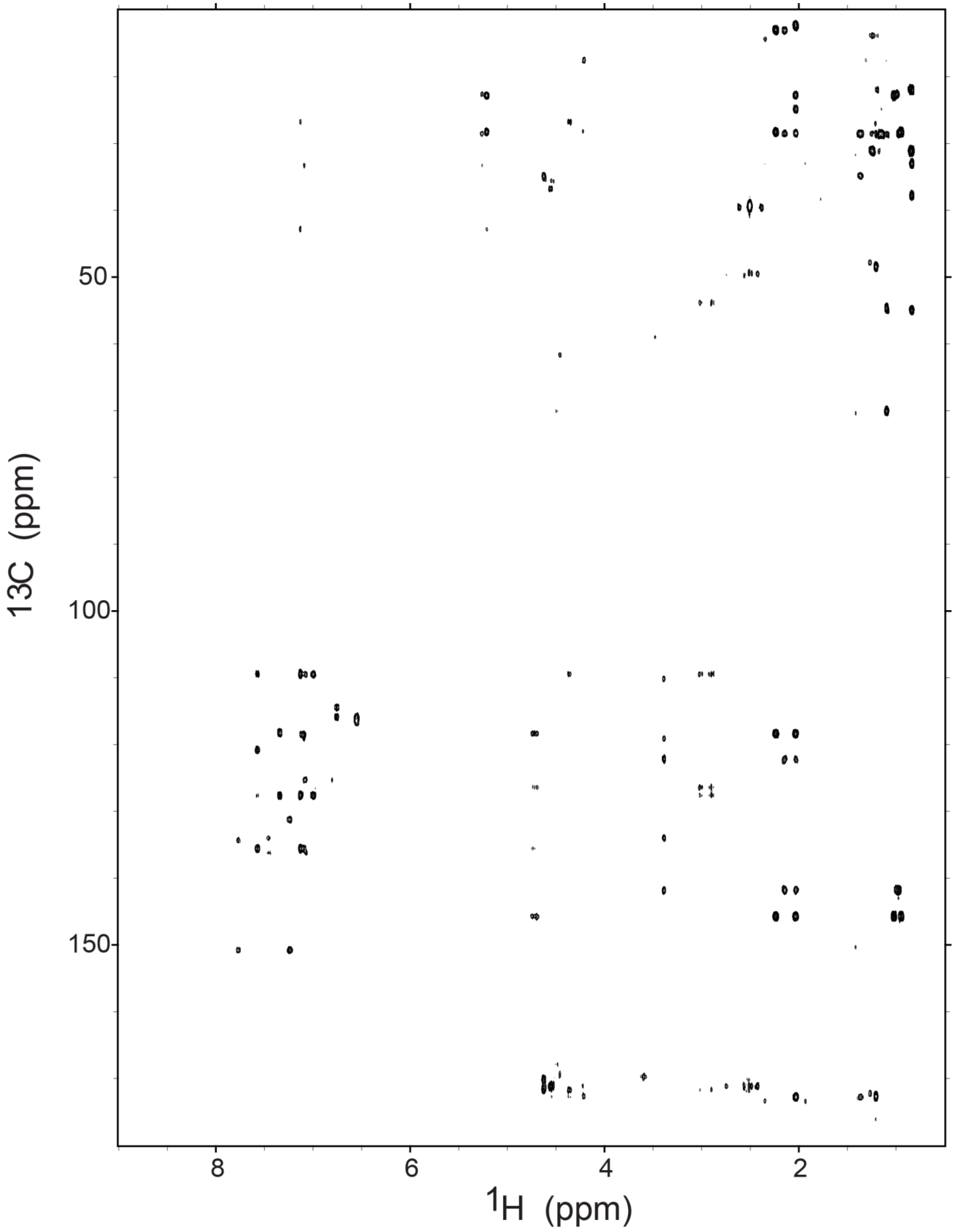

2D ^{1}H-^{13}C HMBC NMR spectrum (d_6-DMSO, 600 MHz of ^{1}H) of

90% pure ***N1*-7-Dap (44)**

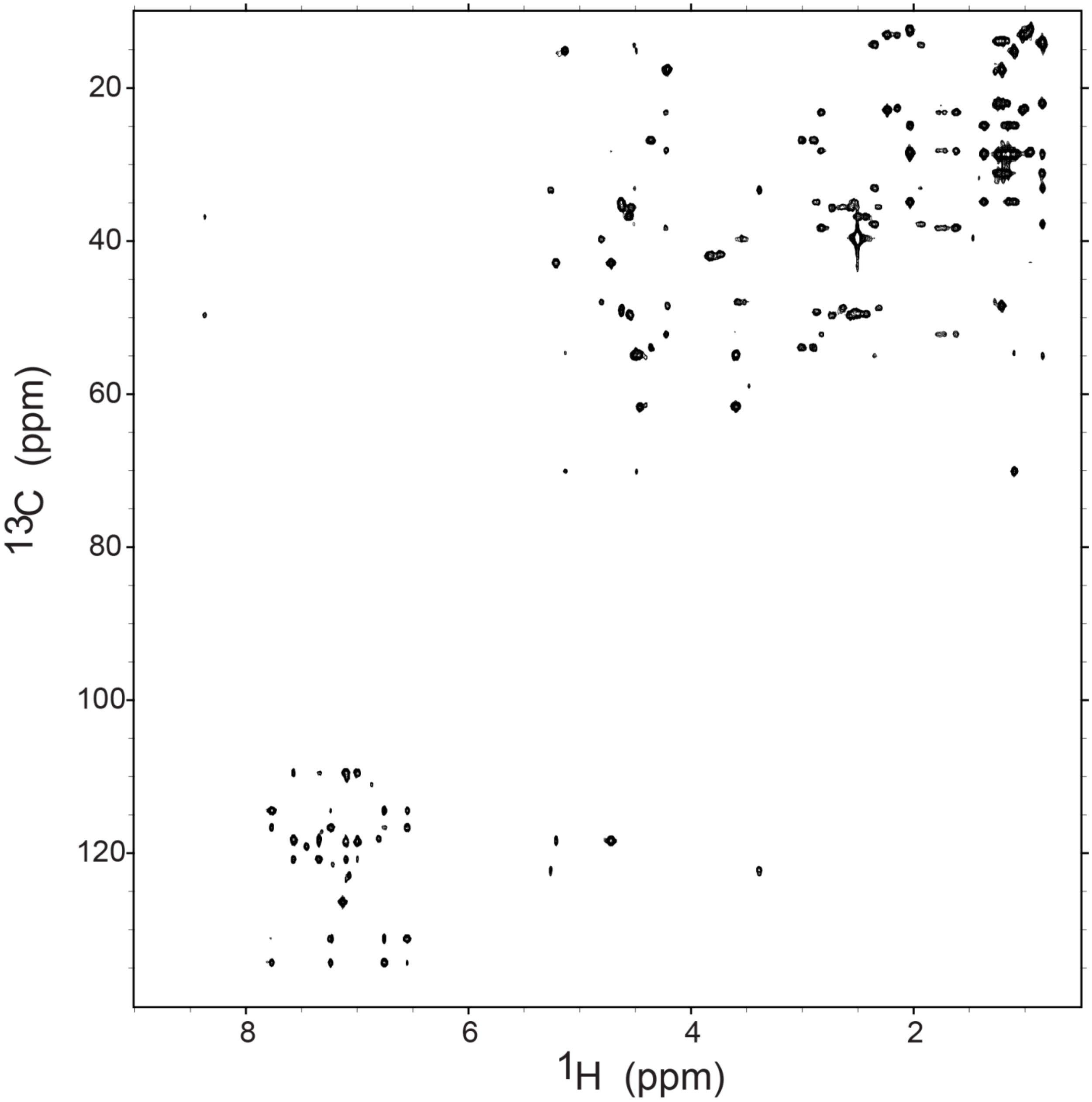

2D ^{1}H-^{13}C HSQC-TOCSY NMR spectrum (d_6-DMSO, 600 MHz of ^{1}H) of 90% pure***N1*-7-Dap (44)**

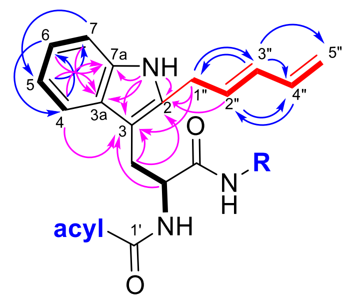

***C2*-15-Dap (45)**

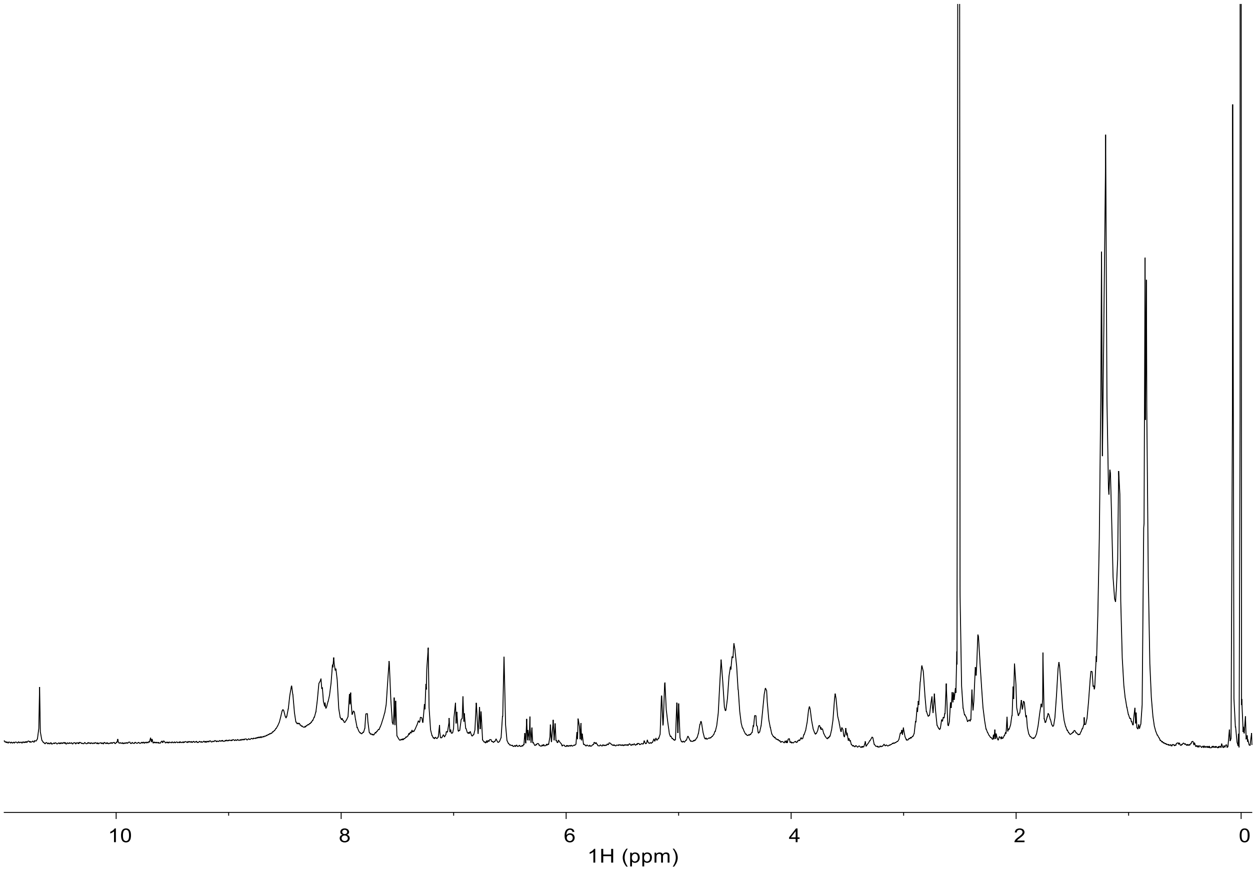

1D ^{1}H NMR spectrum (d$_6$-DMSO, 600 MHz of ^{1}H) of ***C2*-15-Dap (45)**

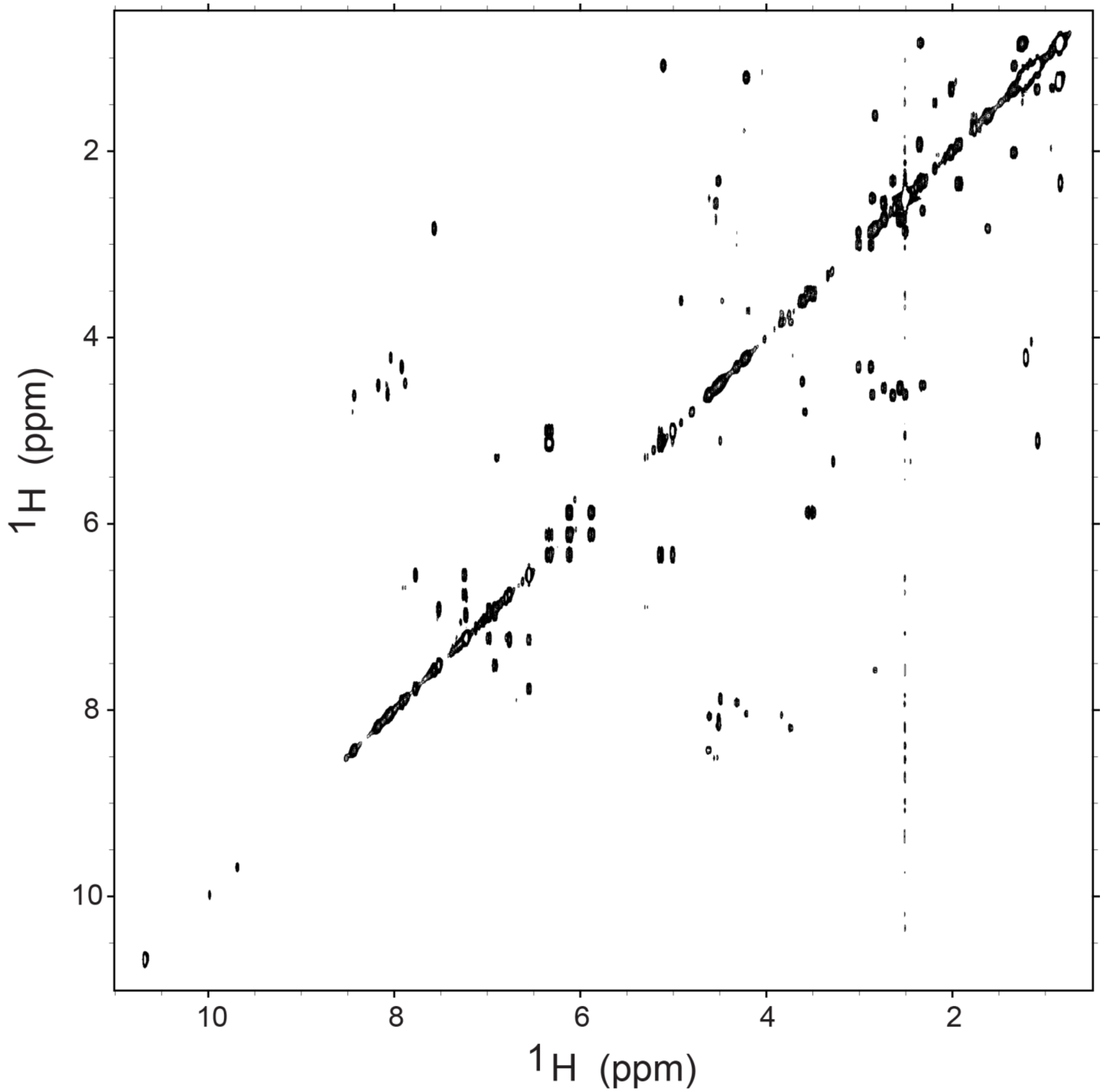

2D ^{1}H-^{1}H COSY NMR spectrum (d_6-DMSO, 600 MHz of ^{1}H) of ***C2*-15-Dap (45)**

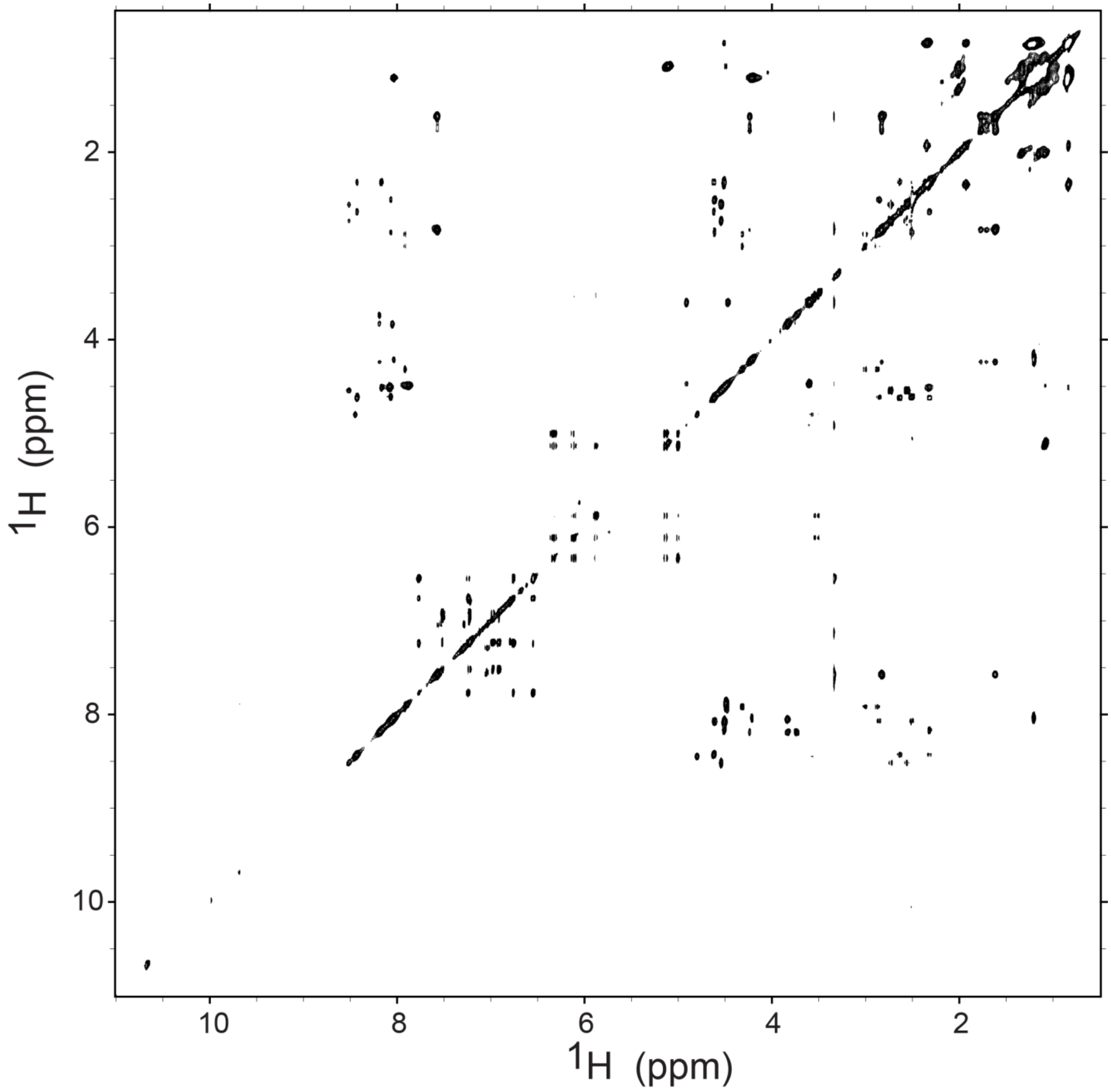

2D ^{1}H-^{1}H TOCSY NMR spectrum (d_6-DMSO, 600 MHz of ^{1}H) of ***C2*-15-Dap (45)**

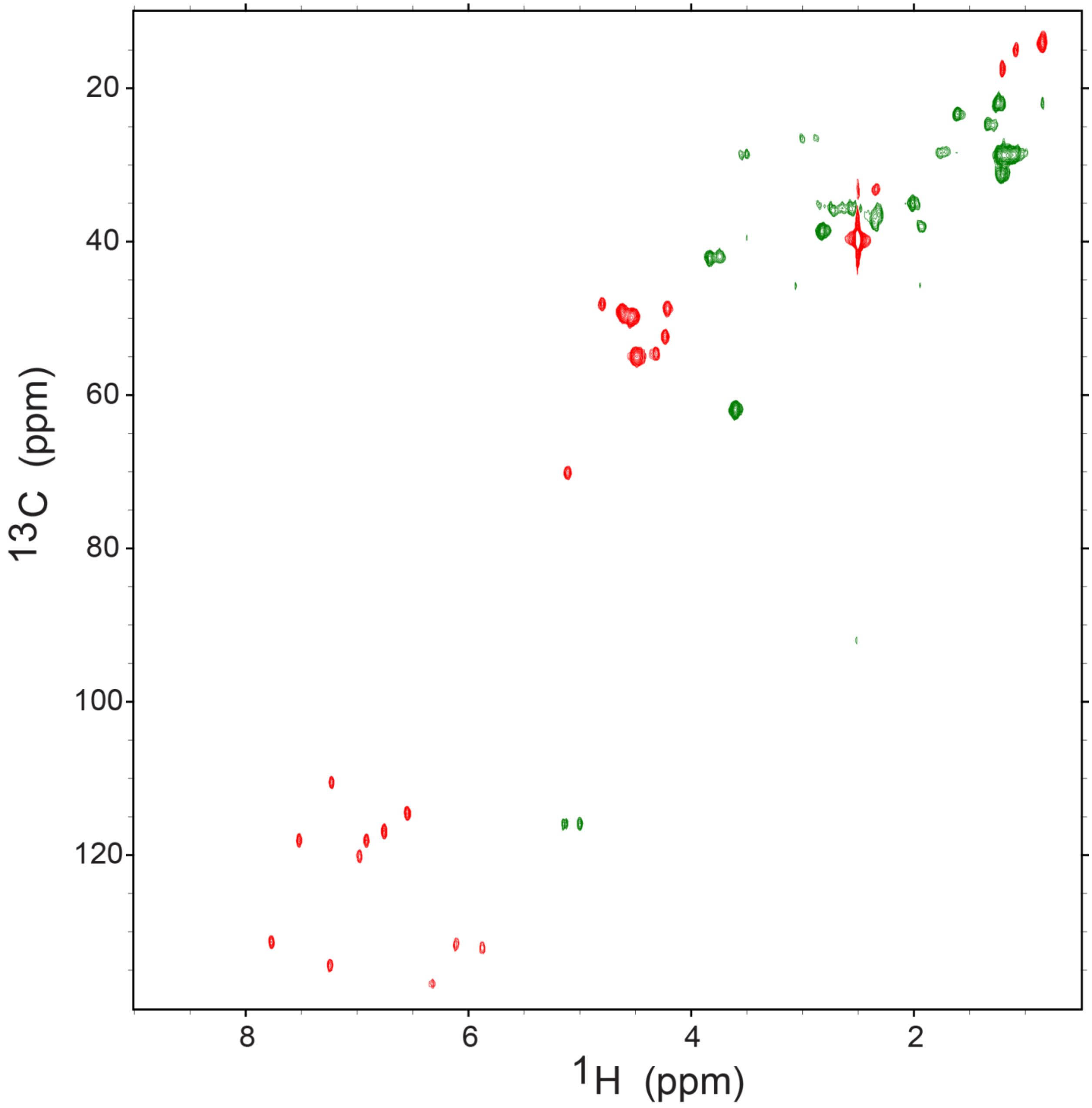

2D ^{1}H-^{13}C HSQC NMR spectrum (d_6-DMSO, 600 MHz of ^{1}H) of ***C2*-15-Dap (45)**

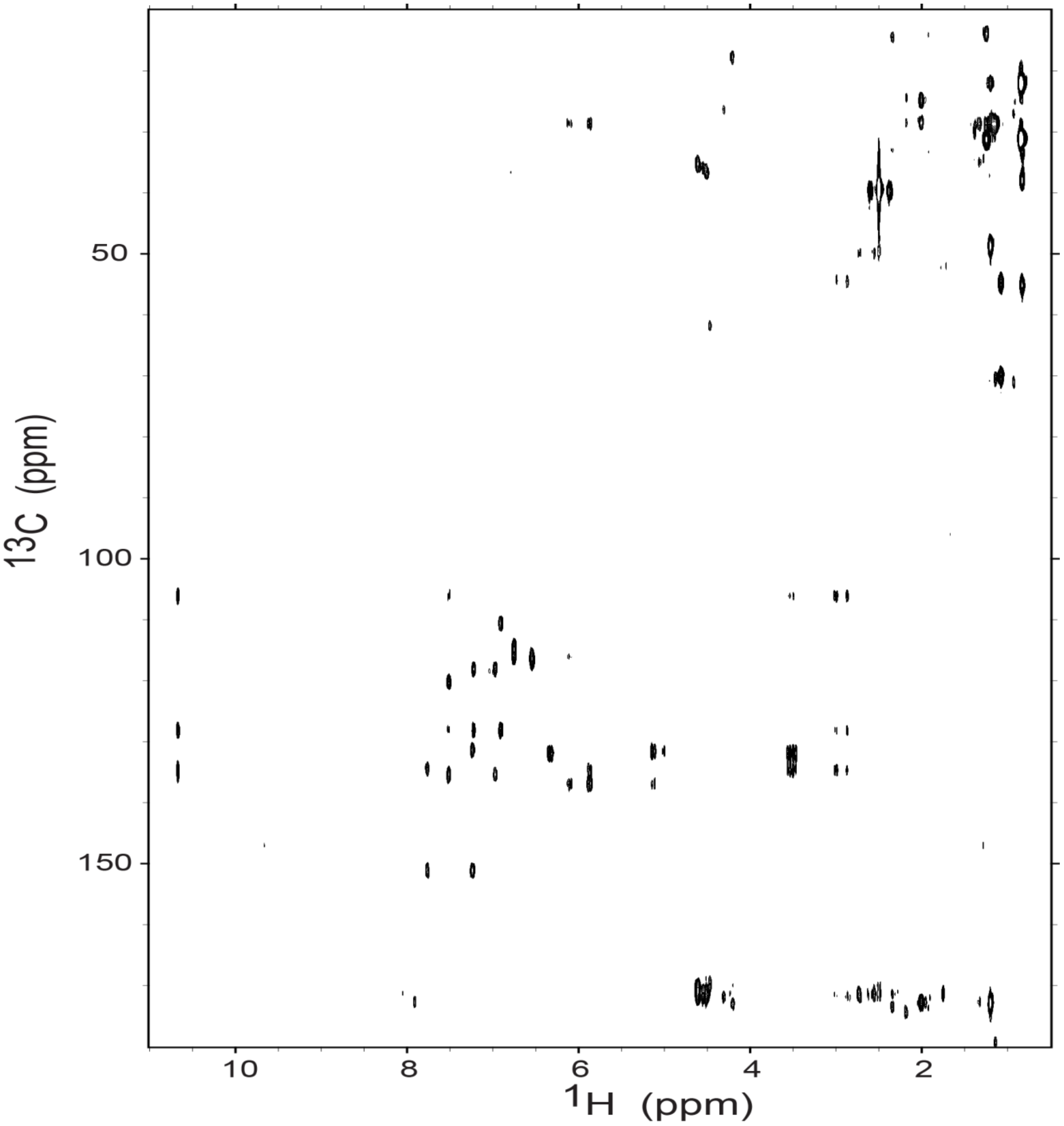

2D ^{1}H-^{13}C HMBC NMR spectrum (d_6-DMSO, 600 MHz of ^{1}H) of ***C2*-15-Dap (45)**

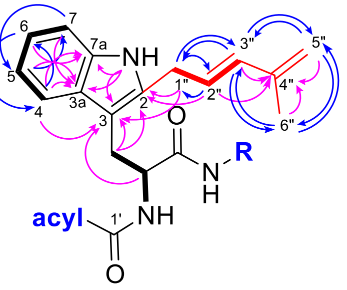

***C*2^R-16-Dap (46)**

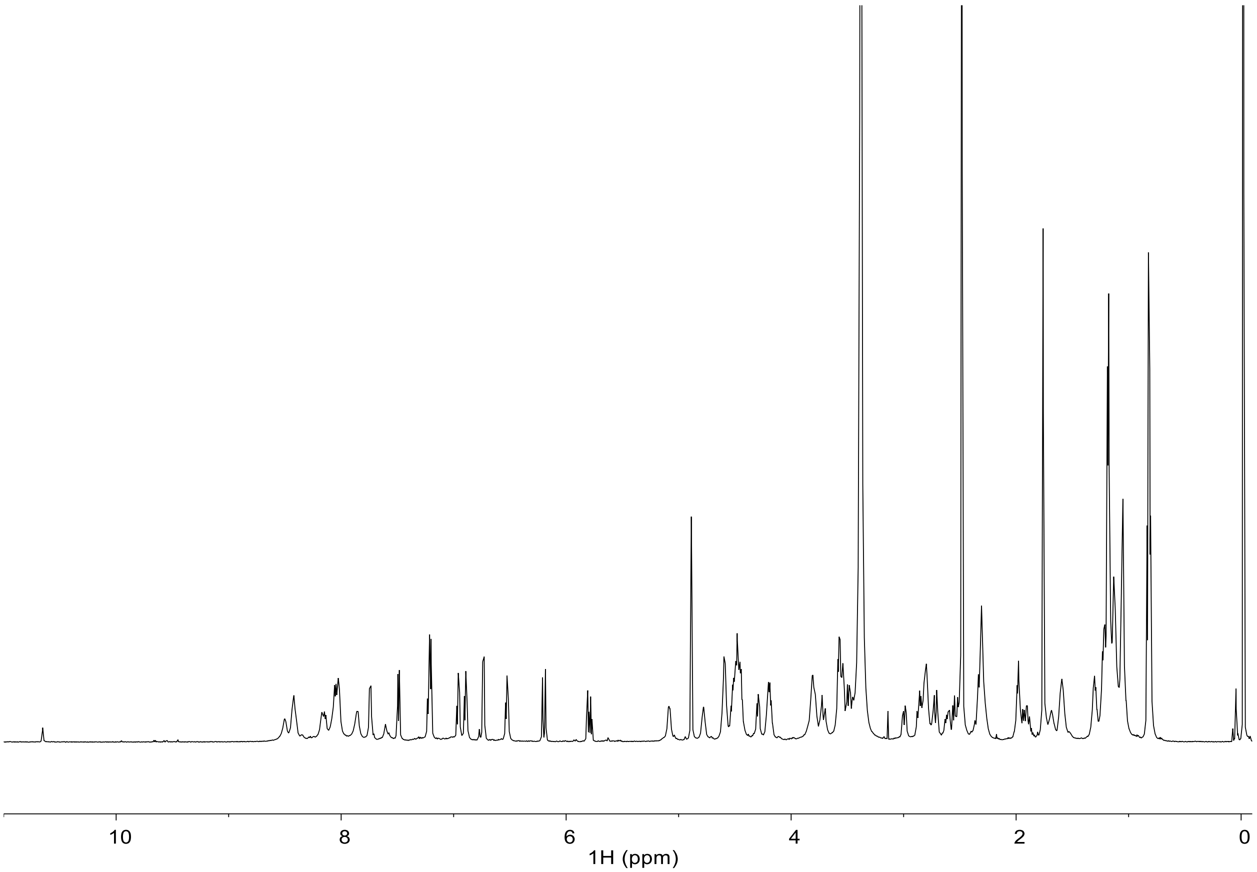

1D ^{1}H NMR spectrum (d_6-DMSO, 600 MHz of ^{1}H) of ***C2*^{R}-16-Dap (46)**

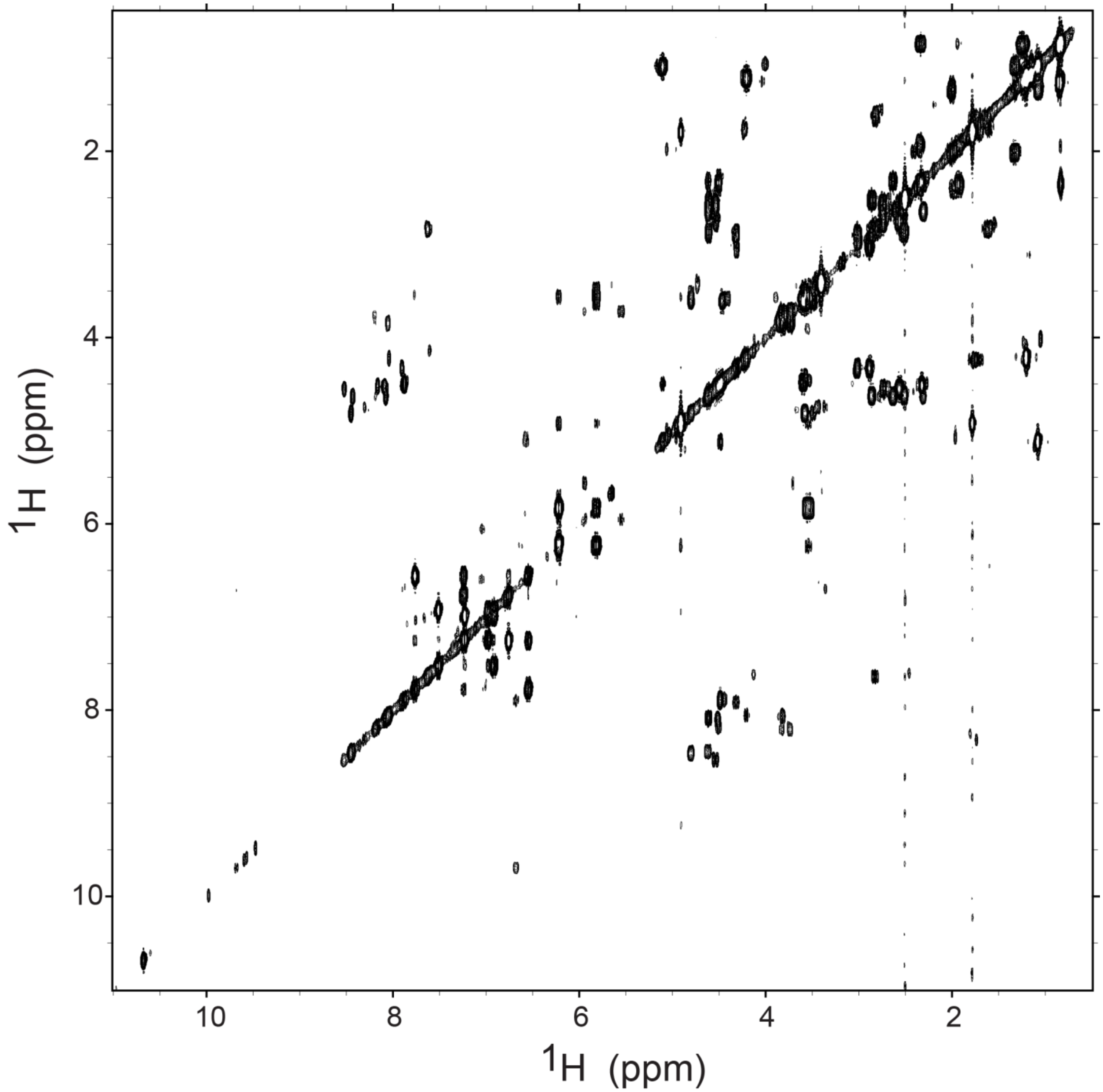

2D ^1H-^1H COSY NMR spectrum (d_6-DMSO, 600 MHz of ^1H) of ***C2*^{R}-16-Dap (46)**

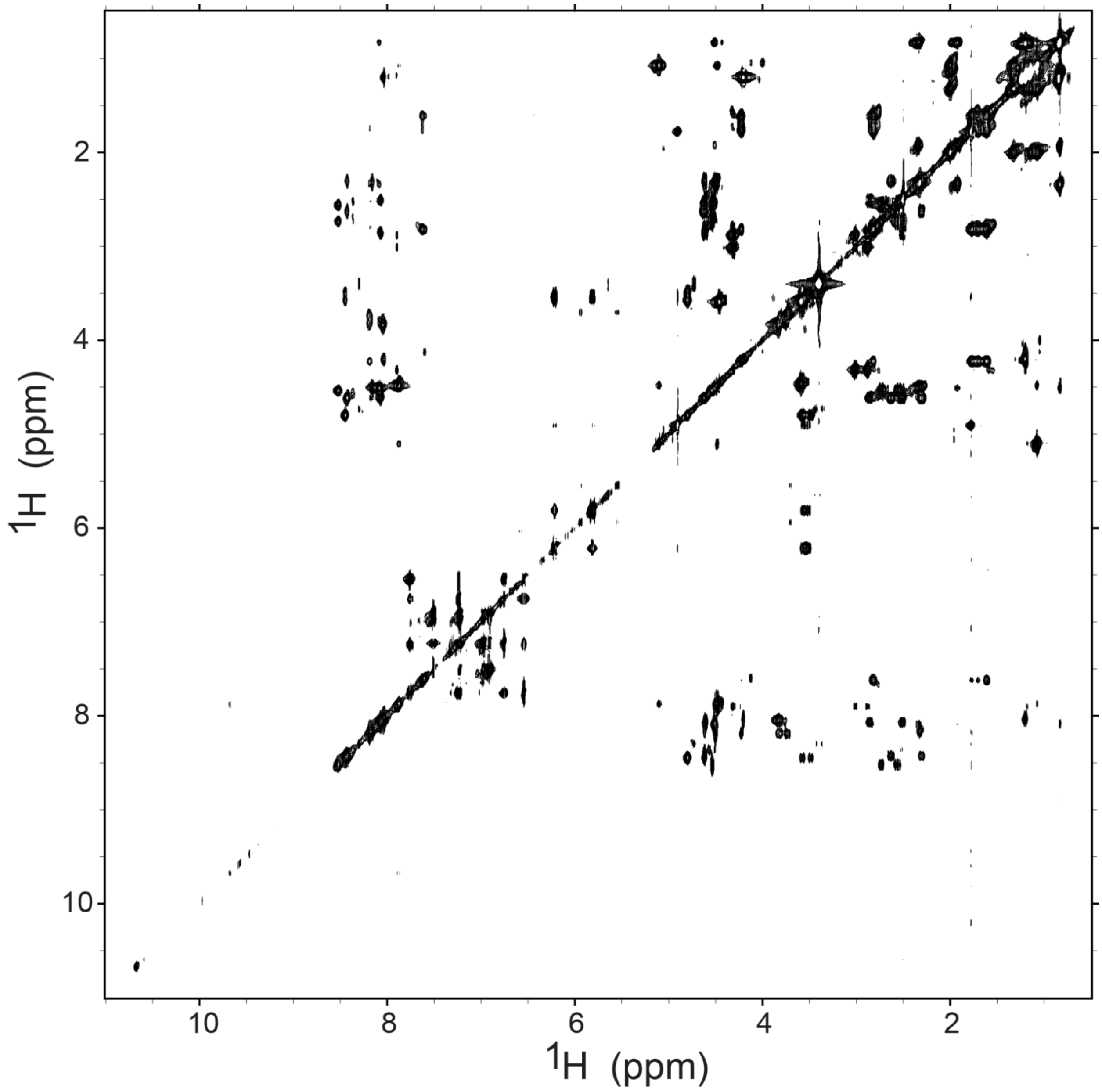

2D ^{1}H-^{1}H TOCSY NMR spectrum (d_6-DMSO, 600 MHz of ^{1}H) of ***C2*^{R}-16-Dap (46)**

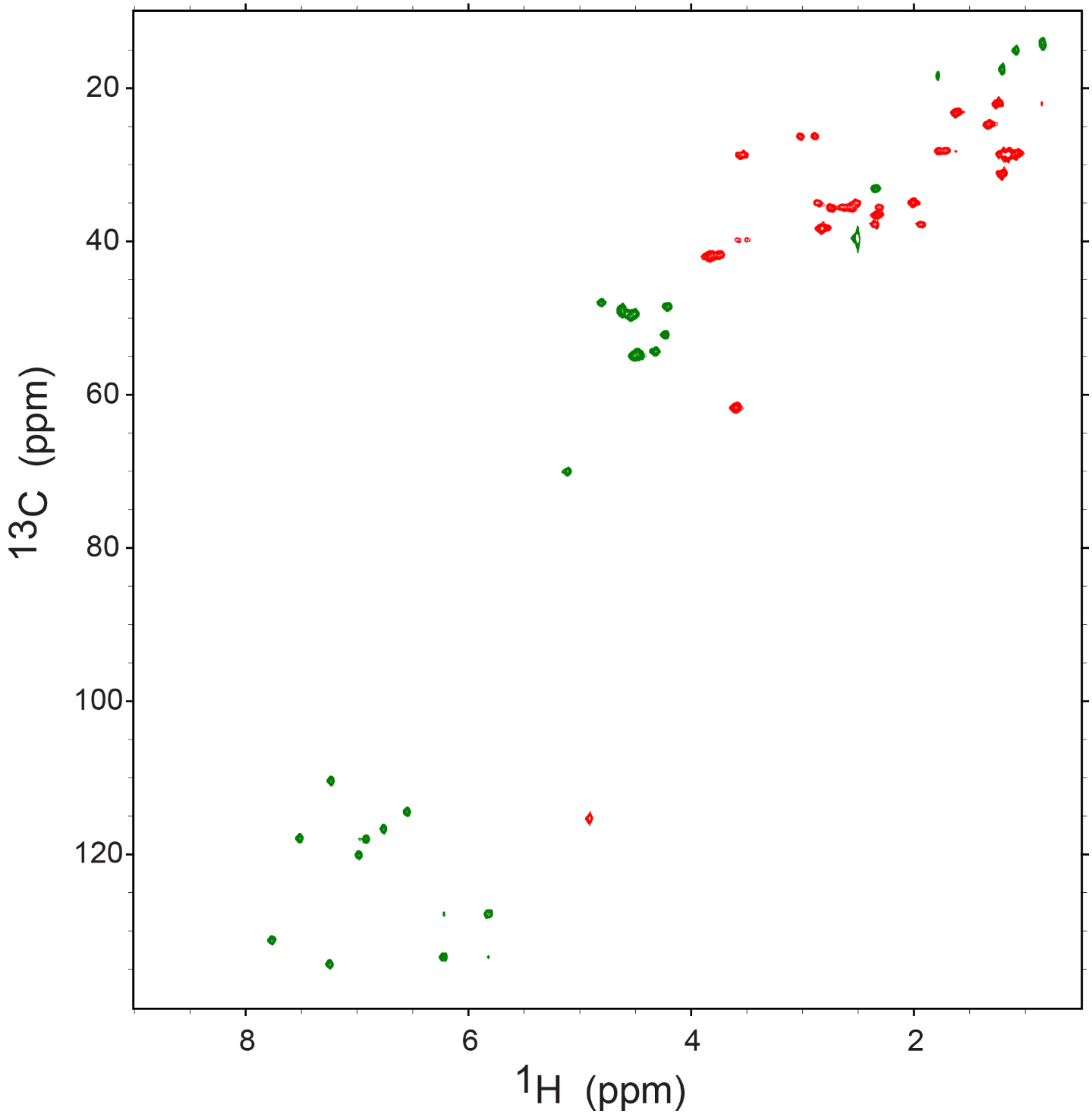

2D ^{1}H-^{13}C HSQC NMR spectrum (d_6-DMSO, 600 MHz of ^{1}H) of ***C2*^{R}-16-Dap (46)**

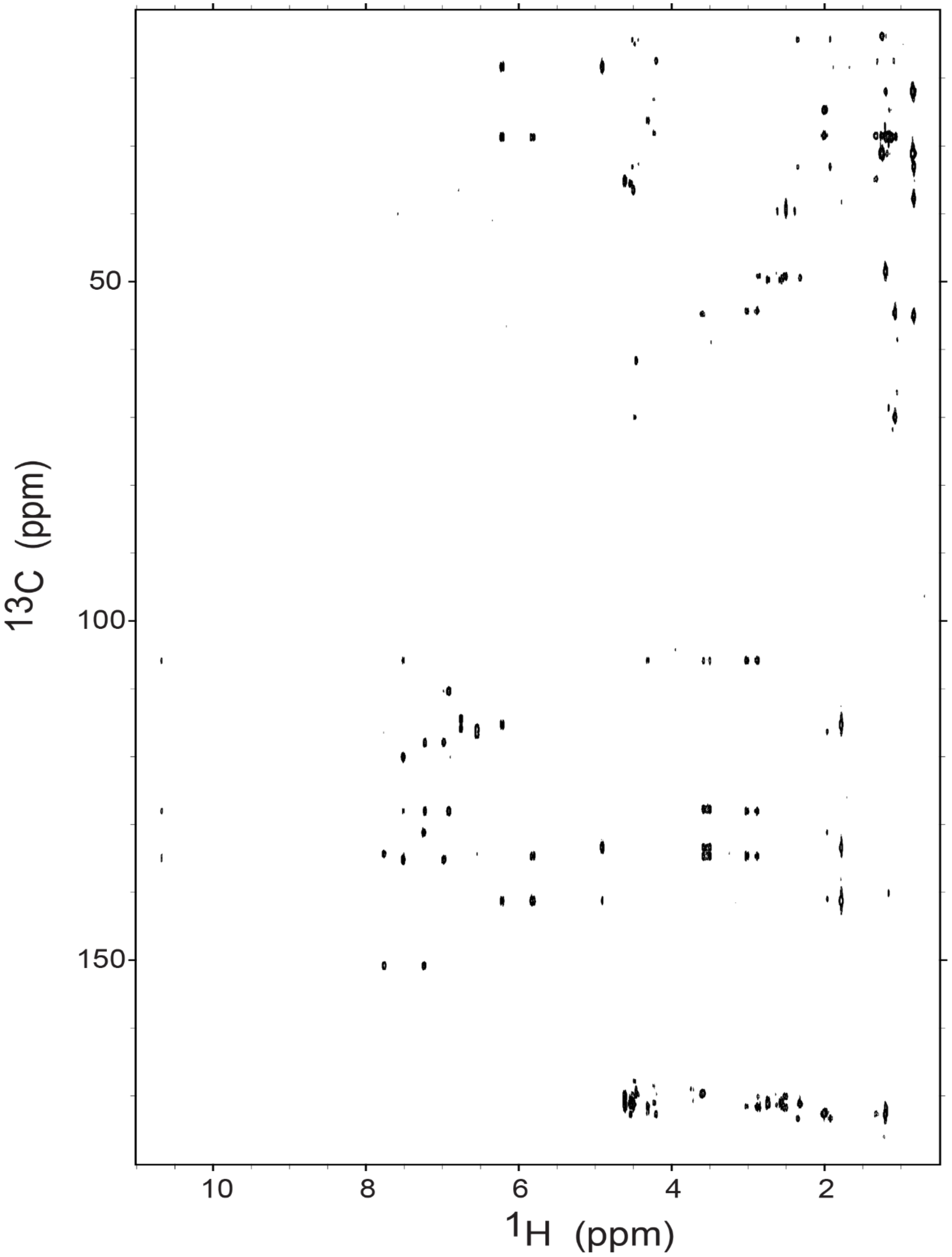

2D ^{1}H-^{13}C HMBC NMR spectrum (d_6-DMSO, 600 MHz of ^{1}H) of ***C2*^R^-16-Dap (46)**

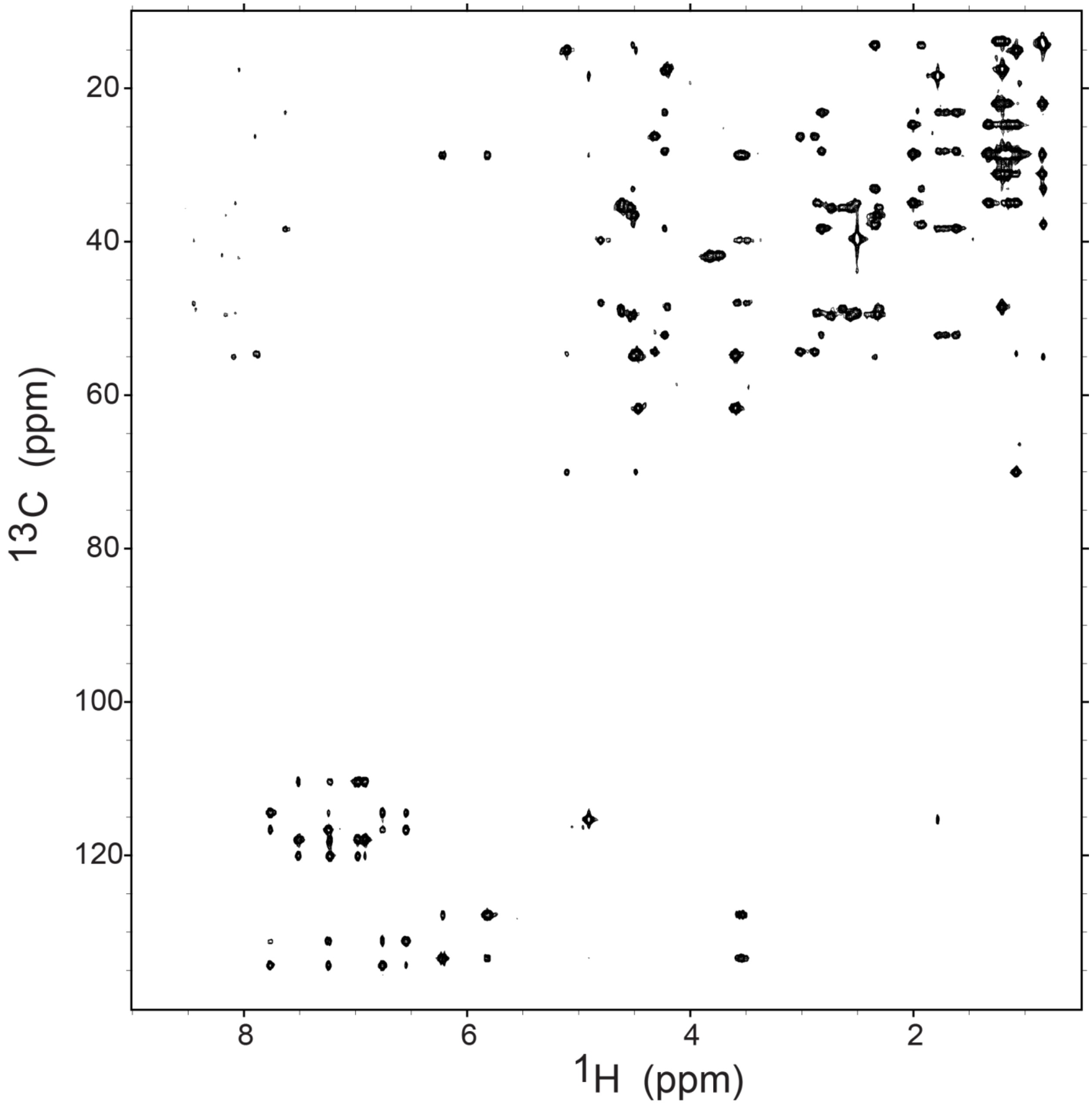

2D ^{1}H-^{13}C HSQC-TOCSY NMR spectrum (d_6-DMSO, 600 MHz of ^{1}H) of ***C2*^R^-16-Dap (46)**

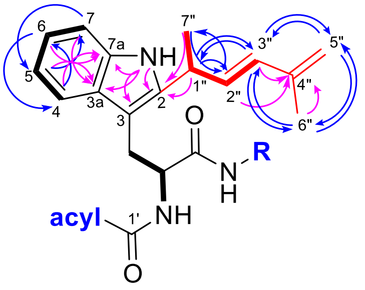

***C2*[R]-18-Dap (47)**

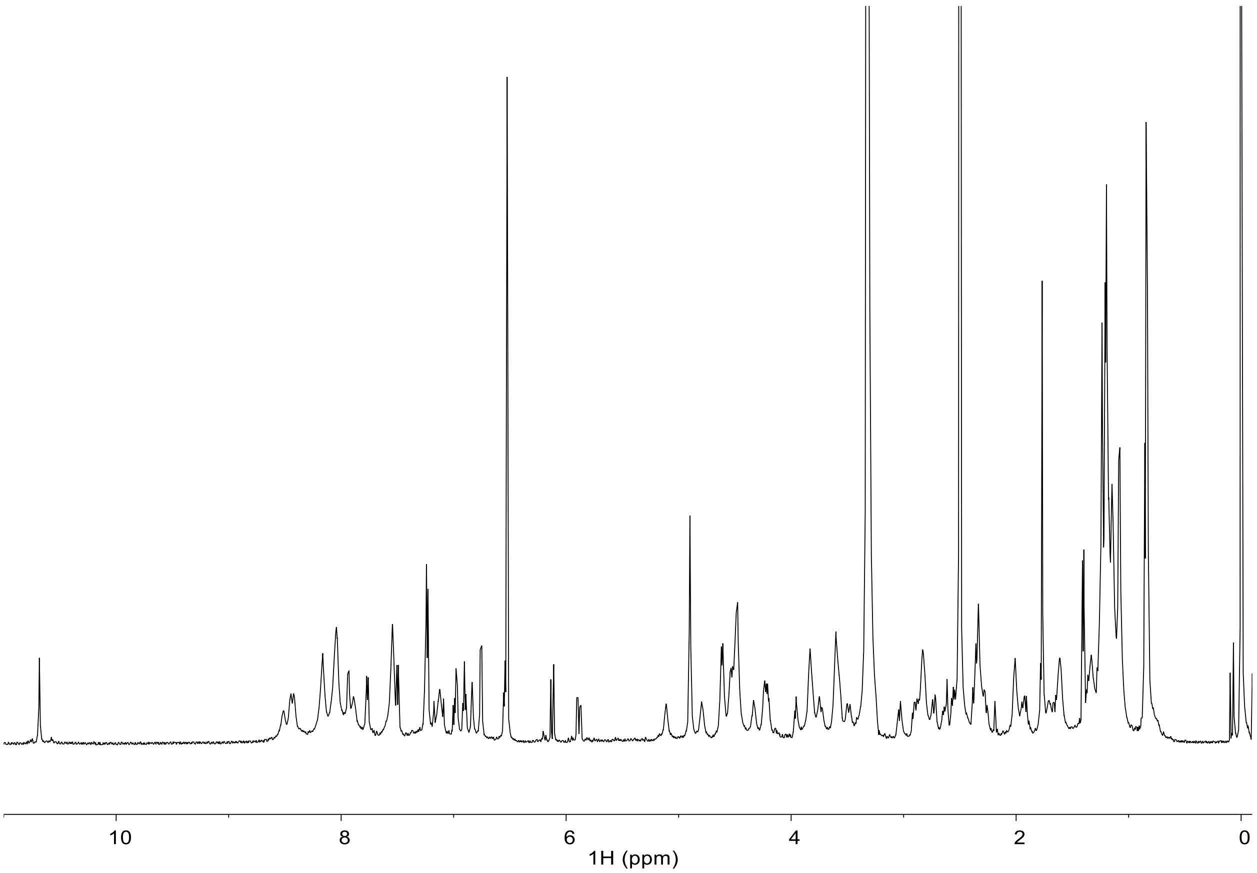

1D ^{1}H NMR spectrum (d$_6$-DMSO, 600 MHz of ^{1}H) of ***C2*[R]-18-Dap (47)**

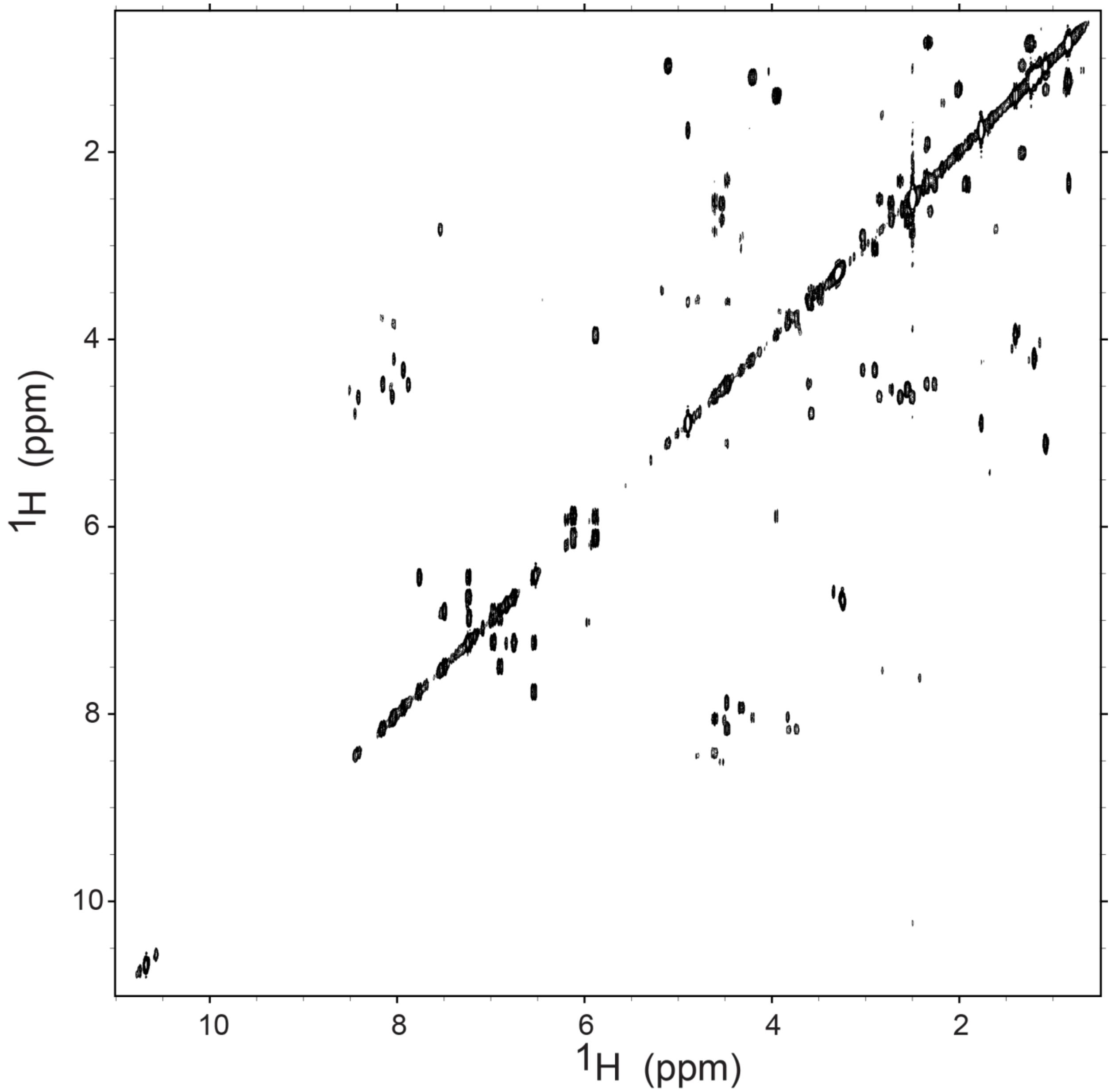

2D ^{1}H-^{1}H COSY NMR spectrum (d_6-DMSO, 600 MHz of ^{1}H) of ***C2*^R^-18-Dap (47)**

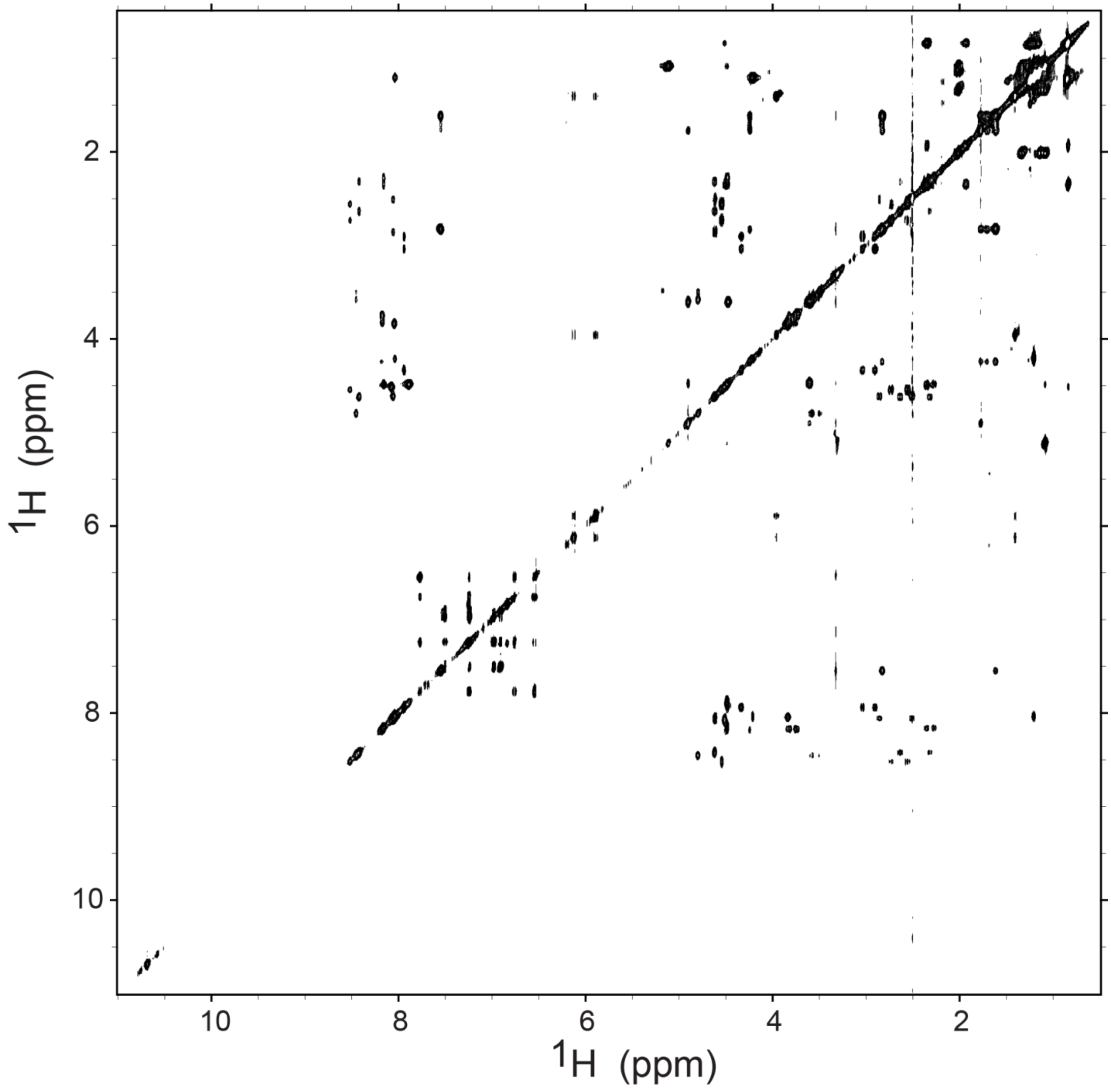

2D ^{1}H-^{1}H TOCSY NMR spectrum (d_6-DMSO, 600 MHz of ^{1}H) of ***C2*^R-18-Dap (47)**

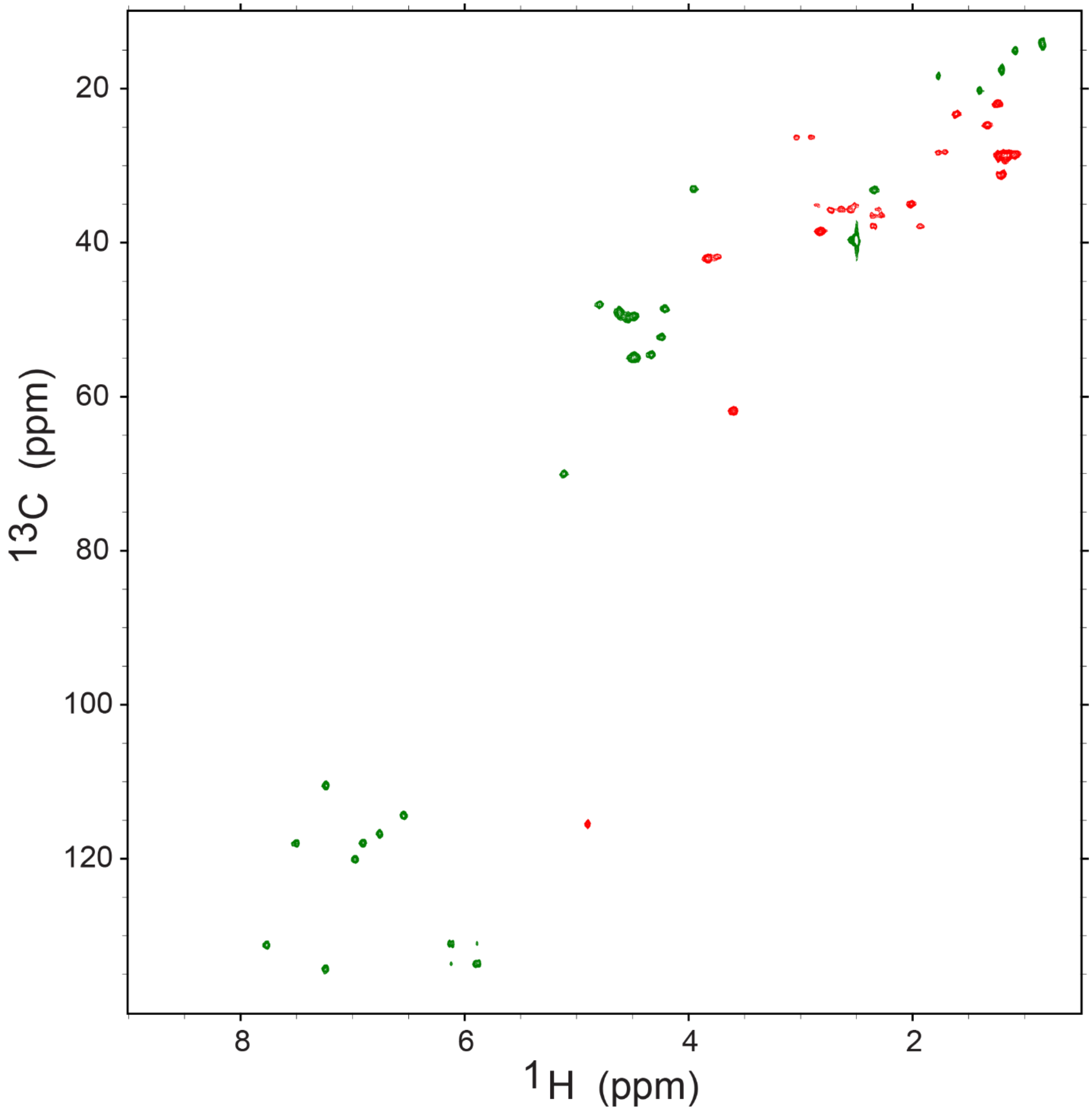

2D ^{1}H-^{13}C HSQC NMR spectrum (d_6-DMSO, 600 MHz of ^{1}H) of ***C2*^{R}-18-Dap (47)**

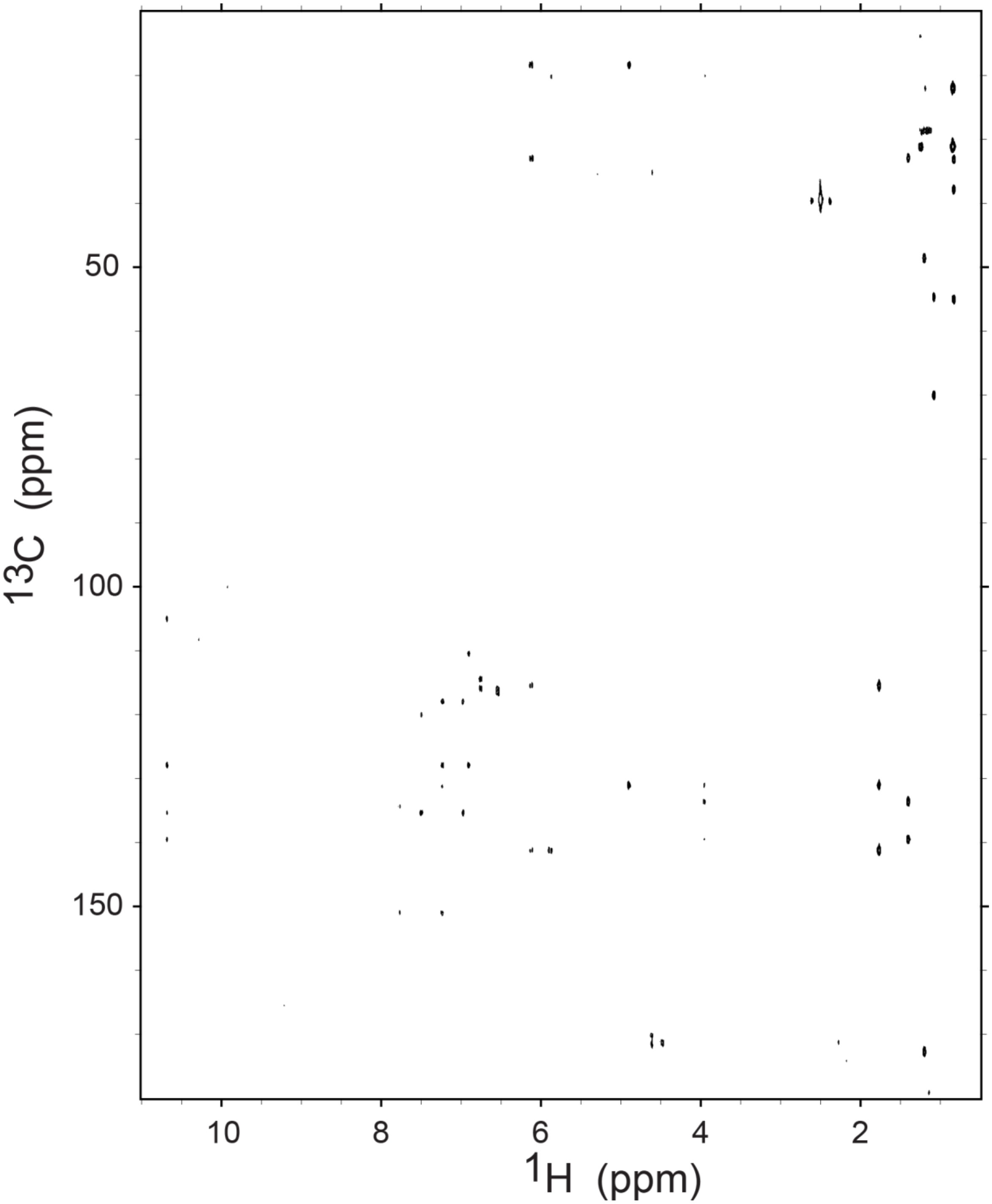

2D ^{1}H-^{13}C HMBC NMR spectrum (d_6-DMSO, 600 MHz of ^{1}H) of ***C2*^R^-18-Dap (47)**

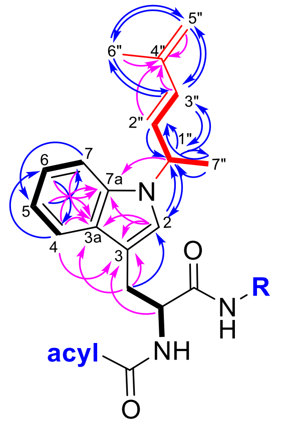

***N1*^R^-18-Dap (48)**

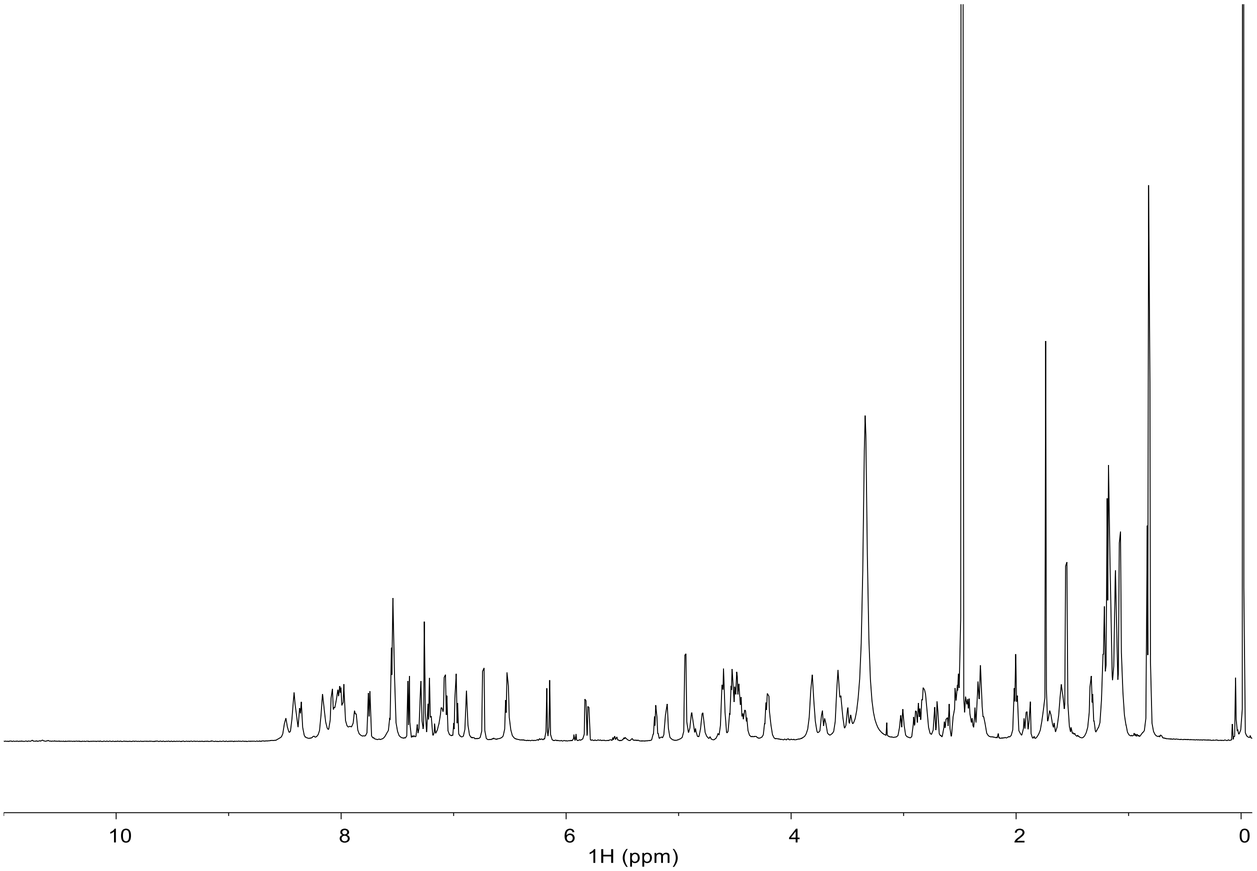

1D ^{1}H NMR spectrum (d$_6$-DMSO, 600 MHz of ^{1}H) of ***N1*$^{\mathbf{R}}$-18-Dap (48)**

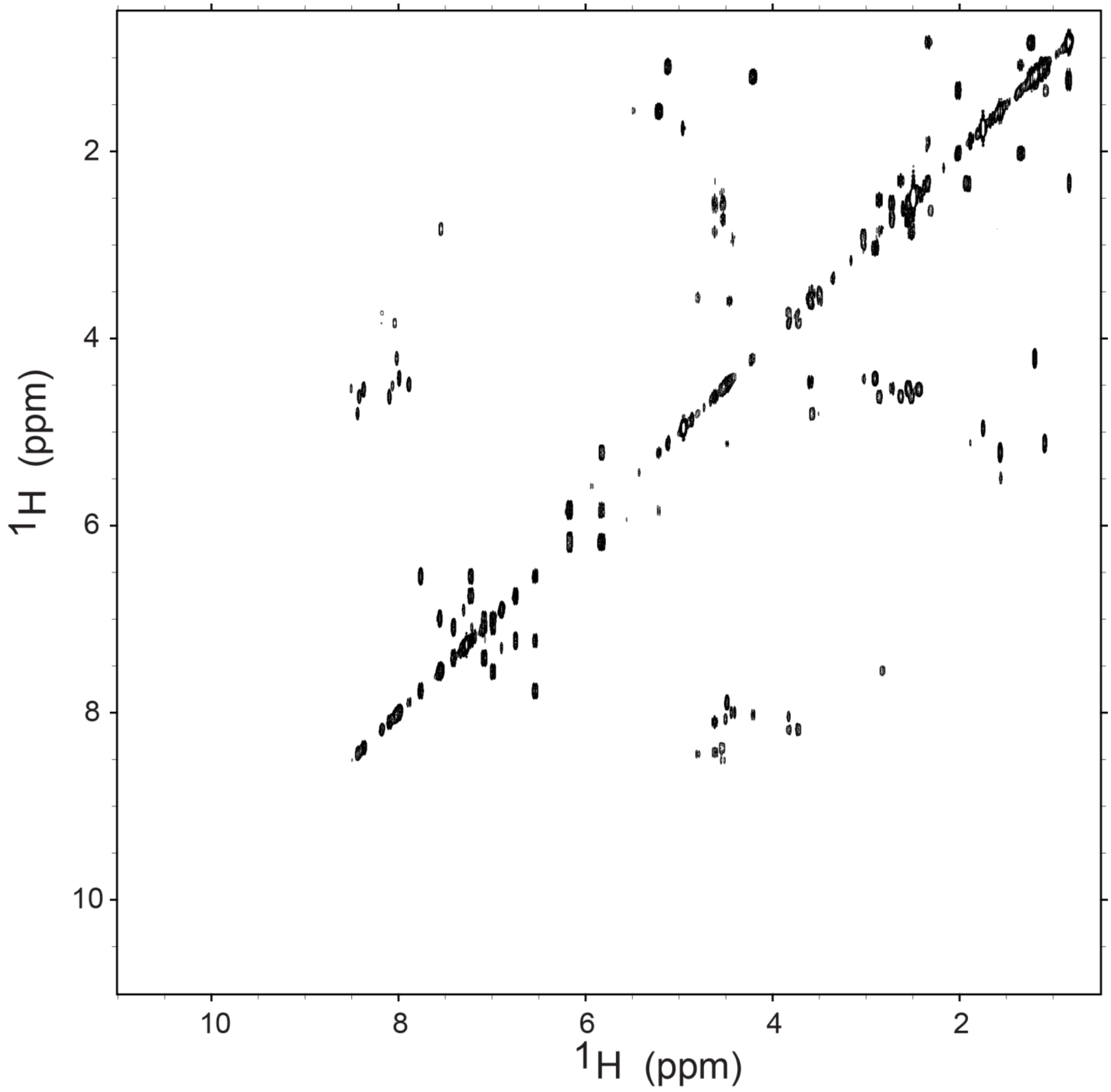

2D ^{1}H-^{1}H COSY NMR spectrum (d_6-DMSO, 600 MHz of ^{1}H) of ***N1*^{R}-18-Dap (48)**

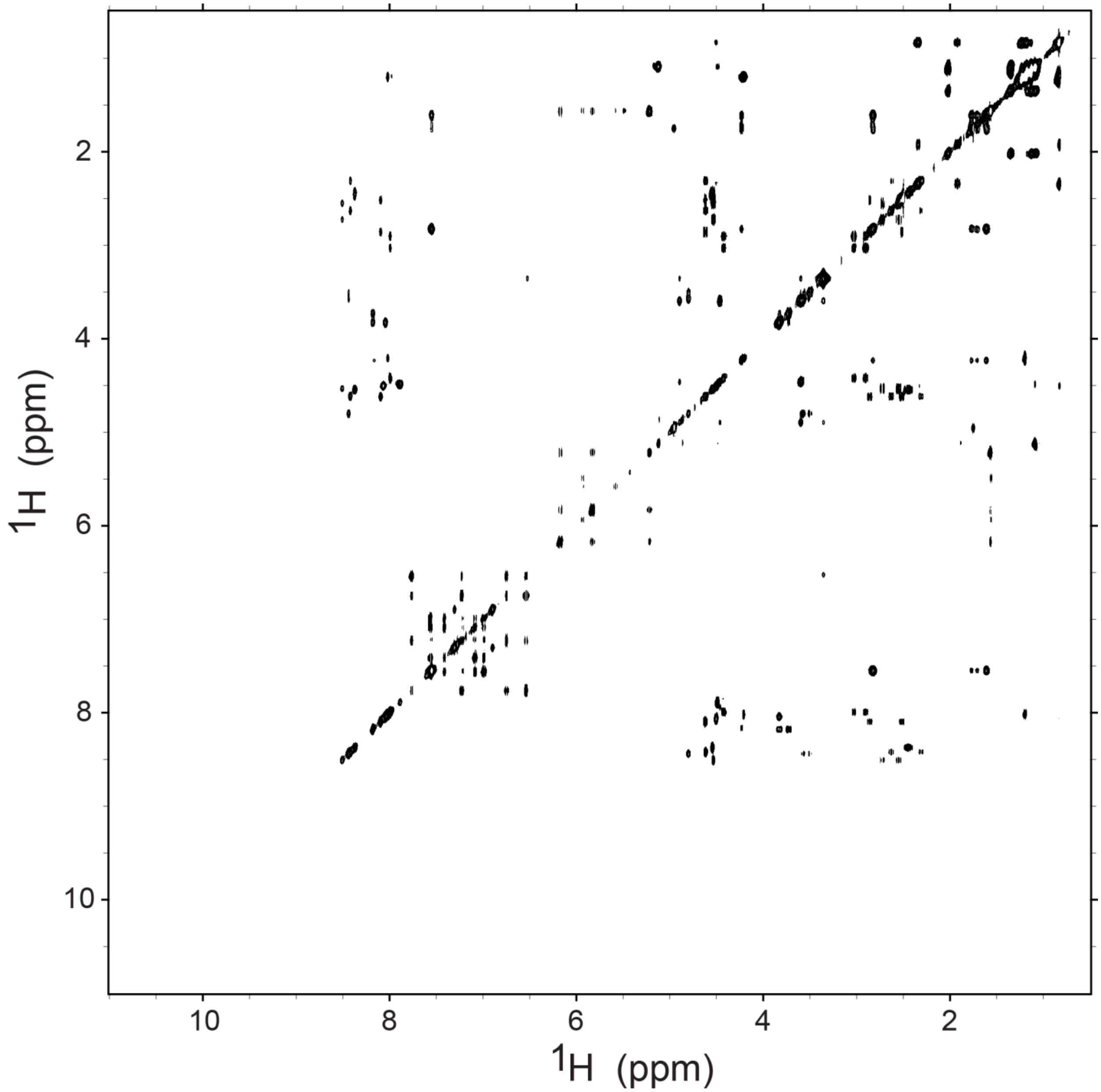

2D ^{1}H-^{1}H TOCSY NMR spectrum (d$_6$-DMSO, 600 MHz of ^{1}H) of ***N1***

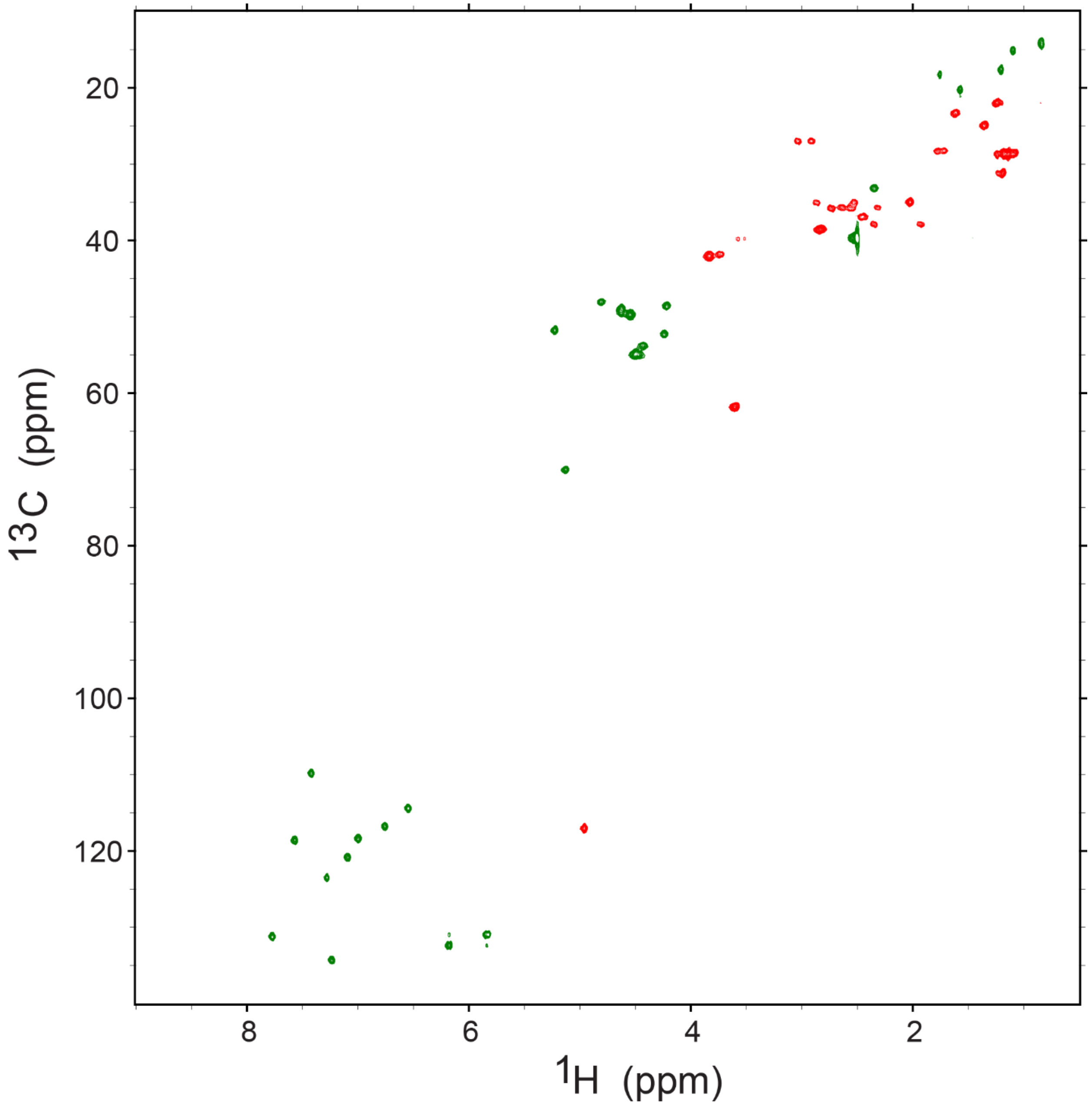

2D ^{1}H-^{13}C HSQC NMR spectrum (d_6-DMSO, 600 MHz of ^{1}H) of ***N1*^{R}-18-Dap (48)**

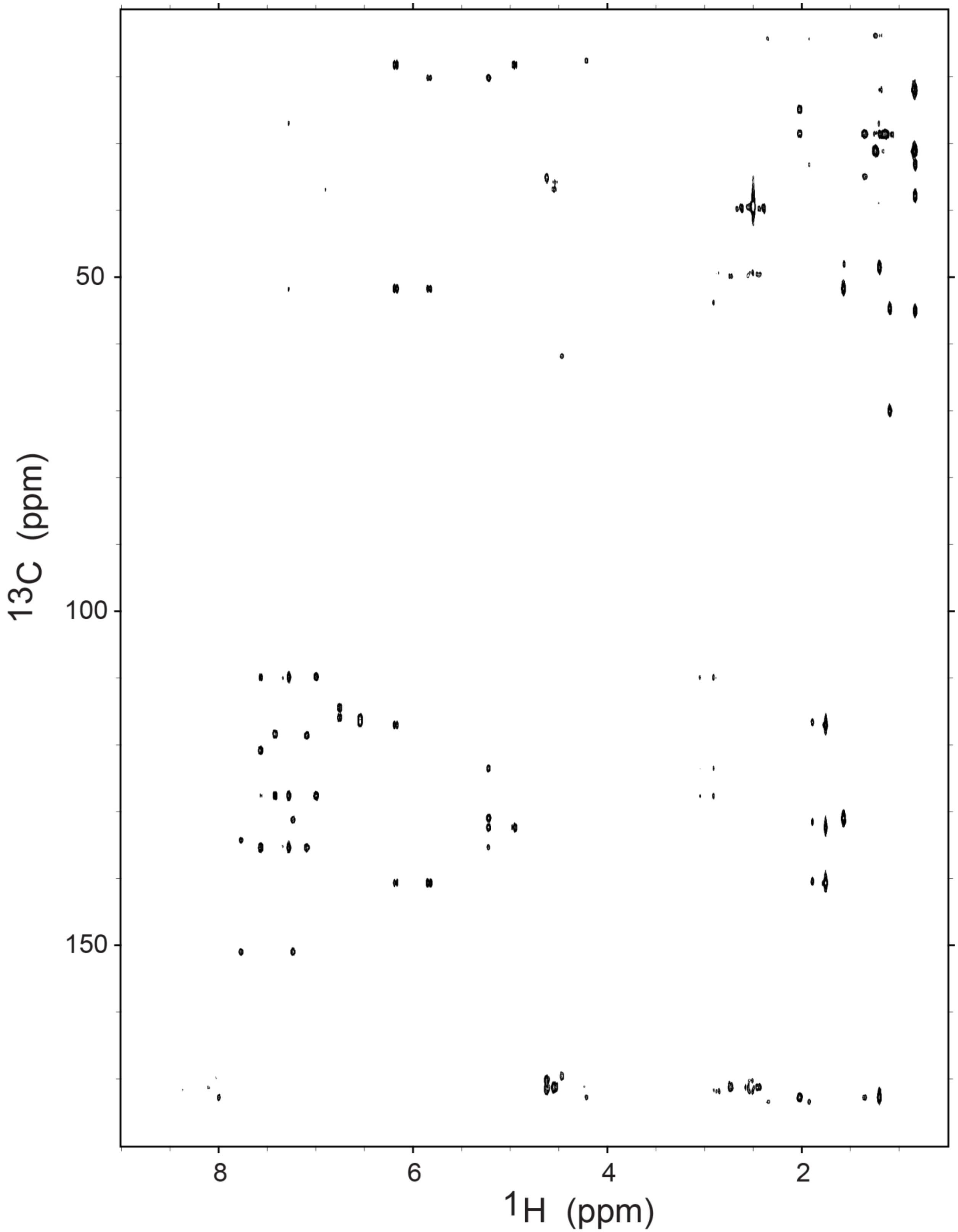

2D ^{1}H-^{13}C HMBC NMR spectrum (d_6-DMSO, 600 MHz of ^{1}H) of ***N1*^R^-18-Dap (48)**

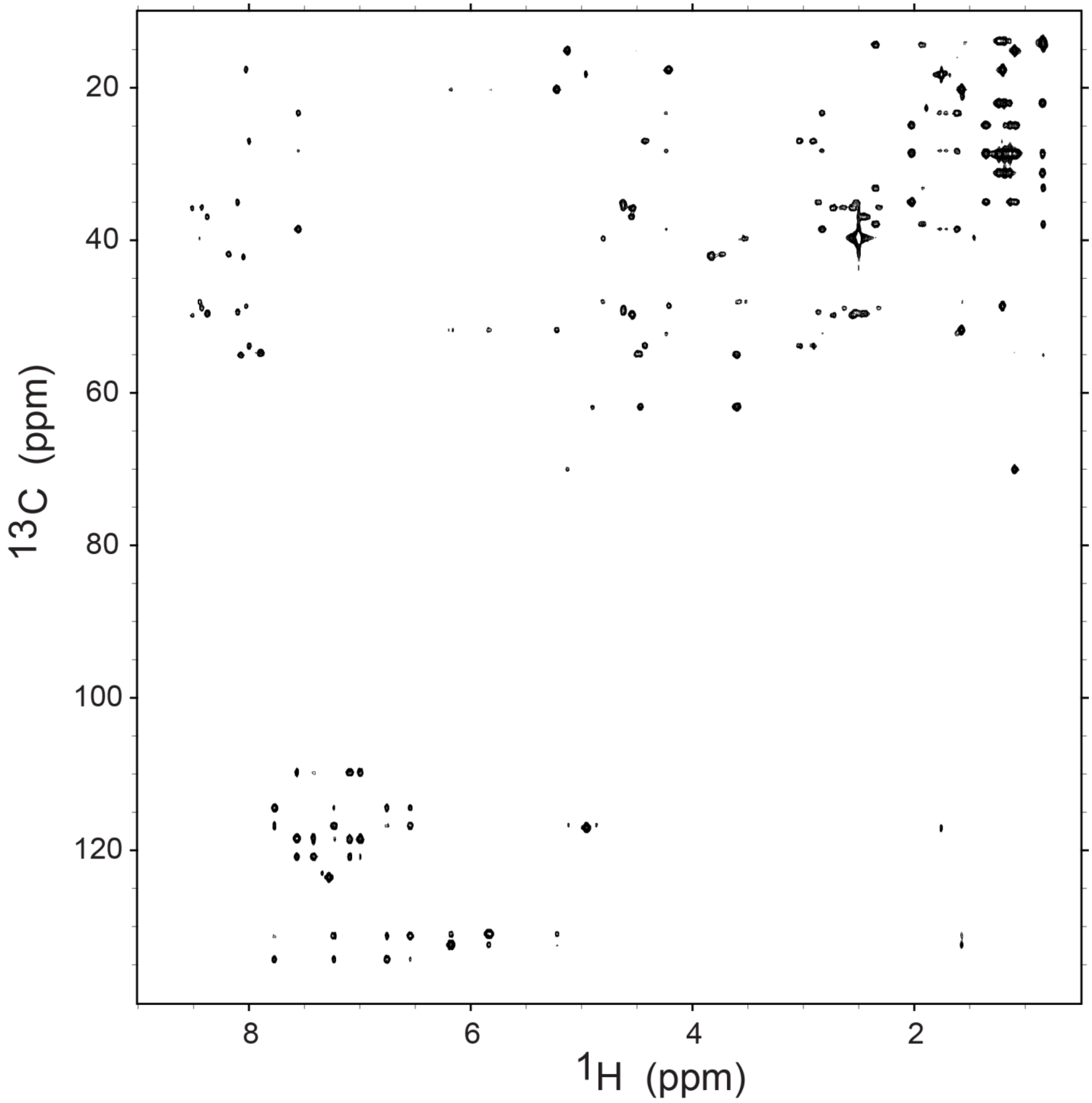

2D ^{1}H-^{13}C HSQC-TOCSY NMR spectrum (d_6-DMSO, 600 MHz of ^{1}H) of ***N1*^R^-18-Dap (48)**

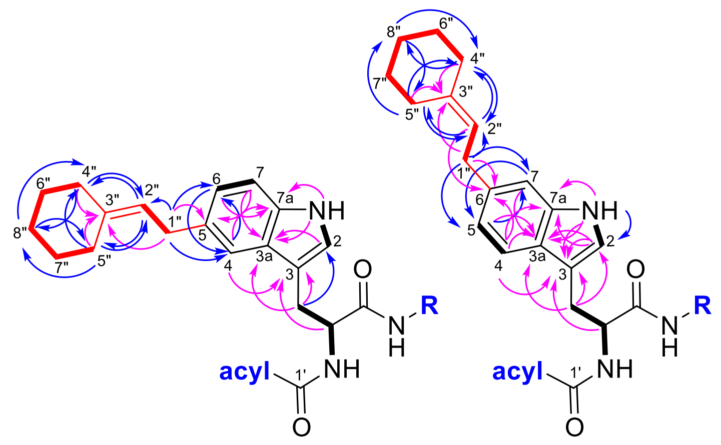

***C5*-19-Dap**
15%

***C6*-19-Dap**
85%

(49)

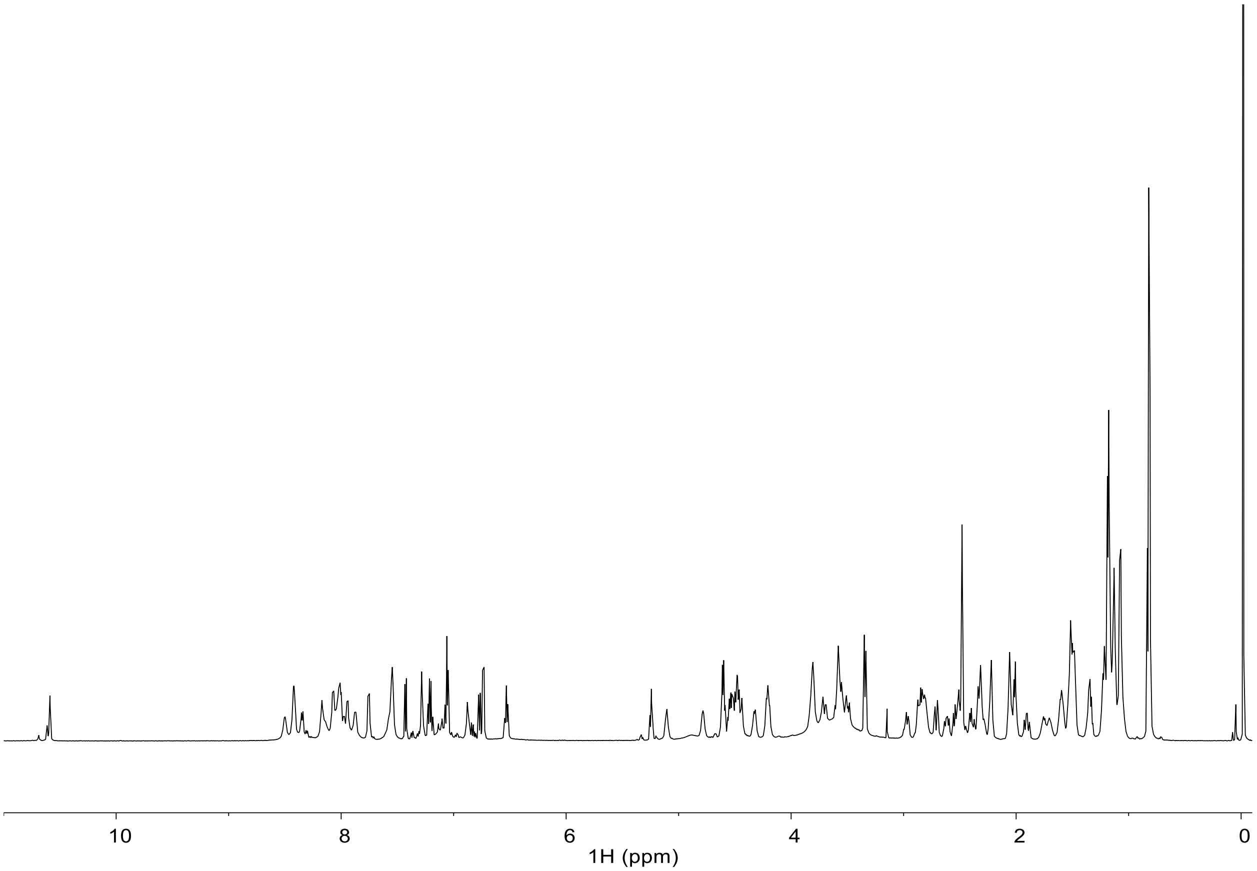

1D ^{1}H NMR spectrum (d$_6$-DMSO, 600 MHz of ^{1}H) of
0.15:0.85 mixture of ***C5*-** and ***C6*-19-Dap (49)**

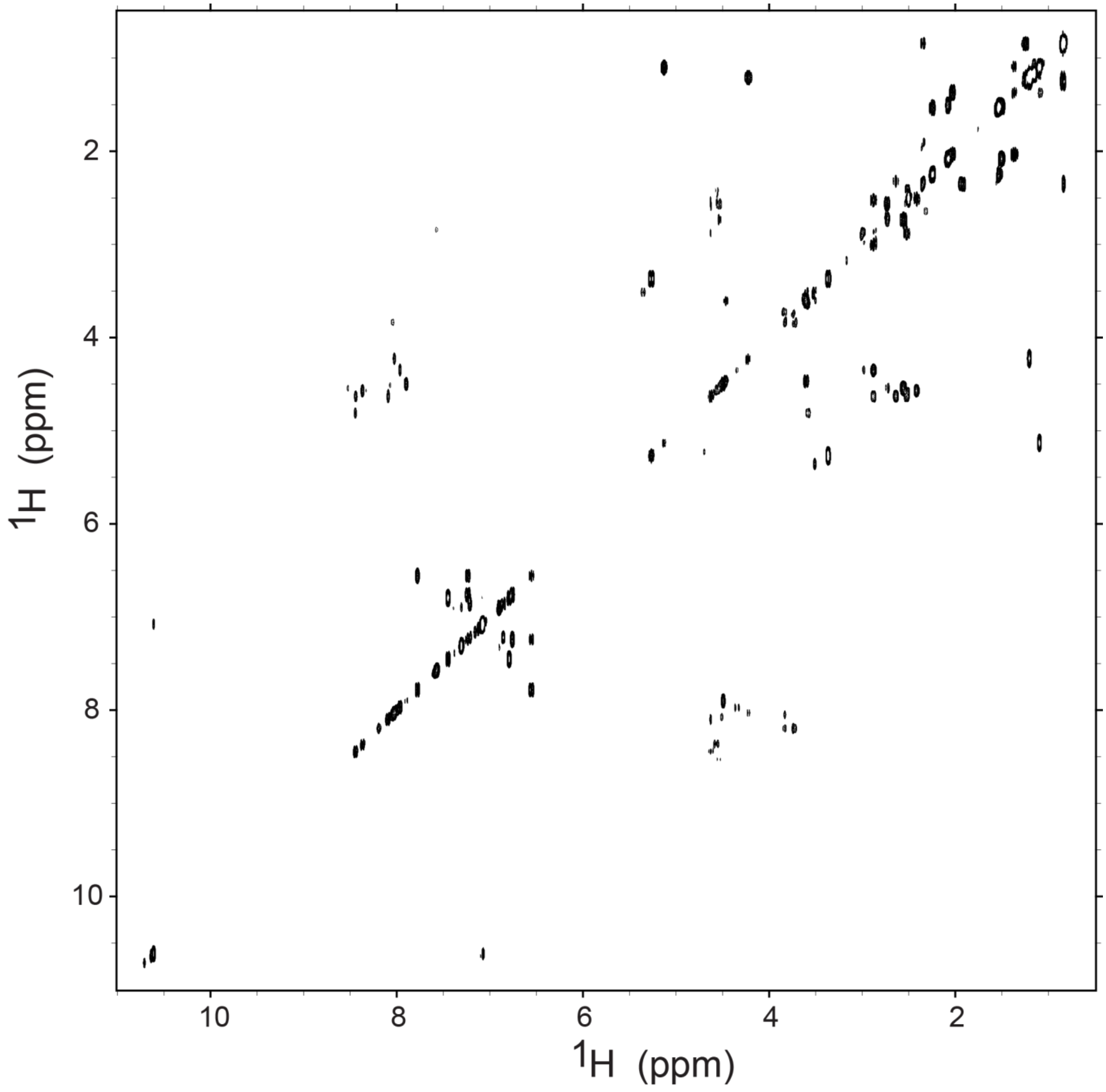

2D ^{1}H-^{1}H COSY NMR spectrum (d_6-DMSO, 600 MHz of ^{1}H) of

0.15:0.85 mixture of ***C5*-** and ***C6*-19-Dap (49)**

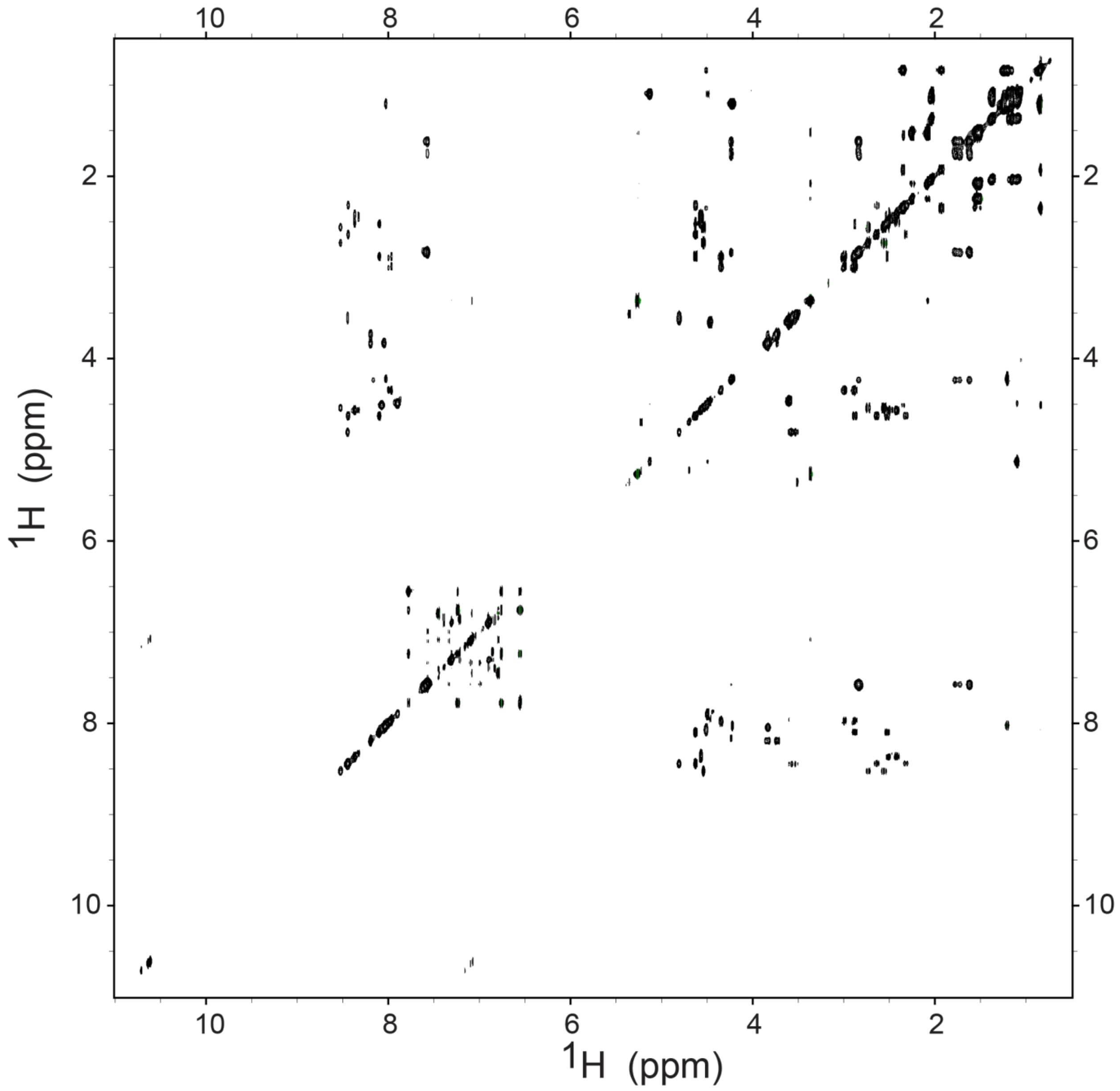

2D ^{1}H-^{1}H TOCSY NMR spectrum (d_6-DMSO, 600 MHz of ^{1}H) of

0.15:0.85 mixture of ***C5*-** and ***C6*-19-Dap (49)**

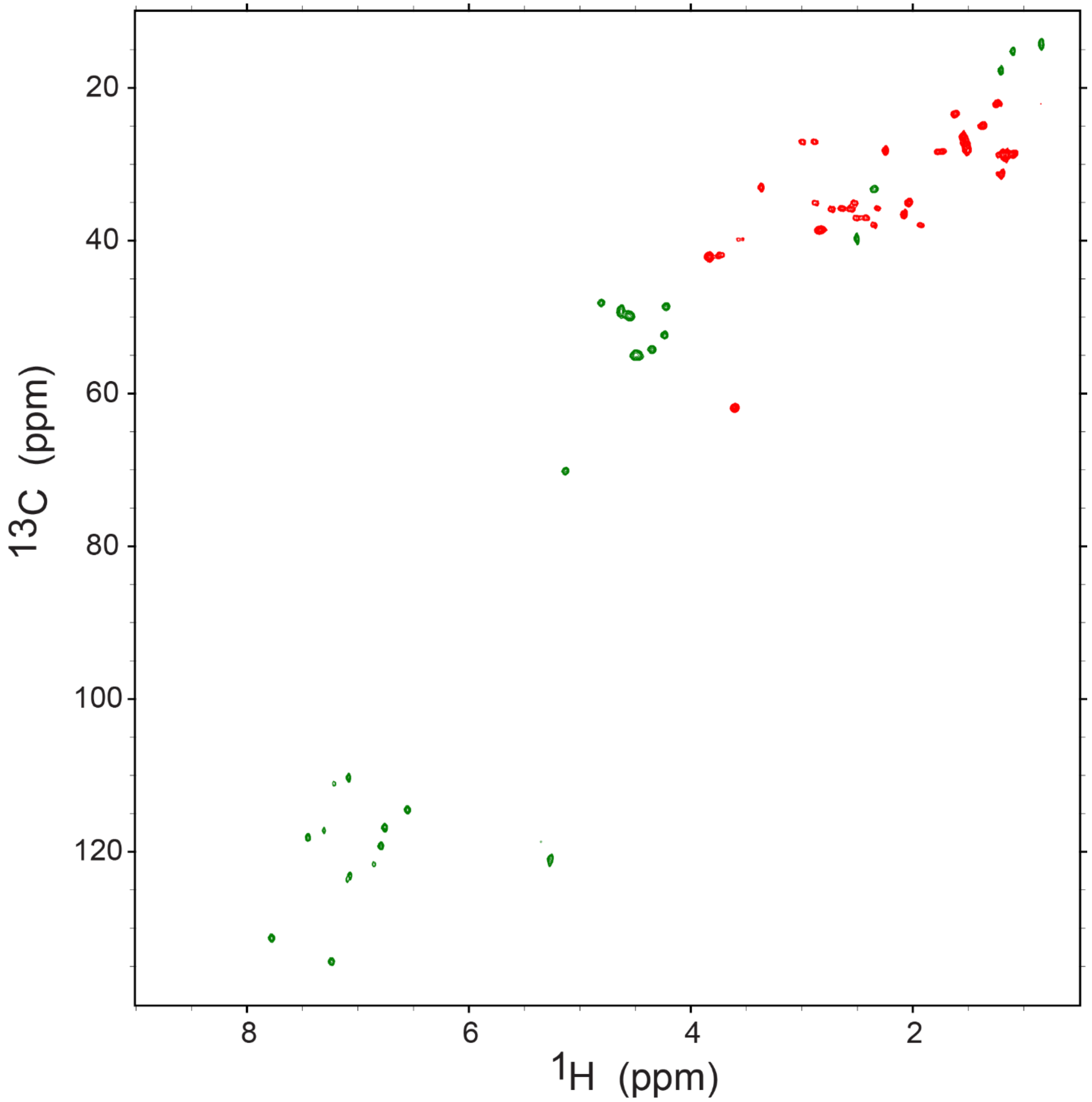

2D ^{1}H-^{13}C HSQC NMR spectrum (d_6-DMSO, 600 MHz of ^{1}H) of 0.15:0.85 mixture of ***C5*- and *C6*-19-Dap (49)**

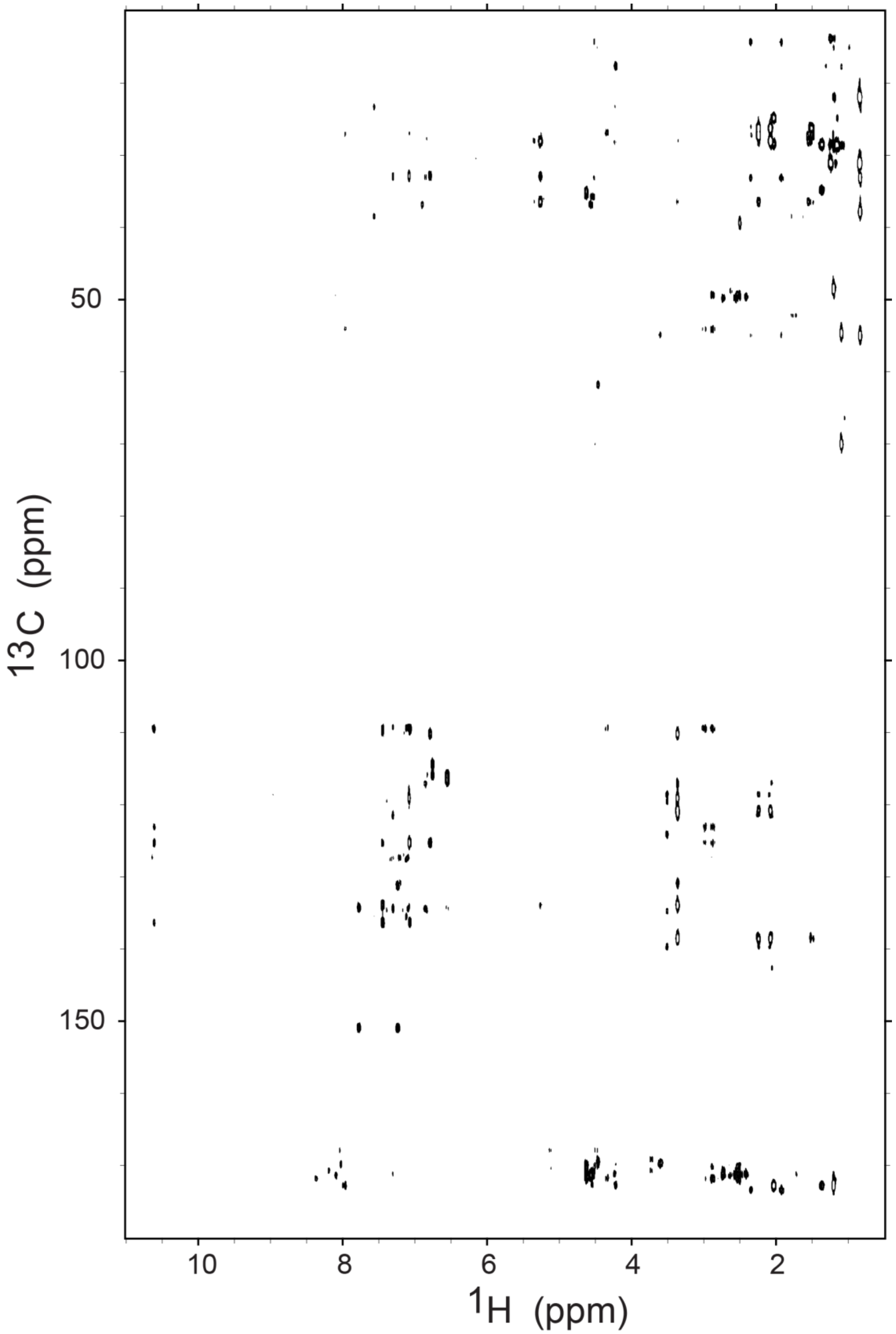

2D ^{1}H-^{13}C HMBC NMR spectrum (d_6-DMSO, 600 MHz of ^{1}H) of

0.15:0.85 mixture of ***C5*- and *C6*-19-Dap (49)**

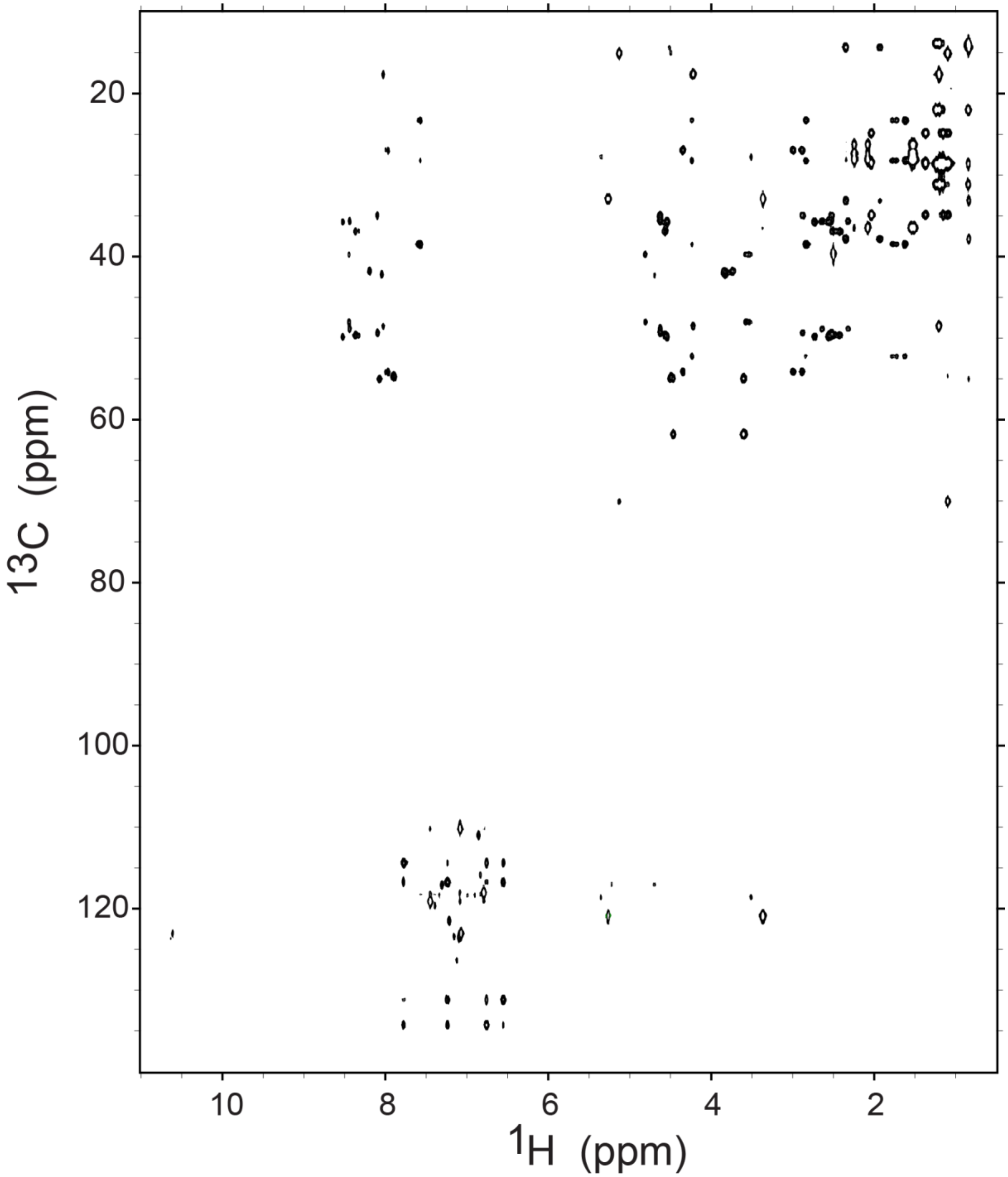

2D ^{1}H-^{13}C HSQC-TOCSY NMR spectrum (d$_6$-DMSO, 600 MHz of ^{1}H) of 0.15:0.85 mixture of ***C5*-** and ***C6*-19-Dap (49)**

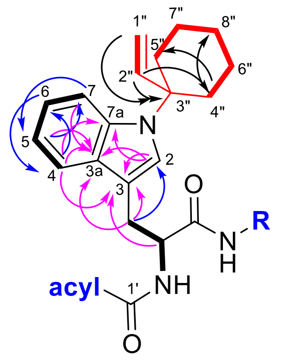

***N1*[R]-19-Dap (50)**

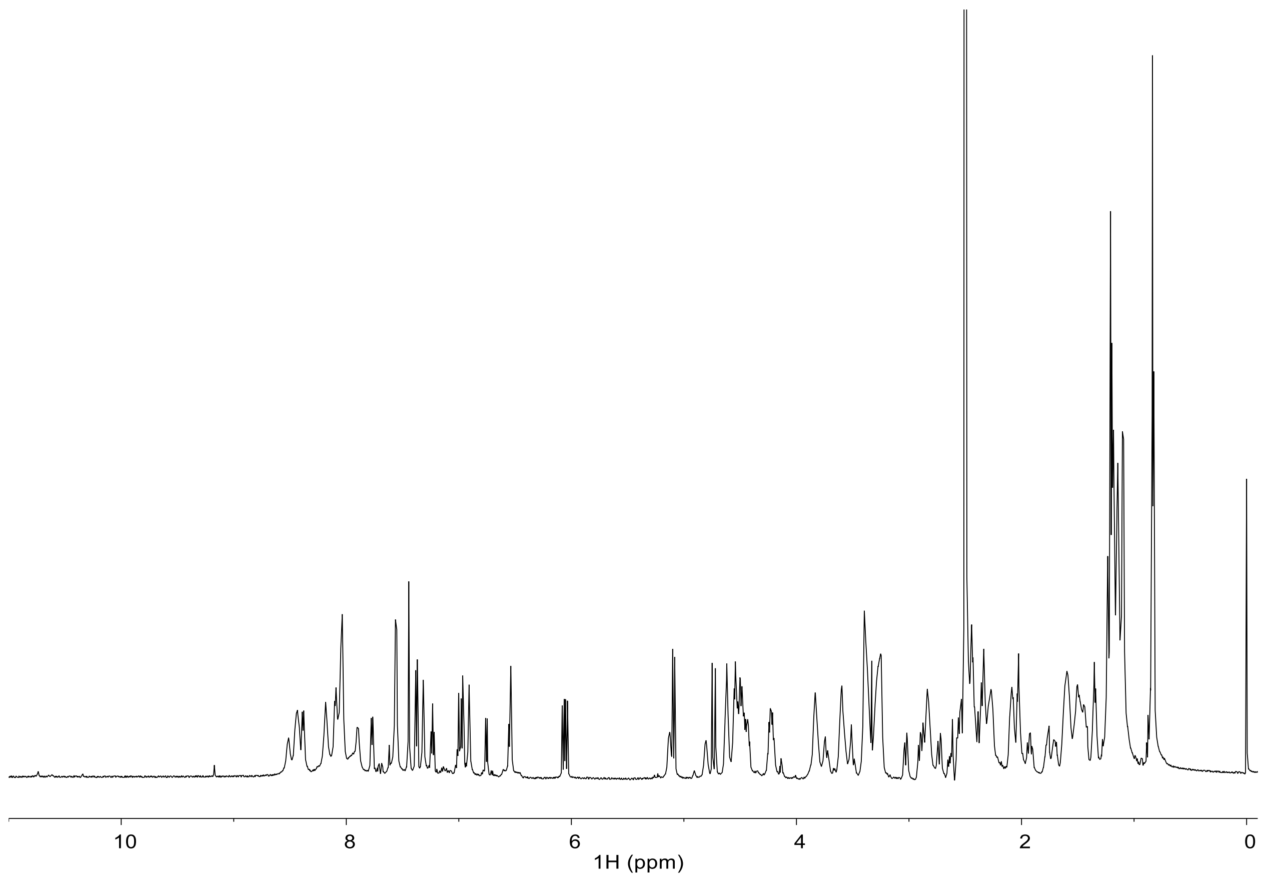

1D ^{1}H NMR spectrum (d$_6$-DMSO, 600 MHz of ^{1}H) of ***N1*[R]-19-Dap (50)**

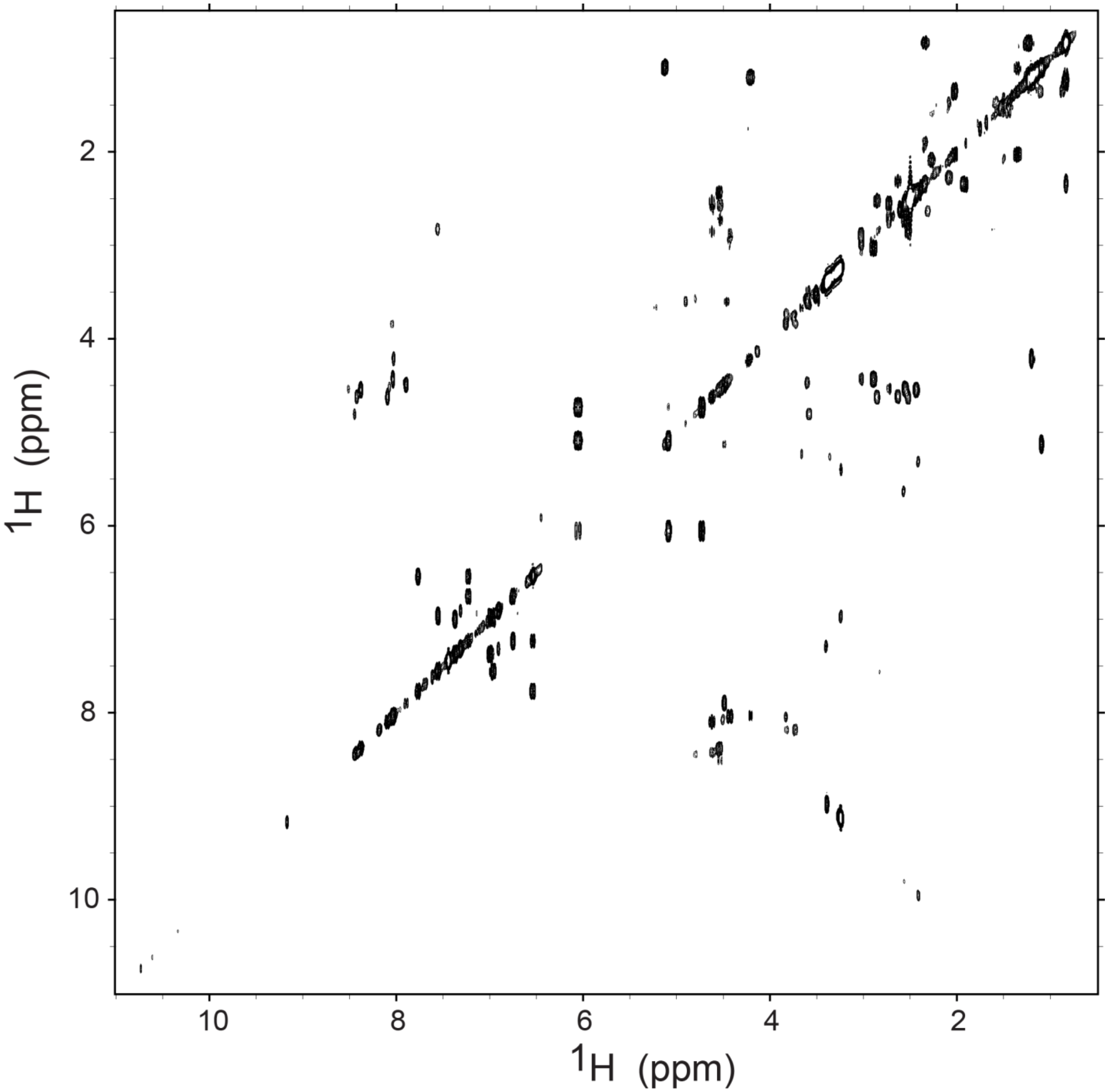

2D ^{1}H-^{1}H COSY NMR spectrum (d$_6$-DMSO, 600 MHz of ^{1}H) of ***N1*^{R}-19-Dap (50)**

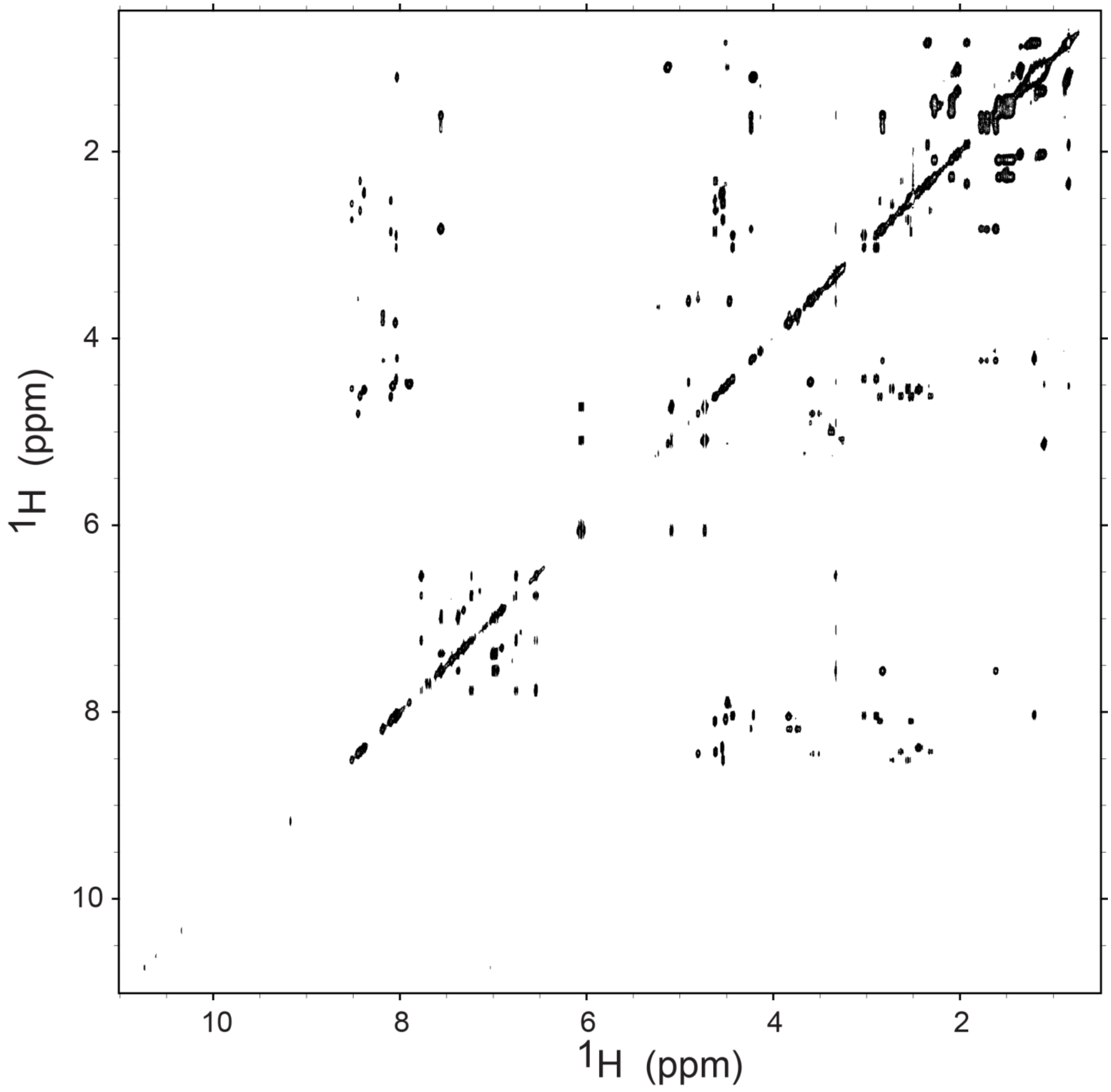

2D ^{1}H-^{1}H TOCSY NMR spectrum (d_6-DMSO, 600 MHz of ^{1}H) of ***N1*^R^-19-Dap (50)**

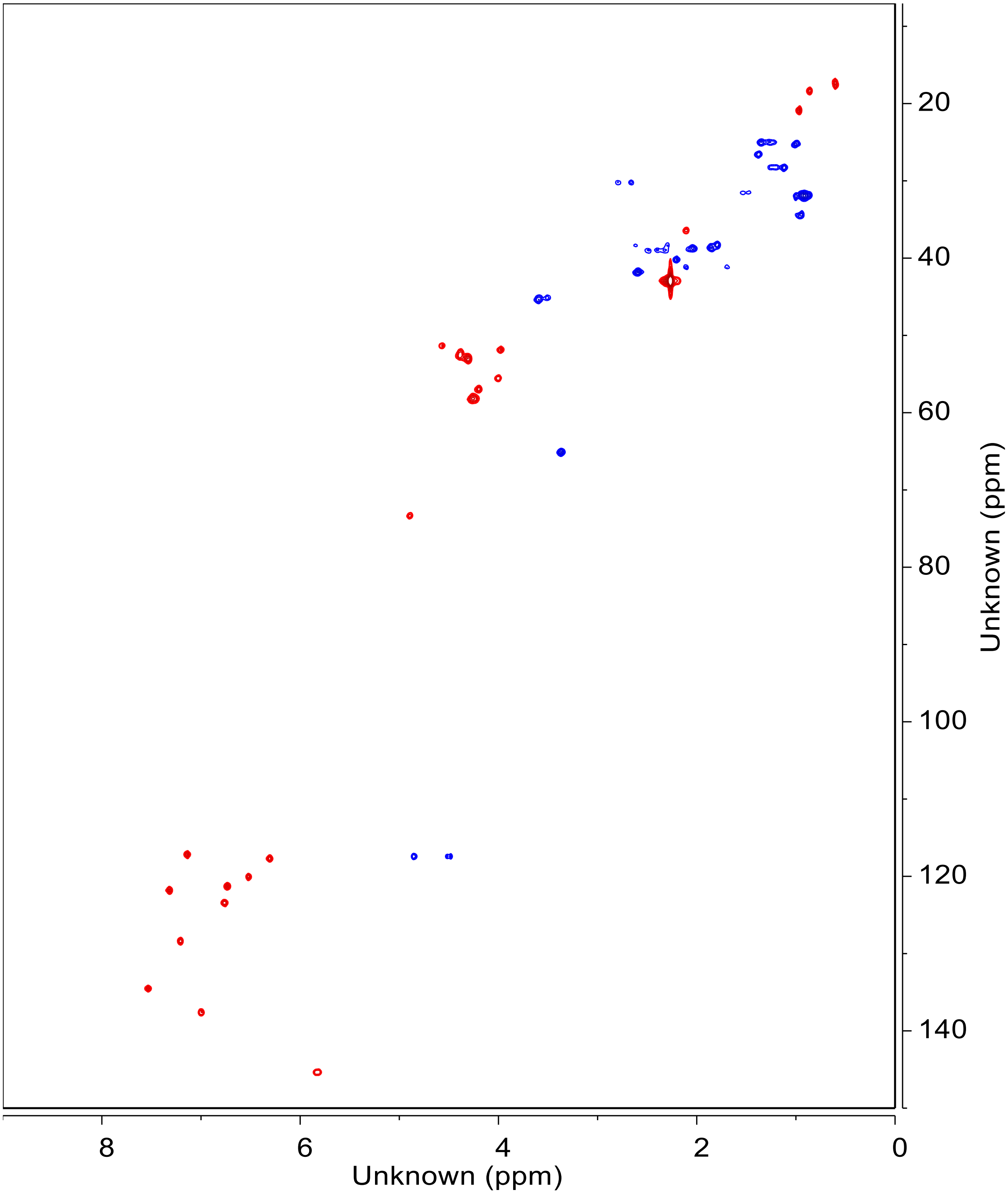

2D ^{1}H-^{13}C HSQC NMR spectrum (d_6-DMSO, 600 MHz of ^{1}H) of ***N1*^{R}-19-Dap (50)**

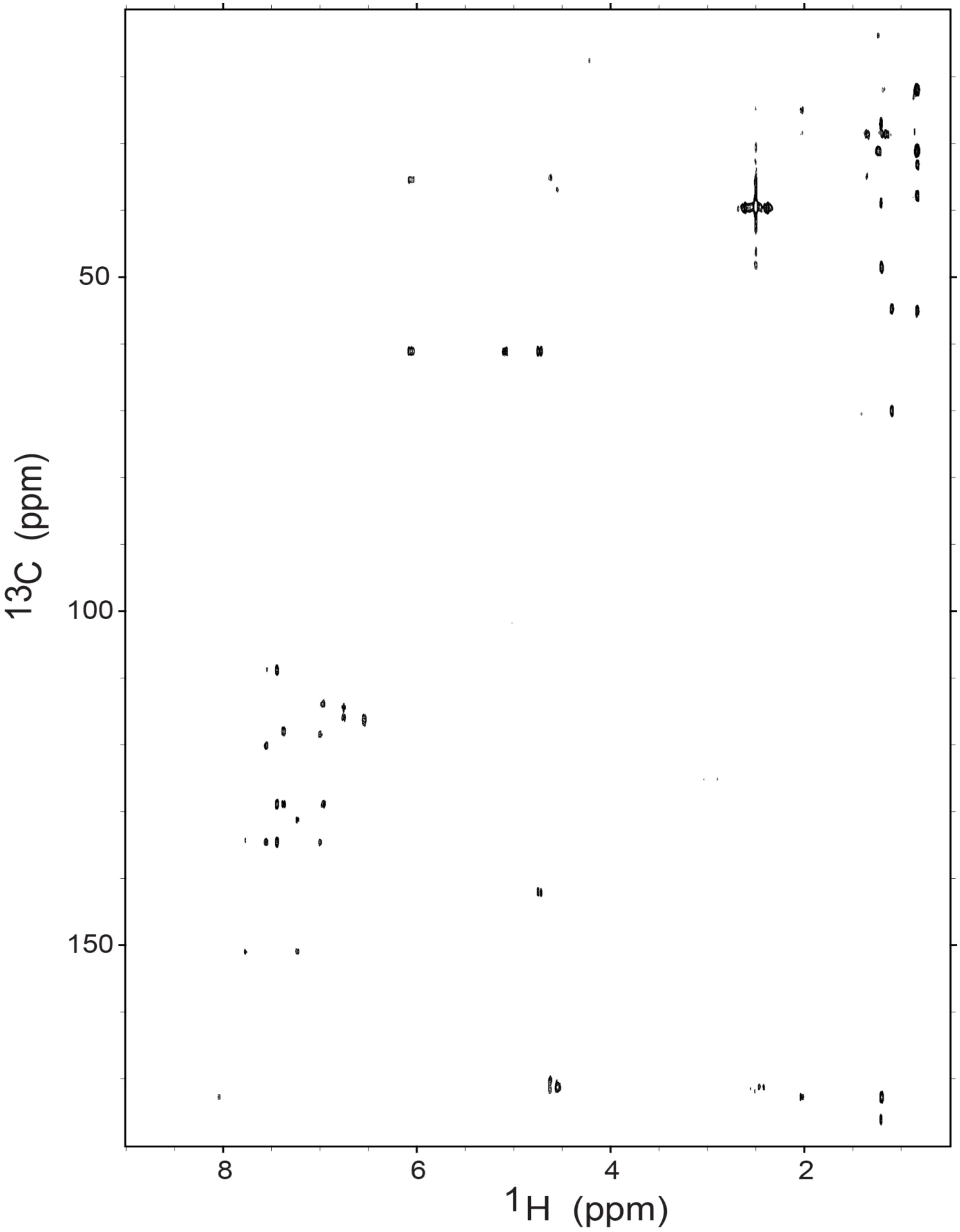

2D ^{1}H-^{13}C HMBC NMR spectrum (d_6-DMSO, 600 MHz of ^{1}H) of ***N1*^R-19-Dap (50)**

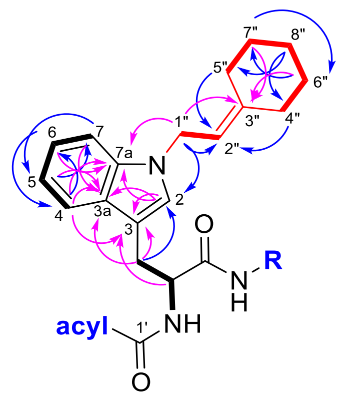

***N1*-19-Dap (51)**

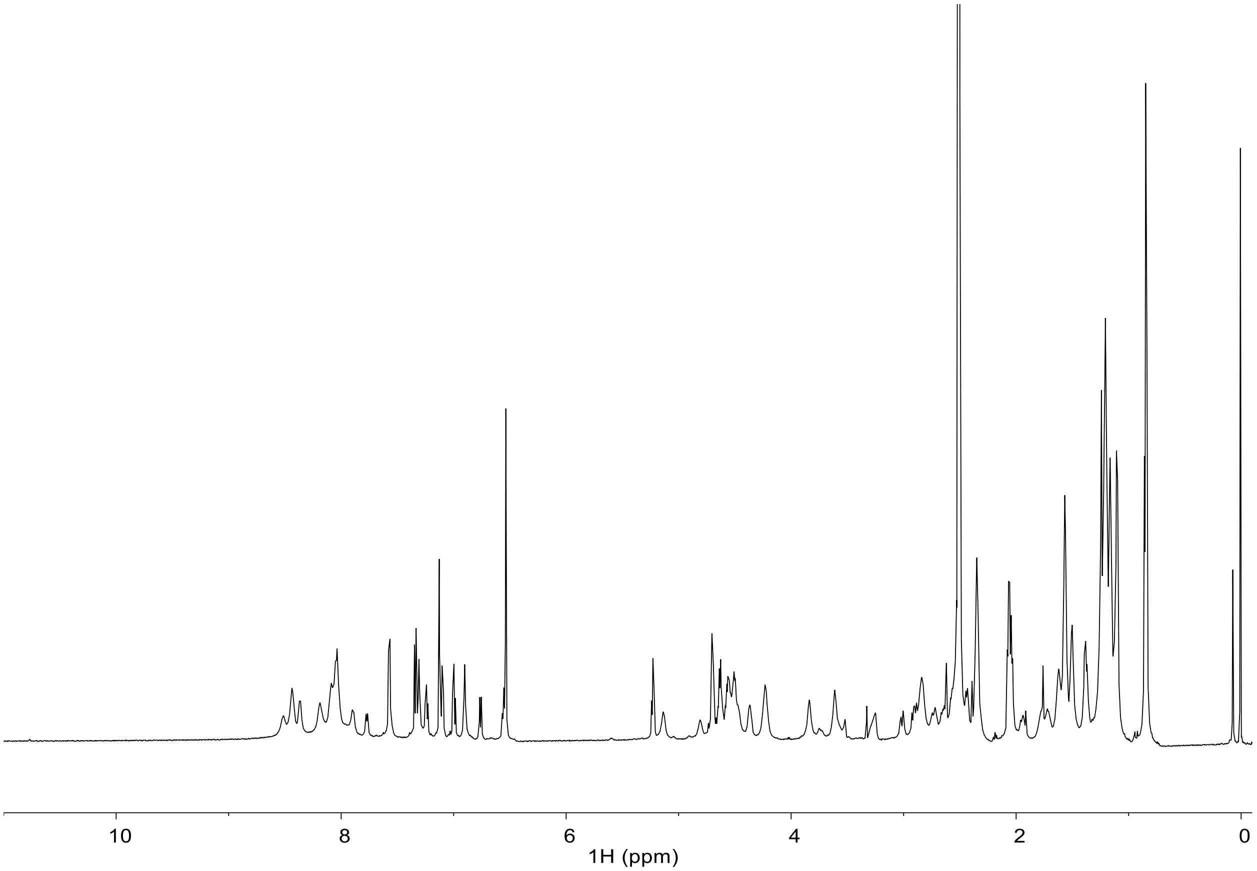

1D ^{1}H NMR spectrum (d_6-DMSO, 600 MHz of ^{1}H) of ***N1*-19-Dap (51)**

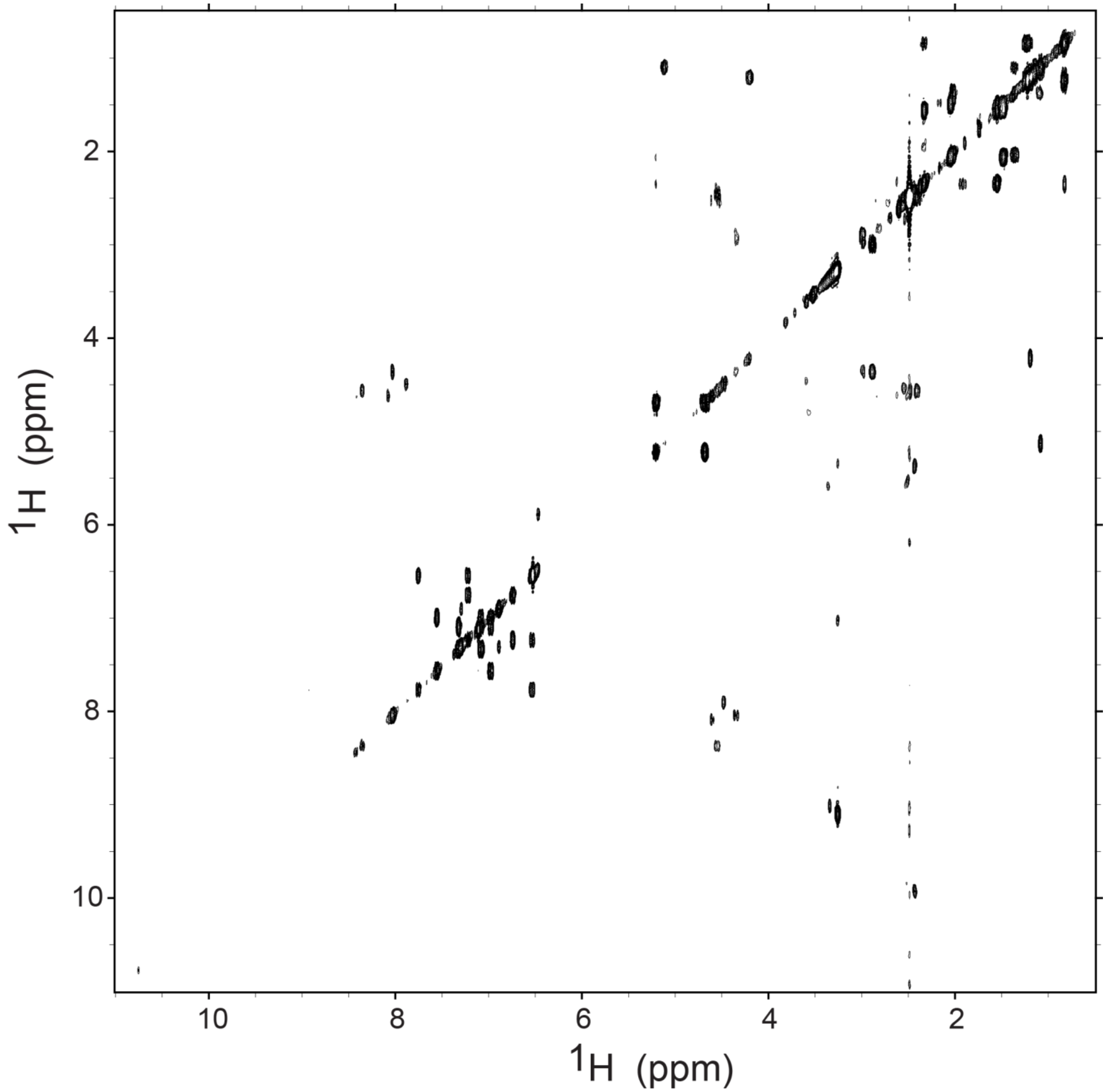

2D ^{1}H-^{1}H COSY NMR spectrum (d_6-DMSO, 600 MHz of ^{1}H) of ***N1*-19-Dap (51)**

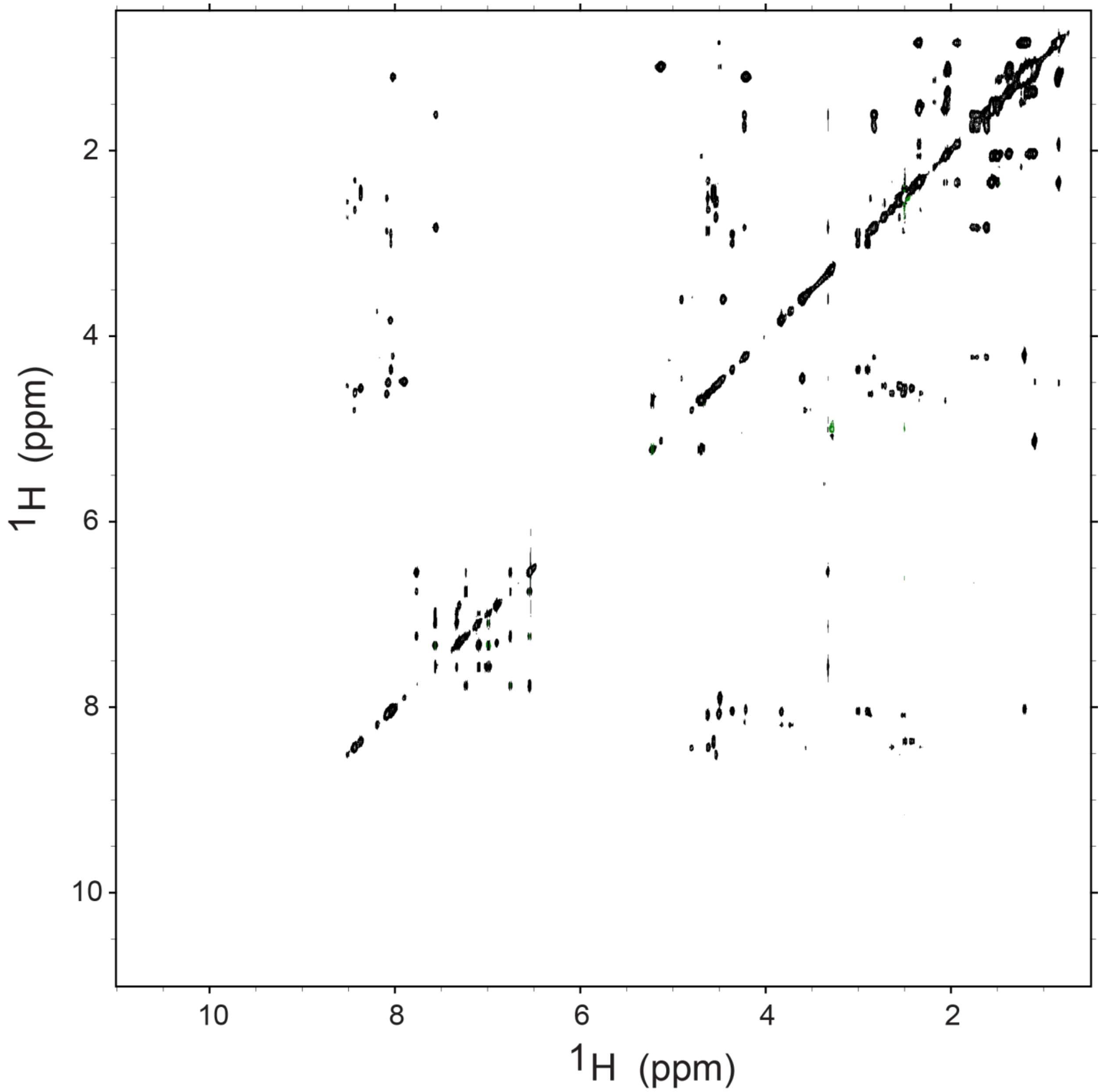

2D ^{1}H-^{1}H TOCSY NMR spectrum (d_6-DMSO, 600 MHz of ^{1}H) of ***N1*-19-Dap (51)**

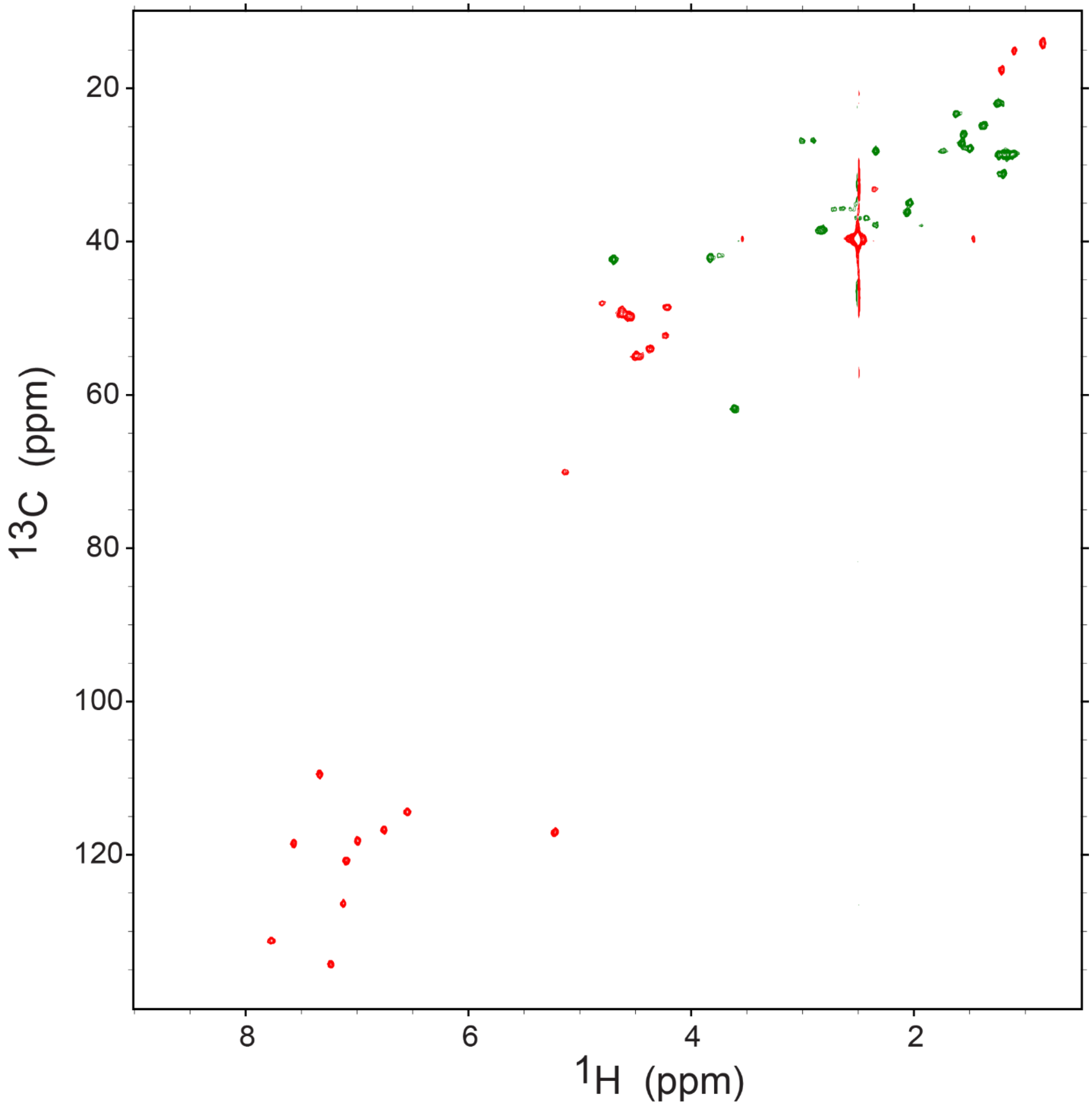

2D ^{1}H-^{13}C HSQC NMR spectrum (d_6-DMSO, 600 MHz of ^{1}H) of ***N1*-19-Dap (51)**

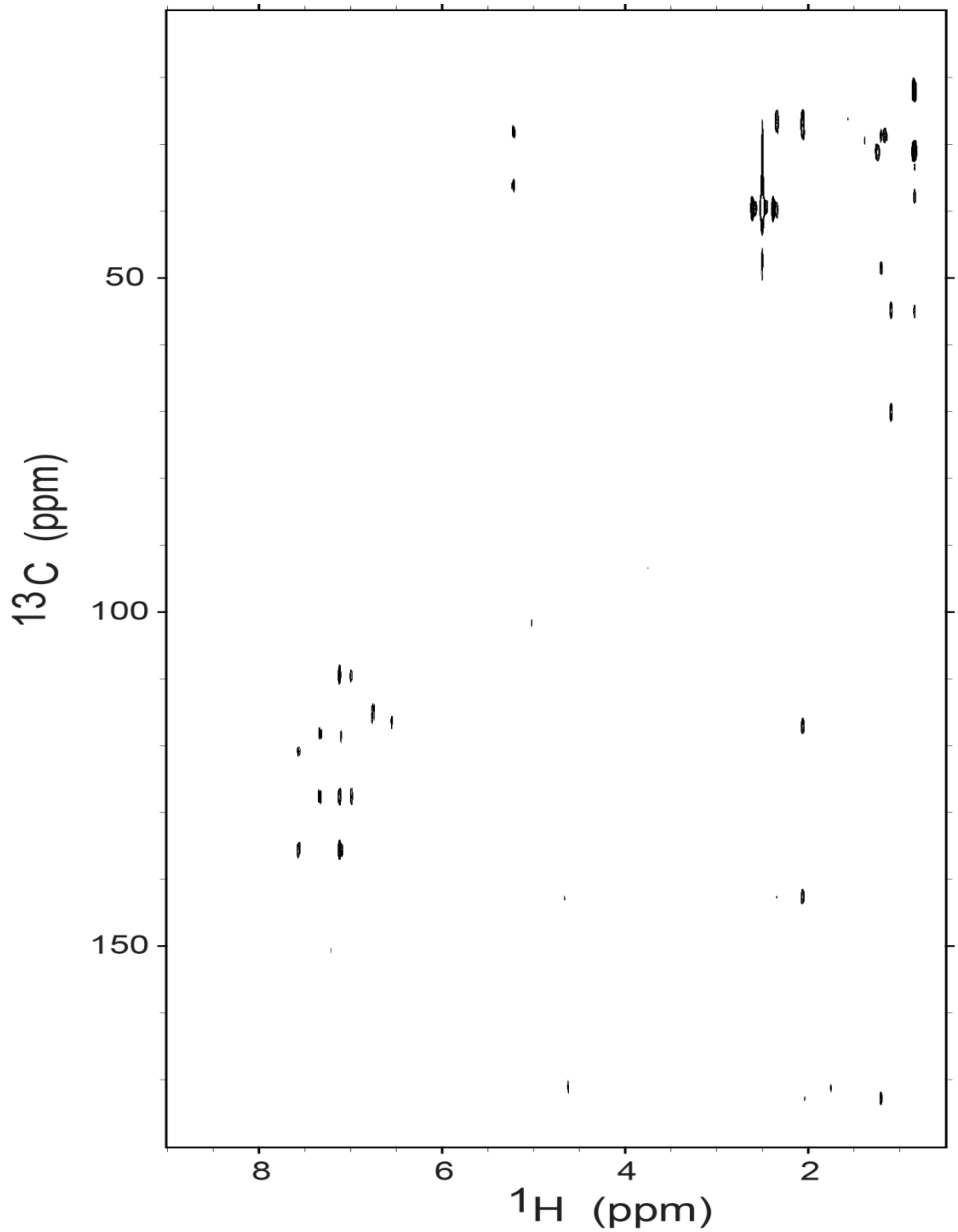

2D ^{1}H-^{13}C HMBC NMR spectrum (d_6-DMSO, 600 MHz of ^{1}H) of ***N1*-19-Dap (51)**

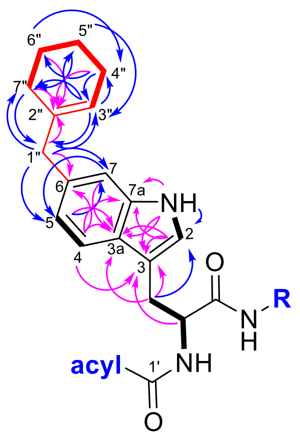

***C6*-20-Dap (52)**

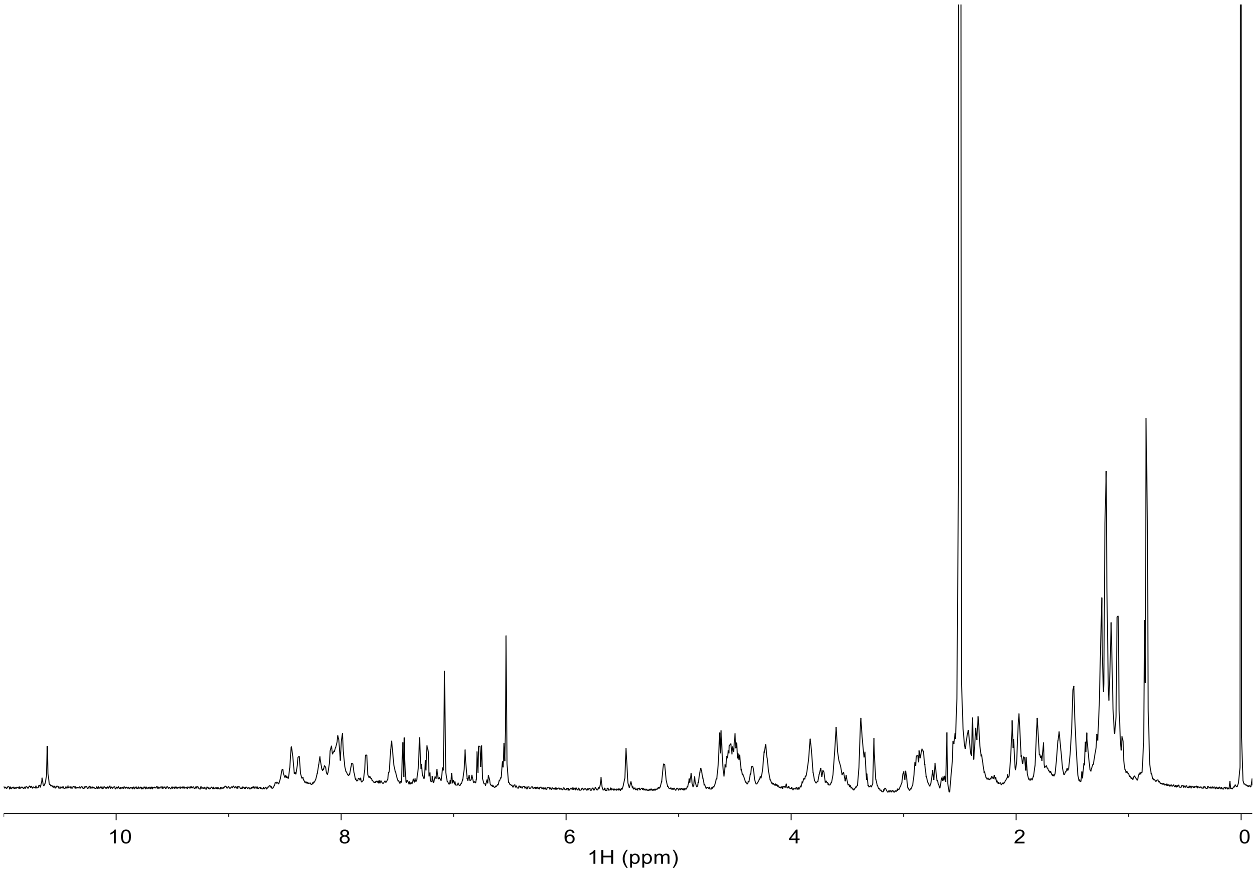

1D ^{1}H NMR spectrum (d_6-DMSO, 600 MHz of ^{1}H) of ***C6*-20-Dap (52)**

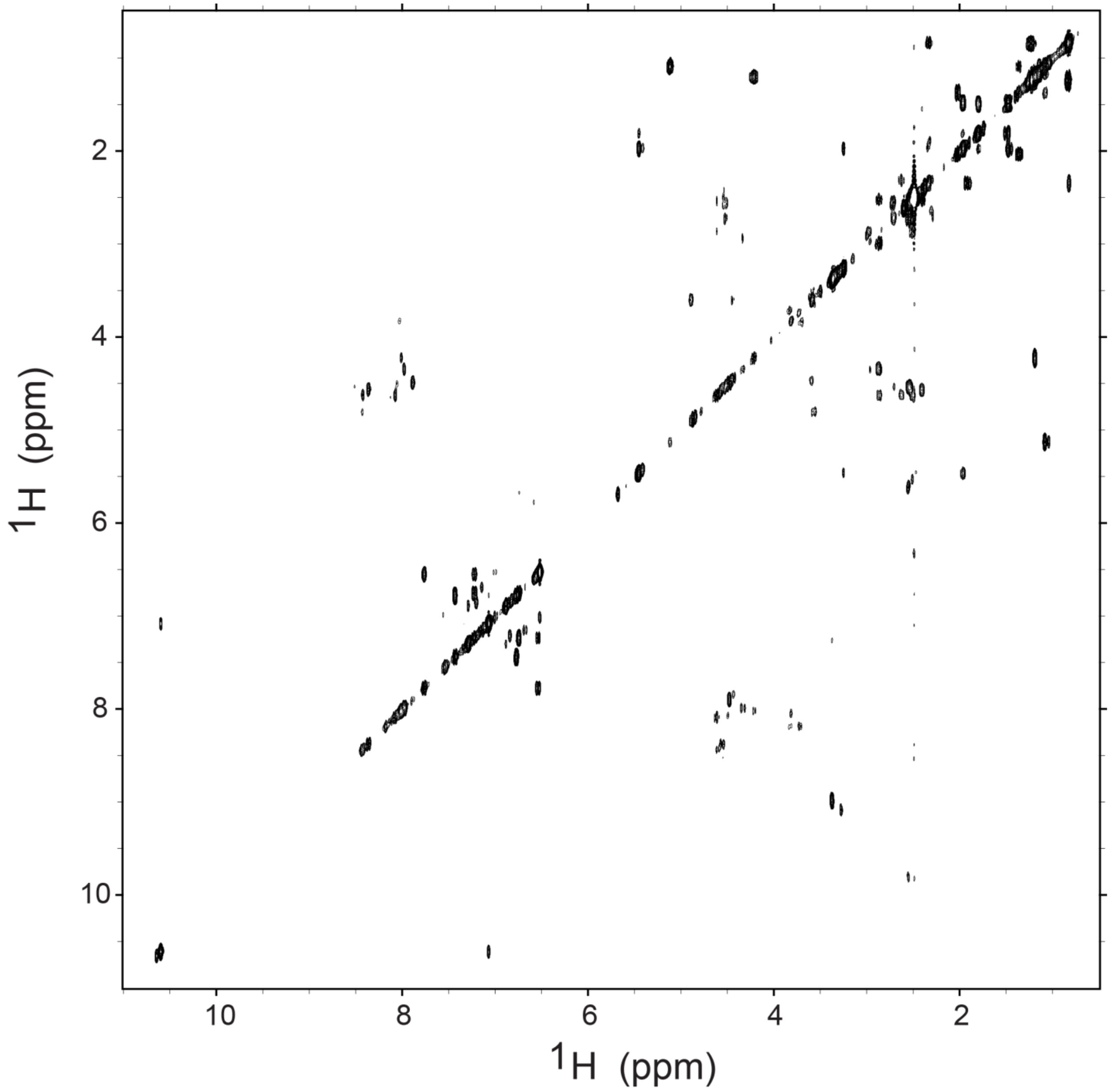

2D ^{1}H-^{1}H COSY NMR spectrum (d_6-DMSO, 600 MHz of ^{1}H) of ***C6*-20-Dap (52)**

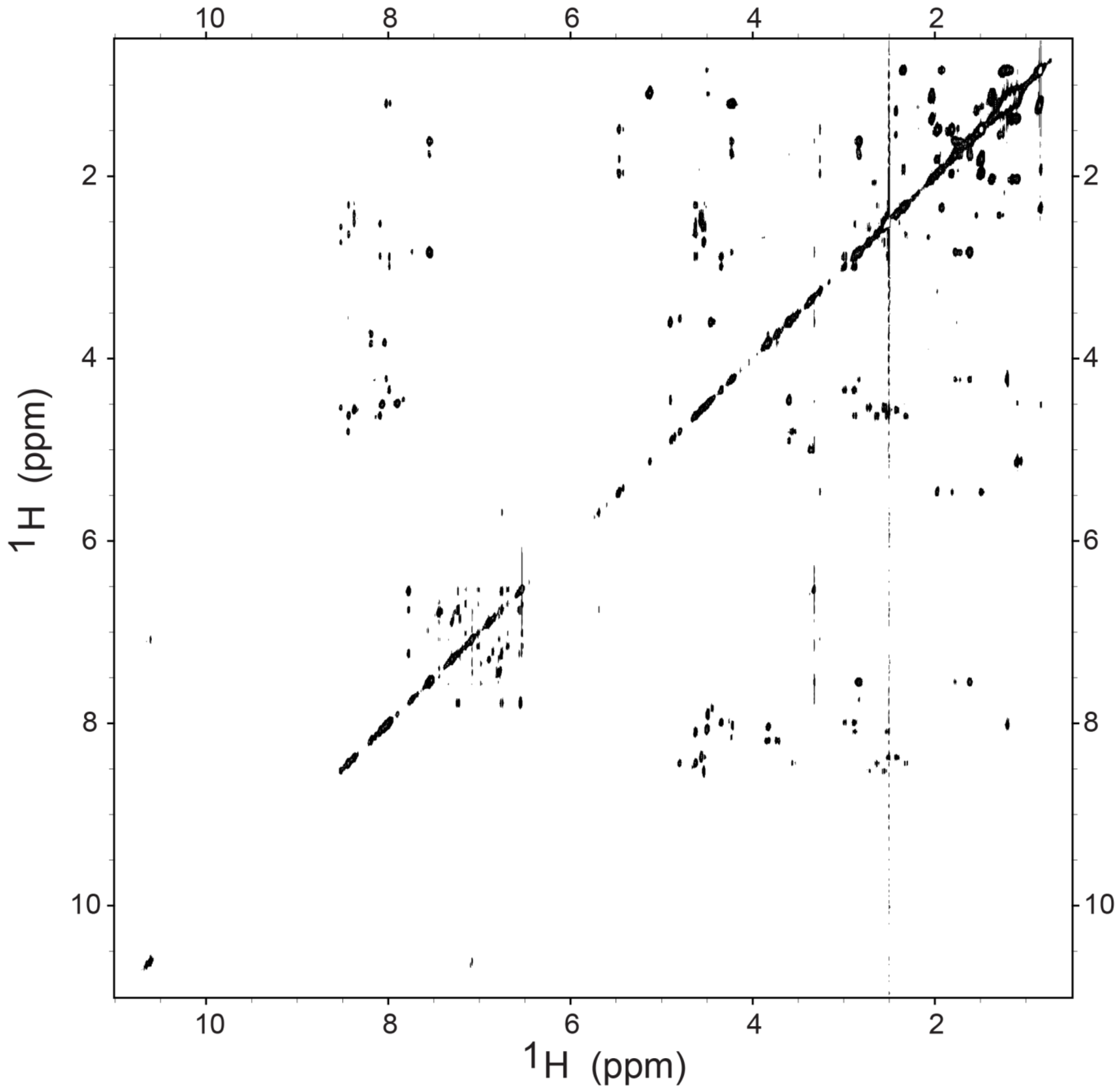

2D ^{1}H-^{1}H TOCSY NMR spectrum (d_6-DMSO, 600 MHz of ^{1}H) of ***C6*-20-Dap (52)**

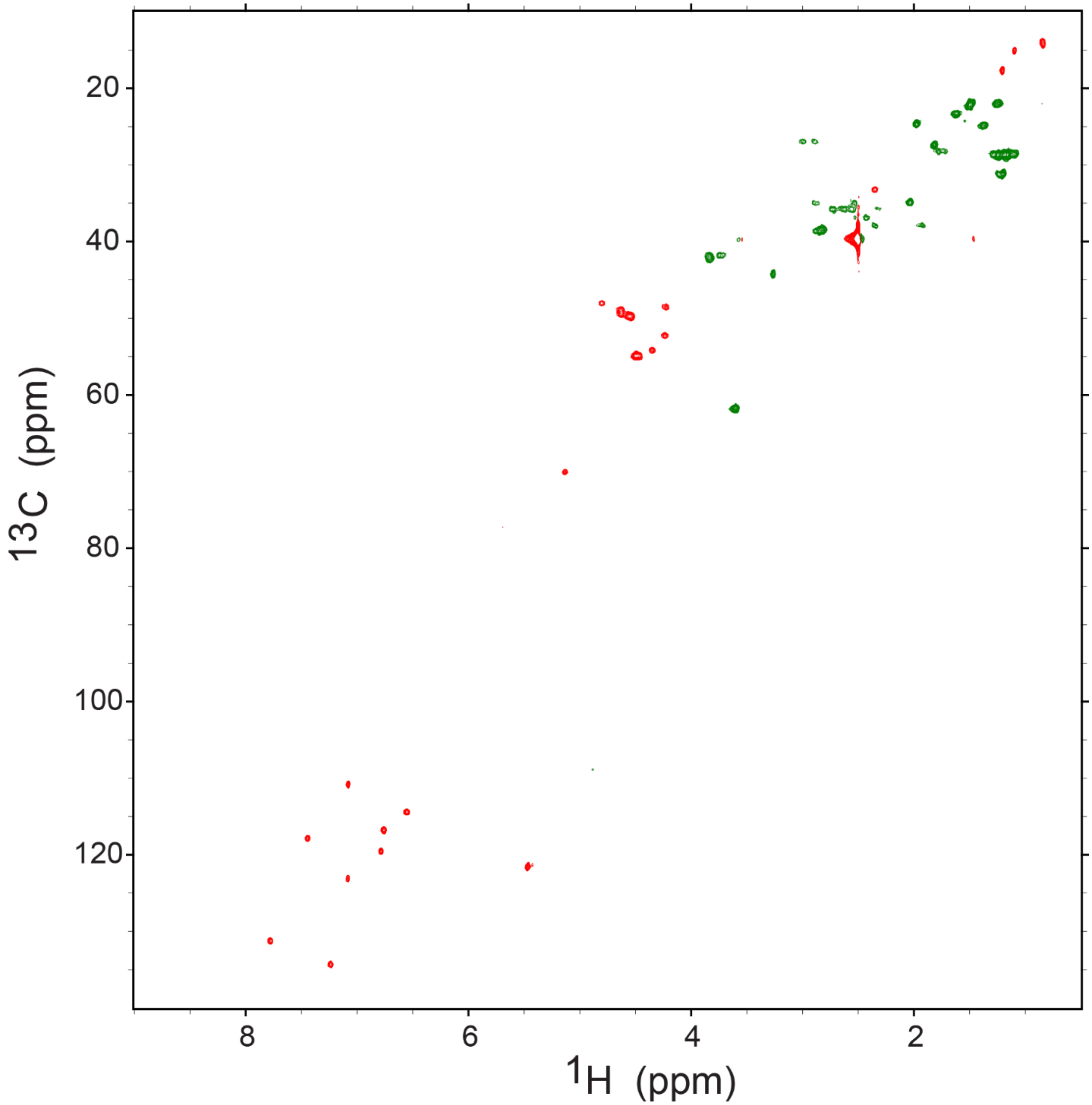

2D ^{1}H-^{13}C HSQC NMR spectrum (d_6-DMSO, 600 MHz of ^{1}H) of ***C6*-20-Dap (52)**

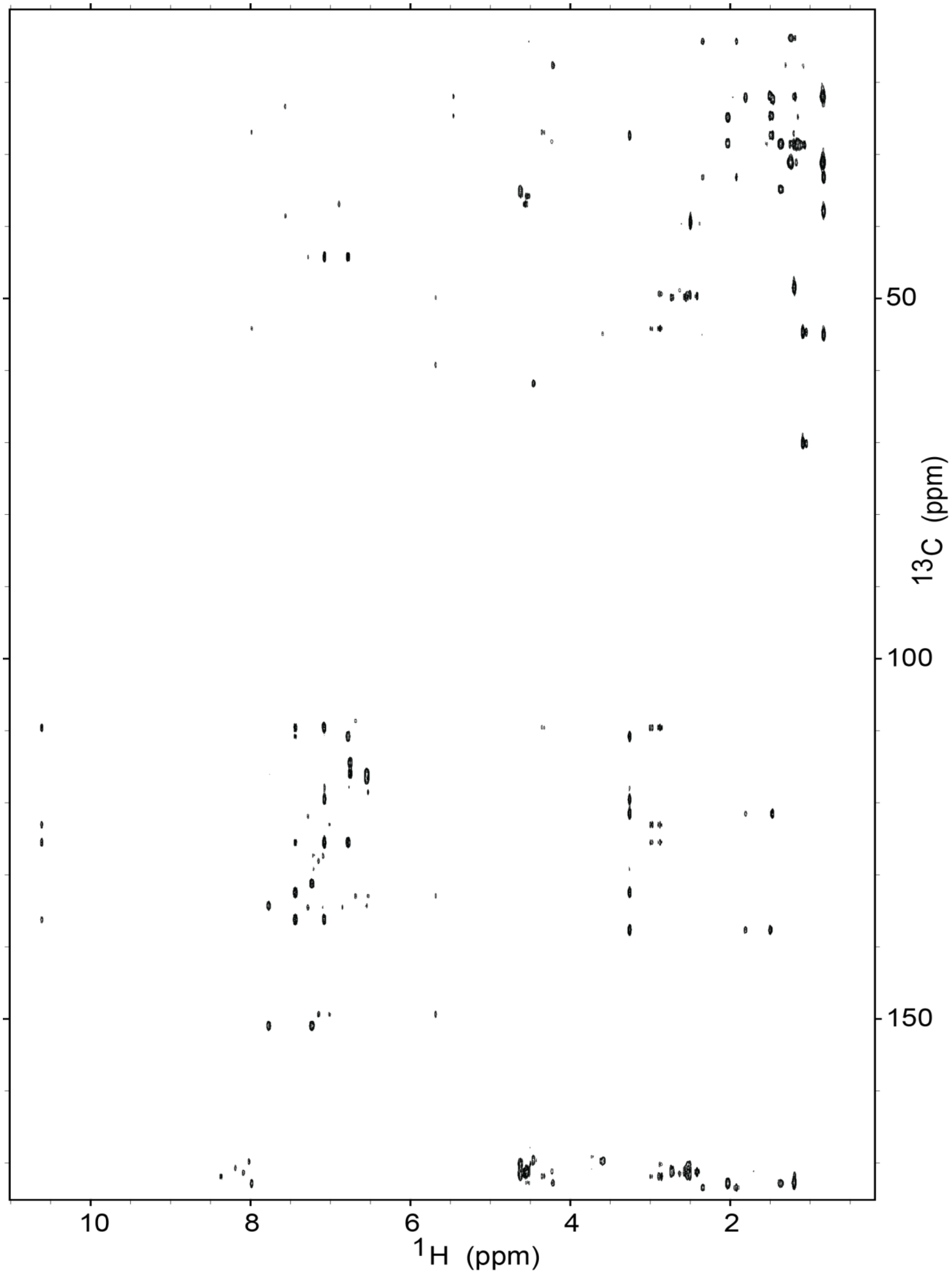

2D ^1H-^{13}C HMBC NMR spectrum (d_6-DMSO, 600 MHz of ^1H) of ***C6*-20-Dap (52)**

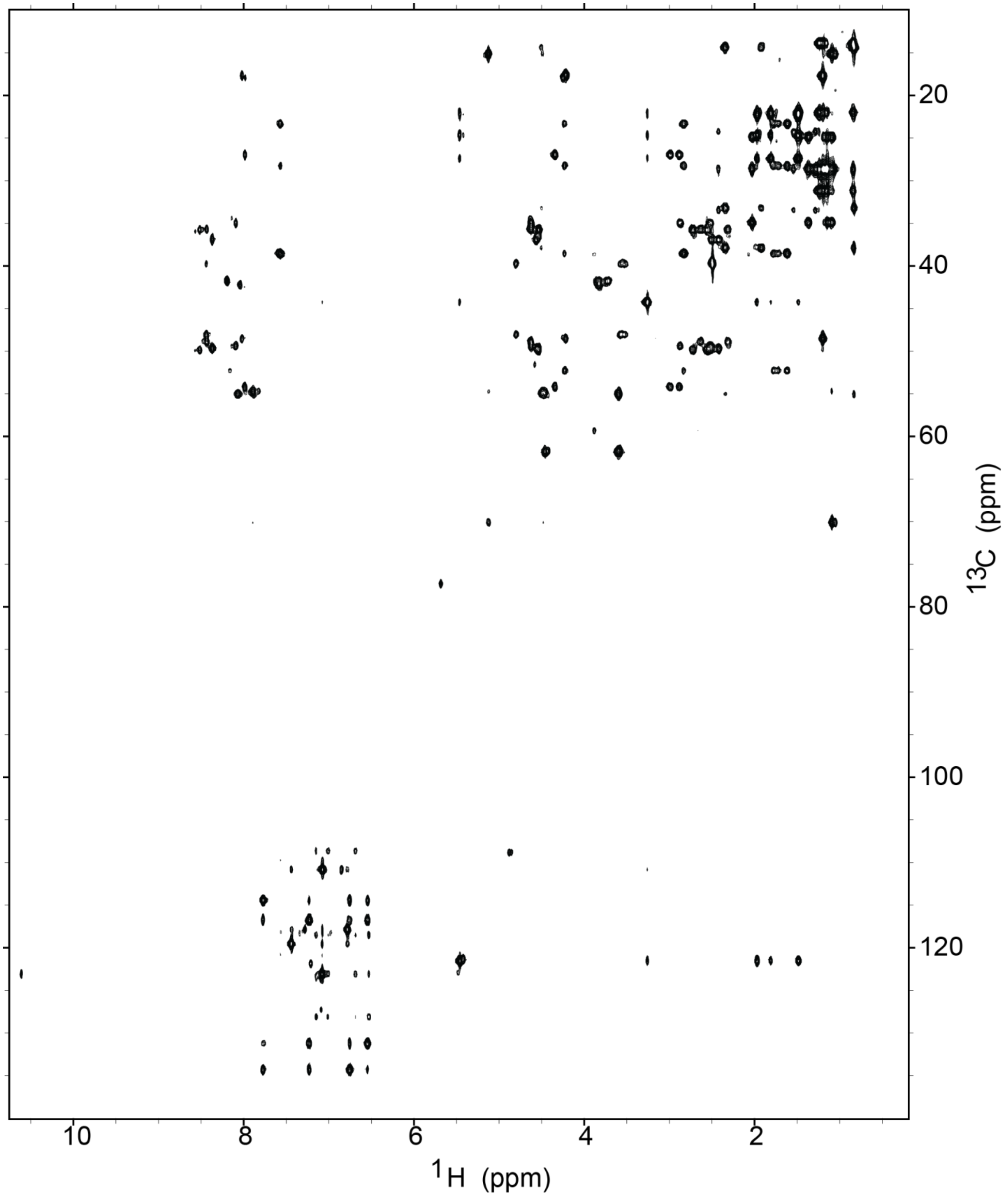

2D ^{1}H-^{13}C HSQC-TOCSY NMR spectrum (d_6-DMSO, 600 MHz of ^{1}H) of ***C6*-20-Dap (52)**

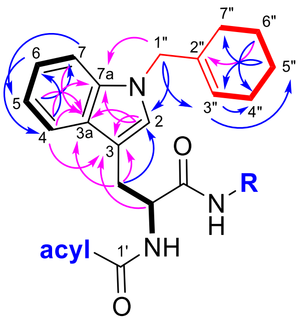

***N1*-20-Dap (53)**

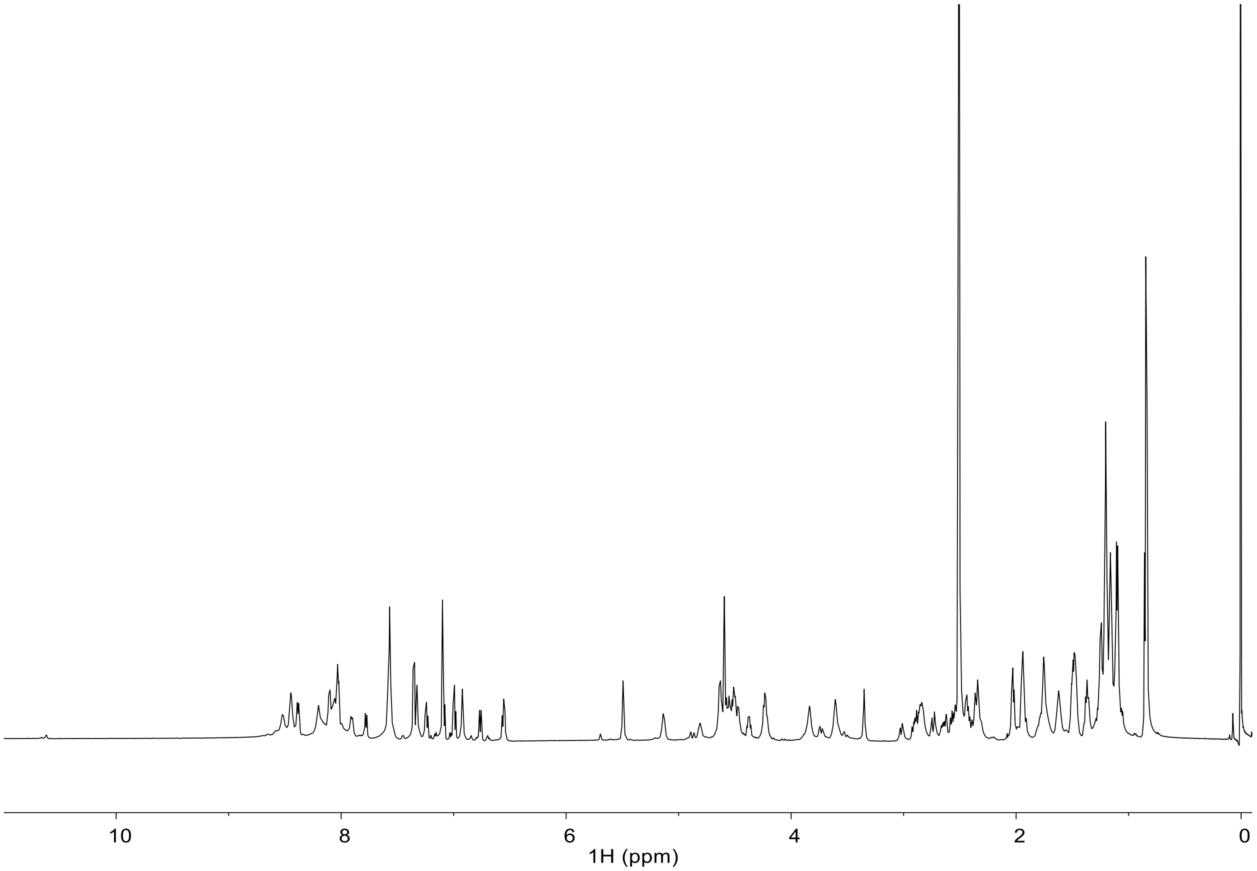

1D ^{1}H NMR spectrum (d$_6$-DMSO, 600 MHz of ^{1}H) of ***N1*-20-Dap (53)**

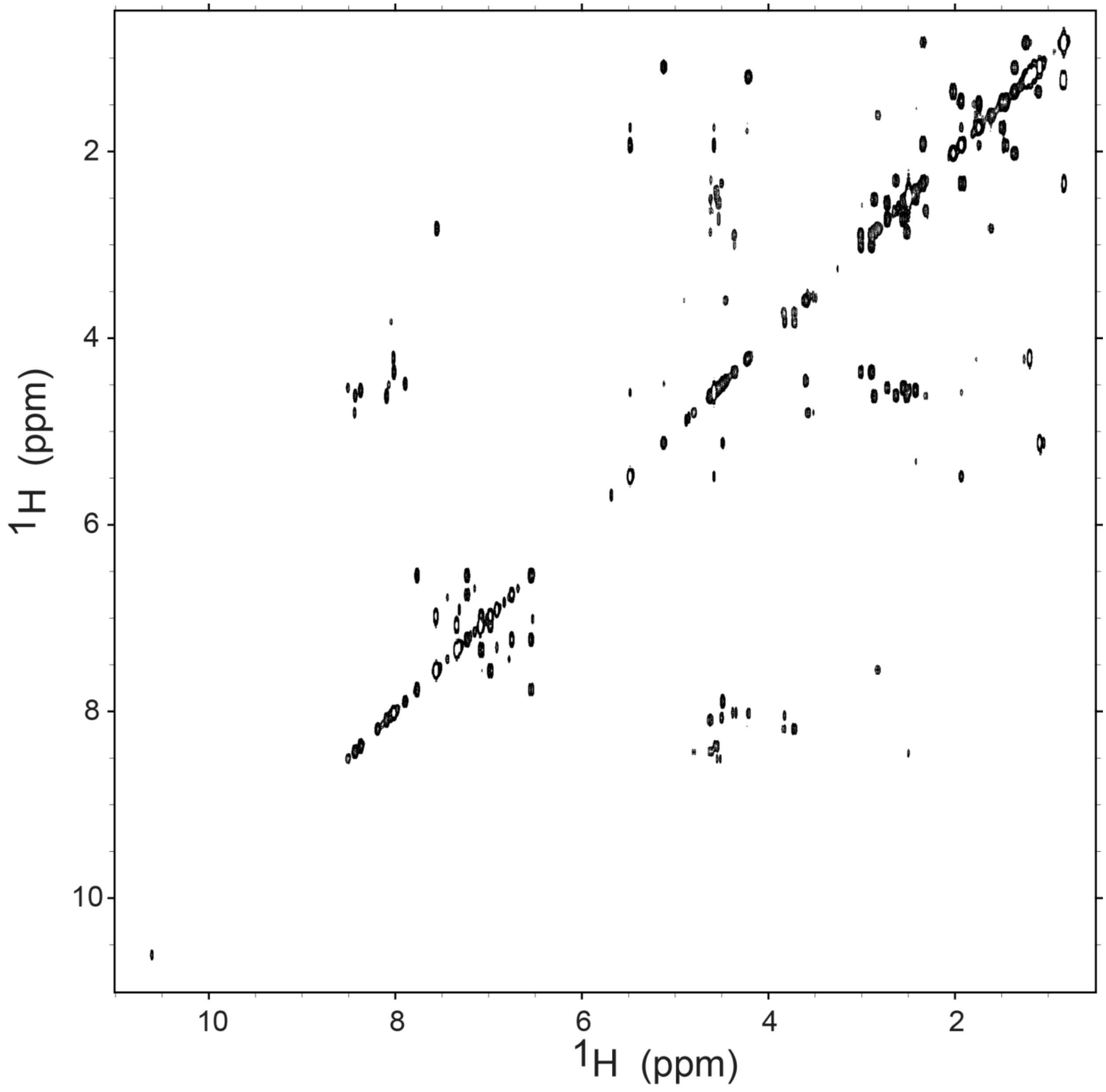

2D ^{1}H-^{1}H COSY NMR spectrum (d_6-DMSO, 600 MHz of ^{1}H) of ***N1*-20-Dap (53)**

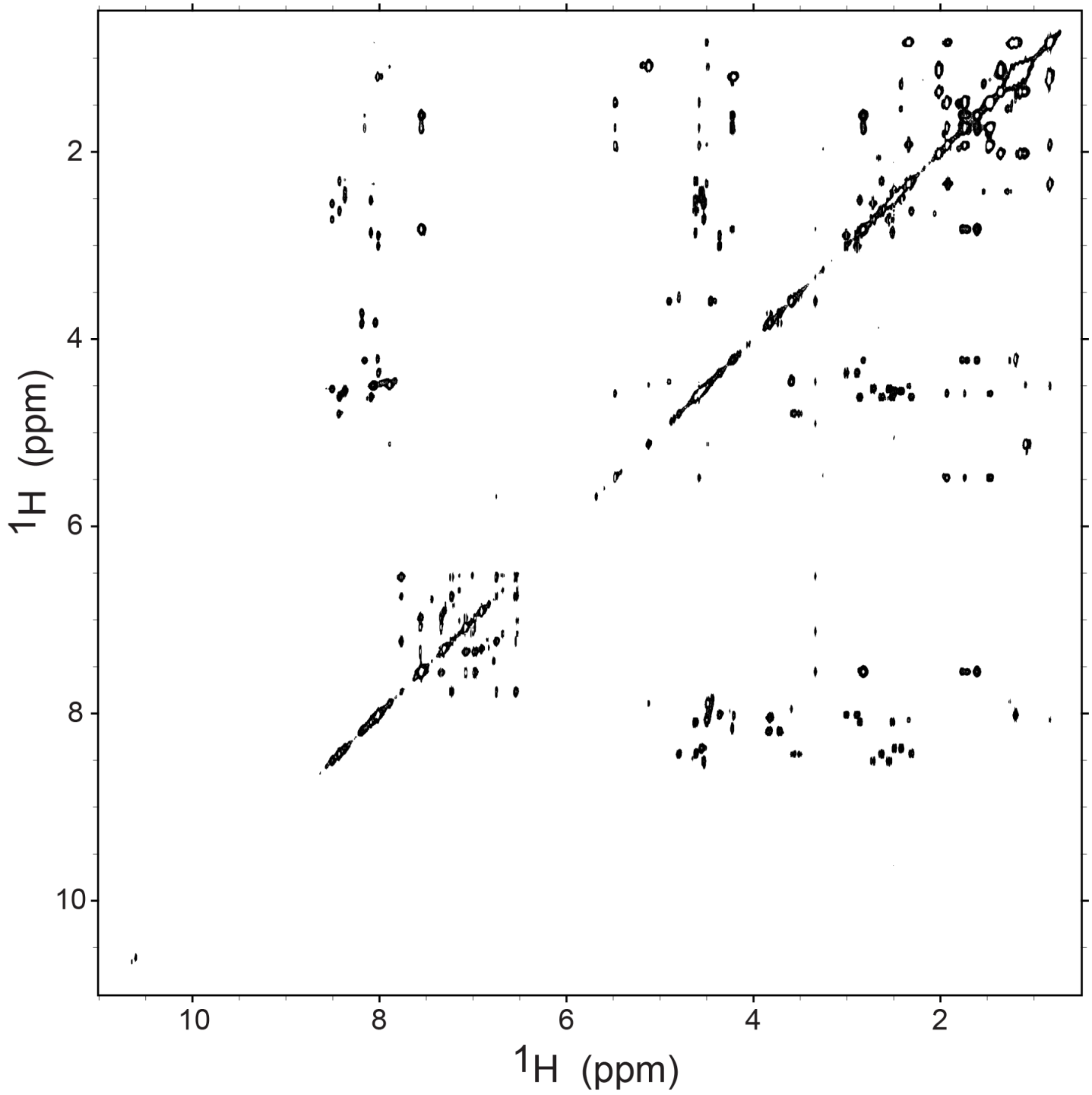

2D ^{1}H-^{1}H TOCSY NMR spectrum (d_6-DMSO, 600 MHz of ^{1}H) of ***N1*-20-Dap (53)**

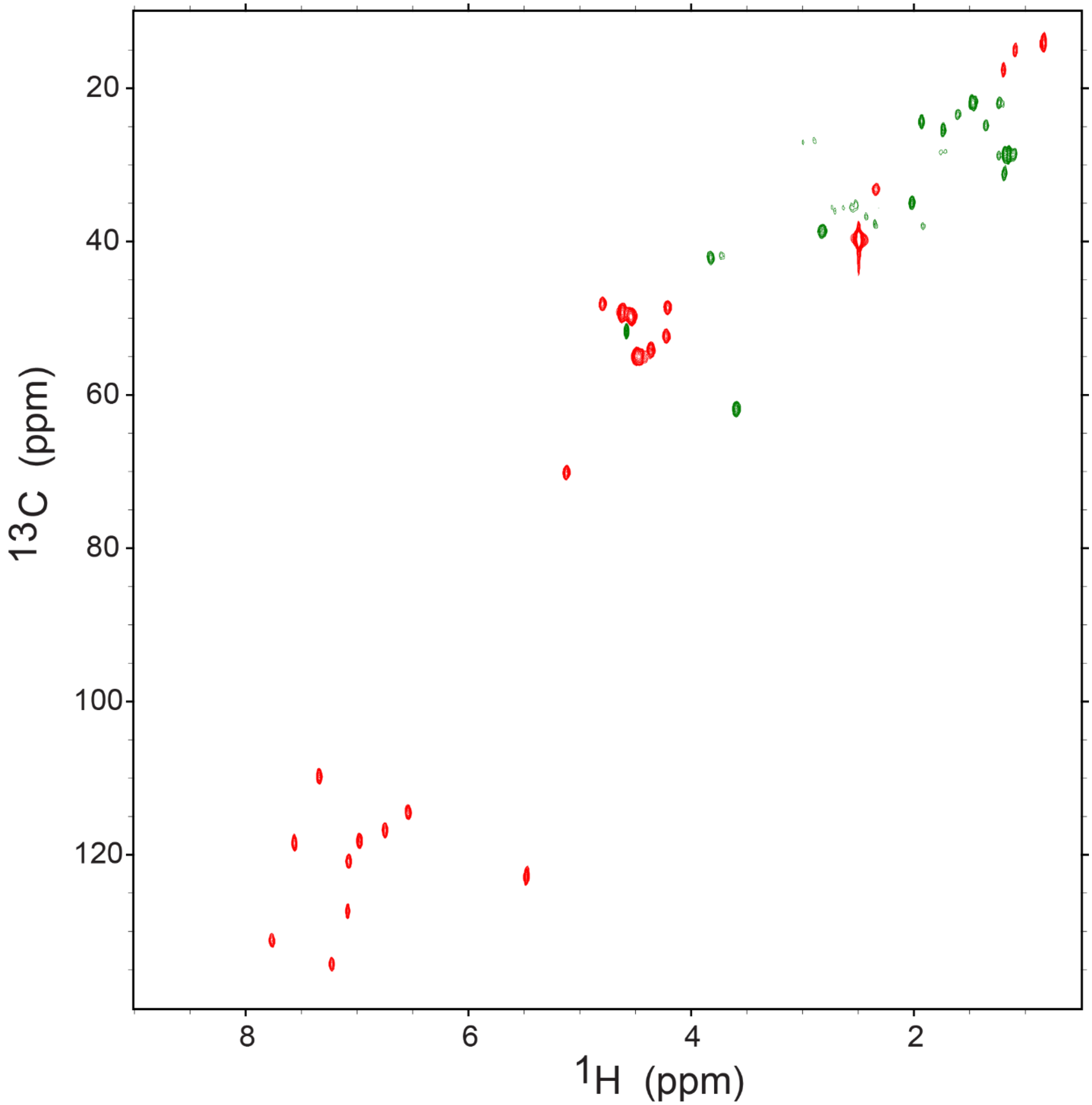

2D ^{1}H-^{13}C HSQC NMR spectrum (d_6-DMSO, 600 MHz of ^{1}H) of ***N1*-20-Dap (53)**

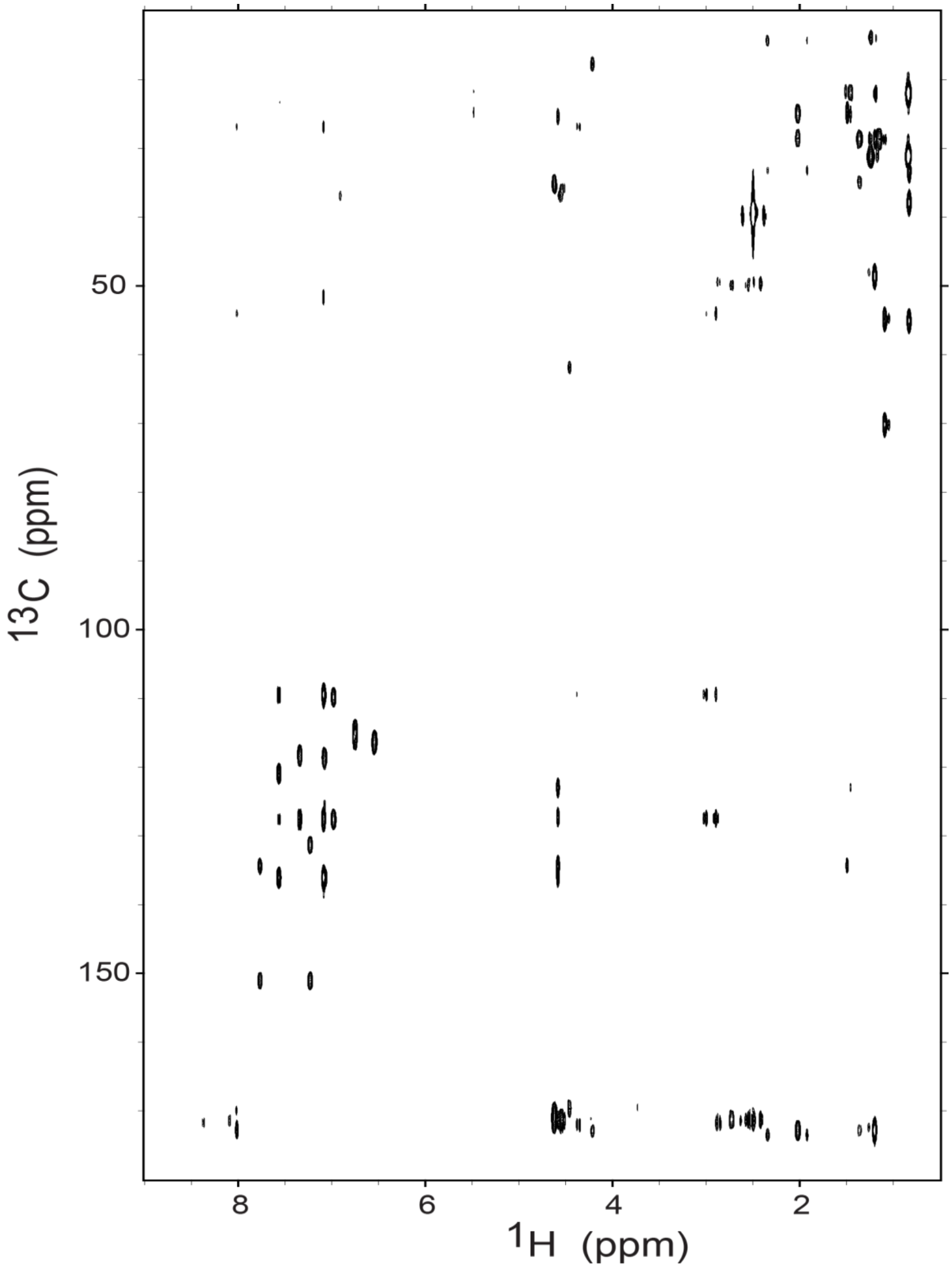

2D ^{1}H-^{13}C HMBC NMR spectrum (d_6-DMSO, 600 MHz of ^{1}H) of ***N1*-20-Dap (53)**

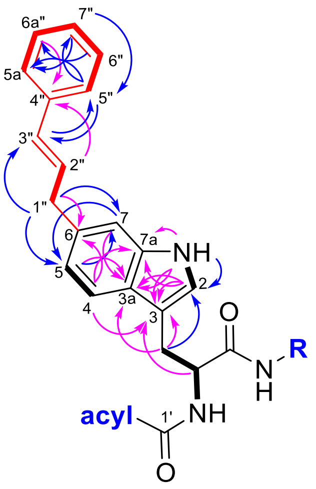

***C6*-22-Dap (54)**

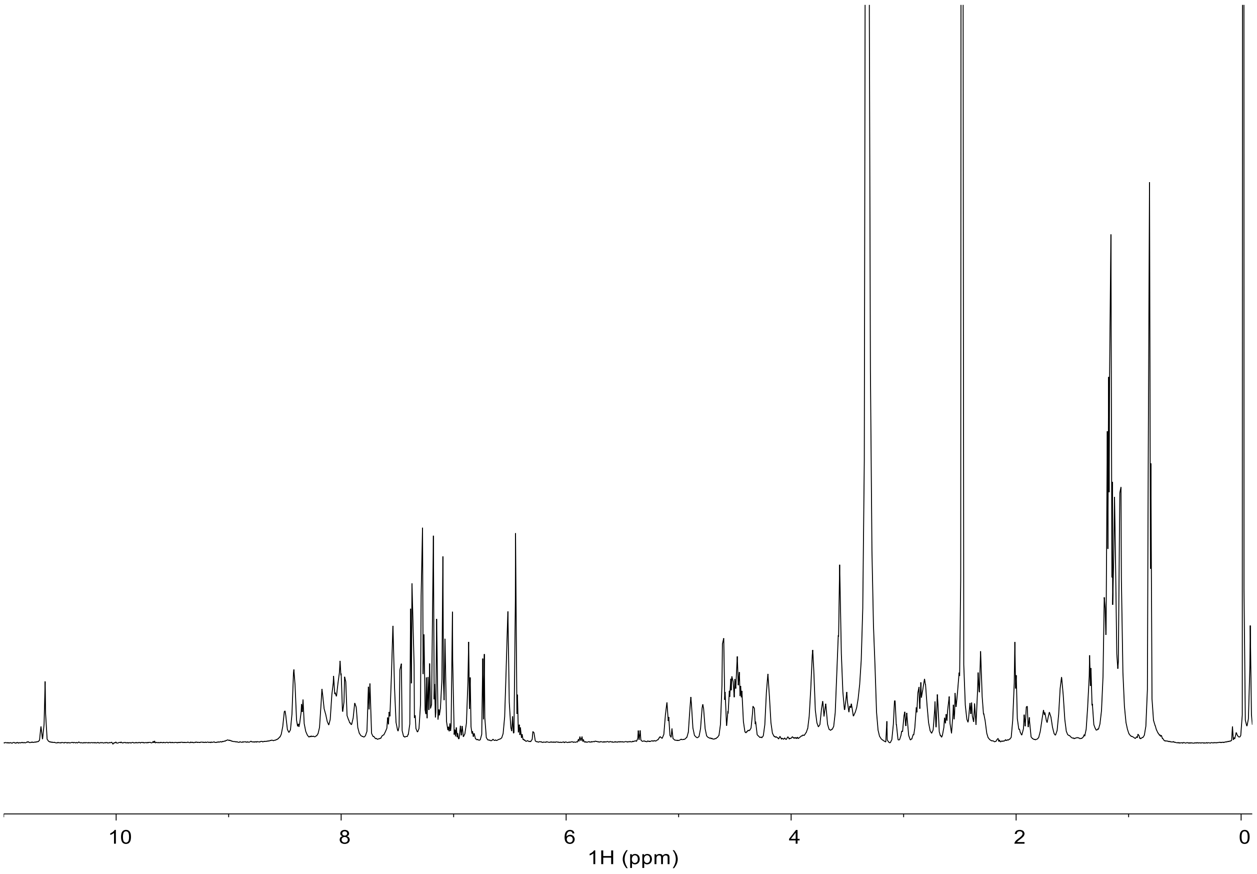

1D ^{1}H NMR spectrum (d$_6$-DMSO, 600 MHz of ^{1}H) of ***C6*-22-Dap (54)** (~85% pure)

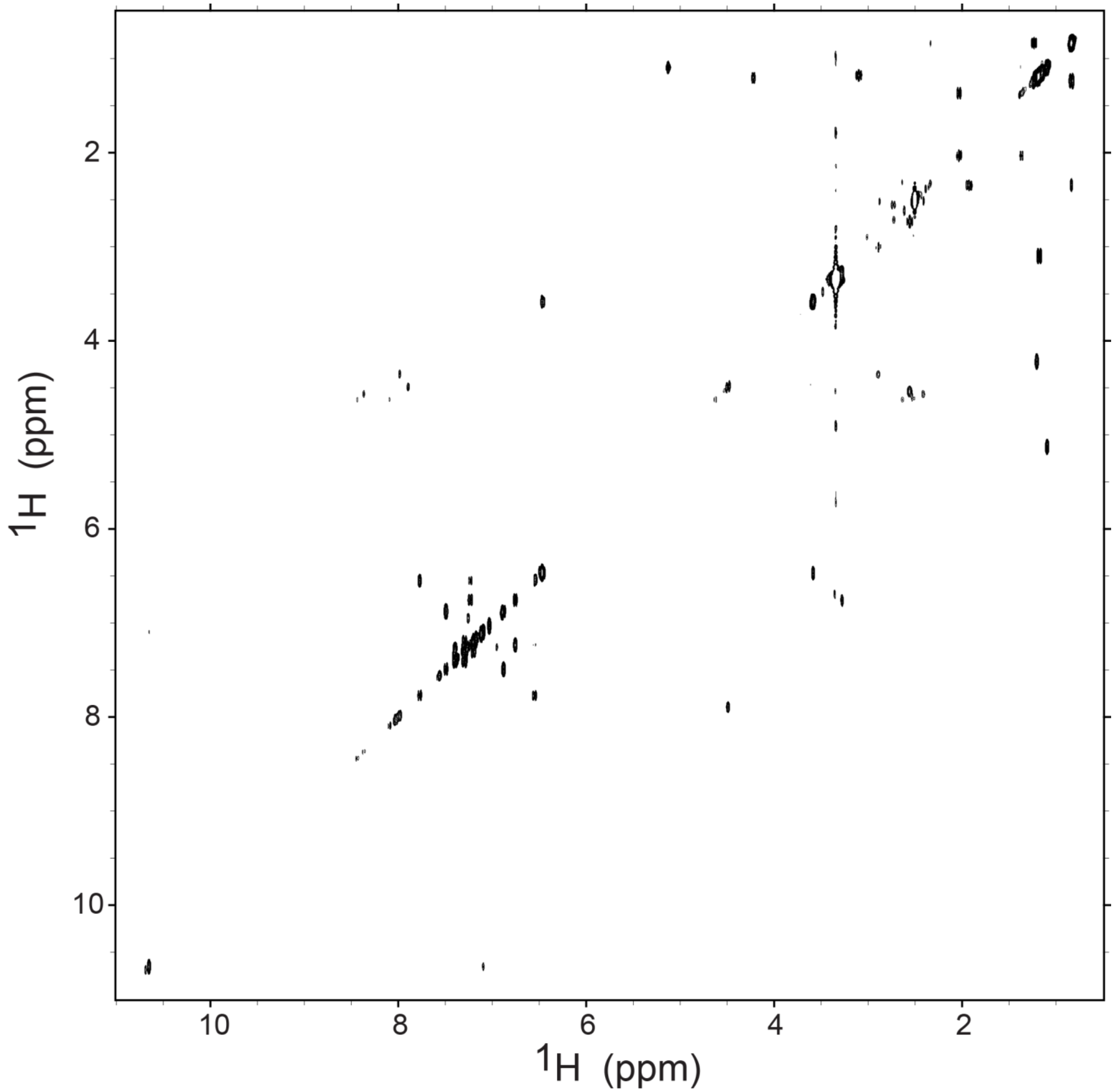

2D ^1H-^1H COSY NMR spectrum (d_6-DMSO, 600 MHz of ^1H) of ***C6*-22-Dap (54)** (~85% pure)

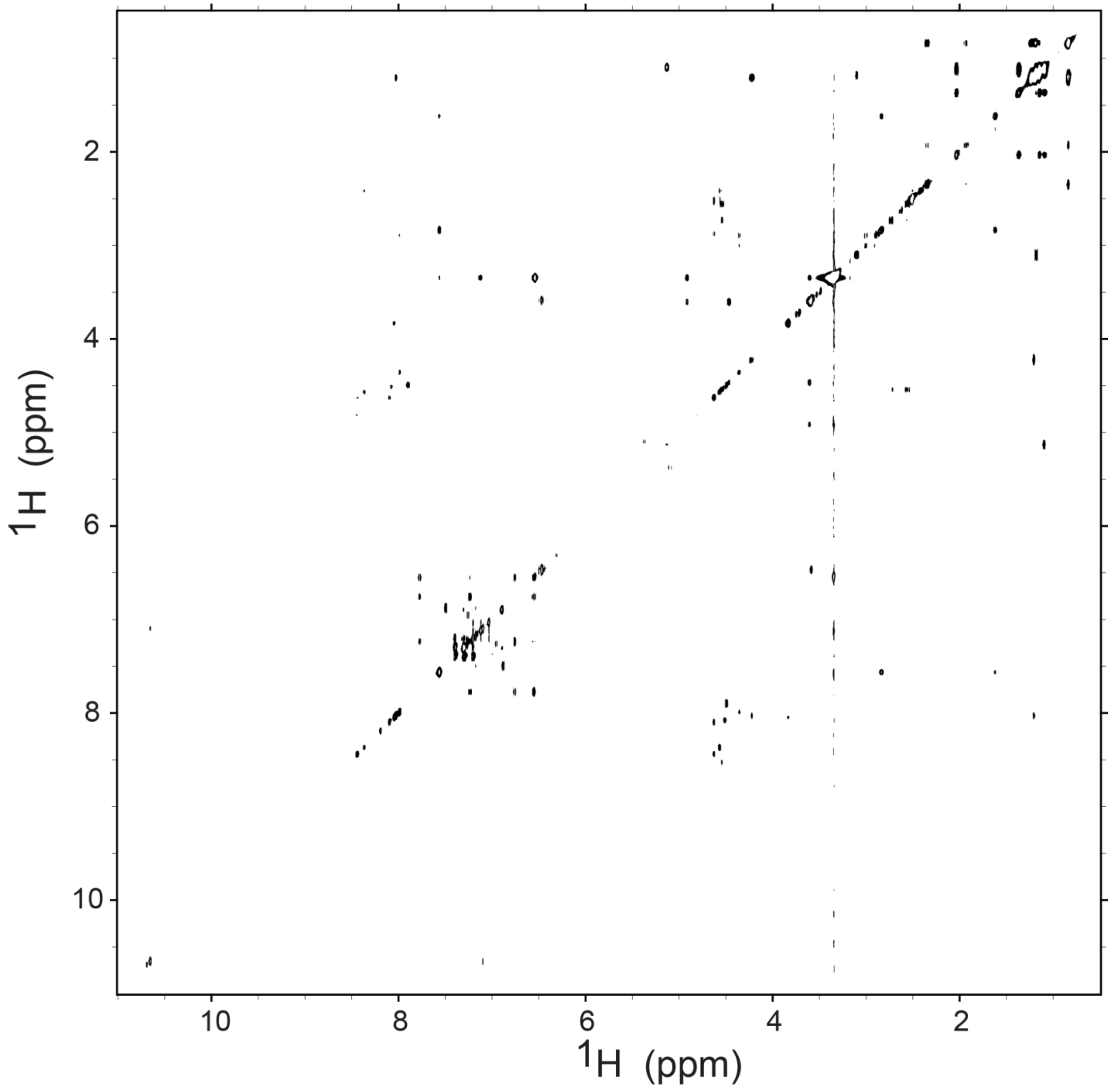

2D ^{1}H-^{1}H TOCSY NMR spectrum (d_6-DMSO, 600 MHz of ^{1}H) of ***C6*-22-Dap (54)** (~85% pure)

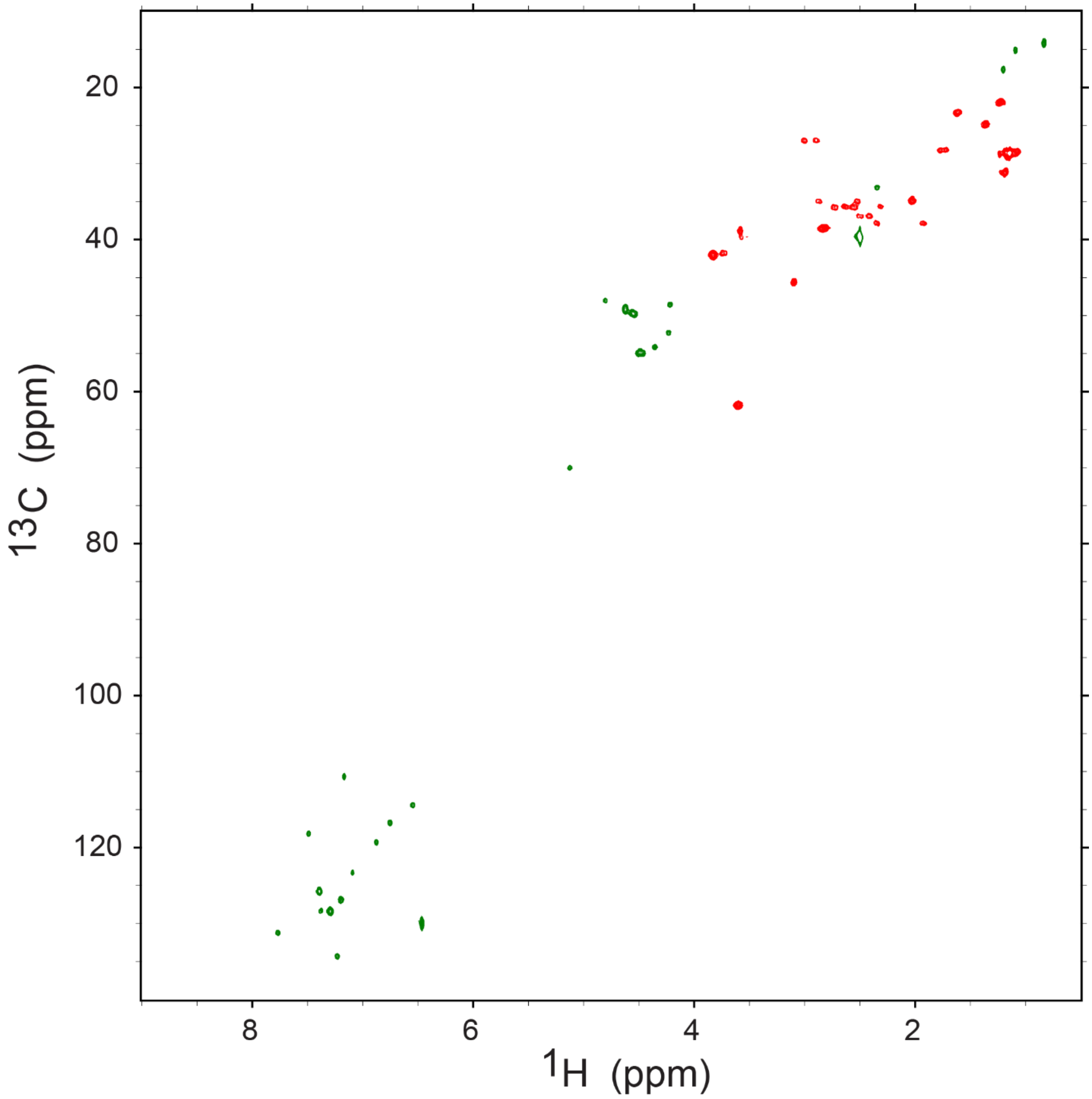

2D ^{1}H-^{13}C HSQC NMR spectrum (d_6-DMSO, 600 MHz of ^{1}H) of ***C6*-22-Dap (54)** (~85% pure)

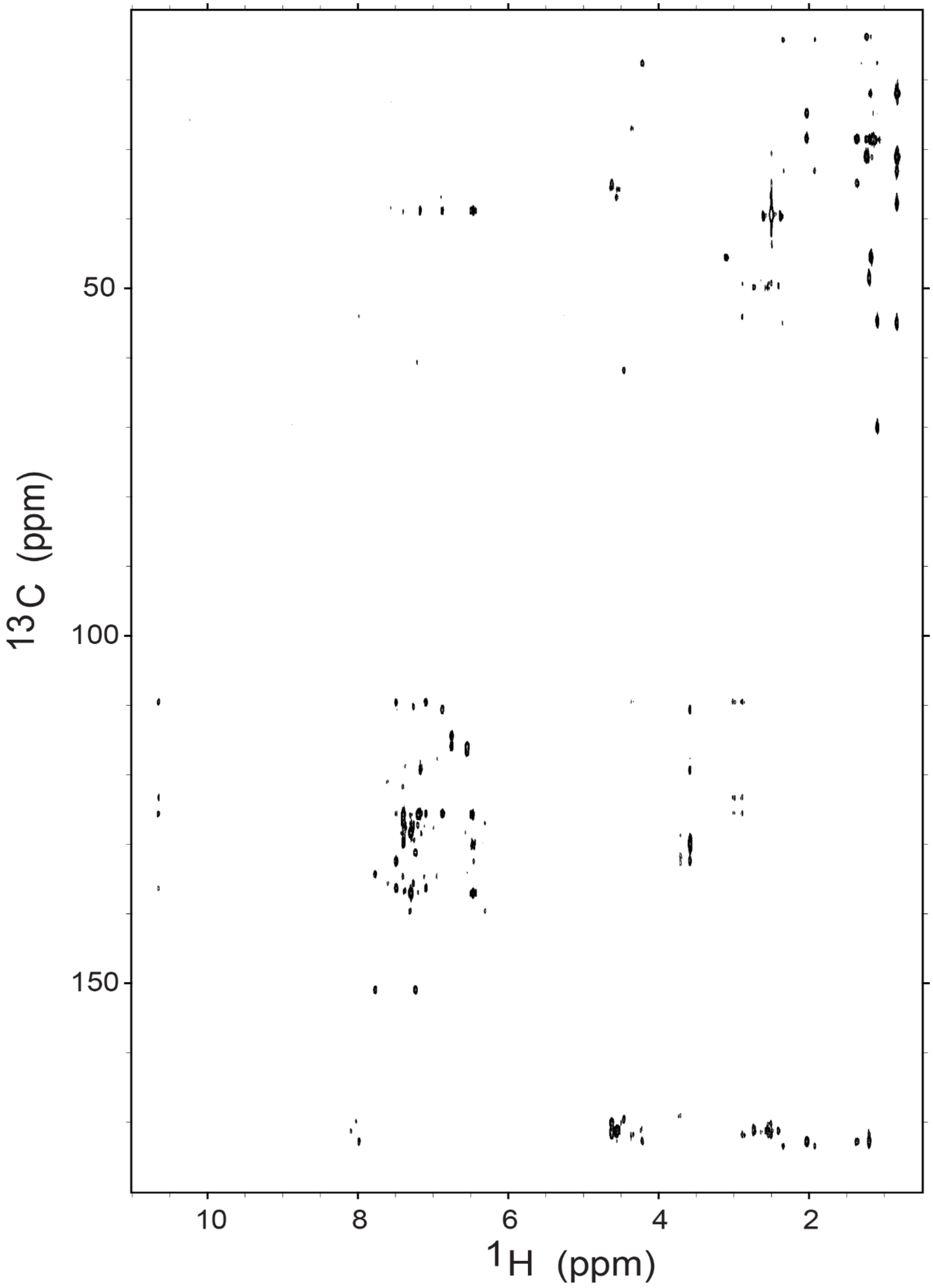

2D ^{1}H-^{13}C HMBC NMR spectrum (d_6-DMSO, 600 MHz of ^{1}H) of ***C6*-22-Dap (54)** (~85% pure)

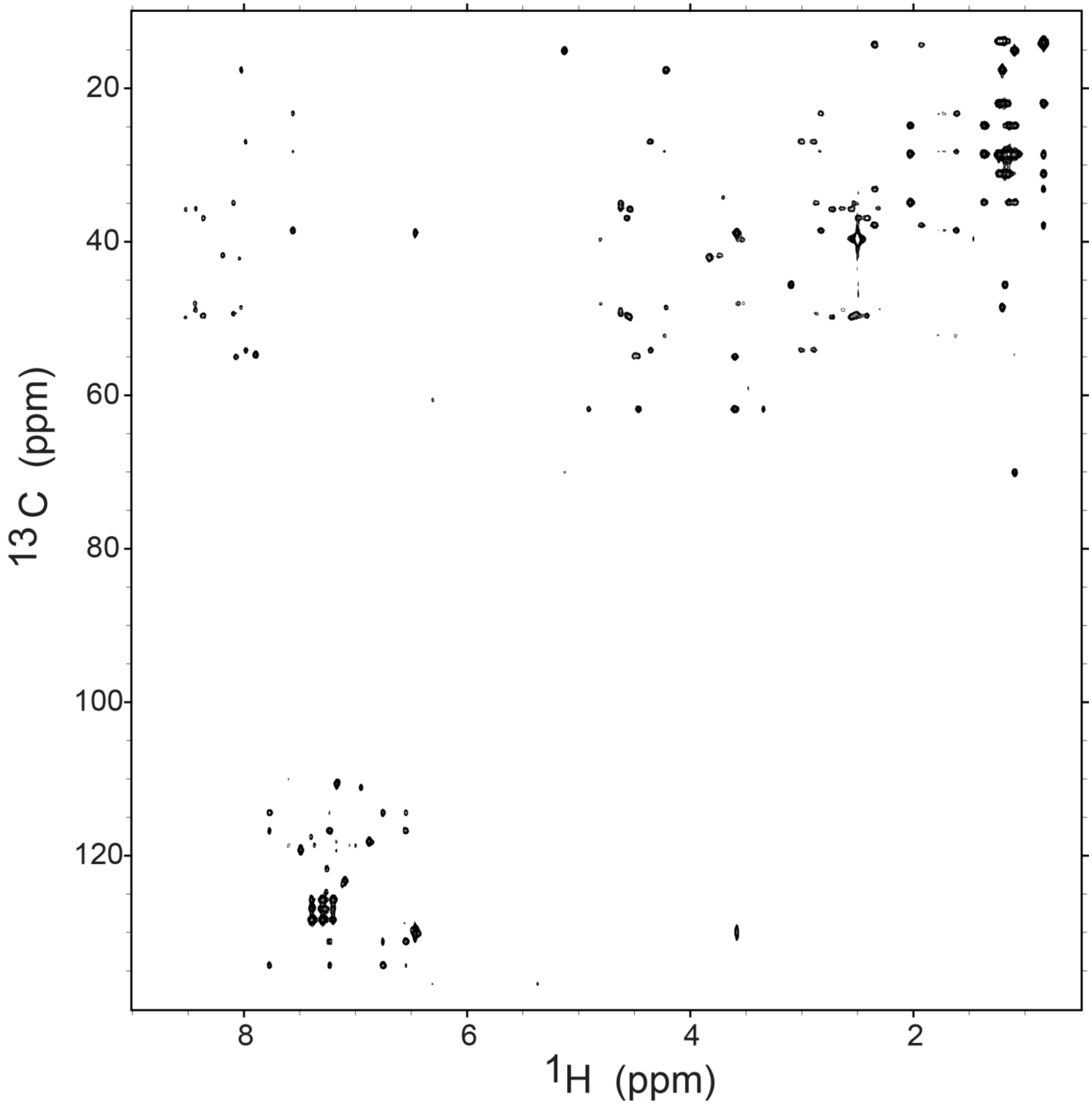

2D ^{1}H-^{13}C HSQC-TOCSY NMR spectrum (d_6-DMSO, 600 MHz of ^{1}H) of ***C6*-22-Dap (54)** (~85% pure)

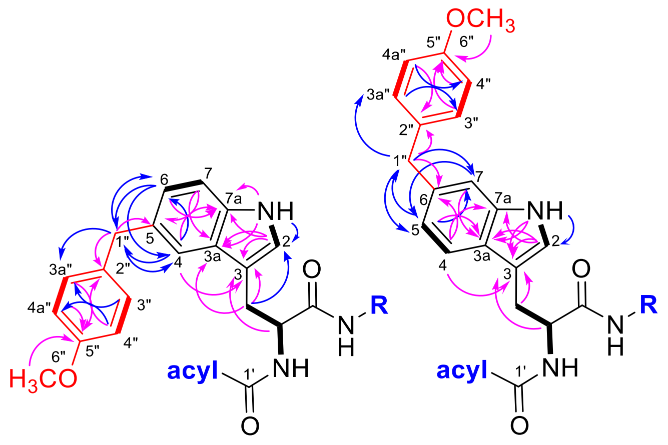

***C5*-32-Dap**
75 %

***C6*-32-Dap**
25 %

(55)

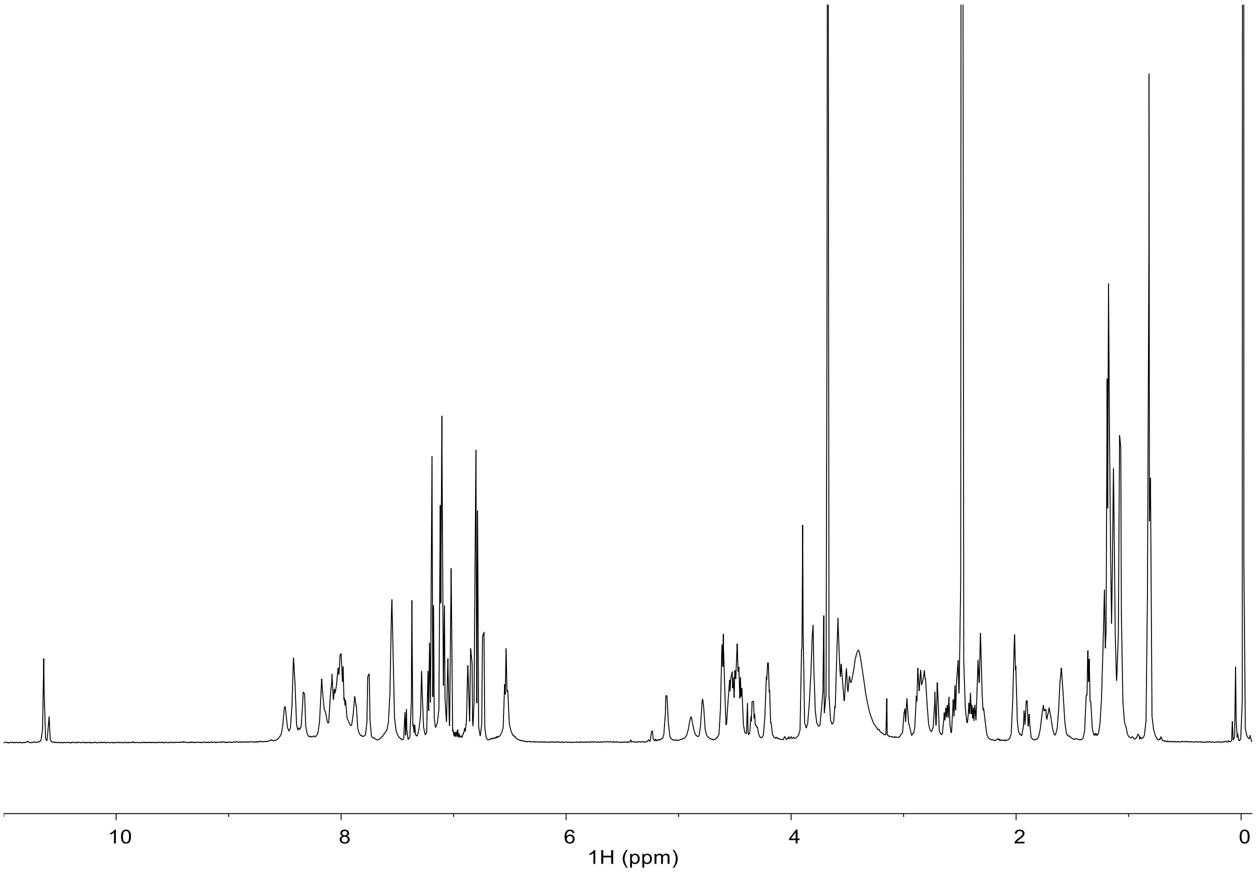

1D ^{1}H NMR spectrum (d$_6$-DMSO, 600 MHz of ^{1}H) of mixture of

0.75:0.25 of ***C5*-** and ***C6*-32-Dap (55)**

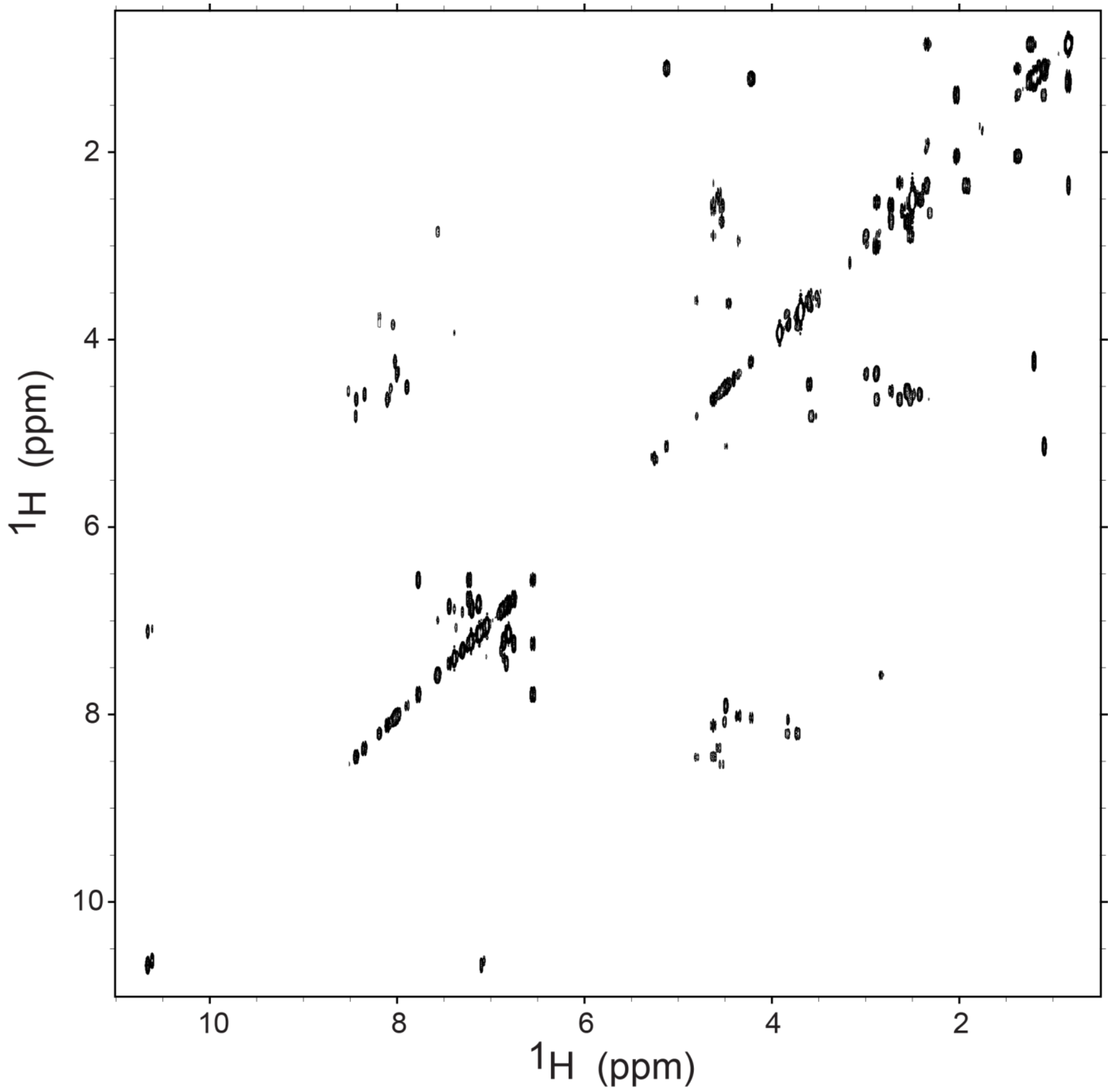

2D ^{1}H-^{1}H COSY NMR spectrum (d_6-DMSO, 600 MHz of ^{1}H) of mixture of 0.75:0.25 of ***C5*-** and ***C6*-32-Dap (55)**

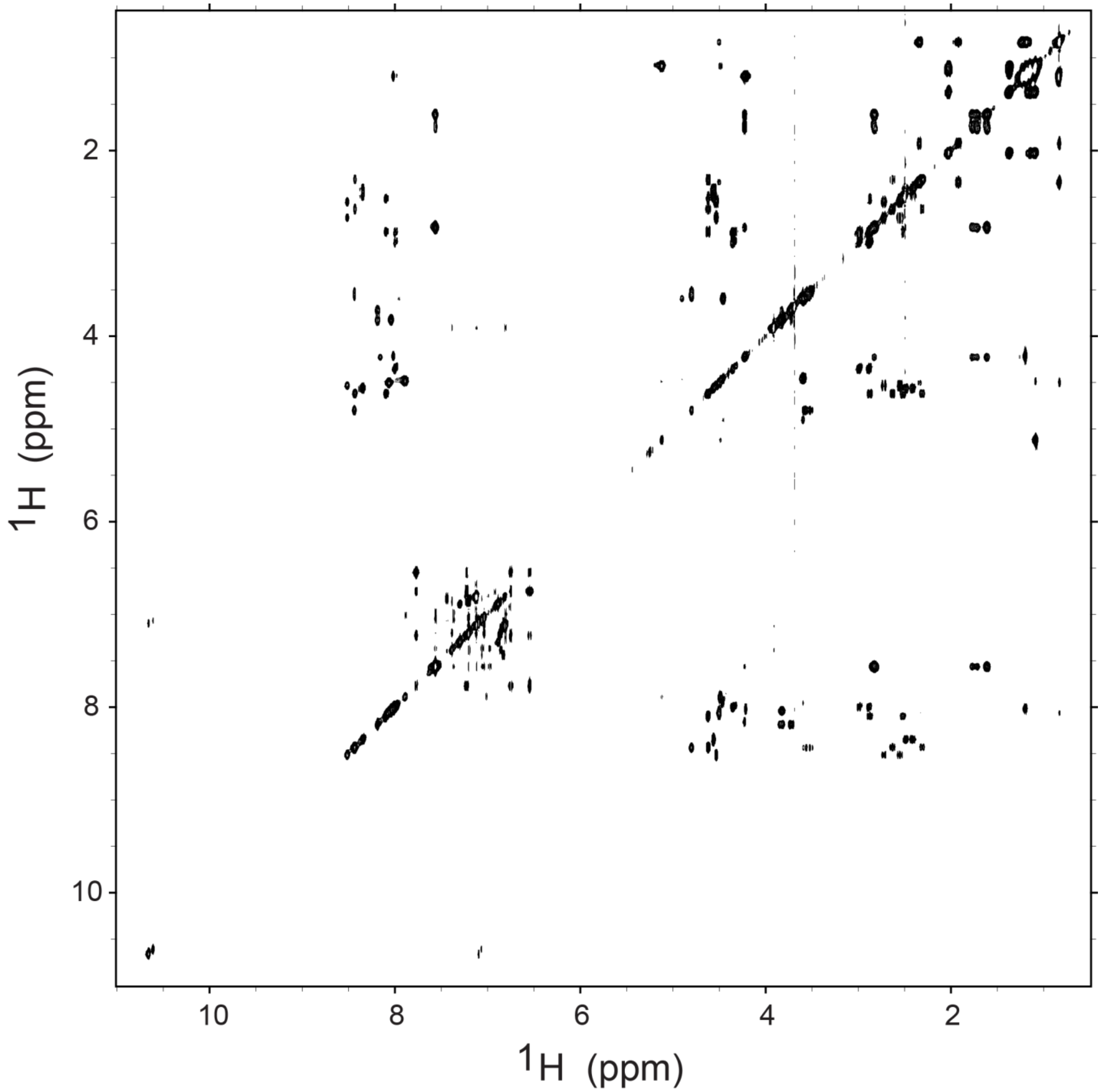

2D ^{1}H-^{1}H TOCSY NMR spectrum (d_6-DMSO, 600 MHz of ^{1}H) of mixture of 0.75:0.25 of ***C5*-** and ***C6*-32-Dap (55)**

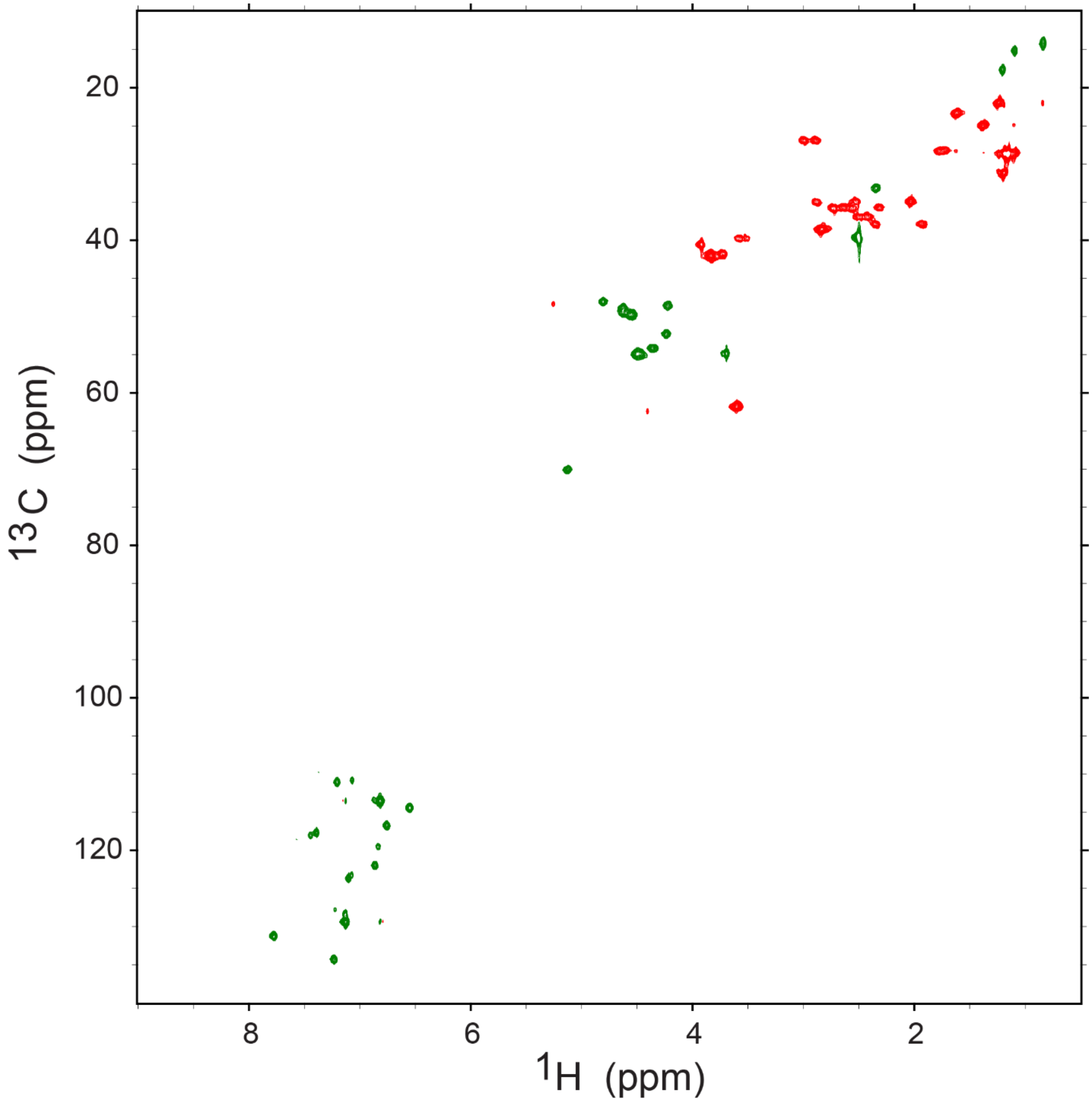

2D ^{1}H-^{13}C HSQC NMR spectrum (d_6-DMSO, 600 MHz of ^{1}H) of mixture of 0.75:0.25 of ***C5-*** and ***C6*-32-Dap (55)**

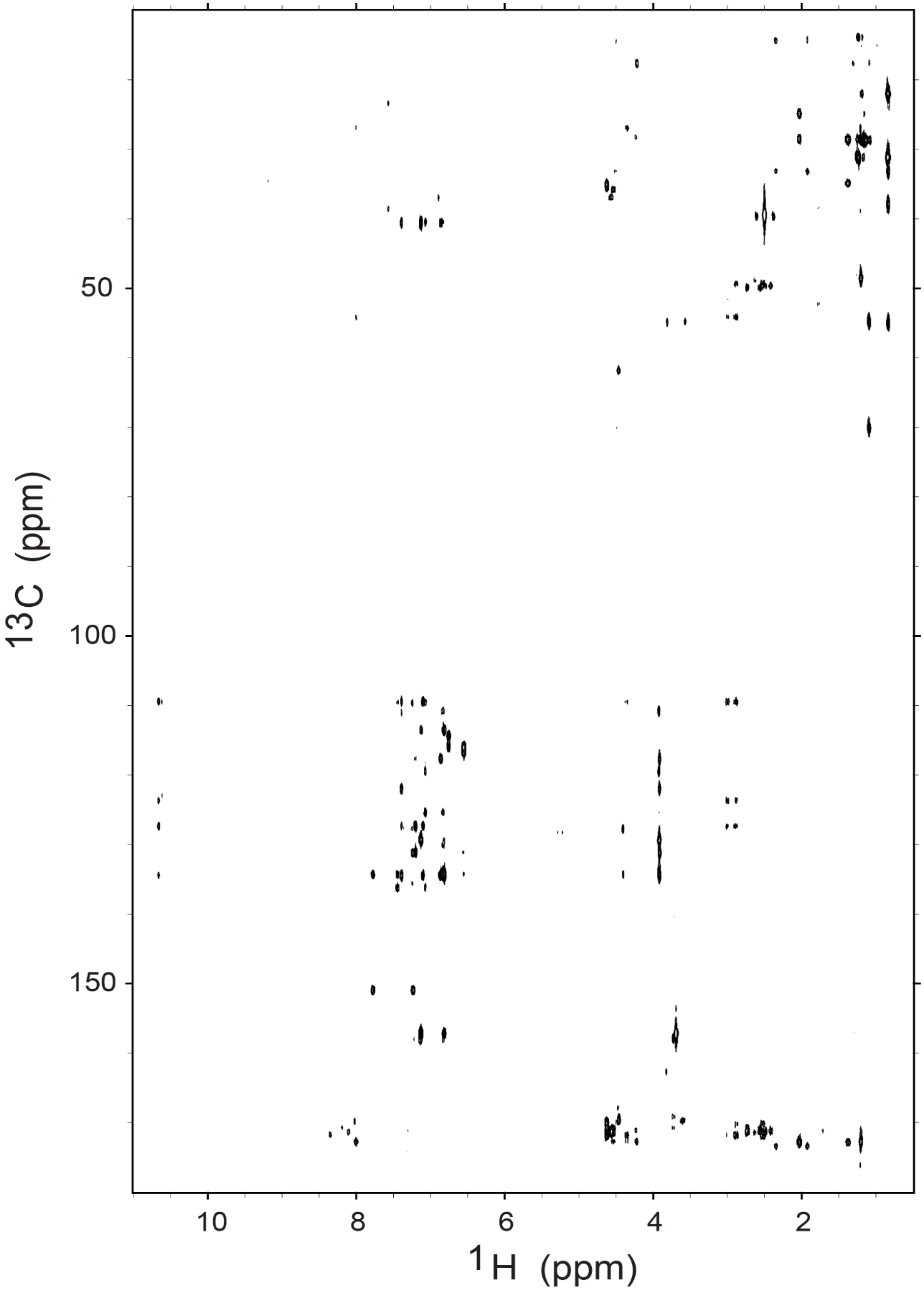

2D ^{1}H-^{13}C HMBC NMR spectrum (d_6-DMSO, 600 MHz of ^{1}H) of mixture of

0.75:0.25 of ***C5-*** and ***C6*-32-Dap (55)**

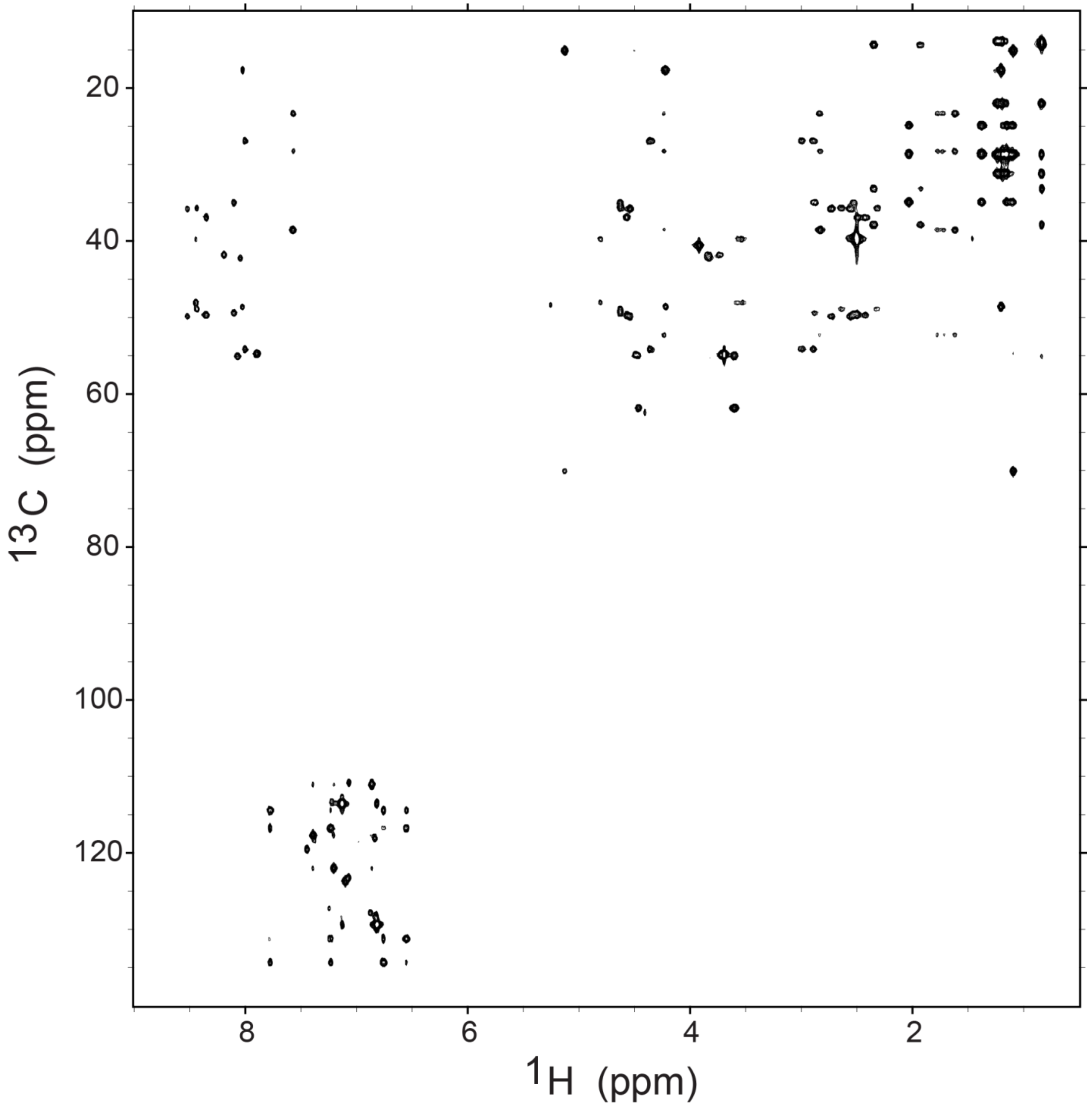

2D ^{1}H-^{13}C HSQC-TOCSY NMR spectrum (d_6-DMSO, 600 MHz of ^{1}H) of mixture of 0.75:0.25 of ***C5*- and *C6*-32-Dap (55)**
